# Supplementary material for: Optimising reporting of adverse events following immunisation by healthcare workers in Ghana: A qualitative study in four regions
Source: PLoS One. 2022 Dec 20;17(12):e0277197. doi: 10.1371/journal.pone.0277197 (PMC9767370; doi:10.1371/journal.pone.0277197)
Supplement: S1 Data — (ZIP) [file pone.0277197.s001.zip › Minimal data/S5 Why caregivers don't report.docx]

**Name:** 11. Why caregivers don't report

**Description:** This node contains all information on why caregivers do not report AEFIs to health workers.

<Internals\\IDI EPI\\GAEPI_01> - § 1 reference coded [6.47% Coverage]

Reference 1 - 6.47% Coverage

I: alright. ‘erh’ please sir we will also like to know what might be the reasons why caregivers do not report AEFI to health care workers

P: yeah, one of them could be that I mean if a care giver is not told about any possible things can happen after the immunization, they will not come or they might see it as a, as a normal thing. Oh! My child, I mean ‘erh’, they get fever when they get vaccinations so the child has got fever so it’s normal. Or they don’t even know that they should even report so those are some of the ‘erh’ I mean, possible ‘erh’ I mean things that is there.

<Internals\\IDI RHMT\\GARI_01> - § 1 reference coded [10.24% Coverage]

Reference 1 - 10.24% Coverage

I: so now we want to know why do you think some of the reasons care givers do not report AEFI cases to health care workers

P: oh! Perhaps they take it to be normal because like I said any intro, anything, any foreign thing that they introduce into your system you will react. For example, there are certain types of food you would want, want to eat the moment [claps hands] you take it you will react. And it could be transient, it, it could be, could also be fatal. Some people when you take it, you will react you can even get ‘erh’ [inaudible] rash and that one [rubbing palms together] it is not ‘erh’, ‘erh’ transient. It is ‘erh’ moderate this thing ‘uhuh’! Some people react fiercely and the normal one is the feverish conditions and where you will find most of our staff tell…when they go home and then you realise the baby is a bit ‘erh’ feverish just, start paracetamol syrup, but they use to tell them, if you give and still then just report and like I’m saying everybody is in the know that from the day you administer, you count from that day; one to twenty eight days. [Clapping] Anything that happens within that time, you cannot say because it is five days so it is not ‘erh’ adverse event. They should know that it is within…yeah. After twenty eight days you can say it is any other thing. Because we have had examples of ‘erh’ people even dyeing. It takes some coincidence and we have to post-mortem. For example, LA ‘erh’ Hospital, they did it, somebody died and…yeah.

<Internals\\IDI RHMT\\GARI_02> - § 1 reference coded [8.07% Coverage]

Reference 1 - 8.07% Coverage

I: so we will like to know next is, what do you think are some of the reasons why caregivers do not report AEFIs cases to healthcare workers?

P: ‘*erhm’*, number one most of them don’t also know what an AEFI is and then number two ‘erhm’ those who know also think that is normal. You know, most of the time we tell them that when you come for vaccination we tell… the child will have some fever and there will be pain and slight fever, slight pain. So when they go and is even severe they think it is normal, so they don’t come. So sometimes its ignorance and some of them too coming back to the health facility is a problem for them. Yes! Probably, may be transportation cost and all that or fear of coming to the hospital. You comes to the hospital, she have, she comes they will say go to the hospital. She is now going to make a card and then move around. Go here, go there, they think is cumbersome so they just get something for the child and they are okay and they won’t come back to us

<Internals\\IDI RHMT\\NRRI_01> - § 1 reference coded [7.60% Coverage]

Reference 1 - 7.60% Coverage

I: “Arr”, so we were on the reasons why caregivers don’t report AEFIs to health care workers.

P: One of them I say is fear, fear of been scolded, you know sometimes when the send those things to our health workers on the ground, the way they are treated is know they even turn to be afraid for reporting such things because they don’t want to be scolded.

I: Ok.

P: “Yaah”. And one is sometimes it is not made clear us to weather they are coming to pay for whatever service or not so it scares them away even if they have such things.

I: Ok.

P: If she can add on?

P1: (Laughing), you have already mention them and AEFIs, you see is it depends on we the staff telling them they women or not letting them be aware of where to report. Supposing I come for the immunization am given, the child is given something I go back, temperature and other things that should have been reported, they just take them as normal because they haven’t been told that if I go and this happens this and this happens they should come and report to the facility.

I: Ok.

P1: Is partly the staff problem.

I: Ok.

P1: For not able to tell the mothers to report back to us when they go back and am giving you this immunization when you go back and there is something happening they should come back and report .

I: Do you have more to add to that?

P: Why mothers do not come?

I: Yes.

P: Basically that is the revolving pint we are mentioning.

<Internals\\IDI RHMT\\NRRI_02> - § 1 reference coded [13.05% Coverage]

Reference 1 - 13.05% Coverage

I: Thank you. What might be the reasons why care givers do not report Adverse events following immunization to health care workers?

P: To?

I: Health care workers. That care givers do not report Adverse events following immunization to healthcare workers, why the mothers don’t report?

P: sometimes they may not report because the will not even know that it is an adverse effect from that vaccination err the child receive or been received.

I: Ok.

P: Especially if “ehh” they were not educated or communication didn’t go on well with “errrr” after, before vaccinating them.

I: Ok

P: You know but if for instance the information was given that, after this vaccination if you happens to experience ABC signs and symptoms report, probably the will report. And so if I just come and then you just vaccinate me without giving me information on adverse events I may not even know that the err adverse effects err that am experiencing is from the vaccine I received so that is one reason why the will not report. Another “errr”, issue is that err, probably if they had, if you report and you don’t get the necessary support “emmmm”, and then another time somebody is reacting know we say “ayiiii”, this people “koraaa” when you, you go the wouldn’t give necessary support, you even go and be paying mean while they immunized you but we all know that when somebody err gets a reaction we don’t have to ask for money from the person to actually attend to the person, we just need to do what, to just go ahead and treat and manage.

I: Ok.

P: You understand but if they have experienced 1or2 cases they were not supported at the facility level to get the management that the need, probably they will not report that is what I think “eerrrrr” is the reason why community people “aaaa” not report, probably we don’t educate them well. if we educate them it means the education is not enough and more so the attitude, our attitude too can also let them stay back because of fear that if you came to say that is the drug that you vaccinated my child, my child vaccinated may be that is making me have “ayiii” fever, itching, they may not know, our attitude may not, sometimes scares them away, because if we are not nice, if we are not nice to you, you know and something is happening, you may not feel free to come closer to me to discuss “errrr” your problem so that is what I can say.

<Internals\\IDI RHMT\\UERI_01> - § 1 reference coded [5.46% Coverage]

Reference 1 - 5.46% Coverage

M: on the part of the health care provider what might prevent that person from reporting on AEFI’s?

R: they themselves don’t know that there is something like adverse event which must be reported. The other thing is if the reporting forms are not readily available for them to complete and they will have to go and look for them somewhere then they will not report or some it is attitude they will not just do it not that they don’t know and sometimes to get a feedback. I mean it is also important so if they don’t get all these then they will not report.

<Internals\\IDI RHMT\\UERI_02> - § 1 reference coded [7.03% Coverage]

Reference 1 - 7.03% Coverage

M: What might be the reasons why care givers do not report AEFI’s to health workers?

R: For me I think I said that sometimes earlier, my experience tells me that it is misunderstanding. What actually is an AEFI? We have different levels of understanding. Somebody thinks it has to be something very unusual about the vaccine before we would say it is an AEFI but the definition says anything at all. If the manufacture says if you have fever you could have this or that. They are adverse events. When we say adverse event, it means opposite of what is expected. We are not vaccinating you to have fever, run diarrhea. So it is because of the little understanding about what constitute AEFI and then we tried to draw causality even at the point when it is suspected because you don’t begin to say it is normal of the drug or vaccine. The moment you say that, there is likely for them not to report. That it is only fever or malaria. We want to see something like an affective shock or something very serious then we say it is, but there is a criteria to determine which one is severe and not all that. So my thinking is that the level of sensitization is low and the level of understanding regarding what constitute AEFI is on a bit of the low side. That is what I have seen

<Internals\\IDI RHMT\\VRRI_01> - § 1 reference coded [18.91% Coverage]

Reference 1 - 18.91% Coverage

I: ok, alright, thank you very much. Emmh, now let’s delve a little bit deeper as far as the reporting is concern. What do you think accounts for the low reporting that we, we. we are witnessing on adverse events?

P: Errh, for the low reporting, like I mentioned, one the staff at the facility level that carry out the vaccinations, errh, their confidence level is low.

I: ok

P: Because some have the feeling, may be if I report something they will feel that I’m not doing the right thing

I: ok

P: because they not trained on it.

I: ok

P: Because they are not trained on it. So I think errh there should be a proper training for all the people that do the vaccinations so that they would be able to report.

I: Ok.

P: Ahaan, because the system is there, the reporting from facility to district, errh sub district to district to region, national. The system is there but sometime how to monitor it because some, some, may be like the clear definition, what to report, some of them may also not be too sure on some of the things to report. So I feel errh a lack of tracing of the staff that actually do the vaccinations on AEFI is one of the reasons we having how reporting rate.

I: ok. Any other please?

P: Emmh and other ones too, is like maybe like the communication aspect.

I: ok

P: That to sensitize caregivers about the AEFIs because all you know, some of the caregivers too if there is AEFI all that they do is…., if is even severe, and they have not reported they feel ok; I have gone to the vaccination session and this happen next time I will not send my child. Because they also don’t know that may be this and this are the things I have to report on.

So the second thing I will think is errh, we should also errh think of sensitizing the caregivers.

I: ok.

P: Ahaan on the AEFI. When they know that, ooh these things can happen, these ones are expected, though they talk to them at the clinics but I don’t think those education is errh, very, very errh, enough or adequate,

I: ok

P: so there should be more or we should step up education on the AEFIs to caregivers.

I: ok. Emmh, does your unit

P: yes

I: have in place the mechanism to monitor AEFI cases in the region?

P: errh, yes, we have a mechanism but hern, with challenges.

I: ok.

P: nhmm, we have the mechanism with challenges.

I: What are they please?

P: Because every, every year, we put in plans for monitoring

I: ok

P: ahaan, but sometimes you, the times you would want to go for the monitoring, the funds are not available for you to go and do the monitoring. So I think that’s one of the challenges.

I: ok. Ammh, could that also be a factor, contributing factor to the low reporting from, from the facilities?

P: That we are not able to do monitoring?

I: yes

P: Errh, yes it’s also part of it.

I: Ok

P: Because if you visit the district that may be you have to get at least one or about 10, a district is supposed to get about 10 and you report only one or two, what are the challenges? If we also visit them and they also look at the sub-district, may be this sub-district you are suppose get at least three in the year. So what are their Challenges? I think the supportive supervision also plays a role.

<Internals\\IDI RHMT\\VRRI_02> - § 1 reference coded [5.47% Coverage]

Reference 1 - 5.47% Coverage

I: Ok. And again with respect to the mothers, the caregivers could there be a reason why there is low reporting, from their side?

P: Oh, I’m sure they do report. It is our nurses may be they think if after immunization and the child have, has a fever is normal so they will not report such a thing to the next level.

I: ok

P: I’m sure mothers, if they find that there is problem, they send the children back to the clinic because that’s the education we give them. That after immunization, if there is any adverse effects or any change in the situation, they should allow the nurses to know about it. So I think the mothers don’t hesitate.

<Internals\\IDIs DHMT\\GADI_01> - § 1 reference coded [12.18% Coverage]

Reference 1 - 12.18% Coverage

I: Can you please tell me the reason why some care givers do not report AEFI to health workers?

R: What I may say is that, the nurses let the parents know what will happen and some go ahead to prescribe paracetamol syrup and other first aids, so when the child gets fever they are like give paracetamol for three days and after the days when it is gone they don’t come to report, some too when they come to report there is the fear that I gave an injection and this adverse event has happened so it means am not doing my work well and some also, the mothers this is my thinking they are afraid if they report the child might go through some procedures they the parents are not ready for. (Door opens and …… pauses)

<Internals\\IDIs DHMT\\GADI_02> - § 1 reference coded [7.19% Coverage]

Reference 1 - 7.19% Coverage

I: What may be the reasons why some care givers are not reporting AEFI to the health workers?

R: I think it’s the post education after immunization, they just advise the mothers to give paracetamol after every injection and more so because I don’t deal primarily with them I cant tell what could be the reasons why. I have not had any feedback from the nurses of any case.

<Internals\\IDIs DHMT\\GADI_03> - § 2 references coded [19.58% Coverage]

Reference 1 - 7.16% Coverage

I: So what could be the reasons why some caregivers do not report to the health worker?

R: Ok with the mild ones of course, care givers will just think it’s just a transient something and not pay much attention to it, secondly some of the care givers may not be informed that when such events happen they should report back to the nearest facility, basically I think this is the reason why they might not report, its either they don’t know they are to report especially with the mild reactions so they don’t see the need to report.

Reference 2 - 12.42% Coverage

I: What might be the reason why the health workers who encounter AEFI may not be reporting?

R: With the health workers it is a challenge because we have trained them, with my former station we have had trainings and subsequent refresher training on the need to complete the forms so that when the mothers come in with complaints after the vaccination, why they are not reporting I can’t tell either to just putting it down to commitment, they are not committed because the forms are available and they have been oriented on the needs to have the form completed so I would say it’s just lack of commitment to it, some of the health workers to think the signs are just transient and of no importance, they don’t see the need of what the care giver is complaining about as a warrant for he or she to fill the form to that effect, so this are the issues importance that is placed on the reaction the mother is talking about.

<Internals\\IDIs DHMT\\GADI_04> - § 1 reference coded [12.98% Coverage]

Reference 1 - 12.98% Coverage

I: What are some of the reasons why the care givers are not reporting AEFI to the health workers?

R:Maybe as care givers we don’t take time to explain things to them, for instance BCG is given and they have to explain to them that this , this is what will happen and tell them what it is supposed to prevent, in a week’s time you will have that blister, in a month’s time it will form a sore, maybe when you find something which is not in line with what am telling you report, we don’t spend time to explain to them and that is my problem, I am trying to talk to my nurses but basically we are not able to let them know when they come, on the other hand some come because they had swelling, and I think they should be informed promptly so they can also report.

<Internals\\IDIs DHMT\\GADI_05> - § 1 reference coded [10.78% Coverage]

Reference 1 - 10.78% Coverage

I: So what are some of the reasons the care givers may not be reporting AEFI to health workers?

R: Lack of education on how to report and also when they report who pays the bills, when I come nothing will be done or I have to come and pick folder, see the doctor and the cost who bears it and then also I would say the feedback service providers don’t give feedback if sometime happens there should be a report back and we are also fond of home care when someone is sick we tend to manage and I think we must stop.

<Internals\\IDIs DHMT\\GADI_06> - § 1 reference coded [6.57% Coverage]

Reference 1 - 6.57% Coverage

I: What are some of the reasons why some caregivers do not report to health workers?

R: The reasons they vary, when health workers don’t give them the right information they tend not to report, then the client care givers relationship, the caregiver may say now that am coming to report won’ the health worker shout at me, so they choose who to give information to, that one too is there and also the care givers may not have time with their wards early morning they are gone to work then they come back late and the child might be in the hands of some care taker who will not even look at those things, and those things will delay the reporting of the AEFI.

I: Are those all the reasons?

R: Yes and also the attitude of the health worker, if the knowledge is not passed down well that one too is a problem, they will not be able to identify to report.

<Internals\\IDIs DHMT\\GADI_07> - § 1 reference coded [7.20% Coverage]

Reference 1 - 7.20% Coverage

I: thank you. So ‘erhm’ we would also like to know what might be some of the reasons why care givers do not report adverse events following immunization to the health care workers?

P: ‘erhm’ some of it I attribute it to attitude yes. ‘erhm’ some the, some of the reasons they give is that they were busy and then sometimes they also say that the complains from the guardians and mothers are sometimes insignificant but we impress upon them that ‘erh’ when there is a complaint from a guardian or a parent, it is not insignificant. Once she has found it necessary to complain, we must listen to it and look at it ‘erhh’ so that we can save lives.

<Internals\\IDIs DHMT\\GADI_08> - § 1 reference coded [8.24% Coverage]

Reference 1 - 8.24% Coverage

I: We also want to know what are some of the reasons why caregivers do not report AEFI cases to the healthcare workers?

P: ‘Hmmm’ what’s my problem now when I went round few, few areas to just talk to [inaudible] I think some of them don’t report to the health facilities because they thought it wise, they can take care of it at home. So upon doing that, if it’s gets out of hand they then report to the nurse maybe a week later maybe when they are coming for a review or something they will come and report it. So they don’t, and so the thing that we have to do know is to educate the people whenever we are doing immunization to them we have to educate them and tell them that if there is anything, I think that the education don’t go down well, if it go down well I think they will also rush and come and inform us whenever they have challenges.

<Internals\\IDIs DHMT\\GADI_09> - § 1 reference coded [7.32% Coverage]

Reference 1 - 7.32% Coverage

I: please we would also like to know some of the reasons why caregivers do not report these adverse events to health care workers?

P: they sometimes think it’s normal. They always think that ‘ooh’ when you give your child immunization they, by all means the child will be warm and but we always tell the nurses that the slightest thing that they notice about it they should tell us so that at least ’erh’ we will also forward to ‘erh’ wherever it’s supposed to go. Hmm! [knock on the door].

I: so like you were saying, you said they think it’s normal

P: yes! They think it’s normal with immunization so they do not report unless it’s very severe. May be they go and there is an abscess formation or something, then that one they will come. Otherwise, they always think that it’s, it’s normal and I think sometimes the way we also tell them, that when you take the immunization you have to take paracetamol and sponge the baby and all those things. So they think it’s something they should expect. Yes.

<Internals\\IDIs DHMT\\GADI_10> - § 2 references coded [16.02% Coverage]

Reference 1 - 9.95% Coverage

I: So, thank you! We would also like to know what might be the reason why care givers “erhm” do not report AEFIs to the health care workers

P: “Erhm”, sometimes it could be that ‘erhm’ probably someone went in for ‘erhm’ a shot or a vaccination and the child experienced ‘erhm’ any form of pain. Already in our setting, it’s more like a rural setting and you know that in such settings most of the times they don’t want to even come to the hospital. Well they have their own perceptions about what goes on in the hospital doesn’t ‘erhm’ match their beliefs sometimes. So if they come and something happens they wouldn’t even want to go back then they would want to let’s say resort to some a traditional method of solving that problem. It could also be that ‘erhm’ if someone has experienced it the first time, let’s say, first child experienced it then ‘erhm’ it’s he or she went back to the took the child back to the hospital and goes for second immunisation or a second child goes for or takes a second child for ‘erhm’ immunization once ‘erhm’ she knows that it happened to the first child and it resolved maybe it’s just pain she doesn’t bother to come over but we know every, every adverse effect must be reported regardless every whether we know that it will resolve or not resolve must be reported so sometimes based on ‘erhm’ previous experiences they wouldn’t want to bring it err the child.

Reference 2 - 6.07% Coverage

I: We would also like to know what might be the reason why health workers themselves who encounter AEFIs are not reporting?

P: ‘Erhm’, the main reason if you ask me will be their knowledge you know of even the fact that whether there is a reporting system where there are forms, the protocols involved. That will be one. The other one which is like a wild guess is ‘erhm’ it’s possible that well if someone may report and you because you gave the vaccination you will think that it might go against you, you are afraid to you know report and yeah you don’t want people to hear about probably what has happened and so let’s say, out of fear, you wouldn’t ‘erhm’ want to report so that could, that could be a reason. But I think the main reason would be that, err the lack of knowledge of the protocols yes in place so we would have to definitely intensify.

<Internals\\IDIs DHMT\\GADI_11> - § 1 reference coded [12.83% Coverage]

Reference 1 - 12.83% Coverage

I: okay, okay. So we would also like to know what might be the reason why the care givers, okay? ‘erhm’ do not report AEFI cases to health care workers

P: ‘hmm’ ah! They think some of the things are normal ‘uhuh’ but though they were trained on the job but still, so what we did now we chip something in. anytime we are doing some training we chip it in that they should report.

I: am talking about the care givers; the mothers

P: the mothers?

I: yeah. What, what, what is the problem, like the reason why they are not reporting more to the health care workers

P: ‘hmmm’ ohhh. They report. The few that ‘erh’ get the adverse events, they, they report like swollen on the thigh and then…as for the fever because at the immunization centre they educate them; if your child have fever you should do A, B, C, D. So unless is more than [inaudible] [talking in the background] before they will come and report.

<Internals\\IDIs DHMT\\GADI_12> - § 1 reference coded [7.33% Coverage]

Reference 1 - 7.33% Coverage

I: Alright so the next question says what might be the reason why caregivers do not report AEFI to healthcare workers?

P: Yeah, the caregivers some of them see as waste of time. Because the facilities, in fact from here to some of our facilities are far. Some of them, if we even take you to the last point…a mother will not even come from that point to this place, we were discussing it as an open forum now and then someone of the participant were saying that mothers, mothers prefer taking the, I mean using the concoctions in the house than bringing the children to the…, because some of them don’t have the money to even travel that long distance to the district.

<Internals\\IDIs DHMT\\GADI_13> - § 1 reference coded [11.01% Coverage]

Reference 1 - 11.01% Coverage

I: what might the reason care givers do not report AEFI’s cases to the health care workers

P: A care giver might not report An AEFI to a health care worker because, probably, not so sure…she thinks is, well my child has been given a shot and has been given a…has been vaccinated and this is something that I expect would happen so its normal you know? Some of them go home and when there is a swelling or something they try to manage it themselves. They put all sort of things on the site; kerosene, sheabutter, whatever. Sometimes… every time a child is vaccinated the community health nurse tells the mothers, if you find anything bring the child back. If there is a swelling, if there is pain on at the site of injection, you know. There are certain things you might expect, you know and so I think that sometimes the mothers think well is normal so they keep it to themselves. And sometimes distance, access is an issue. I mean, you bring the child for vaccination on a certain day and if they find anything wrong, coming back to the clinic to come complain or getting access to the health care worker to make a complaint is a challenge because of…because access is a challenge. Because you need money to board a vehicle to go to where the health care worker is. We are doing CHPS anyway but we still have access issues. That’s what I will say.

<Internals\\IDIs DHMT\\GADI_14> - § 1 reference coded [13.70% Coverage]

Reference 1 - 13.70% Coverage

I: So please I would also like to know ‘erh’ what might be the reason why caregivers do not report ‘erh’ AEFI to healthcare workers?

P: As much as I know caregivers report but sometimes they come after ‘erh’ my baby was immunized, normally before they are immunized they are educated worse things they should expect. If they are going to be given BCG they will tell you the, the, the, the site will swell, you shouldn’t rub it, don’t generate heat, it will form ulcer don’t put anything on it and then maybe if they are given another medicine like measles, you child might run fever if your child runs fever [inaudible] on your child give anti-biotic. So they are given that education before they go and so if they are not reporting ‘ah’,’ ahhh’ I will say that because they have been, their minds have been conditioned to expect something so if the thing has occurred what’s alarming…but there are some if is ’erh’ [inaudible] they will, they will come and report but if it’s within what it is to be expect having their minds conditioned, this is what will happen when it happens do this. So because their minds have been conditioned, if they are not coming it’s because we condition their minds as to what might happen.

<Internals\\IDIs DHMT\\GADI_15> - § 1 reference coded [9.78% Coverage]

Reference 1 - 9.78% Coverage

I: having said that, what might be the reasons why caregivers who encounter AEFIs don’t report them to the health care workers?

P: what I was just discussing with one of my community health nurses, what I have, I have, I have noticed and what is going on is that when they go out to the field to give immunizations, they do health education, so the one at the immunisation who provides the immunization service do educate the mothers on what to expect. So it’s like they tell them what to do and then they tell them to report uhuh…so I’m sure because of the knowledge of the mothers. unless may be there is abscess has formed, but the common ones like the management of the fever and they have, they’ve taught them how to do it so they are able to manage it but if there is anything they cannot do then they can now report to them and then refer them to the facilities

<Internals\\IDIs DHMT\\NRDI_01> - § 1 reference coded [14.11% Coverage]

Reference 1 - 14.11% Coverage

I: What might be the reason why caregivers do not report AEFI?

P: Sometimes some of them will say sometime too some staff maynot inform mothers about it and some of them too when they are even been informed but then they may not see it a need to come and report or the distance that they are because mostly is outreach centers they do go so some of them may feel reluctant walking to come and tell you that my child have fever or this they sit at home and give their own remedies.

I: For is it the health caregivers side that is accounting for it more or the the the parents?

P: I will say is both sides

I: Is a two way affair.

P: Is a two way affairs and the thing is that if the education is not continues it can also bring such a thing because we do have staff that come in, we train them on such things but then may be when they get to the field is not all the time they may be hammering on it, but then I believe that if the education continues at every outreach center you say it before, you council the mother before that then it will be more effective and mother would come and at times too the mother’s, some of them know they wouldn’t just come because they feel like the distance that they will walk to the health facility because the staff don’t stay there they go for outreach and when it happens they may not come and report to you or better still when they even come you the staff that may be went there they wouldn’t let you know they would either go to the clinical side for treatment without involving you in what is happening to the child so I think those are some of the things that sometimes make us not to report.

<Internals\\IDIs DHMT\\NRDI_02> - § 1 reference coded [15.03% Coverage]

Reference 1 - 15.03% Coverage

I: yoo another question I would like to find out is what may aa– might account for reasons why health care workers do not report AEFIs?

P: Eeem one I think that is a fairly new phenomena …

I: mm

P: In the past it wasn’t something that we were reporting on…

I: Ok

P: is a fairly new phenomena that is number one and number two most immunizations are done on outreach level where health staff go to the various communities to do the immunization and after that the health staff leave the place and then go back to their bases so if it occurs, I mean if it is the minor ones eeh the parents may not report at all they may not even come and report because aah eeh if it is just some slight fever may be on the day of immunization or the following day eeeh the parent may just give the child paracetamol and then the fever goes away so eeh it will not be aah they will not be motivated to travel all the way from wherever they are to the health facility to to to to come and report just a minor thing if of cause it is one of the major ones that may receive attention not it is just this minor ones may be excessive crying and then may be some small redness at the site of the injection some small redness at the site of the injection and whatever I,I don’t think that they will be motivated to come all the way to come and report. Eem em and with our current resource situation the resources that we are we have now struggling with is also going to be almost an impossible task to ask the health workers to go back to the place may be after a day or two to go and look for AEFI mm eeh I don’t think is not practically possible with the current resource we are working.

<Internals\\IDIs DHMT\\NRDI_04> - § 2 references coded [9.72% Coverage]

Reference 1 - 6.73% Coverage

I: What might be the reasons why caregivers do not report adverse events following immunization to health care workers?

P: Emmm sometimes you cannot … Go ... without, I mean you can say, we can say anything without talking about the attitude of health staff ……… because if you are, if you’re your rapor is good to the clients even their personal issue they will explain to you. But if your rapor your attitude towards the clients is not good enough even if you have done something wrong to them they can’t come closer to you because they are afraid that something else, you can, you can insult them. You can, you may not give them the chance to explain, so, clients or caregivers they fear nurses because of their attitude. Am not relieving myself there. So far as a still in the system we are all there but the, if the attitude is changed towards clients, even their personal issues at home they can come and tell us that ohh my husband did this and did that ehhrr so that you can you help them with advice or counseling but because of the negative attitude toward clients, shouting on them, they will call you, you don’t even mind them, you look superior to them when I actual fact you should look very similar, bring yourself down to them and then they they feel free to open up to you with discussions and all then then. So is the attitude that will let them not come.

Reference 2 - 2.99% Coverage

I: Any other reason?

P: Arrrh any other reason that I think will hold caregivers from not coming to share with us when they have these inci... incidence could be probably financial issues or distance. Somebody is staying far away from the community. ehh from the health facility when it happens, look at oh for me to move here to this particular facility oh there is a distance let me see if I can manage it at home or sometimes may be if I go they may say we should, we should they will admit us, do I have money? I don’t also have health insurance. So this are the things that always keep them at home.

<Internals\\IDIs DHMT\\NRDI_05> - § 1 reference coded [9.63% Coverage]

Reference 1 - 9.63% Coverage

I : So What might be the reason or reasons the reason or reasons why caregivers do not report adverse events following immunization to Health Care Workers?

P : Emmmm I must start by saying that emmm ...... one of the factors could be that ehhh Health Care providers do not make this known to the mothers. Because if the mother is aware ..... that if one or two happens they will have to go back or take the child to the care giver and then .... though some do, most of them do not, they also complain of time, the time to go and you know you are going for a session you don't have time you see a number of women their children and you are alone or one or two you have to fill several forms, and so the time to even talk to them which of course to me is not an excuse any way .... but they don't usually the button line is they don't usually tell the mothers. But even .... as at when even the mothers are put in the known ..... you naw some of them feel that since the child is suffering from that situation the system has failed and for that matter they will not want to go back they want to resort to their own way of trying to see how they can solve the problem ....... I think basically these are some of the challenges that will not make them to report ....... And even the fear that emmmm well I went there and it happened to my daughter or my son so why should I go back there again probable they may compound my situation for me and so I think basically this are some of the things I think that makes them in fact the critical one is that our staff don't sell the information to them, because before you administer any antigen the mother should aware of the particular antigen you are administering and the site and what it is going to protect or the disease condition is going to protect. But basically they don't tell them just because they want to hurry up finish with every body and leave.

<Internals\\IDIs DHMT\\NRDI_06> - § 1 reference coded [9.30% Coverage]

Reference 1 - 9.30% Coverage

I : So What might be the reason or reasons the reason or reasons why caregivers do not report adverse events following immunization to Health Care Workers?

P : Emmmm I must start by saying that, one of the factors could be that, Health Care providers do not make this known to the mothers. Because if the mother is aware that if one or two happens they will have to go back or take the child to the care giver and then though some do, most of them do not, they also complain of time, the time to go and you know you are going for a session you don't have time you see a number of women their children and you are alone or one or two you have to fill several forms, and so the time to even talk to them which of course to me is not an excuse any way but they don't usually the button line is they don't usually tell the mothers. But even as at when even the mothers are put in the known you naw some of them feel that since the child is suffering from that situation the system has failed and for that matter they will not want to go back they want to resort to their own way of trying to see how they can solve the problem ....... I think basically these are some of the challenges that will not make them to report ....... And even the fear that emmmm well I went there and it happened to my daughter or my son so why should I go back there again probable they may compound my situation for me and so I think basically this are some of the things I think that makes them in fact the critical one is that our staff don't sell the information to them, because before you administer any antigen the mother should aware of the particular antigen you are administering and the site and what it is going to protect or the disease condition is going to protect. But basically they don't tell them just because they want to hurry up finish with every body and leave.

<Internals\\IDIs DHMT\\NRDI_07> - § 3 references coded [16.62% Coverage]

Reference 1 - 5.68% Coverage

**I:** Thank you very much. What might be the reasons why caregivers do not report AEFIs to Healthcare workers?

**P:** Ah, you see, really traditionally what has been of practice is that, excuse me to say clients traditionally have been scolded, like after an immunization a mother comes to say, Oh yesterday as I had the immunization for my child, this is what has happened. Healthcare workers are quick to disperse the report, to say that, it is not as a result of the immunization, thinking that they are being, their works are being compromised; or for that matter are being blamed, while it is you who have done it. But the training now going on, it is making health staff to know that, No!, any client coming accept the client, whatever report the client brings whether related to the immunization or not, but once it is after your immunization that the person has come to complain, listen to the person and try to review the situation. So currently, that is what has being preached but this was not the. The Health workers were not listening to mother just coming to complain of anything related to after immunization process.

Reference 2 - 10.31% Coverage

**I:**  Do you have more reasons to give to that one?

**P:** Others reasons have also being that, initially our way of criticizing staffs for maybe after immunization, a report made that there is some effect so the system made it look like it you the giver, the healthcare giver who has cause that problem. So that also made people to feel that no, they, they should not also be held reliable for such damaging, by the because some of them felt that after all vaccine self is made by the vaccine component, the child is likely to have fever. So he has come and you now want to say that I am the one that the better, I don’t even listen to that report or I don’t accept the report. So that also a systemic problem on our side. And quiet apart from that, some of the reports coming would be so ridiculous. You see, like the person probably was having incubating let say malaria parasite and after the immunization then he gets the malaria condition, so if he comes and say that when I took you’re this thing and my child has gotten malaria. You know, you see that, you know you Gave a vaccine for preventing measles and the child comes down the next day with malaria and they are attributing it to your vaccination you see that it don’t click naturally you know that how measles vaccines how, what does it have to do with malaria. How? So naturally staff won’t take and we have seen such incidences but of late because of the awareness, you say , if the person’s child comes and you give the person the vaccination and the next day is down malaria and the mother comes and test proves that it is malaria, document it, reassure her, so now staffs too are comfortable that the malaria it has come, much as it is not related to any vaccination, but I have accepted that, I mean I have to be accepted to be of concern to the person and say that oh yes malaria has come. You have no problem. There is treatment for it. You will be treated and because of that you urge for free treatment even if the person hasn’t got a work do we call it? Err…Health Insurance.

Reference 3 - 0.63% Coverage

**I:** OK

**P:** Because the person has come and report and it is in relation to AEFI, ensure that the person is treated for malaria.

<Internals\\IDIs DHMT\\NRDI_08> - § 1 reference coded [11.93% Coverage]

Reference 1 - 11.93% Coverage

**I:** Ok. What might be the reasons why caregivers do not report AEFI to health care workers?

**P:** Eh! Some of them do not know it is a reaction `or they do not know that is ..., some report wrongly. Like he is already having his, the child is ready having some sickness then after well say it is the immunization that has made this main while the did is already having this. Some too did not see it is be necessary, they want treatment when something happens they want to go to the hospital for treatment. They do not know that it is from the immunization. Do you understand?

**I:**  (laughs) please explain more.

**P:**  I said, - you said why is that some people do not report

**I:**  Yes

**P:** And I said, they do not know, some do not know that they should report.

**I:** Ok.

**P:** Eh and some too they just, when it happens, they are just like that but they would associate it with the injection.

**I:** Ok. Do you have more reasons to add to that?

**P:**  Another reason is what? Let me see..., for the forms, the forms are there, so I do not know. On the part of the women?

**I:** Yes.

**P:**  Hmm, that is just the reason. They do not know they are to report and others do not know that it is a reaction to the immunization.

<Internals\\IDIs DHMT\\NRDI_09> - § 1 reference coded [11.90% Coverage]

Reference 1 - 11.90% Coverage

**I:** What might be the reason why caregivers do not report AEFIs to health care workers?

**P:** Caregivers do not sometimes report AEFIs to health care workers because they might take it as a normal condition and probably on what is happening to their children or to them has nothing to do with immunization they have just taken. So in the nutshell, I will say they don’t know, they don’t have much knowledge on AEFI and then our nurses too are not educating them very well. Looking at the kind of information they may give them when they give their wards the immunization or even the mother themselves the immunization. because I once chance on a health worker who after giving an immunization to a mother, a child and then I asked her, did she explain anything about AEFI and she said yes and I asked her that what did she tell her and she said she told her that when she goes and the child develops fever she should sponge the child and if it persists she should come back to the facility and I said ok, and I said weather, that was all? And she said yes and I said ok. So, there i realized that it was inadequate because if is not fever but any other thing what happens. So I think the information given is inadequate, yea.

<Internals\\IDIs DHMT\\NRDI_10> - § 1 reference coded [14.93% Coverage]

Reference 1 - 14.93% Coverage

**I:** OK. To you, what might be the reasons why caregivers do not report AEFI to health care workers?

**P:** As I said earlier on, they might not take it as consequences of the immunization; so I have fever, vomiting, I will go to the hospital than reporting to the one who immunized me. Probably, it will be later that we will get to know. So for that matter, a lot of the things, the Adverse Events we don’t see them but they will go direct to clinical side, which might not be. They might not also know and the patient or client will not tell then I had immunization that might be why this thing has happened, that is why if not, of course it is a serious reaction, they cannot sit in the house, like you child is convulsion, you take it to the hospital. He will not come to the Directorate or Community Health Nurse or Disease Control Officer, they will go straight to the hospital and the history too must be later on probably that we will get to know, so what we can do is we only have to counsel them, tell them if you go home and anything at all and if you go to any place, also mention that you had immunization and other end too can call so that we come and fill the form and it is managed accordingly.

<Internals\\IDIs DHMT\\NRDI_11> - § 3 references coded [12.40% Coverage]

Reference 1 - 4.11% Coverage

I: What might be the reasons why caregivers do not report AEFIs to health care workers?

P: Well, I may be speculating but what I think is that, some of them don’t see them as things that are life threatening besides I think that “err” may be our “err” education to care givers may not be that comprehensive what we will see as AEFI, a care giver might see it as a normal occurrence, for instance a rash on the skin, care giver is likely to ignored it depending on the level of “err” the education we give at the facility level.

Reference 2 - 6.38% Coverage

P: The reasons why caregivers?

I: Do you have more to add?

P: “Yaah”, sometimes, it might be ignorance, ignorance could also be another issue. For instance if the caregiver miss the education, the time of the education at the clinic session then there is the possibility that she might not be able to know that this is an adverse effect and for that matter i should report. We equally have “errmm”, let me say a lot of quacks within the system sometimes a caregiver will recognize that this is “errr” an AEFI but may be a quack will appear and say oh, this is nothing and then some other communities too, the mere fact that they are inaccessible, it takes them a long time to travel and may be to get to the nearest health facility to report some might be reluctant to report some of these occurrence. (Phone ringing)

Reference 3 - 1.91% Coverage

I: Ok.

P: Care givers may report or they themselves might see it but they will take it as a common occurrence or somebody can see heat rashes but the person will not be able to probe into details to know that exactly this is what is the cause.

<Internals\\IDIs DHMT\\NRDI_13> - § 1 reference coded [4.76% Coverage]

Reference 1 - 4.76% Coverage

I: What might be the reasons why caregivers do not report AEFI to health care workers?

P: I for the caregivers as times we say when it doesn’t immediately happen at the health facility and they go home as times because of the distances where they are coming from they always feel reluctant to go back to the health facility unless it is very serious but if it is that the child is inching or may be vomiting they always feel it will go

<Internals\\IDIs DHMT\\NRDI_14> - § 1 reference coded [4.65% Coverage]

Reference 1 - 4.65% Coverage

I: What might be the reasons why caregivers do not report AEFI to health care workers?

P: I think they don’t report sometimes we don’t pre inform them and also sometimes when aa you give something like and they come we don’t have the patience to listen to them we feel that that might not be the cause of the immunization or antigen we have given so because of that some of them entertains fears.

<Internals\\IDIs DHMT\\NRDI_15> - § 1 reference coded [6.55% Coverage]

Reference 1 - 6.55% Coverage

I: What might be the reasons why caregivers do not report AEFI to healthcare workers?

P: mhm sometimes I think it because people trying to just neglect certain things because sometimes they feel is not most the AE AEFI that are reporting are not live threaten so they feel it’s not that important to report it mean while its basic necessary to report every AEFI

<Internals\\IDIs DHMT\\UEDI_01> - § 2 references coded [14.36% Coverage]

Reference 1 - 6.08% Coverage

I: ok, so would you happen to know why I mean, give me reasons why caregivers may not report an adverse event following immunization to healthcare workers?

P: well, sometimes, I don’t know

I: (laughs)

P: but most of the caregivers feel like you know those who believe in the, the traditional system, they love to attribute a lot of conditions that come out to spiritual and all those things but aside that, there are some of our health workers who tell them ooh eerrm, maybe they bring the child and they tell you, this injection I am giving the child, the thigh will be a little hard or it will be warm and with(inaudible) so when the mother starts to see those signs, some of them take it to be normal (ok)so they don’t even report (ok), they keep it till it is worse

Reference 2 - 8.28% Coverage

I: ok…. (Time) so any other reasons they may not report?

P: no, as for that one I really don’t think there is any other reason they may not report

I: ok

P: if it is in the part of the health workers, as for that one I could say and I, haha, and I know very well, we ever had, it wasn’t a community health nurse, (ok), it was a general nurse who did an injection, I think it was a wrong site

I: alright

P: so the person had the abscess and came back and the way the matter was handled, it wasn’t nice (ok) so it, it brought some kind of fear to the others. It is like when you inject somebody wrongly (ok) and an adverse reaction comes or the person gets an abscess or something and comes to report, you will be singled out, as if you don’t know your work much (ok) so even though some of the health workers might report, the, the caregivers might report but some of the health workers will keep it at their level(ok), they wouldn’t want it to come out because they might feel like they will say they don’t know how to inject or something like that.

<Internals\\IDIs DHMT\\UEDI_02> - § 1 reference coded [6.63% Coverage]

Reference 1 - 6.63% Coverage

I: So I wanted to know reasons why the caregivers of the infants that they vaccinate may not report an adverse event to health workers.

R: O the caregivers

I: Yes.

R: Ok. Yeah that’s a very good question, but it has to do with ignorance (Ok). You see, in the health setting eh… our clients normally do not know what to expect from even the health workers (yeah). So it’s an issue of ignorance – they don’t know that some of these things they’re important information to us. So we also need to do some public education (Ok) on these things so that people will be alert and report these things (Ok). Yeah.

I: So earlier on you were even talking about the fact that health workers need focal persons and because of the workload and maybe like many reporting forms they may not get there the time. Are there any other reasons why health workers too may not report adverse events following immunization? (7 seconds)

R: I think that is what (laughing) I think that is what I can say about that.

<Internals\\IDIs DHMT\\UEDI_03> - § 2 references coded [12.41% Coverage]

Reference 1 - 7.18% Coverage

I: So what might be the reasons why caregivers (Umm) Umm do not report adverse events following immunization to healthcare workers?

P: Umm. Caregivers?

I: Umm.

R: To me, specific (Umm), I will say that it is…(TIME) maybe the way we give the education to the caregiver (Ok), in the sense that you know drugs or vaccination, definitely you vaccinate the child temperature will increase. But we say that because it’s the vaccination, so give paracetamol, it will go. You give it go, will they report? (No). if it’s drugs you know every drug has its side effects small small (Uhmm), so maybe but as we give the solution to the client, when it happens like that the client will not take it to be serious again to report (yeah), it will end there. To me that may be the reason (Ok). There are some drugs, you take it you have headache, and then with the history or the inaudible(14:54) it’s indicated, but it will go (yeah). So as you’re giving to the client too, maybe you may tell the client O, if you take it this is what will happen. But that is it. But we look at what we’re going to cure. Is that not it? (yeah) and the person too is having the pain; the person will take it like when it feels like that, but I’m already informed that it will go (Umm) so within a short time when it go, that’s it; you continue taking the drug it will go. But he know that that’s the actual thing (Ok). So how will the, the person will never report (yeah). He will never report (Umm Ok). To me that may be the the. If you just say that anything happening, one thing that I’ll tell you that: all the people that have come to the facility, they all come to report (Umm). If you just go anything different with what you’re seeing, I’m telling you all those who have come to the hospital today, they will all report that they have problems (Ok) (7 seconds). Time

Reference 2 - 5.23% Coverage

I: So are there any other reasons why, aside the fact that because maybe they tell them that this may happen and so they feel reluctant to come? Are there any other reasons that the caregivers (yes) would would not report.

P: The the care the is the care from the caregiver eh?

I: Yes, yes.

P: That is what I’m just telling. The caregiver I’m not sure there’s that is just the major thing (Ok). Unless it’s severe, he cannot bear (Ummm). Beyond what he can bear, then he has to come (Ok). But just like, maybe like the way I was saying the fever, maybe headache, all those things that we are supposed to report, that’s what I’m saying. Like the person is having maybe a serious convulsion (Uhmm), meaning they still have to rash and come. So that’s what I’m saying. It’s beyond their level, but like ehm these petty petty things, I’m not sure nobody will come and report (yeah). Maybe the person may even travel far away to come. The person is even thinking think the distance will tell you that I cannot come (yeah). Maybe the person come here morning, morning afternoon time he left the facility. You get what I’m saying? (Umm). You get to the house, evening time maybe something is happening it’s not so serious, the person (may not come) may not come (yeah). And maybe the following day it will not occur again (yeah). So that ends it.

<Internals\\IDIs DHMT\\UEDI_04> - § 1 reference coded [7.97% Coverage]

Reference 1 - 7.97% Coverage

I: okay, alright. Thank you very much Sir. Sir what would you say are of the reasons why caregivers are not reporting adverse events to health workers

P: probably before immunizations we normally will explain possible events they will encounter so probably when they encounter events they see it as normal because somebody is been immunized I mean vaccinated and we telling the person that there is a possibility of getting a fever, so what do I do when I get the fever, when there is a fever we take paracetamol it would reduce the temperature so once the person gets fever, paracetamol is administered and the temperature goes down, then reluctant to report that. We would need all that information building up a database, but once children are immunized and those minor ones are taken care of at home, they are hard to be reported. So those we would report will be severe or moderately severe ones most probably that the caregivers will come complaining for the forms to be filled.

<Internals\\IDIs DHMT\\UEDI_05> - § 2 references coded [6.91% Coverage]

Reference 1 - 5.21% Coverage

I: okay, what might be the reason why caregivers do not report adverse events to health workers?

P: sometimes you know we have new staff coming in to the system. Sometimes maybe proper orientation is not been given to them and they, in Garu for instance most of them work alone. a staff might just come from maybe he’s just newly posted to the field, he goes there and starts work on her own, there is nobody to supervise the person unless the DHMT move there or the sub-district leader or the team there happen to go there and see what is happening. So I think that they don’t actually have adequate knowledge about it that is because they have not being proper training on the reporting format on these cases.

Reference 2 - 1.70% Coverage

I: will there be any other reason why you think caregivers are not reporting?

P: no I think basically it’s the information that they are not having, maybe they don’t have much information about it, not enough training given to them.

<Internals\\IDIs DHMT\\UEDI_06> - § 1 reference coded [6.93% Coverage]

Reference 1 - 6.93% Coverage

I: okay sir Thank you so what might be the reasons why caregivers do not reporting AEFI’s to healthcare workers?

P: yes, one of them is, when they come, sometimes the care, I have experienced it in one facility where the health staff will tell you, this is not anything, this is not anything, they will try to rubbish what the caregiver is saying, go go go, it will go. Not that they don’t report, they report but the health workers will rather tell them that oooh this one it will go by itself that is one of the main reasons, I have witness that before, I was there and said if it s eve just fever we need to report, and so caregivers, caregivers another thing too is some of them prefer self medication, and this “I don’t have money” “I don’t have health insurance too” and when I come you will demand to, once am coming with this case I have to pay something, so those things are all part of the reasons why caregivers will prefer not to come or report. So if am to list them into three;

1. I will say, health staff attitude, trying to rubbish what the care giver says, then
2. Caregiver coming with the condition then the health staff will say that, okay if you don’t have health insurance, pay, then
3. We can say that, eerrh, some of them they prefer self medication, ahaaa at home. Somebody can even say that ooh am a health staff or a family member, a member of the family is a health staff so let me take the child to the person and do so.

<Internals\\IDIs DHMT\\UEDI_07> - § 1 reference coded [11.62% Coverage]

Reference 1 - 11.62% Coverage

Thank you Sir. Sir what might be some of the reasons why caregivers do not report adverse events to health workers should they encounter any?

P: ...that hasn’t come to my knowledge yet, because I know the small deviation from normal after the vaccination the caregivers get scared and they report except that they may not know that its related to the vaccination and report as a normal sickness to the health facility, right but where they know that it relates to the vaccination, then they report as such. That depends on where they are reporting to. If they are reporting to a health professional who is not involved like the clinical staff who is not involved in and they do not mention that mmhm it was after the immediately after they vaccinated that it happened then it will go unnoticed except that its directly related like a boil at the site of or a swelling at the site of the vaccination then the clinical staff will pick it up as such but if it’s just vomiting or fever, they may think it’s an ordinary thing

<Internals\\IDIs DHMT\\UEDI_08> - § 2 references coded [8.60% Coverage]

Reference 1 - 6.31% Coverage

I: okay, thank you very much. what might be the reasons why caregivers are not reporting adverse events to health workers?

P: mmhm, some of them especially those who don’t have health insurance, because they cant afford they don’t want to come to the facility where they will say pay this for this drug before your child will be attended to, but with those with the health insurance some of them they think the local drug store at the eerrh community llevel can treat such conditions for them so some of them rely on those things and alos those quark doctors at the community base sometime they prefer to go to them to see how best they can assist they but at the end, they will fall back to us when it is worse, so those are some of the issues

Reference 2 - 2.29% Coverage

I: will there be others?

P: others, some feel, you brought up, like its from us we caused those condition to their children so there is no need to rely on us again so they prefer to go to someone else or somebody at the community level, so those are some of the issues.

<Internals\\IDIs DHMT\\UEDI_09> - § 1 reference coded [5.53% Coverage]

Reference 1 - 5.53% Coverage

M: What reasons might prevent a care giver from reporting an AEFI to the health worker?

R: To me sometimes they don’t know that, it is something that they have to report and the next thing might be going through a long distance to report may be if the distance is far from where they came for the immunization and they had to come back through that long distance and say this is what happens. May be we don’t give them the education that when A and B happened they should let us know immediately.

<Internals\\IDIs DHMT\\UEDI_10> - § 1 reference coded [4.32% Coverage]

Reference 1 - 4.32% Coverage

M: What reasons might be or might lead to a care giver not to report AEFI’s to a health care worker?

R: I think care giver right! Usually those people are very ignorant like they don’t know what to do. Dough they are always told but unless of course the person is bed ridden. That is where they see it to be important. They feel reluctant to report and secondly they are always afraid to go and report. They think they have given to all the children why is it that your child is reacting? , they feel reluctant to report.

<Internals\\IDIs DHMT\\UEDI_11> - § 1 reference coded [5.66% Coverage]

Reference 1 - 5.66% Coverage

M: You know in the health care delivery system we have the care givers and the health care workers? What reasons might prevent a care giver from reporting an adverse event following immunization to the health care worker?

R: Sometimes the regard them as being minor, sometimes I say it depends the type of information we give to them, we tell them if it is just headache give them paracetamol it will go, what if the headache doesn’t stop? So the care giver will also say the nurse said if you get common headache or pain, give paracetamol it will go. So they would also sit at home for some time before they would come. So to me it depends on the way we give information to the care givers in the community.

<Internals\\IDIs DHMT\\UEDI_12> - § 2 references coded [9.74% Coverage]

Reference 1 - 5.76% Coverage

I: alright thank you very much, so what might be the reasons why caregivers do not report AEFI’s to healthcare workers?

P: I think one of the reasons is the lack of knowledge on the aspect of it,(voices in a distance) some of them when you vaccinate or you administer a drug it doesn’t take immediately, a client goes home, a client goes home and it happens within the day or after some few days after the drug or the vaccines it happens, the caregiver, if the caregiver does not receive information from the client, he cannot give you but most of the caregivers come back and if they come back, they always look at the other methods of solving that adverse event(phone ringing in the background) and lacking the knowledge of reporting it because the knowledge has to be that it may be the cause of the drug and that thing has to be corrected, so that kind of knowledge is not there that is why I talked about the training.

Reference 2 - 3.99% Coverage

I: okay, so when you say knowledge, on who’s on whose part, lack of knowledge on whose pat?

P: on the caregivers

I: okay, will that be your only reason?

P: that may not also be the only reason, the other reason is eerrh who to, they don’t see it as a responsibility of one, let’s say we are about three working giving immunization at the facility, this thing happens, who reports? its suppose to be, so all activities that is compiled should be added, so a responsible person is suppose to be with every facility, that we have people who are responsible for activities to monitor such things so that it would speed up the reporting rate.

<Internals\\IDIs DHMT\\VRDI_01> - § 1 reference coded [14.28% Coverage]

Reference 1 - 14.28% Coverage

I: That is great. Moving on what might be the reason why caregivers do not report AEFI to healthcare workers?

P: ooh I think they report, I cannot be specific, am not at the grassroots so I can’t say much, but I think during health education they need to be informed through that in case your child is giving this and you see that, fine, as I said anxiety some might not se, se, one might not see that before so there is that sort of eeeh allaying of anxiety, oh my child wasn’t this way when I brought the child you know it happens sometimes ahaa, but so I thing, I don’t know whether they are, fine the reporting or none reporting may be due to the information not getting, or caregivers not aware of what they should expert but I think routinely they should inform them to expert

I: okay

P: yeah so in case it happens, it shouldn’t be news and they should tell them the steps to take in case it occurs that’s reporting back to them or whatever depending on where they are

I: okay that’s that is great.

P: mmm because they move to outreach points too so all the communities might be around them but fine if somebody has eeeh communicating gadget, the phone I think, give your, ooh this is my number any, whatever happens you can call on me for us to discuss take it from there.

I: That is helpful. And eeem moving on again, please what might be a reason why healthcare worker, themselves right, who encounter AEFI are not reporting.

P: Hmm (3seconds) as for that I think the eer core person in the, as I said some of the reasons I gave if I were to be on the ground this should have been what I would have done, this is my number call me, initial discussion you give health talk this is what you may expert or you may see when it happens, but you know that person at the grassroots I cannot tell on their behave, maybe unless you interact with them down there because they are been informed when it happen or do this and that educate on it, what might be happening what may happen because it might not happen among all children, what they may see or any … reaction that will come after the immunization let me simply put it, so they are, I think they are aware but it’s now left to put it into action.

<Internals\\IDIs DHMT\\VRDI_02> - § 1 reference coded [7.37% Coverage]

Reference 1 - 7.37% Coverage

I: Okay, alright that’s fine. Errrm Would you help us with some of the reasons why care givers do not report adverse events to health workers? The parents of the…

P: The parents don’t report? … I think, I’m rather of the other view because when they see any adverse errr this thing they will report to us.

I: Okay

P: They report. They’ll come to you that oh when you left this that that happened to the baby. They report…

<Internals\\IDIs DHMT\\VRDI_04> - § 1 reference coded [17.30% Coverage]

Reference 1 - 17.30% Coverage

I: Ok Errhm can you please give me some of the reasons why caregivers do not report adverse events to health workers?

P: Care giver as in the the the community level?

I: Yes, the parents of of the infants who come for the vaccination…

P: Yes…

I: Is it…

P… they they they will always report.

I: Okay!

P: I mean from the experience I’ve I’ve had when when the child … reacts beyond the acceptable this thing they will surely report, they will get back to the CHN trying to inform her that oh! This is what happened. What of help can you offer to to to to solve or resolve the condition? But where I had the challenge was like I stated earlier some of them were actually not reporting, the staffs.

I: Okay!

P: Because they feel like it’s an indictment on their part. I mean, I am a nurse when I… It will mean that no! I don’t know the work I’m doing. You understand…

I: Okay!

P: Yes, so I have to come and tell them that no, that it’s not the issue. I think this issue came up during the training. I told them that with adverse events following immunization, there are, I mean varied factors that could result in that, one could be that erhmm technological issues, from the manufacturer. The vaccine could have a problem that when the the the child is vaccinated, the child will react. We also have the individual, I mean errh system you know we are made of diff.. someone will react to certain things, we react to things differently as individuals. That’s the recipient aspect. And then the the the capapcity issue of the staffs. Because it looks like if the the the vaccine is supposed to be given subcutaneously and ended up giving it at the wrong, I mean route of administration, you end up, I mean sometimes too I think the the the one classical one that we investigated that we deal with the the the dosage aspect.

I: Okay...

P: …The staff gave the wrong dosage and the child reacted I mean seriously to it so we have to come in, speak to the mother…they they will always report but where the gap occurs is the this thing level. So in our community interaction too, any engagement we have with the community members, I mention it to them when the… their child they receive the vaccine and the child is reacting to it be it crying, swelling they should quickly inform the nurse and then errh… Fine they will do that like I said maybe the nurse coming to inform us… but anytime they see me too or any of the officers, they should also approach us so we will see how we will we will deal with it.

<Internals\\IDIs DHMT\\VRDI_05> - § 1 reference coded [7.07% Coverage]

Reference 1 - 7.07% Coverage

I: Thank you and eeem moving on, what might be the reason why caregivers do not report AEFI to health workers.

P: Well I, I will think that after the, before they give the injection they will tell you that you will have fever, after this the child will have fever so if it happen give paracetamol, if after three days the fever is not down then bring the child to the hospital. So they wait for the three days and if still the child is eem having before they will come but is a long time I have seen any EFI may be because am, and ….that might be the reason because they are pre-inform that this will happen if it happens, if is fever after three days is still there come but if swelling or the place is redden come back. But for the mothers not coming then it has not happen that is why they don’t come to report.

<Internals\\IDIs DHMT\\VRDI_06> - § 2 references coded [28.05% Coverage]

Reference 1 - 14.45% Coverage

I: okay! errhmm what might be the reasons why you think care givers are not reporting adverse events to health care workers?

P: errhmm… basically, errhh… I think sometimes, the way it is discussed errhh for instance, if there’s an abscess and sometimes the discussion says maybe the technique for giving the… erhh… whatever…

I: …vaccine…

P: …the vaccine…may be at fault so some of them assume that they… if they report then it shows that I wasn’t good enough.

I: okay…

P: …with my technique

I: Okay…

P: other times, they think it’s a common fever so… (mtchew), it’s not an issue make errh… so much noise about about it so…sometimes…and they think… and that one too… and other times they do other things they errh they…by the time that…urhh so may forget because especially if it’s not as significant errh… an issue, like just a fever or errhh small abscess or something, you think you can manage at lower level. *Vehicle passing*

Reference 2 - 13.60% Coverage

I: …and do you think some of the parents also do not report?

P: yeah! Some of the parents also do not report ‘because sometimes they attribute it to another issue, other times too, it was well explained so they think it’s okay, they can manage, yeah! Ehehh!...

I: alright urmm do you have any suggestions for improving the process of adverse events?

P: oh basically, I…I think erhh erhh there should be an active errhh search enough after immunization programs errhh, community health nurses should be actively instructed so that it’s not just the exception reporting…

I: Okay!

P: …but the general asking…

I: Okay!

P: …what has happened and those active errh information should come up, as a reporting system. It is already, but it is like if there’s an adverse…

I: mmhmm!... yhhh!

P:… and eheh… yeah! But if it is a general routine, then we may pick a lot more than...

<Internals\\IDIs DHMT\\VRDI_07> - § 1 reference coded [3.86% Coverage]

Reference 1 - 3.86% Coverage

I: Ok, ok. Alright, now to the level of caregiver, the mothers, could they also be a contributing factor to the low reporting of AEFI?

P: There are times that errh, they might not be aware, what is happening to… that is as a result of maybe the drug the children took or the vaccination. So lack of knowledge will also be contributing factor from the side of the caregiver, who might not know. Maybe the care, errh, the health personnel did not explain well to that caregiver, what are the possible side effects of this particular vaccination they are going to take.

<Internals\\IDIs DHMT\\VRDI_08> - § 2 references coded [15.17% Coverage]

Reference 1 - 6.82% Coverage

I: Ehmm, what do you think could possibly be the reason why ammmh, we are experiencing this low reporting or zero reporting even from the level of caregivers? What could possibly be…

P: Well, my stake on this is that errh, it looks like errh we are not seeing very severe adverse events. They are minor ones, so our staff think that the minor ones are not necessary. They give their paracetamol or whatever that will…. It normally happen, they assured the people so they don’t think that it’ necessary to… even the slightest complain to fill a form and then alert us. They fill that is just a minor adverse event, so there was no need to report on them. I think that’s the main reason why we have the low reporting.

Reference 2 - 8.35% Coverage

I: yea, that can be look at from the point of care….

P: Yes, yes at the lower level.

I: At the lower level. That is within the facility.

P: Yes,

I: but I’m also looking at…. From the mothers.

P: From the mothers?

I: Yes, if the mothers don’t report the case. Nhmm ok. Or report cases to the facility, they may also not have any to report.

P: Yes, I think that will be due to the fact that, they have been educated and counselled on the possible adverse events that they may see.

I: ok

P: Be it minor, to the major.

I: ok.

P: So the minor ones, they are aware and they know how to manage them. So some of them don’t border to come back to us and report on them.

I: Ok.

P: I’m sure that might be the reason why there is so… The low reportage. Unless is a very severe one that, they realized that it’s beyond them, then they quickly rush back to the clinic.

<Internals\\IDIs DHMT\\VRDI_09> - § 1 reference coded [4.42% Coverage]

Reference 1 - 4.42% Coverage

I: ok, ok, alright. Can we look at some of the factors that account for this low reporting of AEFI, first from the caregivers’ level? What do you think could possibly be the reason why ehrr, we have low reporting with respect to mothers?

P: ok, with respect to mothers, some, sometimes herr, if you take fever for instance, they are made to understand or by their own experience, they know that, there are some of the vaccines, a child takes and then becomes warm or has some fever. And so with that it is accepted to be normal

I: ok

P: ok, although is also an AEFI. It’s accepted to be normal and so that does not call for concern, but if it has to do with ehrr something more severe like an abscess, ahaan, that definitely they may report.

<Internals\\IDIs DHMT\\VRDI_10> - § 2 references coded [8.60% Coverage]

Reference 1 - 8.15% Coverage

I: Alright, emmh, do you know what could possibly be some of the reasons why emmh, there is low reporting with respect to caregivers of adverse events?

P: Ok to, to, to the best of my knowledge errh, the way we perceive the AEFI in our locality, we believe that as the training given to our people so far, is not happening.

I: ok

P: Yea, because upon all these that we’ve said and even at times we also go in for our supervision and we’ve never come across any of these. So we believe that is not happening. It means the… our active health staff, they are also vigilant in all the areas and we the supervisors too or monitors too, when they go in, we are not also seeing… Is also giving us another signal that actually errh, is not happening.

I: Ok, so it isn’t that mothers are not forthcoming with reports on cases

P: yah

I: but because they are not experiencing those cases or something?

P: Yea, errh, what I believe most is that, if any mother should see any change with the child, surely the mother must report.

I: ok

P: Because there is no way a mother would see a child sick and the mother will even keep mute about this without reporting.

I: ok

P: And moreover too, our nurses, they do home visit.

I: ok

P: So does that mean when they go to the house, they see the mother together with the child. Would they see the mother alone and go without seeing the child? So it means during

Reference 2 - 0.45% Coverage

their home visits, they are not even seeing such a case in our communities.

<Internals\\IDIs DHMT\\VRDI_11> - § 2 references coded [9.76% Coverage]

Reference 1 - 5.82% Coverage

I: ok ok, ammh, what do you think could be the reason for the low reporting of cases? We’ll first look at it from the point of caregivers. Ok? What could be accounting for the low reporting of adverse events following immunization?

P: Errh, what I think will account for that is erhm, errh, lack of knowledge. There hadn’t been enough or adequate post immunization education. And then also, as I stated earlier we think errh or they think pain after immunization is normal because if you have a prick you’ll feel pain, so that should be the common one they should have being reporting but once they consider it normal, they don’t report. But we hardly come across abscess, we hardly come across swelling at the site of immunization. So these maybe the common ones that they should be reporting, but as I said…

Reference 2 - 3.94% Coverage

I: they don’t report.

P: Mmmh

I: Don’t you think there could be some…., apart from them seeing it to be a normal thing, couldn’t there be a…, possibly be a reason why mothers don’t… probably from the way they are treated by staff or something like that?

P: Well for that one, errh I will not say errh because of the way they are treated. yea, to the best of my knowledge here in this municipality, we don’t errh usually encounter that kind of behaviour, attitude towards the clients to the extent that if there is any problem, they cannot report.

<Internals\\IDIs DHMT\\VRDI_12> - § 1 reference coded [12.49% Coverage]

Reference 1 - 12.49% Coverage

I: ok. Emmh, can it also be, be also be attributed, attributed to the mothers, possibly for not reporting cases to the facilities?

P: Ooh ok, yes it can, errh, most especially when they have been informed or they have been told that, they shouldn’t apply anything to the injection site and they do that, definitely they will not report on it if something happen. They will not, I mean report on it. And also the mothers not knowing what to report and what not to report.

I: Ok, ammh, have you had any such experience before?

P: Yes

I: And when was the last one?

P: Ooh, it was 2000 and…

I: I mean in your

P: in here

I: capacity

P: as a public

I: as a public health nurse.

P: Yes, when I was in Akatsi, I was also the public health nurse, so I experienced that when the, this thing was given, TT; Tetanus Toxoid, those days. That’s 2001,The arm was swollen and apparently, it seems we went to Ho and the community health nurses told... they were using cold compress. And with the cold compress, they left it there. Ahaan, they didn't, I mean spread it, it was at the site and it got to a time that, the woman too went and apply Kerosene and all kind of things and at the long last, it has to be referred to the regional hospital. But then… and they wanted sue us but I don’t know but because of the kerosene *(laughs)* that they’ve used that have actually errh, errh. And I think the arm got amputated.

<Internals\\IDIs DHMT\\VRDI_13> - § 1 reference coded [9.56% Coverage]

Reference 1 - 9.56% Coverage

I: Sir what might be the reason why caregivers do not report AEFI to healthcare workers?

P: Mhm for that one it could ignorance or they are they don’t have the education to understand that there is the need for them to report to whoever has given them the vine immunization so and at times the timing they don’t have time for them to even to come for next session of EPI activities they don’t come at times you have to force them so that when they realize effects of immunization, they are at times they are not boarded to report so for me I may say maybe the time that they will use to come to you and maybe whiles they come to you are also doing something you are not giving them attention it could contribute to their ignorant like them be ignorant of reporting to you. And at times the seriousness also maybe the minor one they think it is normal or eere they have receive counselling right after the immunization so they give time for it to get down before they whatever happens before they notify.

<Internals\\IDIs DHMT\\VRDI_14> - § 2 references coded [8.49% Coverage]

Reference 1 - 3.33% Coverage

I: Ok ok ok madam what might be the reason why caregivers do not report AEFIs to the health care workers?

P: In my view is like we don’t sale the idea to caregivers we don’t tell them that this is what they will see and when they see it they should come to us to report what we do tell them is the child will be just worm they should give peemore aahna so that is it.

Reference 2 - 5.16% Coverage

I: Is there anything you want, you want to add, (laughing) is there anything you like to tell me about why they don’t report?

P: And some too maybe people like market like this very market day we do go to the market for immunization and people don’t live around here people who access the market services don’t live around here they live on the island so if you do that and the child is having that challenge they can’t come back to you until another time so maybe in two days three das, three days time the child will be fine so market day she will not bring the child.

<Internals\\IDIs DHMT\\VRDI_15> - § 1 reference coded [9.63% Coverage]

Reference 1 - 9.63% Coverage

P: Mhm we lease with region

I: Ok what might be the reason why caregivers do not report AEFIs to healthcare workers?

P: Yeah I,I don’t I can’t actually tell but what l know is we always give education even me when l get to any facility , service delivery point l give them education on various things so AEFI we always want the mothers to know what type of immunization we give them and what they should expect aha so if it is beyond the normal we always tell them to report but usually you ask and they said nobody has reported ahaa so maybe it is a challenge now we haven’t delve in to it to check why are they not reporting so maybe is something we need to do.

<Internals\\IDIs FDA\\GAFDA_01> - § 1 reference coded [2.52% Coverage]

Reference 1 - 2.52% Coverage

I: Okay,’erhm’ please what might be the reasons why caregivers do not report AEFI to healthcare workers?

P: Because they think the events are non-reportable or they are already known.

<Internals\\IDIs FDA\\GAFDA_02> - § 1 reference coded [8.71% Coverage]

Reference 1 - 8.71% Coverage

I: Okay, please ‘erh’ what might be the reason why caregivers do not report AEFIs to healthcare workers?

P: ‘Hmmm’ caregivers ‘erhm’… they may, I cannot think of any reason but if even if they cannot report maybe one reason why they may not report is that, if they stay far away from the health care worker they may not want to report because I mean, why would you spend time, pay transportation to go the health worker, to go to the health facility to report an AEFI. So they may choose to manage it at home rather than spending so much money and time to go to the hospital. Secondly, you know in our…system it takes some time to see a health worker. Assuming you go to a district hospital it may take you [laughs] sometimes two hours to see a health worker, so they may stay at home that ‘oh’ I don’t want to go and waste all this time to see a health worker so they may go, stay at home and manage it. So these are two reasons that I think they may not want to report the AEFI.

<Internals\\IDIs FDA\\NRFDA_01> - § 2 references coded [6.42% Coverage]

Reference 1 - 3.39% Coverage

I: what might be the reasons why caregivers do not report AEFIs to healthcare workers?

P: this, the reasons may be multi-faceted

I: ok

P: but most of the time, we ask the field workers that the tell us, oh the thought having may be high temperatures is normal or vomiting is like the reactions are not so severe so for that matter the don’t see the need to report but we always tell them that weather is severe or not they have to report because we want to know you know the reactions associated with the vaccine that has been given out so that we can may be prevent any future occurrence.

Reference 2 - 3.02% Coverage

I: is that all concerning that? The reasons why caregivers are not reporting to healthcare workers or?

P: I think

I: is there any other reason?

P: here er, we are not giving more publicity or education on that and for that matter people think is normal if there is more education on it that even when you take the vaccine any untoward reaction that you feel, you have to report, I think it will also boost you know reporting.

I: ok

P: so there should be more education day in day out , every now and then.

I: ok

P: yes

<Internals\\IDIs FDA\\UEFDA_01> - § 1 reference coded [4.19% Coverage]

Reference 1 - 4.19% Coverage

M: What reason might account for a care given not to report an adverse event following Immunization to a health worker?

R: may be lack of understanding that this is an adverse event because lacks of education down to the care giver that they have to report every adverse event to the health care professional. And I would also say motivation because something that has been put in by one of their core mandate or core duties. They always don’t take it as important so they are not serious in reporting so I will say first of all lack of education and motivation that could result to that.

<Internals\\IDIs FDA\\VAFDA_01> - § 2 references coded [12.48% Coverage]

Reference 1 - 8.97% Coverage

I: Ok, alright thank you very much. Now to the core issue, the low reporting of, of AEFI; adverse event following immunization. What do you think could possibly the reasons why we have the low reporting? What could account for that?

P: I believe we looked at AEFI as a campaign sort of thing.

I: ok

P: We have not descended further to the routine level.

I: ok

P: Emmh, anti-tetanus is it a vaccine?

I: Yes

P: yes but how do we consider it? As a normal injection or a vaccine? As a routine. We’ve not gone to the level of availing to the routine guide. They are different from the immunization thing.

I; yes

P: As to whether we have availed forms to that level that specifically this is the reason why I don’t believe we have gotten to that. I’ve, I’ve follow-up on this thing sometime and I think procedurally or structurally, we should look at this before we even come to the motivation.

I: ok

P: yea

I: Any other reason you think…

P: The other, other reason is that, still people haven’t gotten to level terms that your reporting does not actually lead to incrimination, where you are liable to punishment or something and that when you do it, is supposed to help the system. It supposed to help re-designing training material, it supposed to help re-design education material. People haven’t gotten to those terms yet because it’s believe that when you do it either you will be castigated, either you will be dented, either you will be seen as a conduit for creating problems for the district or some sort of hierarchy.

I: yah

P: In my region we, we had it that all reports are to go to the office of the regional director of health

I: ok

P: the regional of Ghana Health Service, before they come to FDA for onward transmission to erh, erh Accra. Now it cause the delay, it cause even mix, mix erh, erh, mix-up. It cause the mix-up. Emmh, I can show you a form that came with a different medication as the sample attached to the form and when you looked at the form, now the form is gone, they are to bring the samples and they brought samples that do not have any inclination to the form that was submitted. But when we were going there to collect, when there is any problem the ICP is able to relay it direct. Now I don’t know whether it is felt that the ICP might be reporting on somebody’s non-availability

I: yah

P: to be at work or whatever or something, so they are not… And you know there are hierarchies, and errh, Ghana health service is not FDA,

I: yes

P: so something to come out of there to FDA, the headship must know.

I: yes

P: Structurally, we’ve not looked at this, so even though…. I’m talking about this because I made a… there was a form of scholarship forte ICPs too. So we were supposed to choose and then inform our head office and then they give the scholarship to who we feel. So we looked at the ICP that is mostly reporting. Frequency,

I: ok

P: we realized one gentleman from…. For the sake of whatever let me keep the details. So chose this person to enjoy the scholarship but Ghana Health Service had a different idea, a different opinion. Yea, you see, how can we make a choice within their structure?

Reference 2 - 3.51% Coverage

I: Alright thank you very much. Emmh, we’ve looked at it from the point of the health care worker, when we look down the lane to the caregiver referring to the mother or the mothers, could they possibly be a reason why… could they be a factor to the low reporting that we are experiencing? What do you think?

P: Personal opinion here, a caregiver as you use is… when you look the economy, you may think we are all the same but when you actually go further down, you realize that the use of the mobile phone, when you happen to be in some communities, the credit units that sell there is the one cedis and the two cedis. That tells you of the level of the economy in that place so the person picking the phone to report… initially, there was no toll free as we have now,

I: ok

P: yes there is no toll-free as we have now, so for then to pick their phone and then call, there is no refund. They don’t see the need. Already they are doing self-medication there already and then you’ll quiz to find out what else they are adding to the medication that has been given for them to be *(coughs)* observed that they are adding a herbal concoction to what you are giving them already. So that also adds up that, they not giving the whatever. Yah.

<Internals\\IDIs PROVIDERS\\GAPI_01> - § 1 reference coded [8.01% Coverage]

Reference 1 - 8.01% Coverage

I: What might be some of the reasons caregivers do not report AEFI’s?

R: The reasonis probably because we tell them to manage it and most of the reaction that they get is fever and rashes and when we tell them to manage it we are not encouraging them to report and when they come for the next immunization we are not asking did you get case so that if she didn’t willinging come to report you could ask and fill a form. So because of that we are not encouraging them to report. After applying all the measures the child got better so no need to report.

<Internals\\IDIs PROVIDERS\\GAPI_02> - § 1 reference coded [6.60% Coverage]

Reference 1 - 6.60% Coverage

I: What might be the reason why some caregivers may not report to the health worker?

R: Sometimes out of ignorance, or sometimes they take it as it is normal and sometimes too the nurses might not do their work well instead of educating them to say if you see a swelling, rashes or fever that is more than three days come back and report but they don’t give such education and tell them when to come or we can give them our contacts such that if we are not available they can call and talk to us.

<Internals\\IDIs PROVIDERS\\GAPI_03> - § 1 reference coded [8.16% Coverage]

Reference 1 - 8.16% Coverage

I: So what might be the reason why some care givers do not report AEFI’s to health workers?

R: We haven’t had any instance but if a care giver report on a different aliment or not giving the full history about what happens like not telling the doctor my child took yellow fever and is running temperature and comes to say my child has fever where the doctor may not question if the child took any vaccine that resulted in that and that I think is why a prescriber may miss an AEFI.

<Internals\\IDIs PROVIDERS\\GAPI_04> - § 1 reference coded [5.72% Coverage]

Reference 1 - 5.72% Coverage

I: What might be some of the reasons why caregivers are not reporting to the health workers?

R: Education is more important, they don’t know it and they don’t educate them to say when you give this vaccine this what will happen, they say just give para for some number of days but they don’t tell that if u see sometime like that come and report early so we investigate to know whether it is from the vaccine or not but if they say just give para it won’t help the mothers.

<Internals\\IDIs PROVIDERS\\GAPI_05> - § 1 reference coded [11.59% Coverage]

Reference 1 - 11.59% Coverage

I: What are some of the reasons the caregivers are not reporting to the health workers?

R: The reason maybe one; they give paracetamol syrup at every session when they come and fever is the commonest AEFI so they tell them to give the paracetamol or they already have the paracetamol with them before coming, they give it to them all the time so fever is not reported now on but injection site abscess that one too they have knowledge of using ice block to massage the place and because they come every month when anything happens before that they don’t want to come back, they only come to tell after the incidence has happened and if they manage and it is not going then when they come they come to report that I have been trying to manage this and it is not going, apart from that the severe ones like rashes or a very severe pain at the injection site, so those are some of the reasons why I think the mothers don’t report, the mindset of having paracetamol on them to give when the child has temperature or the place when they stay is far from the clinic and most of them don’t have time.

<Internals\\IDIs PROVIDERS\\GAPI_07> - § 1 reference coded [7.02% Coverage]

Reference 1 - 7.02% Coverage

I: if that’s the case, what might be the reason why caregivers do not report AEFI to healthcare workers?

P: are you sure?

I: yeah! Some do not report AEFI to healthcare workers…we want to know the reason

P: that one I can’t say because usually our place here, they report to us, so I don’t know the reason why they don’t report.

I: but in general, do you think that why, so reasons why some may not report

P: …then that means they didn’t document it so they forgot or they think they can handle it themselves.

<Internals\\IDIs PROVIDERS\\GAPI_08> - § 2 references coded [14.58% Coverage]

Reference 1 - 7.77% Coverage

I: Okay so having said that then, what might be the reason why caregivers do not report AEFI to healthcare workers?

P: I think most of the time it has to do with fear of being reprimanded like somebody who has given immunization and later on ‘erhm’, them, they reported with swollen ‘erh’ leg at the site or there is a sore or something.’ Erhm’ how come I feel that if she report it they might reprimand or they might take some ‘eih’ ‘erh’ action that might go against her, them so they have this fear that reporting it will, will cause ‘erhm’ bring them problems, they will bring them so they normally try to cover it up.

Reference 2 - 6.81% Coverage

I: So this time I want to know what about the mothers or the caregivers what will be the reason why they do not report Adverse Events to the healthcare workers?

P: Mothers, most of them they have I,I , we say it’s lack of education most of them are not able to identify the Adverse Reactions. The common ones that is the injections that one is clear the mother can be able to, to relate it to the injection but the other ones like diarrhea and she might not be able to say that it’s a so she will not report it. So I think it’s lack of education.

<Internals\\IDIs PROVIDERS\\GAPI_09> - § 1 reference coded [8.02% Coverage]

Reference 1 - 8.02% Coverage

I: so if that’s the case, then what might be the reason why the care givers ‘erhm’ do not come to report AEFIs to the health care workers

P: I can’t really tell. Sometimes I will say; illiteracy is part, others too don’t really have time to listen to what you are saying. Sometimes you can give a health talk, at the end of the day you ask and nobody will be able to give you the exact what you’ve said. Or even just ‘erh, ‘erh’, ‘erh’ half of what you said, they can’t even, they can’t even tell you. So sometimes you know, you can’t really tell or may be what we explained to them they couldn’t get it well ‘eheh’ or even the process that we even gave, maybe they did not even understand. So it’s both side

<Internals\\IDIs PROVIDERS\\GAPI_10> - § 1 reference coded [5.14% Coverage]

Reference 1 - 5.14% Coverage

I: okay, having said that, we will also like to know if erh… why ‘erhm’… what will be the reason why caregivers who encounter AEFIs ‘erhm’ do not report to the health care worker?

P: oh! Maybe they do not have knowledge about AEFI. They don’t know that it is an adverse effect, ‘erh’, ‘erh’ event following immunisation that is the reason why. You know sometimes some people are late comers, by the time that they will come we have finished giving health education. They are [inaudible] so for them even they wouldn’t know what is AEFI. So with them automatically, they wouldn’t report but rather bring the child to the clinic ‘uhuh’

<Internals\\IDIs PROVIDERS\\GAPI_11> - § 2 references coded [12.23% Coverage]

Reference 1 - 7.53% Coverage

I: so if that’s the case, then what will be reason why caregivers do not report the AEFI cases to health care workers?

P: why they do not?

I: yeah

P: sometimes you may explain to them alright but some may think otherwise. Some too probably the maybe distance for them to come back or maybe the financial cost and they want to come

I: is that all?

P: because they don’t have any option than to….you shouldn’t go and report and you won’t be charged for that so if they should have come. But some just don’t want to come they will try their own self-medication. Like the fevers, ooh let me give some paracetamol

Reference 2 - 4.70% Coverage

I: So please, what might be the reason why health care workers themselves who encounter the AEFIs are also not reporting them? Could it be administrative staff issues or registers?

P: oh it maybe staff issues because we have the forms, we have everything. We have… if you want to call with a telephone, we have a telephone. Why should it be….? So maybe it’s attitude. Human attitude

<Internals\\IDIs PROVIDERS\\NRPI_01> - § 1 reference coded [5.21% Coverage]

Reference 1 - 5.21% Coverage

I: So what might be the reasons why care givers do not report AEFI to health care worker?

P: Certain times they don’t see the need if its only fever they think oh my body, my child temperature or body is warm so may be the person has para syrup or any other thing she gives and when the temperature is down she is confortable so because of that the don’t see the need unless otherwise it is severe

<Internals\\IDIs PROVIDERS\\NRPI_03> - § 1 reference coded [5.90% Coverage]

Reference 1 - 5.90% Coverage

I: okay, what might be the reasons why caregivers do not report adverse events following immunization to health care workers?

P: I think they don't take it serious like we ourselves don't take it serious, so and then most of them either they have forgotten that they have been told or errh errrh something like that, not for remembering to say it.

I: Any other reasons?

P: Emmmm, and then may be it could also be that when they tell us we don't give feedback. Because if you don't get feedback yourself you don't give it to them. Nhmmm I think.

I: Any other?

P: I can't remember any other.

<Internals\\IDIs PROVIDERS\\NRPI_04> - § 2 references coded [7.94% Coverage]

Reference 1 - 4.79% Coverage

I : So……………what might be the reasons why caregivers do not report Adverse Events Following Immunization to your health care workers?

P: Mmmm ..... most at times am thinking many be they don’t know. Errh sometimes they don’t know Errh even some times some people come here they they just consider it as normal illness someone will not tell you is after they injected their child that this thing happened ehhrr. She will just take it as normal and even they too they they will just say ohh noo err err is a normal sickness so they bring it sometimes they won’t the tell you is even an AEFI.

Reference 2 - 3.15% Coverage

I : Any other reason?

P : Errh and mostly sometimes they are afraid of the, those who went and did it. They don;’t want to like ehherr Errh jopadise their repu err reputation ( inaudible) or injection [laughing] and it will be swelling . if he comes and say it, it will be like the fellow did not do the injection well ehher ( inaudible)

I : Any further reason?

P : Errr I don’t think.

<Internals\\IDIs PROVIDERS\\NRPI_05> - § 1 reference coded [9.42% Coverage]

Reference 1 - 9.42% Coverage

I: Why are they not reporting to the health care workers?

P: Well! Probably because if it is a minor …… like incident or event they, they will not report like if a child is having fever, they will just bath the child and give para and try to observe and see. If they observe and it goes away the fever goes away they will not come. Errrhrrr they will not waste their time to come, if they it is abscess that one they may come, if it is abscess they may come for us to attend to them but the minor minor ones they will not come even abscess like this you know it can go after some days errherrr so they will just observe, usually they want to observe, you know people don’t like going to hospital naturally, errrherr so they will like to observe at home for a number of days. If it is not going away then they will report but if it goes away they will not come again.

I: What other reasons …. may stop them from reporting?

P: Ahhh .... may be ...... I think the other way could be that like when they come to the facility errrrherrr when they come to the facility probably the health we the health workers don’t treat them well. We say oh, somebody can say oh this small thing and you are bringing it … to the hospital? So is also how we communicate to them and also the education too like the need, the needed to also be educated or awareness creation among the caregivers that one after giving you this when you get back home and this and this happen bring the child’s back to the facility immediately. I think that one too is not done properly. Hmmmm.

<Internals\\IDIs PROVIDERS\\NRPI_06> - § 1 reference coded [7.07% Coverage]

Reference 1 - 7.07% Coverage

**I:** What might be the reasons why caregivers do not report AEFIs to healthcare workers?

**P:** Sometimes [Laughs] it is just our attitude.

**I:**  Attitude of??

**P:** One basic thing this our attitude

**I:**  Of the Health workers?

**P:**  Attitude of health workers, yes, our attitude, a woman will come may be , you know some of them when they come they will tell you that they injected my child and my child is doing thing, even though you will know that what the woman is reporting is not based on the immunization but the way approach the woman sometimes they will come . I have seen it here. So that is basically the main thing and sometimes, it is the distance, as I am talking some of our communities are almost in East Mambrusi. So if I go there to do my immunization and there is anything they go to the nearest facility there and I wouldn’t know. So those are the things and some of them too the distance is far they will just sit down and find their ways to manage their thing. And will not even come because how to get to the facility isn’t easy.

<Internals\\IDIs PROVIDERS\\NRPI_07> - § 1 reference coded [4.98% Coverage]

Reference 1 - 4.98% Coverage

**I:** Thank you. What might be the reasons why care givers they don’t report AEFI to health care workers.

**P:** Err... I would not say they dont report, err prior to immunization, we give education and the education we give is that these are the possible symptoms of side effects of the drug that might come and we assure that, we give them the assurance that it just take some few hours to resolve. So some parents when they begin to get some minor signs and symptoms, they feel that we are told already, so they feel relaxed, they don’t come, yea. But we normally also insist when you see that it getting severe or serious the definitely they will realize the side effect or the adverse effect is being severe. They normally come as many as will have that symptoms or situation.

<Internals\\IDIs PROVIDERS\\NRPI_08> - § 1 reference coded [7.57% Coverage]

Reference 1 - 7.57% Coverage

**I:** What might be the reasons why caregivers do not report AEFIs to healthcare workers?

**P:** The reasons...hmm, I think, this one at times it the…, I can say it; it is the attitude of the staff. Some feel that if they came back and report, they scold them or something or that sort. Some, they, they say because of these things they don’t come to report. Some also maybe, they stay far away, so they don’t see the need or have the time to come back to give the complaint. They go, consult their colleagues instead of them coming back. They feel that the distant is also far, so they feel like consulting a colleague than coming back to the facility. So I think they are some of the reasons. Some too would feel they won’t attend to them; nothing will be done to them so why should I come. So they stay at home.

<Internals\\IDIs PROVIDERS\\NRPI_10> - § 1 reference coded [4.88% Coverage]

Reference 1 - 4.88% Coverage

I: What might be the process why care givers do not report AEFI to health care workers?

P: it may be dueit may be that some caregivers may see it to be normal or some at them may be afraid to come to the facility

<Internals\\IDIs PROVIDERS\\UEPI_01> - § 2 references coded [8.42% Coverage]

Reference 1 - 2.18% Coverage

I: so what might be the reasons why care givers do not errm report an adverse event following immunization to health workers, health care workers?

P: care givers as am saying if the child is having any unusual this thing immunization, when the care giver feels that is from drug usually they will come with the child uhuh that this after taking this in the night we couldn’t sleep, the child was (inaudible) temperature and all those things, the child was vomiting, they even say it’s because of the drug they will come with the child to the facility … they will come

Reference 2 - 6.25% Coverage

I: so with that how can we like improve an adverse event following immunization reporting by care givers

P: yeah, mostly when we are giving the immunizations we tell them … for this that we are giving we will expect that the child will have temperature or the site of the vaccination there will be pain or there may be swelling so if there is pain or swelling they should apply (inaudible) press uhuh they should give paracetamol if there is fever uhuh so we will tell them that we will tell, we will pre inform them the child as we are giving this the child is likely to experience this so if they experience this they should come back to the facility

I: ok …. Are there any other ways we can improve reporting from these care givers on adverse events …. Aside just telling them, you know you mentioned you always tell them about some of the side effects they may get and what to do when these side effects come so do you think errm by doing that it helps them to be able to report more … on these cases?

P: yeah errhh it do help, but most often we even have community volunteers within the communities who are there with errh people we tell them that the community volunteers their also in the community they move around uhuh they come to the outreach service point with us to help us uhuh so if they have difficulty getting us they should get to the community volunteer they will call us oh this child have received this and this what is happening then we will instruct the volunteer to tell the care giver to bring the child uhuh or if sometimes it become necessary we can dispatch a community health nurse to follow up mhmm

<Internals\\IDIs PROVIDERS\\UEPI_02> - § 1 reference coded [6.72% Coverage]

Reference 1 - 6.72% Coverage

I: Ok. So Uhmm…what might be the reasons why caregivers do not report adverse events to health workers?

P: The reason? That’s why one of the reason may be maybe that they’re ignorant – they don’t know

I: Ok

P: You understand? Some of them, because they’ve just given them the immunization, they go they don’t know that that’s (yeah). So when it happen they just leave it and wait till the day they’ll come back for the next immunization. You understand. But here…to me here, we have been telling them, so anybody who goes home…then there’s any problem with the child, they just come back. Even if they can’t come, those who are far, they g o to the volunteers, and we also give them our numbers (Ok). If you can’t come, you can’t go to a volunteer, you just call us. We will come there and attend to you.

I: Ok. So aside the fact that some are being ignorant…they don’t know…so they don’t report, are there any other reasons, they may not like to report? Or

P: Well, there’s no other reason. Mostly the other person why I said ignorant was that…that woman even came…like the woman that I’m even talking of; the one we had, she came from Kumasi (Ok). You understand. Ahaa. That’s why I say sometimes some are ignorant because our colleagues might give the immunization but they probably they didn’t tell us some of these things that she didn’t know. But here we tell them, so any little thing, even if you go and there is something wrong with your child, bring the child back (Ok) umm, they come.

<Internals\\IDIs PROVIDERS\\UEPI_03> - § 2 references coded [5.80% Coverage]

Reference 1 - 3.96% Coverage

I: ok … so what might be the reasons why care givers do not report adverse events following immunization to health workers?

P: another one is that some of them they have our phone numbers so sometimes some can call so that when if the we can do the follow up some of them they I don’t know some you would you would tell them this and then still some of them will be sitting and say when you go when you go and meet them you will say but we say you should come they will say. It’s like you didn’t tell them or they forgot or some of them will say some of them they in the house the older people there will also give them a different thing all together so they would be sitting in the house also trying to do other management in their level which may not help matters but for now I think most of the mothers are enlightened just when they experience that the first thing is to run to the facility

Reference 2 - 1.85% Coverage

I: ok … any other? … any other ways to

P: I think these are the or even the opinion leaders in the communities

I: ok … so meaning they can get in touch with the opinion leaders too

P: yes especially we if we the …. We even though most of the volunteers already know some of the this adverse events we are talking of so like if we bring them if we bring them more closer on board concerning this I think it will help

<Internals\\IDIs PROVIDERS\\UEPI_04> - § 1 reference coded [6.80% Coverage]

Reference 1 - 6.80% Coverage

I: thank you for your response, madam, what might be the reasons why caregivers do not report AEFI’s to healthcare workers?

R: mmmm, some of them maybe they don’t even know so ignorance can be one of them. Some too like if it is the fever, they will feel that maybe by 2, 3 it will go so they sit down with it, except that it is something serous as I said with the abscess so they had to sit for 2 week before they brought the child back. So sometimes caregivers don’t {silent} whiles they think that the immunization is to protect the children, they always feel that, the fever, the rash they can manage them

<Internals\\IDIs PROVIDERS\\UEPI_05> - § 1 reference coded [13.79% Coverage]

Reference 1 - 13.79% Coverage

I: okay, so what might be the reason why care caregivers do not report AEFI’s to healthcare workers?

P: maybe they have not been educated, they are ignorant, so if the information has not been given to them, they don’t see that it’s an adverse effect, it is it is for we the health staff while we are administering the vaccines we should tell them that should you have observe anything untold or unusual in the symptoms of the child come and report to us, sometimes we have not, lack of education we have not been able to carry on the information.

I: will that be all or you will have any other....?

P: no, other things maybe that, some people you know the lackadaisical attitude of people, sometimes they say ooooh lets wait till tomorrow, some people are doing that. The other thing is that mention must be may today that the the the health challenges (voices of people in the next office) now have being, we are over over people either that the health staff are now managing the health challenges for people, you go to, in my district here, we have about two spiritered churches. You will be surprise that the patients are admitted there before they come to me when the condition is getting worse then they push the patient. They are now believing that patients, people can pray and the patients can be healed, so those are some of the things, it’s now the order of the day and I don’t know what the health authorities are doing, I don’t in your area where you coming from the big research centre, I don’t know what is happening? Am sure you are experiencing something like that. I don’t know is but if I were a health administrator or manager, I will not agree that I go to a spiritered church, see patients admitted, I will discharge them, I will order and discharge them. Churches are you go to the churches to pray you don’t sleep in a church, if you are sleeping in a church it means that they are turning the church into health facilities and it’s not the purpose of a church, its criminal, is it not true? Should churches admit people, no?! We go to the church to pray and go home, so if you get to a church and people are admitted do you think it’s a church?! So the health administrators should do something about that. You go to a spiritual church and find about 100 people are laying there, they say they are there for prayers, which prayers? And then the spiritual father or pastors turns his house and makes facilities to admit the people, is it a church? Huh, we are not in the revolution, they said freedom of worship not in the pastors house freedom of worship in the church or in the pastors house huh, if the freedom of worship in a pastors house is not a worship, he’s turning himself I mean he is deceiving public and we should do something about it, I don’t know your know view about that...

I: ....well....

P: the health authorities should do something about that

<Internals\\IDIs PROVIDERS\\UEPI_06> - § 1 reference coded [3.32% Coverage]

Reference 1 - 3.32% Coverage

I: okay, would you have an idea what might be some of the reason why caregivers do not report AEFI’s to healthcare workers?

P: I think it should be communication gap, communication gap such that if I forget to tell the caregiver that look as I’m giving you this child, you know I have being telling people when you give someone the drugs tell the person the side effects the person will see, when he sees it, it is nothing new, he will still continue to take the drug and if it is an adverse reaction, you know side effect is different from adverse reaction, this is the side effect you will see, these are the adverse reactions that you may also see but the adverse reactions you have to report. No caregiver who he will be told these things and he will not report but in in in an event where the service provide forgets to tell the caregiver that look, what you need to know about it and this is what you do should you see ABC.
