# Supplementary material for: Optimising reporting of adverse events following immunisation by healthcare workers in Ghana: A qualitative study in four regions
Source: PLoS One. 2022 Dec 20;17(12):e0277197. doi: 10.1371/journal.pone.0277197 (PMC9767370; doi:10.1371/journal.pone.0277197)
Supplement: S1 Data — (ZIP) [file pone.0277197.s001.zip › Minimal data/S7 Receipt of Feedback on AEFI reports.docx]

**Name:** 14. Receipt of Feedback on AEFI reports

**Description:** This node contains all information on feedback from adverse events that were reported to the next level.

<Internals\\IDI RHMT\\GARI_01> - § 1 reference coded [1.75% Coverage]

Reference 1 - 1.75% Coverage

I: so do you receive feedback on AEFI cases from the next level?

P: ‘oohh’, if there is anything then they will report to us. If, normally it is like, if they don’t seems to ‘erh’ find anything, then [giggled] is silent. But if there should be anything,…

<Internals\\IDI RHMT\\GARI_02> - § 1 reference coded [5.70% Coverage]

Reference 1 - 5.70% Coverage

I: the question I’m going to ask I’m sure you would have an idea. How do you receive feedback on AEFI cases from the next level?

P: ‘uhuh’! you know initially I said that feedback is a problem. ‘Uhuh’! Because sometimes they send the forms there and it takes a while, it takes a while to get the feedback. But ‘uhh’, I’m sure they’ve been sending feedback to them. They send it to, you see where the offices are apart? ‘uhuh’ so, normally it will go to that side, that’s where the deputy director, public health is and that is where the surveillance officer also is. So it goes to the surveillance officers or the deputy director, public health and they store it there. They keep it in their records.

<Internals\\IDI RHMT\\NRRI_01> - § 1 reference coded [3.98% Coverage]

Reference 1 - 3.98% Coverage

I: Ok, ok do you receive feedback on AEFI case from the next level?

P: “Yaah”, we do. That is how come we even know that other regions are doing better than we are doing and that is why at and this will normally happen at other regions at the national level when regions are presenting.

I: Ok.

P: Then you will have the idea of what others are doing and of cause, National level is always the next level that we report to and they are those who will normally share the information with us.

P1: Dr. Acheahu will share the information. Dr.Acheuhu, when he gets the feedbacks he gives us the feedbacks.

I: Ok.

P1: AEFIs.

I: Ok.

P: And even feedback, feedbacks is also is some of the bulletins they send to us on weekly and monthly bases.

<Internals\\IDI RHMT\\NRRI_02> - § 1 reference coded [5.63% Coverage]

Reference 1 - 5.63% Coverage

I: You already spoke about the feedbacks so do you receive feedbacks on AEFIs?

P: Me I don’t receive feedbacks (laughing) the feedback may be that, may be during “errr” EPI programs and the just encourage us “errrr” to report. Let’s say when you are embarking on a mass vaccination of “errrr” a targeted drug, targeted vaccine and group, the will encourage us to report and things like that and the feedback that comes is that we don’t report but sometimes with mass vaccinations “errrr” we, the report two times and this is collated and sometimes you see the next mass vaccination we are given us the results and then the reports or the previous once and the may indicate that this number of people reacted and then the “errrr” conditions were that, that, that, fever, this this this this then encourage you know so but “errrr” I think that for me if I reported an adverse effect at the end of the day I should know exactly “errr” what is really the issue, it is really an adverse effect or is another condition or that “en heerr”.

<Internals\\IDI RHMT\\UERI_01> - § 1 reference coded [3.01% Coverage]

Reference 1 - 3.01% Coverage

M: do you receive feedbacks from adverse events following immunization cases?

R: may be the EPI coordinator later may be able to answer that because I don’t directly submit forms and if there is any feedback it doesn’t come to me. It goes to the EPI coordinator so he would be in the best position to tell.

<Internals\\IDI RHMT\\UERI_02> - § 3 references coded [9.77% Coverage]

Reference 1 - 5.71% Coverage

M: Would you therefore suggest that those things contributes for care givers or parents not to report AEFI’s to health authorities?

R: Mostly likely and as I tied it with the feedback because our reporting goes through a lot of channels. For instance, if it is picked up at the community level, you have to pass through the CHPs compound, to the sub district to the district, to the region, to FDA and to national. Even with feedback it has to come same way. So most times feedback is lacking. So if I report to you and I don’t get to hear what happen to it. I am not motivated to report because at the end of the day, I don’t get convinced that the information I have given is something important. So if no feedback it means nothing concrete has come out of it, so it is like you are reporting for reporting sake. So if I have not seen that this report has led to this with regards to these vaccines, then what will motivate me to report? So these are some of the issues I have realized could be attributing to low reporting of AEFI.

Reference 2 - 2.25% Coverage

M: Do you receive feedback on AEFI’s from the next level?

R: As I said, that is the major challenge as a surveillance officer I am not sure I received any concrete feedback regarding cases reported. All that we hear is that the reporting is a bit on low side. That is why I earlier on said the reluctance to report may be as a result of feedback, I think they need to do a lot with regards to the feedback.

Reference 3 - 1.81% Coverage

M: so what could account for the failure of the feedback to you people?

R: That is difficult to tell because the challenges are at the next level. That will be a guess and I am not too sure but I am aware they are all aware of the importance of feedback I don’t think I will be in the position to know what might cause that.

<Internals\\IDI RHMT\\VRRI_01> - § 1 reference coded [2.93% Coverage]

Reference 1 - 2.93% Coverage

I: Please do you have any other issues you think is important to…

P: The AEFI?

I: Yah with respect to the AEFI?

P: Yah, like you mentioned that do we give feedback to the district or the facilities, and I mentioned that we have not be getting feedback from national. If there would be a system when they go, national look at them, which ones are serious, which are not serious, and they give feedback. If it’s serious they should give feedback, even if it’s not serious they should give feedback.

<Internals\\IDI RHMT\\VRRI_02> - § 1 reference coded [2.70% Coverage]

Reference 1 - 2.70% Coverage

I: ok, ok. So emmh, when you receive such information and you forward it national, do you usually get feedback on that?

P: emmh, that one, the district disease control officers are in touch not really my office.

I: ok

P: maybe they could give the right answer to, whether they have constant feedback from national.

<Internals\\IDIs DHMT\\GADI_01> - § 1 reference coded [7.04% Coverage]

Reference 1 - 7.04% Coverage

I: Do you receive feedback on the reports you send to the next level?

R: Hmmm, that is the problem at the district level we don’t receive any feedback because when I hear from the facilities that brings the report FDA gives them the feedback directly but it doesn’t pass through the district, I think it is good because at least those who did something will be happy to know what they reported has been resolved.

<Internals\\IDIs DHMT\\GADI_02> - § 1 reference coded [3.93% Coverage]

Reference 1 - 3.93% Coverage

I: But do you receive feedback on the cases you report?

R: For case investigations, yes of suspected cases to know whether it is negative or positive we get a feedback. AEFI has not been well documented.

<Internals\\IDIs DHMT\\GADI_03> - § 2 references coded [9.40% Coverage]

Reference 1 - 4.98% Coverage

I: Do you get feedback when you send the forms to the next level?

R: Yes, it’s difficult to remember now, most at times we have received some feedback but I don’t know if it is in terms of drug reaction or adverse event following immunization, I think they acknowledge receipt of our forms at my former place the forms are there so I could just pick it up and tell you.

Reference 2 - 4.42% Coverage

I: Do you get feedback on the cases you report?

R: The feedback is usually verbal; because we work in chip zones when the mother comes for the next weighing then she tells the nurses what, sometimes they go for home visit and find out how the child is improving and what has been done. It’s just verbal feedback no written one.

<Internals\\IDIs DHMT\\GADI_04> - § 2 references coded [6.55% Coverage]

Reference 1 - 1.36% Coverage

I: Do you get feedback on the cases of AEFI you report to the next level?

R: No

Reference 2 - 5.18% Coverage

I: Why?

R: I think the reporting is even a problem so like you asked me if there were any reports, yes there may be but because I don’t have it on my desk it is not reported and the second thing too is the process of reporting is so long and that makes the feedback too delay and I think it is not good.

<Internals\\IDIs DHMT\\GADI_06> - § 2 references coded [5.07% Coverage]

Reference 1 - 2.99% Coverage

I: Do you receive feedback on the cases you report to the next level?

R: Yes we get feedback but not in a written form, when we go for trainings they just tell us, some are reporting 0, some 1 and those that are not reporting at all they should try and report so the feedback is mostly oral, when we go for training and those things they stress on the need for us to be reporting of AEFI.

Reference 2 - 2.08% Coverage

I: But nothing has been done to improve upon the monitoring of the reports?

R: No, I wouldn’t say there is a concrete plan for us to fall on but we just go round for visits and we just reinforce the reports but there has been nothing concrete program that is earmarked.

<Internals\\IDIs DHMT\\GADI_07> - § 1 reference coded [4.18% Coverage]

Reference 1 - 4.18% Coverage

I: so please having said that, do you get feedbacks on the cases from the next level on.

P: yes, yes.

I: on AEFI cases

P: we get feedbacks when we send. If we don’t get we ask, if we don’t get we ask for that feedback from the regional level because we channelled it through the regional level yes. But sometimes, the feedbacks don’t come ‘erh’ early hmm, they don’t come.

<Internals\\IDIs DHMT\\GADI_08> - § 1 reference coded [2.80% Coverage]

Reference 1 - 2.80% Coverage

I: Okay so will would also like to know if you receive feedback on AEFI cases from the next level.

P: I don’t receive it, that one ‘dier’ I don’t receive anything. I normally send it I don’t receive anything, information that this condition or maybe we have received this, this, this but…

<Internals\\IDIs DHMT\\GADI_09> - § 1 reference coded [1.44% Coverage]

Reference 1 - 1.44% Coverage

I: so please, do you receive feedback on AEFI cases from the next level?

P: [4 seconds]…not really. Unless there is a workshop and they’ve seen something [inaudible] and then they will give ‘erh’…

<Internals\\IDIs DHMT\\GADI_10> - § 1 reference coded [1.70% Coverage]

Reference 1 - 1.70% Coverage

I: Please do you receive feedback on AEFI cases from the next level

P: That I cannot give a definite answer. Yes! Because it hasn’t gone through, yes! But I am sure that if it goes through it will even, if it delays it will still come. Yes

<Internals\\IDIs DHMT\\GADI_11> - § 1 reference coded [5.02% Coverage]

Reference 1 - 5.02% Coverage

I: okay, so we want to know if; doyou receive feedback on AEFI cases from the next level?

P: [12 seconds] I think so.

I: we would like to know why?…if you receive feedback from the next level, we would like to know why

P: yes ‘erhm’ like if maybe it’s from the vaccine, they will tell them so that maybe they can withdraw those vaccines from the system.

<Internals\\IDIs DHMT\\GADI_12> - § 1 reference coded [2.77% Coverage]

Reference 1 - 2.77% Coverage

I: so please do you [inaudible] usually receive feedback on AEFI cases from the next level?

P: next level as to region?

I: yes, maybe regional or national

P: ‘erh’ that one too unless maybe you ask the [inaudible] because for me I’m not aware of that

<Internals\\IDIs DHMT\\GADI_13> - § 2 references coded [7.64% Coverage]

Reference 1 - 3.61% Coverage

I: How did you receive those reports?

P: It came from the sub-district to the district, routinely. It’s a system that is established, so routinely they report and that’s how it came. So there was a hard copy form and before then one of the incidence came directly to us because a mother came directly to the office to come and report which we found a bit strange but we had a form filled afterwards and that’s the process that it went through

Reference 2 - 4.02% Coverage

I: We would also like to know if you received any feedback on AEFI cases from the next level

P: From the regional level. They put together…they aggregate the data. They put the data together and at ‘erhm’… some meetings and some conferences they would beam it back to us and tell us, let’s say at the end of the year these are the number of AEFI cases that we saw, these are the districts that are not reporting that are silent. Stuff like that. So that’s the kind of feedback that we receive

<Internals\\IDIs DHMT\\GADI_14> - § 1 reference coded [5.61% Coverage]

Reference 1 - 5.61% Coverage

I: ‘Erhm’ we would like also to know if you receive feedback on AEFI cases from the next level.

P: We, above us like Accra? [Participant fanning] feedback in what form?

I: After ‘erh’ you send you’re, let’s say maybe a report on Adverse Events Following Immunization from the top?

P: If it will come [inaudible] will be directed through the director and then she will discuss it with us.

I: So that means you people get feedback?

P: If there is any feedback it will get through her to us ‘uhuh’.

<Internals\\IDIs DHMT\\GADI_15> - § 1 reference coded [2.71% Coverage]

Reference 1 - 2.71% Coverage

I: but do you give “erhm”, do you receive feedbacks erhm on AEFI cases from the next level?

P: yes. When we send it to the next level, we get the erhm feedback. Yeah! But it takes a longer time because of our system, it takes a longer time.

<Internals\\IDIs DHMT\\NRDI_01> - § 1 reference coded [6.99% Coverage]

Reference 1 - 6.99% Coverage

I: So the the forms that you complete and then send them to the next level do you normally receive feedback on those forms.

P: Actually those I can say whether we do receive feedback some I remember some time ago we did and sent but the feedback mostly is the disease control officer he communicate with them hmmm he will communicate with them about whatever that is going and may be the facility that has reported that particular this thing so the communication is then between the linked to either the health worker that goes to that particular this thing so that you can get back to the people and tell them this is what is happening this is where we have gotten to and those so because mostly the way the nature of our works are all though we are part of it [baby crying] but at times [somebody badges in].

<Internals\\IDIs DHMT\\NRDI_02> - § 1 reference coded [1.31% Coverage]

Reference 1 - 1.31% Coverage

I: Ok, the forms that you compute and then forward to the next level do you receive feedback on them?

P: I have never received a feedback before.

<Internals\\IDIs DHMT\\NRDI_03> - § 1 reference coded [1.59% Coverage]

Reference 1 - 1.59% Coverage

I: Do you receive feedback on the AEFI eeh AEFI forms that you sent?

P: No, the ones that I sent to the region I have not received any feedback.

<Internals\\IDIs DHMT\\NRDI_04> - § 2 references coded [2.79% Coverage]

Reference 1 - 1.68% Coverage

I: Do you receive feedback on adverse events following immunization cases from the next level?

P: Arrrh for now I have not received any …. From any level.

I: Do you receive I mean you unit?

P: This unit?

I: yeah the district?

P: The within the district?

I: *Yeah*

P: For now *noo*…no

I: Why?

P: Probably there is no any

Reference 2 - 1.11% Coverage

I: I am not talking of the care giver but your other staff who are under you who report their cases to you

P: Yeah when they report to me, because we have not receive any report. So there is no feedback in that regard. Yeah

<Internals\\IDIs DHMT\\NRDI_05> - § 1 reference coded [5.07% Coverage]

Reference 1 - 5.07% Coverage

I : Do you receive feedback on adverse events following immunization cases from the next level?

P : (smile) in fact ehh I must say (laugh) that has been one of the challenges.

I : So why not?

P : Well we cannot tell ......... Even not that alone there are other disease conditions that ehhh we have to report immediately even as at when we do ehmm considering their incubation period .... we were suppose to get a feedback ..... even a case ..... or some of the disease conditions is an outbreak and for that matter we need to mobilize logistics to you noo try to see how we can mitigate the situation or stop ehhh the condition from traveling to other places but sometime you get feedback when ...... time is is is is is passed ... So feedback is really a challenge I cant remember receiving any feedback since I came to this District but because the feedback when it comes also opens your eyes and then helps you enow to put one or two things in place, but feedback is a serious challenge.

<Internals\\IDIs DHMT\\NRDI_07> - § 1 reference coded [3.21% Coverage]

Reference 1 - 3.21% Coverage

**I:** Do you receive feedback on AFEI cases from the next level?

**P:** No, no.

**I:** Why?

**P:** That is why I am saying, because it is the mild ones, for this it is just the non-serious ones we don’t hear anything when you hear reports it is finished. Even the this thing, when we did the exercise, I can just show you if you look at the electronic this thing laying here, I mean no one will tell anything because once it doesn’t create anything and for that matter may not help the producer of the vaccine or for that matter the manufacturer of the drug or the programme this thing, the programme managers then we don’t hear anything again.

<Internals\\IDIs DHMT\\NRDI_08> - § 1 reference coded [1.83% Coverage]

Reference 1 - 1.83% Coverage

**I:** Do you receive feedback on AEFI cases from the next level

**P:** From the region

**I**: Those that the district report to

**P:** I am sure; I am sure when they report they receive feedback

<Internals\\IDIs DHMT\\NRDI_09> - § 2 references coded [9.40% Coverage]

Reference 1 - 4.67% Coverage

**I:** Do you receive feedback on AEFI case from the next level?

**P:** Feedback on AEFI, I will say Yes, because the Regional Health Directorate always give us weekly bulletin and any district has reported but the outcome of the AEFI is not actually detailed in the, the weekly bulletin. And well, let me understand well because the outcome of AEFI will definitely be stated in the case-base form, so I will say, yes, yes, we receive feedback, we receive feedback, we receive feedback

Reference 2 - 4.74% Coverage

**I**: But if it is a serious case, like the weakness of the limbs, don’t they investigate?

**P:** Yes, we investigate at the district level, we are in charge of the investigation and so we should rather give feedback to the lower level but for some time being we have not had such a case

**I:** But assuming the district level also refers a case to the higher level.

**P:** As you said assuming, since it is an assumption we will assume that they will give us feedback. (Both laugh over response)

<Internals\\IDIs DHMT\\NRDI_11> - § 1 reference coded [4.01% Coverage]

Reference 1 - 4.01% Coverage

I: Ok, Do you receive feedback on AEFIs cases from the next level?

P: So far, I haven’t seen a feedback yet.

I: So why do you think that you are not receiving feedback?

P: (“Laughing”) Well in fact that is for is the higher level. “Yaah” “emmm” we reported 2 cases of AEFIs to the regional level but the feedback is usually in our bulletin and usually when it is our cases, they recovered even at a stage and was stated on the form so possible that is the reason why we couldn’t get the feedback immediately.

<Internals\\IDIs DHMT\\NRDI_12> - § 1 reference coded [0.66% Coverage]

Reference 1 - 0.66% Coverage

I: So do you receive feedback on AEFI cases from the next level?

P: From the next level?

I: Yes.

P: “Hmm hmm”.

<Internals\\IDIs DHMT\\NRDI_13> - § 1 reference coded [1.67% Coverage]

Reference 1 - 1.67% Coverage

I: Do you receive feedback on AEFI cases from next level

P: like as you said if we having reported how will we receive but anytime we report we received

<Internals\\IDIs DHMT\\NRDI_14> - § 1 reference coded [2.83% Coverage]

Reference 1 - 2.83% Coverage

I: Do you receive feedback on AEFI cases from the next level

P: apart from what am describing no

I: Why?

P: This what I said from the initial stage that sometime some see it but they don’t report it so for that matters I haven’t received any

<Internals\\IDIs DHMT\\NRDI_15> - § 1 reference coded [3.20% Coverage]

Reference 1 - 3.20% Coverage

I: Do you received feedback on AEFI cases from the next level?

P: laugh… no please [ ] baby scream

I: Why? [ ] baby cry, scream

P: Baby scream cry ….

P: I cannot best tell

<Internals\\IDIs DHMT\\UEDI_01> - § 1 reference coded [3.00% Coverage]

Reference 1 - 3.00% Coverage

I: ok so do you receive feedback on adverse events following immunization cases from the next level?

P: yeah we do receive, because even though I have not heard about eerrm anything like that but we do receive a lot of feedbacks(ok) from both national and region (ok) so I think if if there had been any case like that that was sent, we would have definitely received the feedback

<Internals\\IDIs DHMT\\UEDI_02> - § 2 references coded [6.99% Coverage]

Reference 1 - 4.06% Coverage

I: So how did you receive those reports?

R: Eh we you know for instance when we were doing the malaria intervention for instance eh we had a few of them, so we normally just the sub-district will report to us with a form (Ok), and we return report it to the region (Ok). That is how we do it.

I: So like those reports you send to the region are they sent electronically or

R: They’re hardcopies (hardcopies), they’re hardcopies. But you know when we are doing the report, when we finish the hard report sometimes we send it by email (by email), so the information the report will be captured in the email.

Reference 2 - 2.94% Coverage

I: Ok. So do you receive feedback on adverse events following immunization cases from the next level?

R: Hmm. That’s an interesting question… eh as to whether the concessive I’m not aware of something like that. Ah I’m not aware of something like that. I haven’t received one.

I: Ok.

R: Because honestly, it has to be analyzed to, and the feedback will be given to us (yeah), but more often than not we don’t (you don’t) we don’t get it.

<Internals\\IDIs DHMT\\UEDI_03> - § 2 references coded [5.28% Coverage]

Reference 1 - 2.06% Coverage

I: Ok. So meaning it’s only one paper that you send it to the (yes). Ok. So do you receive feedback on adverse events following immunization cases from the next level?

P: Actually, I never received any any information (Ok). That is the facts (Ok), or they send it and I was not there to receive it (Ok). But I per se, I’ve never seen a feedback that come with saying that O these were the thing that happened from Pusiga district, and this is the feedback on what you reported (Ok). I never received something like that before.

Reference 2 - 3.23% Coverage

I: Would you happen to know why that is happening?

P: Actually yes. I’m supposed to know (Umm), but the only thing is that they’re not giving the feedback in the sense that… you know Ghana Health Service here in particularly (Umm)… you know sometimes program will come and jump up (Umm). We’re doing this, we’re doing ABCD, we’re doing ABCD, we’re doing ABCD. The time you realize, the time even self you supposed to ask of the place (Umm) definitely, how was my reports and all those things, meaning they will also be started asking another people the drugs board or national for the report (Ok), and then the report will not come (Umm). Today even self, and then maybe immediately you’re in eh your attention has focusing on something, the thing will escape your mind because you’re doing a different thing (Umm) at that time.

<Internals\\IDIs DHMT\\UEDI_04> - § 1 reference coded [3.26% Coverage]

Reference 1 - 3.26% Coverage

I: okay and how were these forms also received, in what form or format.

P: the same format as we report all

I: okay, sir thank you. So we can say that by what you are saying feedback is gotten from the next level of reporting.

P: yes for the SMC we haven’t a feedback yet. We ended at in October and I have also been away for some time so I haven’t had a feedback but the previous ones yes we have

<Internals\\IDIs DHMT\\UEDI_05> - § 1 reference coded [2.15% Coverage]

Reference 1 - 2.15% Coverage

I: okay, so do you receive feedback from adverse event cases from the next level and that should be the region I guess?

P: yes once in awhile we do receive some from them.

I: when you say once in awhile...?

P: as and when we send the case, some they give us feedback from the regional level.

<Internals\\IDIs DHMT\\UEDI_06> - § 4 references coded [19.50% Coverage]

Reference 1 - 8.27% Coverage

I: okay so what might be some of the reasons why healthcare workers who encounter AEFI’s do not report?

P: knowledge is one, inadequate knowledge; they don't attach any seriousness to AEFI, that is one. Two, when the person comes and has no health insurance and the treat or they manage the person, they will have to pay, if the person, and we tell them that they shouldn’t charge anybody for anything, so if they shouldn’t charge anybody and the person come, they will document, for instance, this one I just opened to you, they have, the managed them, you see they manage the cases, now maybe you have to refund the money to them, we don’t or we don’t do that, so how then will the person report again next time that they see another case, definitely he will not like to do that, ahaaa, sometimes too they do they they they they accept the case, they accept the caregivers complain that yes this is AEFI, they will fill the form but because of distance from there to the DHA level, the district level for us to also forward it to region, they will like to keep the form there and will not even do anything with it then when it come to, I have submitted a lot of forms, me in particular fro 2012, I have being submitting AEFI forms, you don’t hear anything, I dealt directly with Food and Drugs Board when they were implementing ...(inaudible word) A and this Rhotarix and Pneumococcal vaccine back then, when you send the forms at least you should hear feedback that what you brought was maybe not AEFI or it was due to this or nothing, you understand that, ahaaa and that will make of alot of meaning but you don’t hear anything from them, (chuckles) at times they feel like ahhaaa there is no importance in reporting.

Reference 2 - 5.74% Coverage

I: so will there be any other thing?

P: yeah there will be a lot of things but just that I I, sometimes times too it’s possible that it can't reach here and we will not submit it to region...

I: what will be the reason?

P: same means, same means at times you have sample here, how will it get to region, only one car in the district, so when the car is going and you are not even in the known so the more the forms delay with you it becomes like “kai”-(chuckle), there is no need I report on it, ahaaa let me leave it like that, or we all come in and technology, technology you see if there is something like offline form that you are filling even over there when they give you, when they call you and give you the information, you can just open and fill it and easy way that they can have it there, maybe if that you are sending it through WhatsApp or through mail or something like that, it will be easy, but they want everything hard copy everything hard copy. When I fill hard copy and if region, my colleague is going to region and am here and its soft copy, I can send to him on WhatsApp and they all, can even forward it to you straight away, but it’s true, the hard copy is only part.

Reference 3 - 0.87% Coverage

I: okay, thank you, please do receive feedback on AEFI cases from the next level of reporting.

P: noooooooooo.

I: not at all?

P: not at all. Me, no. I have never received any ...

Reference 4 - 4.62% Coverage

I: why do you think there is that lapse, you don’t get feedback?

P: sometimes we feel like they don’t even do anything on it. That’s how some people in the village feel but mmhm because they don’t also pick sample, you see for example, when I send measles, when am sending measles case base form, it goes with sample, so they will check my sample and tell me that your sample was negative but in this case they don’t you don’t, it doesn’t go with sample just the form that goes that this person presented this with this but we indicate on the form the type of vaccine given, the batch number of the vaccine, so I sometimes feel like just that vaccine that they will check on to make improvement or they will say its program error at this end, so maybe if there will be specific samples that we need to pick I don’t know but maybe with the serious maybe there will be a need for us to pick some sample aaaah, but it doesn’t go with sample so we don’t get the ....

<Internals\\IDIs DHMT\\UEDI_07> - § 1 reference coded [8.73% Coverage]

Reference 1 - 8.73% Coverage

I: okay, sir do you receive feedback, once you are the district from the region upon reporting any case.

P: eerrh, I can’t quiet remember the feedback received but I know we receive feedback in adverse drug reaction but not on the adverse events following immunization (inaudible audio with voices still heard in the background) but am not so sure about that.

I: and do the facilities where these cases come from get feedback too from you.

P: usually when we submit when there are issues with the filling of the form they are immediately given feedback.... (inaudible)... and as I said I can’t be sure if we received feedback from above we in turn give them the feedback since am not sure whether we received feedback from the region, I can’t say much about that.

<Internals\\IDIs DHMT\\UEDI_08> - § 1 reference coded [3.67% Coverage]

Reference 1 - 3.67% Coverage

I: okay, so case that you send, probably if not in the last year, maybe the year before when you send a reported case of an adverse event, were you able to get any feedback?

P: yeah they do.

I: so in that case how is the feedback given?

P: some they call, it’s through call to give the feedback, that’s what I know because we are in the same office, sometimes they call them, what you sent is this this that, so its through call.

<Internals\\IDIs DHMT\\UEDI_09> - § 2 references coded [5.01% Coverage]

Reference 1 - 4.45% Coverage

M: Do you receive any feed backs on AEFI cases from the next level?

R: Yes from the few we send we receive feedback and that could just be phone call. That could be the easier way.

M: So the feedback is just based on phone calls alone?

R: No they send it through the paper form but getting it fast, they can call and say this and this you send to us, this is it but we will send the feedback later.

Reference 2 - 0.56% Coverage

M: Do they send the forms later?

R: Yes they send

<Internals\\IDIs DHMT\\UEDI_10> - § 3 references coded [7.42% Coverage]

Reference 1 - 0.84% Coverage

M: Do you receive AEFI’s feedback from the next level?

R: I haven’t received any report yet so far.

Reference 2 - 3.11% Coverage

M: What could be the reason why when you send reports you don’t get feedback?

R: Usually you know when we submit the report to FDB, they are supposed to come and do the pharmacovigilance to see whether it is true or false at least do something. I cannot explain what is happening or ascertain at their level. I always ask them and they say they haven’t receive the feedback.

Reference 3 - 3.47% Coverage

M: You mention a word like pharmacovigilance

R: Yeah

M: What do you mean by that?

R: You know the food and drug board they are to ensure that the drugs being administered to the target group or people are potent and being put to the right people. So they are supposed to monitor whatever drugs that is been given out and if there are any adverse reactions they should pick the drug and go see what is happening.

<Internals\\IDIs DHMT\\UEDI_11> - § 1 reference coded [5.37% Coverage]

Reference 1 - 5.37% Coverage

M: When you forward your report to the regional level do you receive any feedbacks from them?

R: For now I can’t remember but I remember years back we received feedback from food and drugs board it was a programme that was running and they did it in such a way that if you forwarded the information, your data was captured there and a feedback was sent direct to you to know that the work you did they appreciate it and I think a few staff from this districts benefitted from that. I think it was so nice to do because whoever that pick the form will know that the work you do people at the higher level have seen it and appreciated it and are saying “ ***ayeekoo”*** (Thank you)

<Internals\\IDIs DHMT\\UEDI_12> - § 1 reference coded [2.02% Coverage]

Reference 1 - 2.02% Coverage

I: thank you, do receive feedback on AEFI cases from the next level which will be the region?

P: okay, eehhh yes actually when I talk of the programme time, you see at the end of the programme, they should give feedback on what we have reported then we also disseminate to the sub-district level and the community members.

<Internals\\IDIs DHMT\\VRDI_02> - § 1 reference coded [2.34% Coverage]

Reference 1 - 2.34% Coverage

Errm do you receive feedback on the adverse events following immunization from the higher level?

P: For the period I’m here I’ve not…

<Internals\\IDIs DHMT\\VRDI_04> - § 1 reference coded [3.36% Coverage]

Reference 1 - 3.36% Coverage

I: Oh okay! Errhm, do you receive feedback on the AEFI cases once you refer them to the next level?

P: I remember fortunately, Dr. Atsaney was at that meeting and and I…I happened to know him so, sometimes I even call him to seek guidance in the process to to know what exactly to to to do and and he he he calls… At one point in time I remember receiving a call from Pamela at the national EPI, I think Pamela Koomson. She called that she has received, I think one of our yh! our forms.

<Internals\\IDIs DHMT\\VRDI_06> - § 1 reference coded [7.13% Coverage]

Reference 1 - 7.13% Coverage

I: alright! Do you receive feedback on the cases from the next level when you refer them?

P: errhh when we refer, basically the issue is that we don’t receive that much feedback, but when we do, errh erhh… it’s mostly not a significant…

I: yh!...

P: errhh eheh… because sometimes although we’re expecting always, the batch… the vaccine batch, was there an issue or there… what about that… sometimes… *inaudible*

I: …they never really talk about it…

P: eheh!

<Internals\\IDIs DHMT\\VRDI_07> - § 1 reference coded [4.10% Coverage]

Reference 1 - 4.10% Coverage

I: Ok, ok. Do you usually get feedback from ahm the region?

P: Yes we do, erhm but not all time.

I: Ok.
P: Is not all the time,

I: Ok

P: is not all the time. I remember was it a year or two years ago, Avemectin distribution, there was one particular officer at the border, border post. I think he had something just like Stephen Johnson syndrome.

I: Ok.

P: But we made all the report and then went to …virtually we send… Accra requested for the pictures and we send all but I thought there would have been a feedback on what actually was happening in my…. But I don’t think I had any feedback.

<Internals\\IDIs DHMT\\VRDI_08> - § 3 references coded [6.57% Coverage]

Reference 1 - 1.45% Coverage

I: When you submit report of this nature, do you usually receive feedbacks?

P: Ehmm, I will say that errh, for now we haven’t send any report.

I: Ok.

Reference 2 - 0.57% Coverage

P: so definitely we will not receive any feedback.

I: Ok.

Reference 3 - 4.55% Coverage

I: Ehmm, is it that you sent a zero report or you haven’t sent a report?

P: Yes, we have a, a monthly reporting format where there is… we have to indicate that one. So it means is a zero reporting we have being sending. So well, if it’s zero reporting, it means they could have query us why we are sending zero report and that we should put in more efforts to identify adverse events and report on them.

I: and report on them?

P: Yes, ok that you are right in that aspect.

<Internals\\IDIs DHMT\\VRDI_09> - § 2 references coded [4.90% Coverage]

Reference 1 - 1.64% Coverage

I: ok, so then again, I will ask how often do you receive feedback from, from, from the region?

P: yes, in terms of AEFI, in terms of AEFI, erhmm, I would say the, the feedback from the region is more often as and when they decide to do may be a quarterly ehrr report feedback

Reference 2 - 3.26% Coverage

I: ok

P: to districts concerning number of AEFI cases received or whey we meet for, may be annual or half year reviews, they present on that. With, with the AEFI in particular, because most of time it does not involve picking of samples to the lab,

I: ok.

P: we don’t receive that feedback.

I: ok.

P: But if it has to do with any other case investigation for which sample is taken and send to lab, with that as and when they also receive results from the lab, they forward it.

I: ok

P: they share it with all of us, to our common platform.

<Internals\\IDIs DHMT\\VRDI_11> - § 1 reference coded [4.68% Coverage]

Reference 1 - 4.68% Coverage

I: Please with respect to the reporting, you mentioned about how they fill the form, and then they pass it over the way to the regional level, so when reports are sent to the next level which at your level is the region, do you usually receive feedback on the report?

P: No, we even, if the feedback is sent, it is directed to the director and then… but as I said is errh… I can remember the last time we even receive one, let along expects the feedback but errh since we have our own unit, if there is one sent that I am not aware then maybe the disease control will answer that one.

I: Ok. Emmh, so it means you can speak to that issue?

P: Yes.

<Internals\\IDIs DHMT\\VRDI_13> - § 1 reference coded [3.77% Coverage]

Reference 1 - 3.77% Coverage

I: Ok ….ok do you receive feedbacks on AEFI cases from the next level?

P: Ooh sure yes, yes whenever we send AEFI issues to region they don’t we have platform that we discuss all those things whatsapp platform that they will mention your district and whatever action they were been able to take on your eere case we discuss it at the platform and at times they even send you letter as feedback.

<Internals\\IDIs DHMT\\VRDI_14> - § 1 reference coded [2.77% Coverage]

Reference 1 - 2.77% Coverage

I: Oh ok do you receive feedbacks on AEFI cases from the next level?

P: Next level?

I: Yes the ones you send to maybe region?

P: This one it is disease control that can answer.

I: Ooh ok so you don’t know?

P: When they bring the forms they send it to that place,

I: The disease control.

P: Aahaa.

<Internals\\IDIs DHMT\\VRDI_15> - § 1 reference coded [2.48% Coverage]

Reference 1 - 2.48% Coverage

I: Ok thank you did you receive feedback on AEFI cases from the next level

P: No.

I: Which is the regional?

P: yes,

I: you are district, you don’t receive?

P: No.

<Internals\\IDIs FDA\\GAFDA_02> - § 1 reference coded [3.73% Coverage]

Reference 1 - 3.73% Coverage

I: And then how did you receive those reports?

P: These reports, they came as a hard paper copies and then we enter them into the ‘erh’ database. Before I continue there is also an excel, but what we do is that we, what we enter into the safety well system is an E2B complains database but we can extract from there into excel so the report. as it is exist now has an online electronic form and has an excel database.

<Internals\\IDIs FDA\\NRFDA_01> - § 2 references coded [3.75% Coverage]

Reference 1 - 1.93% Coverage

I: “umm” how did you received those reports?

P: because we have a meeting, am a member of the regional health team

I: ok

P: so every Monday, we attend the meeting so if there is any serious reaction, the disease control officer or public health nurses come to report and we brainstorm on that and then if the need be action is taken

Reference 2 - 1.82% Coverage

I: do you receive feedbacks on adverse events following immunization cases from the next level?

P: oh yes of cause, what the do is like as I said all the reactions are collated, they are sent to the National Pharmacovigillance Centre in the head office of FDA. When something serious happens, for instance in my region

<Internals\\IDIs FDA\\UEFDA_01> - § 1 reference coded [3.69% Coverage]

Reference 1 - 3.69% Coverage

M: When you received the cases of the report and forward it to the next level do you receive feedback from the next level?

R: Yes like I said, we receive feedback and we also send it to those who send us adverse events report when you send us adverse events report. When you sent us adverse events report we also forward it to our national level. They give us feedback and we also send it to you so that you know that attention is also being given to your report and whatever outcome we also communicate back to you
