# Supplementary material for: Optimising reporting of adverse events following immunisation by healthcare workers in Ghana: A qualitative study in four regions
Source: PLoS One. 2022 Dec 20;17(12):e0277197. doi: 10.1371/journal.pone.0277197 (PMC9767370; doi:10.1371/journal.pone.0277197)
Supplement: S1 Data — (ZIP) [file pone.0277197.s001.zip › Minimal data/S8 Giving feedback on AEFI reports.docx]

**Name:** 15. Giving feedback on AEFI reports

**Description:** This node contains all information related to giving feedback on adverse event reports.

<Internals\\IDI EPI\\GAEPI_01> - § 1 reference coded [2.68% Coverage]

Reference 1 - 2.68% Coverage

I: is it, doc do you give the feedback to, on AEFIs to those who report to you?

P: yes.

I: okay

P: it’s normally, as we send it to FDA, FDA gives feedback and we also send the feedback to the reporting regions, to the reporting districts.

<Internals\\IDI RHMT\\GARI_01> - § 1 reference coded [8.84% Coverage]

Reference 1 - 8.84% Coverage

I: so do you please ‘erh’ give feedback to ‘erh’ those who report the AE, AEFI cases

P: yes. If we get feedback we also give it to them. For example, let me put AEFI aside. Those who bring the ‘erh’ measles specimen and the rest, when they send it to the lab, they give us [inaudible], if we get ‘erh’ information from the lab we also send it to them and then even to the facilities that are reporting ‘erh’ that reporting, so that they will know their work is not in vain. It is not that, just that you have reported and nobody is minding you. Because this food and drug authority people when they receive such thing they look into it and then they definitely give feedback. Like I said from the beginning, if they don’t find anything wrong, then it could be like I said progra.., it could be program error. Program error

I: administering the wrong vaccine?

P: no, no, no! not administering a wrong…it ‘erh’ the proper vaccine. When we talk about program error, I mean instead of giving IM, you give subcuts. You know ‘erh’ abscess are all adverse event so why should the person get *abscess*? Then it means you didn’t *administer* the thing correctly. Why is it that you administer measles antigen and the person will get measles? It means either the chain was broken along the line

<Internals\\IDI RHMT\\GARI_02> - § 1 reference coded [5.90% Coverage]

Reference 1 - 5.90% Coverage

I: talking about feedback, it looks like you give ‘erhm’, you people give ‘erhm’ feedback on AEFI cases to those who report them too

P:’ erh’ yeah. If you get the feedback from where you have sent the report, definitely you will send the feedback down there to those at the lower level. But if you don’t get feedback, how do you also send the feedback to them ‘uhuh’. So I’m sure when the feedback comes they will also give. ‘Cos’ I… they don’t bring the err reports to me. The forms don’t pass through me per our system. ‘Uhuh’, it has to pass through the surveillance officers, so everything goes through them and then if there is a feedback, it will come to them and they will now have to send to the lower levels. Yeah.

<Internals\\IDI RHMT\\NRRI_01> - § 1 reference coded [3.79% Coverage]

Reference 1 - 3.79% Coverage

I: Ok, Do you give feedback on AEFI cases to those that report to you?

P: Absolutely like we have never met at fora that we talk about surveillance activities that AEFI is not spoken of or EPI especially when EPI meetings are coming, so AEFIs will always be spoken of and that is by way of feedback and we also have bulletin that we also send to, there are several ways, we have platforms that we normally deals with disease control officers, we also have platforms that deal with the region, region specific platforms and we have other small small groups that we have platforms on so these are more often than not, fed with whatever information there is and as far all surveillance activities including AEFIs are concern.

<Internals\\IDI RHMT\\NRRI_02> - § 1 reference coded [0.82% Coverage]

Reference 1 - 0.82% Coverage

I: Do you give feedback to feedback on AEFI to those that report to you?

P: That’s why I said that you know once they don’t report to me. “en hrrr”.

<Internals\\IDI RHMT\\UERI_02> - § 1 reference coded [3.30% Coverage]

Reference 1 - 3.30% Coverage

M: Do you give feedback to those who report AEFI cases to you?

R: Yes we do. When we collect the data and send to the next level. The best we can do is that, if it is quarterly, monthly reports, we share the data on all the issues and events including AEFI’s we indicate the reports risk of each district to encourage them. What would have made it to be complete the final issue? If it is goes there and it is discussed. What is the duration on each individual case reported, that is often lacking and that is beyond our level apart from telling them what they have within a particular period.

<Internals\\IDI RHMT\\VRRI_01> - § 2 references coded [8.56% Coverage]

Reference 1 - 6.45% Coverage

P: Yes, electronically, so he stores them. And he also forward to national.

I: ok, emmh, do you usually give feedback? I mean is there a feedback system?

P:Yes, for feedback, we have not being receiving feedbacks so much from national because we don’t actually do much with the forms but we call them back and make sure they go and like to monitor the condition. Ahaan, so when they report to us, we, we establish communication link with them to find out how….

I: you mean the districts?

P: The districts but usually, errh, like the other surveillance areas like AEFI, erh! AFP, measles and…., national gives us feedback, we also give feedback to the districts but for AEFI usually we don’t have feedback, any feedback from national for as to give to the districts

I: so

P: but we don’t know whether national gives direct feedback to the reporting units *(laughs)*. Ahaan

I: So because, if I got you right, you don’t receive feedback from national,

P: yes on AEFI

I: so that onward transmission, downward transmission to the district level also don’t come

P: Is also lacking

I: ok

P: Yes.

Reference 2 - 2.11% Coverage

I: ok

P: so we also give the feedback. Because on EPI page I think they reported…, the national put something that one in errh, is it Western Region or so, was a serious AEFI and some action should be taken but if they can give feedback on the non-serious ones, all of them so that we also give information to the lower levels. I think that one can also help.

<Internals\\IDI RHMT\\VRRI_02> - § 1 reference coded [1.00% Coverage]

Reference 1 - 1.00% Coverage

I: ok. So what about, from the region to the district?

P: We do.

I: You give them feedback?

P: Yes that one we do.

<Internals\\IDIs DHMT\\GADI_01> - § 1 reference coded [2.11% Coverage]

Reference 1 - 2.11% Coverage

I: So do you give feedback to those that report to you?

R: Well I don’t get feedback so I can’t give them feedback as well.

<Internals\\IDIs DHMT\\GADI_02> - § 1 reference coded [4.18% Coverage]

Reference 1 - 4.18% Coverage

I: Do you give feedback on the AEFI cases to the care givers?

R: Definitely we give feedback to the community, patients and the sub district and the nurses so when another issue happens they know what they should do.

<Internals\\IDIs DHMT\\GADI_03> - § 1 reference coded [4.42% Coverage]

Reference 1 - 4.42% Coverage

I: Do you get feedback on the cases you report?

R: The feedback is usually verbal; because we work in chip zones when the mother comes for the next weighing then she tells the nurses what, sometimes they go for home visit and find out how the child is improving and what has been done. It’s just verbal feedback no written one.

<Internals\\IDIs DHMT\\GADI_04> - § 1 reference coded [3.89% Coverage]

Reference 1 - 3.89% Coverage

I: Do you give feedback on the cases you reported?

R: For the past one year we have not had any report so I can’t be sure.

I: What of the previous cases?

R: There was one in the previous year that we heard and we followed up.

<Internals\\IDIs DHMT\\GADI_05> - § 1 reference coded [6.17% Coverage]

Reference 1 - 6.17% Coverage

I: Do you give feedback on the cases of AEFI reports from the next level?

R: Because I don’t receive such forms I can’t comment on it.

I: So you can’t be able to tell whether there is a feedback from the next level?

R: No, I think the district pharmacist would be the best person to tell you.

<Internals\\IDIs DHMT\\GADI_06> - § 1 reference coded [3.78% Coverage]

Reference 1 - 3.78% Coverage

I: So do you give feedback on the cases that you receive?

R: Yea, as I told you we have not any reports yet.

I: So what of previous years?

R: Yes but seriously when they report we do our three quarter meetings, half year, quarter and full year we raise these issues those doing well and those not doing well, we bring all those issues up for discussions so we do all that but we don’t have a fixed time but when we have these meetings that is when we do all these feedbacks but it is oral.

<Internals\\IDIs DHMT\\GADI_07> - § 1 reference coded [6.21% Coverage]

Reference 1 - 6.21% Coverage

I:And do you as well give feedback to those who report them?
P: yes we also give feedback to those who report to us at the lower level.

I: we would love to know why

P: why we give them feedback?

I: yes please

P: oh, It’s, that one it’s only to ‘erh’ let them know that ‘hmm’ what they sent to us through the system ‘hmm’ we are aware and we also ‘erh’ inform them to at least motivate them to continue doing that one, because if they send the report and they do not receive feedback they will think that well ‘erh’ that report is not appreciated ‘erh’

<Internals\\IDIs DHMT\\GADI_08> - § 1 reference coded [4.89% Coverage]

Reference 1 - 4.89% Coverage

I: What about feedback on AEFI cases to those who report them to you?

P: Because I don’t receive it from the region I don’t also give them the feedback…that is the challenge. If I send it like when we are taking a sample of a condition when I send it to Accra or to the lab, I inform the region people because I send a copy, they will alert the region and the region will forward whether positive or negative and I will let the person who reported know. But this one I will send I don’t get any feedback.

<Internals\\IDIs DHMT\\GADI_09> - § 1 reference coded [1.46% Coverage]

Reference 1 - 1.46% Coverage

I: if that is the case, do you also give feedback of AEFI cases to those who report them?

P: noo, you mean the clients?

I: yes the clients

P: No, no, we don’t, we don’t…we don’t give any ‘erh’ yes.

<Internals\\IDIs DHMT\\GADI_10> - § 1 reference coded [8.22% Coverage]

Reference 1 - 8.22% Coverage

I: I don’t know whether you can answer, do you give feedback on AEFI cases to those who report them?

P: If it goes through and then we get because this how the channel goes ‘err’ the, the, the information comes from the communities through us, so we ‘er’r it is not in our I say power to give them a feedback. The only feedback we could give them is that it’s been sent but they would have to now investigate and then give us the proper feedback, because the feedback we give them should be feedback we receive from food and drug’s board. Yes!

I: Because the next question was saying, why do ‘err’ do you give feedbacks to those who report them?

P: If you give feedback it’s all for the right reasons; but one; we want them to look out for it, to always search to ask the probing questions that I mean, when, when, when ‘erhm’ mothers come back, you try to find out….the last ‘erhm’ vaccination you came for what happened? You know, did you see this? Did you see that? Was there rash? Was there fever? Did the child even look like he or she had collapsed? You know all that. So if those are…if you give them those types of feedback I think it will help.

<Internals\\IDIs DHMT\\GADI_11> - § 1 reference coded [5.42% Coverage]

Reference 1 - 5.42% Coverage

I: do you also give feedback to ‘erh’ on AEFI cases to those who report them to you?

P: yes.

I: we would also like to know why you do that.

P: so that they will know the reason why it has happen. If it’s the vaccine then they will withdraw from their end to the district but if it’s not the vaccine then you will…tell them what to do, unless [inaudible] we should know what to do.

<Internals\\IDIs DHMT\\GADI_12> - § 1 reference coded [6.26% Coverage]

Reference 1 - 6.26% Coverage

I: and do you give feedback on AEFI cases to those who report them?

P: yes, yes.

I: why, why do you do that?

P: ‘erh’ what we do is we make sure after the, they have brought the children and then the MAs will assess, if they assess and it is like I said some of them will be a mere fever, they will treat them and the charge is free. So we give feedback to them that, look we have treated this person, the MA didn’t take, the facility didn’t take any money, it is free. So that the person will also know that I have brought somebody and the person is being catered for.

<Internals\\IDIs DHMT\\GADI_13> - § 1 reference coded [3.70% Coverage]

Reference 1 - 3.70% Coverage

I: Also please ‘erhm’ do you people give feedback on AEFI cases to those who report them?

P: NO! It’s a shortfall in our system, it’s a short fall in our, short fall in our, in our, in our system. We don’t do that very well, I must say. We don’t do that. When the reports go and we have like, feedback by way of summaries we don’t…. what we do is sit down with them and ask them why you are not reporting, that kind of thing but like individual AEFI’s…

<Internals\\IDIs DHMT\\GADI_14> - § 1 reference coded [4.55% Coverage]

Reference 1 - 4.55% Coverage

I: Do you also give feedback on Adverse Events cases to those who report it to you?

P: Like the caregivers?

I: Yes

P: [participant fanning] then those at the, then the in-charges in the field will have to answer that because they will not tell me, so in the field the in-charges there they will be giving the feedback to the caregivers because I don’t deal directly with caregiver [participant fanning].

<Internals\\IDIs DHMT\\GADI_15> - § 1 reference coded [8.46% Coverage]

Reference 1 - 8.46% Coverage

I: having said that, do you also give feedback to “err” those… [Interrupted by participant]

P: the facilities?

I: yeah!

P: yes, yes, we give feedback and we even do follow ups to ensure that whatever child that is affected fully recovered.

I: is that all?

P: yes. When we, apart from we giving then the feedback that we have sent it to the region and they have sent the form to the various authorities, we also ensure that the treatment for the kids management is free at the facility. Director have, they have a letter from director that AEFI is supposed to be managed free and then they should keep copies of the receipts and if there is any money then you refund to them. So we ensure that whoever is affected is being managed free of charge.

<Internals\\IDIs DHMT\\NRDI_01> - § 1 reference coded [4.80% Coverage]

Reference 1 - 4.80% Coverage

I: Ok so do you give feedback on AEFI cases to those who report them to you?

P: Feedback?

I: hmm

P: That is what I was saying mostly the communication is done when you send the this thing to the disease control officer in the district and he also forwards to region and whatever the communication is done between the region and the disease control officer at the district and he also communicate back to the particular facility that has reported. So from there the health workers that are there will also communicate to the family or the parents involved.

<Internals\\IDIs DHMT\\NRDI_02> - § 1 reference coded [3.81% Coverage]

Reference 1 - 3.81% Coverage

I: Sir how about those who report them to you do you give them feedback?

P: *Oooh* I don’t think so because if if I have not received feedback about what really was the cause eeh what are my going to tell to those who report. The only thing I can tell those who reported is that I have received you form as to whether what it was somebody else at the higher level will have to make that determination and then get back to me.

<Internals\\IDIs DHMT\\NRDI_03> - § 1 reference coded [4.18% Coverage]

Reference 1 - 4.18% Coverage

I: Do you give feedback on AEFI cases that you receive?

P: Because I have not receive anything normally I have not given any feedback but normally what we will do is that we monitor monitor the person we monitor child and see how he is improving if he is body temperature we assume that we do some other other eeh prevention measures and that it will subsided and then it be off.

<Internals\\IDIs DHMT\\NRDI_04> - § 4 references coded [6.22% Coverage]

Reference 1 - 1.51% Coverage

I: Do you give feedback on adverse events following immunization to those who report to you?

P: Yeah, yeah within the period you have ask yes, because the woman I saw I even went to the house…. In two days time errrh three day time I went to the house, saw that the child was now the leg was now better.

Reference 2 - 1.11% Coverage

I: I am not talking of the care giver but your other staff who are under you who report their cases to you

P: Yeah when they report to me, because we have not receive any report. So there is no feedback in that regard. Yeah

Reference 3 - 0.59% Coverage

I: But when they give

P: When they gave us we, we gave them the feedback, we (inaudible) and give them the feedback

Reference 4 - 3.02% Coverage

I: So why do you give them feedback?

P: We give them feedback to know the situation at hand now, how the level we have manage the case to. That helps them…. Whether the case is, the case is doing well or doing bad them it prompt them next time they have to report as quickly as possible or if were the case is doing bad then probably it could be due to the …. Delay in reporting. If they had reported earlier we would, have, would have interventions would have put in place so is just to help them try as much as possible whether they should report quickly when (inaudible) happens….. that’s why we give feedback

<Internals\\IDIs DHMT\\NRDI_05> - § 1 reference coded [5.77% Coverage]

Reference 1 - 5.77% Coverage

I : Okay, thank you sir, we are almost getting to the end of our interaction but I will like you to emmm tell me whether you have given those who have reported to you...r level feedback.?

P : Emmmm ......... Even if you have given a feeding any feedback emmm may be just to tell them you ‘v receive the form probable ehh may be probable verbally is transmitted to the next level or sent to the next level. But ....... Because we dont also receive feedback ..... we are not able to give them any feedback. Ehhh because whatever it is emmm it has to be taken care of elsewhere. If it is that it has to be accompanied by some samples for further investigation then there is a feedback it has to we also give them feedback but usually what we do is that say if it is ehhhh may be the child is having fever we just encourage them to tell the mother to take a sponge or may be if it is an abscess they get they get ehh a cold pack may be try to put on the site to see how .... it can ehhh ..... reduce or stop totally. Apart from that but as at when we also get feedback .... from the next level of course we have to also get them a feedback.

<Internals\\IDIs DHMT\\NRDI_07> - § 1 reference coded [3.58% Coverage]

Reference 1 - 3.58% Coverage

**I:** OK. Do you give feedback on AEFI cases to those that report to you?

**P:** yes, they too know happened is that, because they also just brought and it just means nothing, we don’t also actually officially report back until when you hear May because what they are already bringing they themselves know that it is something that will be followed. Ahh, so when we also send it to the next level we also ideally know nobody will follow up because what is been reported is of no heighten value, ahh… so when we also just they too they will not also ask us when we reported we didn’t hear anything because they know in the same way when we also go the region we don’t ask that we reported AEFI and what did you say?

<Internals\\IDIs DHMT\\NRDI_08> - § 1 reference coded [3.27% Coverage]

Reference 1 - 3.27% Coverage

**I:** Do you give feedback on AEFI cases to those that report to you

**P:** Yes they do

**I:** Why do you give feedback?

**P:** Ah, because, when you refer a case you have to follow up and know how the case ended so the AEFI adverse event when it happens and you brings, you have to know it end and then you take precautions for next time

<Internals\\IDIs DHMT\\NRDI_09> - § 1 reference coded [5.20% Coverage]

Reference 1 - 5.20% Coverage

**I:** Do you give feedback on AEFI cases to those that report to you

**P:** Definitely we do.

**I:** Why do you give feedback?

**P:** We do that to boost their morale, more especially we have common platform that every staff sees the information that we are giving to the lower level and add additional information to let those who are not reporting to know that, it is good to report rather that to see it and cover up. So we give them feedback to encourage those who report to report more and those are not reporting to also try to report.

<Internals\\IDIs DHMT\\NRDI_11> - § 1 reference coded [4.85% Coverage]

Reference 1 - 4.85% Coverage

I: Mummy, do you give feedback on AEFI cases to those that report to you?

P: To those that report to us?

I: Yes.

P: We do.

I: Why do you give the feedbacks?

P: Is to allay the anxiety of the care giver or you mean to our health staff? “Yaah”, we give them feedback for instance the cased that recovered, sometime even before we will see at our level, the facilities will even inform us that may be the case recovered immediately after this but whatever feedback from regional level, we give but as far as these 2 cases are concerned we haven’t gotten feedback and that is why we haven’t also given the feedback.

<Internals\\IDIs DHMT\\NRDI_12> - § 1 reference coded [5.64% Coverage]

Reference 1 - 5.64% Coverage

I: Those that you report to. Do you always get feedbacks from them?

P: actually me ah, when we give it to (ehiii) it must be the EPI people I don’t know, so actually they haven’t given any this thing feedback.

I: So why?

P: I don’t get any feedback from the this thing.

I: Why this?

P: I can’t best tell. I can’t best tell, if am going to answer you, I will tell , I will tell a lie why they don’t give feedback. ( voice of disease control officer) “mmm”.

I: Ok, so do you give feedback to on AEFI cases to those that report to you?

P: err, we call to tell them that, they haven’t come yet, the forms are there, they haven’t this thing. So bear is also a feedback, we are still waiting, we have process it.

I: Ok.

P: err

I: Ok, ok

P: So if I get definitely I will tell them, if I have anything we tell them the form we are still waiting to get the results.

I: Ok.

P: “hmm” feedback can be positive or negative, isn’t it?

I: Yes, yes, yes.

P: “hmm” so that is it.

<Internals\\IDIs DHMT\\NRDI_13> - § 1 reference coded [6.59% Coverage]

Reference 1 - 6.59% Coverage

I: Do you give feedback on AEFI cases to those that report to you

P: yes if they give they will receive but if don’t give

I: Why do you give?

P: for them to know that because at times some of them it might not even be due to the vaccine some children are already having some problems so when they take like recently like this we have the mass drugs distribution somebody was already hypertension but when he took the drug he thought it was the drug that brought but when he is now came to the hospital and look they saw that he was already hypertension person at times some of those things are there

<Internals\\IDIs DHMT\\NRDI_14> - § 1 reference coded [5.90% Coverage]

Reference 1 - 5.90% Coverage

I: Do you know give feedback on AEFI cases to regional or district level

P: This is what I have said again when apart from that particular case but was not last year we wanted the case to be actually AEFI case before we but when he came back from Wa it was Wa people who was attending to him as an old case he was attending Wa technical school so it was from there that we got to know that it wasn’t because of the drug so we just left it there because we knew that that wasn’t the case so we didn’t report.

<Internals\\IDIs DHMT\\NRDI_15> - § 1 reference coded [1.70% Coverage]

Reference 1 - 1.70% Coverage

I: So do you give feedback on AEFI cases to those that report to you? [ ] baby scream

P: Yes

<Internals\\IDIs DHMT\\UEDI_01> - § 1 reference coded [6.98% Coverage]

Reference 1 - 6.98% Coverage

I: so do you give like feedback on adverse event following immunization cases reported to you?

P: from?

I: to those that report to you, do you give them feedback?

P: yeah we do give them feedback

I: ok why do you do that?

P: eermm, its, builds the courage of the health worker himself

I: ok

P: because he gets to know that fine, the thing that happened and he or she was able to report has been solved and then the feedback, normally when you report on something and you don’t get feedback it demoralizes you (Yess) so when they carry the report and get a feedback, and especially if its positive like the patient is recovering or is responding to treatment, it builds up the morale of such a staff and he will continue to report as and when they get

I: ok

P: but if you get the feedback and you don’t send it to that lower level they don’t see the need why they should keep on

<Internals\\IDIs DHMT\\UEDI_02> - § 1 reference coded [8.10% Coverage]

Reference 1 - 8.10% Coverage

I: So do you give feedback on adverse events following immunization cases to those that report to you?

R: No, we don’t also.

I: Can you please tell me why?

R: Eh ehe you know we need to get the concessive factor before we can tell them (Ok). You understand (Ok). All these things we need to we want to find out the causes (Ok). So when we get the causes, we can now tell them to go and educate staff and the other the clients. But if we don’t have that information we’re unable to do that. So the feedback must come from the top (Ok) before we can be enabled to do that.

I: Ok. But like said you are not aware they you get the feedback. You’re supposed to get the feedback but instead

R: Yeah but we don’t we don’t have

I: Have you found out why?

R: No, we haven’t. We haven’t eh. But we just know that you know the analysis are quite complicated (Ok) so and can’t do that I’m not sure we can do that in the region (Ok). So now these things are done by the Food and Drugs authority (Ok), and it takes some time (Ok). Eh maybe that’s why – the process is long and also involve some… a whole committee has to sit down and involve some money (Ok). Maybe that’s why it doesn’t come to us (Ok). Yeah, I think so.

<Internals\\IDIs DHMT\\UEDI_03> - § 2 references coded [8.32% Coverage]

Reference 1 - 2.76% Coverage

I: Ok. So do you give feedback on cases eh adverse events following immunization cases to those that report to you?

P: Yeah! (coughs, clears throat). What we does here is that anything that you report to region, if they are able to send feedback, I was just trying to show you some feedback that they even, some place sent (Umm). I will print it because some of them don’t have emails (Umm). The only thing that I‘ll try to print it like yellow fever, YF and all those things (Umm), I’ll just try to print it. Have you seen it?

I: Umm.

P: And forward to the facilities that, or wherever they are their print they have it. So that you have it and see what is happening (Ok). That is the way I I I do here.

Reference 2 - 5.57% Coverage

I: Ok. So you give them feedback.

P: Yeah.

I: Why would you give them feedback?

P: They are the people who who who who saw the case (Ok). They’re the people. And they have to know what’s happening on my client (Umm). Maybe they have reported the case. If it’s not so serious, I may not go there maybe to see (that’s true). So they are the people who know the client, and know at the time when the thing happened (Umm); what actually happened; what was wrong with the client. Maybe they may say the client is doing ABCD (Umm). Maybe the time I got there, I may not see him (yeah). So that is why they supposed to have the information (Ok). And then from there, they should have been able to also tell the client (Umm). That is it. Because the client will also know that O they picked my ABCD to do and say they want to do ABCD to see what is happening to me when I took this injection, when I took this drug, and all that. So they should have also been… to what to get the client, but the difficulty thing that they will be getting from the client is that maybe the client… some of the clients they give wrong addresses (Umm, Ok). And if you give the wrong address, you may get Alhassan Memuna, especially Moslem communities (Umm). You see the names same. The house address (yeah). And you may get to the community and then… there’re so many things by that in that community (Umm). You see that even you cannot trace the client.

<Internals\\IDIs DHMT\\UEDI_04> - § 2 references coded [7.82% Coverage]

Reference 1 - 3.53% Coverage

I: what would you say ids the importance of sending the feedback back to the...

P: the feedbacks would really motivate for more reporting. It shows some acknowledgement. Whatever you sent has being received and is seriously being taken care of and that makes people to report more. So I previously heard a lot of complaints people were making that they were not feedbacks. For me all the report we give, it may delay but feedbacks come.

Reference 2 - 4.29% Coverage

I: so by that will you say that it has boosted the or there is an increase in the reportage of adverse events in your district.

P: not necessarily, aaah you know the factors that trigger the reporting one of them is the caregiver not reporting to the health worker. Once it doesn’t get to the health worker a report is not filled and forwarded. So at home there could be children with adverse events that we are not seeing and reporting. So they are just minor ones that are reported but there could be alot that we are not seeing

<Internals\\IDIs DHMT\\UEDI_05> - § 1 reference coded [1.56% Coverage]

Reference 1 - 1.56% Coverage

I: okay so do you also give feedback to the cases that are referred to you that is at the district level?

P: (cuts in sharply) yes we do but I think again disease control again would be in the best position to....

<Internals\\IDIs DHMT\\UEDI_06> - § 1 reference coded [2.11% Coverage]

Reference 1 - 2.11% Coverage

I: so by extension when you receive forms and you don’t get feedback from the next level do you also give feedback to the...

P: (interrupts shapely) no no no no, we don’t give feedback to anybody.

I: why will that be the case?

P: but we don’t,(laughs) we didn’t get any feedback so what feedback are we giving, so like it goes, it’s a one way communication, it goes like that but we don’t get feedback so ....(snaps fingers in despair).

<Internals\\IDIs DHMT\\UEDI_07> - § 1 reference coded [4.95% Coverage]

Reference 1 - 4.95% Coverage

I: okay, and what would be the importance of this feedback getting to the facilities?

P: feedback mmhm would help them streamline things for instance if the feedback points to the fact that it wasn’t due to the way the immunization was given then they have more confidence to work and report, alright if it’s the other way round then probably they will have to I mean it’s an entry point for re-training or orientation of the staff.

<Internals\\IDIs DHMT\\UEDI_08> - § 1 reference coded [13.83% Coverage]

Reference 1 - 13.83% Coverage

I: when you alos get such feedback do you also relay to where the case is coming from?

P: yes

I: why do you relay the feedback? (paused to avoid intrusion for about 2 minutes) so I was asking why you relay the feedback.

P: so that they will take an action on it, what has been reported to us.

I: when you say action, what do you mean?

P: lets take it, some time back, eerrh some children were brought in, they were injected then later on some rashes appeared, not knowing it was measles, mmhm so such a case from the feedback you have received is been tested positive that its really measles the person is suffering from, you have to call them, the action they will take is that, they have to go into the community and look at children who have been immunized around that same period and see whether the vaccine given wasn’t eerrh potent, you sometimes the way we handle it, the vaccine losses its potency and we end up injecting the children water, so when it happens that way, they will go back, check with related children within that same period that they were immunized to see whether they have also developed the same thing ,if it is so the that means they need to be treated by giving Vitamin A and then other care too will be given.

I: okay, will that be the only reason why you will be giving feedback to the facility?

P: mmhm, no. I think what eerrh, it could be that the nurse may not know these are signs of this condition, so through the feedback they receive they will learn that oooh this condition come with this sign so they will apply it to subsequent issues or conditions that will come at their facility.

<Internals\\IDIs DHMT\\UEDI_09> - § 1 reference coded [2.19% Coverage]

Reference 1 - 2.19% Coverage

M: Do you give feedbacks on AEFI’s cases to those who report them to you?

R: Yes we do, but like I said it is through the phone call we give them but for the hard copies to be moving like that no.

<Internals\\IDIs DHMT\\UEDI_10> - § 1 reference coded [1.22% Coverage]

Reference 1 - 1.22% Coverage

M: Do you also give feedback of an AEFI’s cases to those that report to you?

R: Yes I give feedback in the sense if it is given to me I tell them

<Internals\\IDIs DHMT\\UEDI_11> - § 1 reference coded [1.97% Coverage]

Reference 1 - 1.97% Coverage

M: When you receive reports from the sub-district levels. Do you give them feed backs?

R: Yes feedback in the form of phone call that we have forwarded the information to the region and if there is any feedback we equally give them the feedback.

<Internals\\IDIs DHMT\\UEDI_12> - § 1 reference coded [4.13% Coverage]

Reference 1 - 4.13% Coverage

I: okay so with your reported cases do you give feedback to where these reports are coming from?

P: yes, we also give the feedback to the sub-district to the community.

I: why do you think that this other chain is followed?

P: this is for it to get to follow so that the channel could be clear to the chain process, for instances, if you give it from the office to the community, you left a gap at the sub-district level, the staff who reported will not be aware that you have given the information and in that other way, you don’t have that link to the community, so it must pass through that channel to enter the community and then it would be the right way.

<Internals\\IDIs DHMT\\VRDI_02> - § 1 reference coded [7.79% Coverage]

Reference 1 - 7.79% Coverage

I: …received anything. Do you give feedback on the cases that are reported to you?

P: As I’ve said since they are minor ones we don’t usually…

I: So you just do it, when you get there they tell you about it. Yh!

P: I learnt there was some serious case, was it after measles that the person was fainting something … but I wasn’t around, that time I was not here. So when I came I heard that that happened and the person was treated and (inaudible)

<Internals\\IDIs DHMT\\VRDI_04> - § 1 reference coded [9.20% Coverage]

Reference 1 - 9.20% Coverage

I: Erhm do you also give feedback on the adverse event cases to those that report to you?

P: Yes, I even go with them to the… what do you call it? You know what happens is that we send the forms and it’s like the the the issue is managed locally…

I: mhmm…

P: so the feedback I have received it indicating that they have received it…

I: Okay!

P: That’s that aspect of it. But at our level here what we do here, we pay visit to the clients.

I: okay

P: We visit them…

I: …To make sure…

P: … to make sure, to assure them and and mostly why we make the follow up visit is that we we try to encourage them not to to to give up on the immunization schedule. Some might feel not to…

I: …Come back…

P: …coming back for the because of the experience they they they have. There was time I would have showed you the picture; I think my machine has some issue.

I: mhhmm

P: they are working, I would have shown you some of the pictures that we we we dealt with. So we followed up and the client was managed at the facility level and the the the parents are… the caretaker are always very happy when we come and errh and even our staff, they are also happy because at least we’ve have come to I mean what do you call it? Boost their morale level. So that they are not alone in managing tho… those conditions that they’ve reported to us.

<Internals\\IDIs DHMT\\VRDI_06> - § 1 reference coded [3.32% Coverage]

Reference 1 - 3.32% Coverage

I: …and then do you give feedback on the cases that are reported to the district?

P: yes! Yes! Yes! So when it’s received…then we send it errh feedback that if it was an abscess, it’s now better and whatever it is…

<Internals\\IDIs DHMT\\VRDI_07> - § 2 references coded [6.79% Coverage]

Reference 1 - 4.10% Coverage

I: Ok, ok. Do you usually get feedback from ahm the region?

P: Yes we do, erhm but not all time.

I: Ok.
P: Is not all the time,

I: Ok

P: is not all the time. I remember was it a year or two years ago, Avemectin distribution, there was one particular officer at the border, border post. I think he had something just like Stephen Johnson syndrome.

I: Ok.

P: But we made all the report and then went to …virtually we send… Accra requested for the pictures and we send all but I thought there would have been a feedback on what actually was happening in my…. But I don’t think I had any feedback.

Reference 2 - 2.69% Coverage

I: Ok. And from your point too, do you give feedback to the facilities as and when the report cases?

P: Yea, erhmm when you don’t know the cause, else you pick and forward. So you inform them about the processes you’ve gone so far and as you wait for the results from the. . . whatever is done to it in Accra. Then you give them the feedback for that particular client who had had that effect.

<Internals\\IDIs DHMT\\VRDI_08> - § 1 reference coded [5.46% Coverage]

Reference 1 - 5.46% Coverage

I: Ehmm, So again from your level here, do you usually also give feedback to the facilities that report cases?

P: I think normally at our review meeting, we still errh, sensitize them on this reporting and then we even revise the form also for them to be used in… so I think errh, since I’ve being here, apart from… I think for EPI, I don’t think we have reported but for… I think one of the program or so, we reported some… I’m sure my disease control officer mentioned those ones. But they’re not… It seems they are not specifically for EPI, but other program, programs.

<Internals\\IDIs DHMT\\VRDI_11> - § 1 reference coded [4.91% Coverage]

Reference 1 - 4.91% Coverage

I: Ok, because I’d wanted to further ask whether…, you know reporting is in, can be seen in two folds, either you are reporting a case or you are reporting a no case, zero reporting.

P: Zero reporting.

I: Yes. (*She laughs*). And so I’m sure for now….

P: Emmh, Ok based on the zero report, I think we are even prompted by region sometime

I: ok

P: that they are receiving such reports from us.

I: Ok, ok.

P: *(She laughs*).

I: So is there equally a trickling down effect to the facility also for not receiving, errh, I mean any case as in, for also receiving zero reporting from the facility?

P: Errh, I think one of our meetings we made this… we made mentioned of it to them.

<Internals\\IDIs DHMT\\VRDI_13> - § 1 reference coded [3.36% Coverage]

Reference 1 - 3.36% Coverage

I: Ok eere talking about feedbacks again do you give feedback to eere do you feedbacks AEFI cases those that report to you?

P: Yeah,

I: From?

P: Down facilities we do whatever report concerning AEFI we receive from the facility level the necessary action that we take before extending it to the next level we use the same route to get back to them.

<Internals\\IDIs DHMT\\VRDI_14> - § 1 reference coded [3.52% Coverage]

Reference 1 - 3.52% Coverage

I: So you don’t know whether they’ve been getting feedback or not from the regional level?

P: Yes please.

I: Oh ok and eehe do you give feedbacks to, feedback on AEFI cases to those who report to you maybe from the, the facilities do you give feedbacks to them?

P: Ohh if is, for me they don’t report until l meet it on the filed ahamm so all this things unless you ask disease control.

<Internals\\IDIs DHMT\\VRDI_15> - § 1 reference coded [6.47% Coverage]

Reference 1 - 6.47% Coverage

I: Thank you do you give feedback on AEFI cases to those who report to you?

P: Yes the few that we have we also give them because sometimes we involve the supervisors in managing the cases, the district level doesn’t only go to the sub eere the communities to manage we involve the facilities, in charges and the supervisors because eere what we do here is if we have anything like that we need to inform the carnations too so they are all aware.

<Internals\\IDIs FDA\\GAFDA_01> - § 1 reference coded [7.03% Coverage]

Reference 1 - 7.03% Coverage

I: Okay, ‘erhm’ please do you get, do you give feedbacks on AEFI cases to those who report to you?

P: Yes we do.

I: Please, why do u?

P: Why do we?

I: What’s the thinking behind?

P: ‘Erhm’ because once a reporter reports [inaudible] they would want to know the outcome of the result , so it’s for communication need to give feedback to have affective communication. So that’s why we give feedback to them to let them know the status of their reports and what happened , what kind of causality was accessed .

<Internals\\IDIs FDA\\GAFDA_02> - § 1 reference coded [4.83% Coverage]

Reference 1 - 4.83% Coverage

I: Okay. ‘erhm’ do you give feedback on AEFI cases to those that report to you?

P: Yes we do. We write back to the reporter through the EPI.

I: And why, why, what’s the rationale behind that?

P: Because the reports came from the EPI, The reports we receive, like I explained to you in a routine vaccination the reports are collected by the EPI and sent to the FDA. So we report back to the EPI and the EPI sends it back. But I some cases the reporter comes directly to the FDA, we provide feedback directly to the [inaudible] reporting.

<Internals\\IDIs FDA\\NRFDA_01> - § 2 references coded [10.55% Coverage]

Reference 1 - 5.69% Coverage

P: the will , we have to investigate, may be officers from our head office together with may be EPI people will come and investigate to make sure that you know there is a, we call a causal relationship between the vaccine and then the reaction.

I: ok

P: and then in the end, we there is a meeting with the health management team in the region, may be in the regional health directorate and then we brainstorm on the reaction that took place to make sure may be most reactions happens in different forms in different situations may be the client is supposed to take in may be food before taking, the client did not take in the food because of that that will happen. May be the public health nurse was supposed to be very careful you know the way she, he or she administer the medicine that cause it , so all these investigations are done then a conclusion is reached and then a communiqué or a report is brought back to the region for us to study so that such future occurrences will not happen.

Reference 2 - 4.86% Coverage

I: ok, ok. Thank you very much. We are almost done. Do you give feedback to those that report AEFIs to you?

P: yes of cause, we do. Especially if the very severe, very serious cases we do

I: why do you do that?

P: no we, we do that for them to know how those things happens such that in the future we will all be very careful such that we will able to prevent such occurrences. You know the idea of reporting about AEFIs is to make sure that in future we are able to you know advice manufacturers to what we call reformulation to make sure that we have a safe vaccine or quality vaccine on the market.

And the 2, we want to make sure that some of these reactions can also be prevented or adverted that is why we try to you know communicate these feedbacks to our stakeholders such that the will also be aware.

I: ok

P: about what is happening.

<Internals\\IDIs FDA\\UEFDA_01> - § 1 reference coded [0.66% Coverage]

Reference 1 - 0.66% Coverage

M: So, in effect you also give feedback to those who report adverse events to you

R: exactly
