# Supplementary material for: Optimising reporting of adverse events following immunisation by healthcare workers in Ghana: A qualitative study in four regions
Source: PLoS One. 2022 Dec 20;17(12):e0277197. doi: 10.1371/journal.pone.0277197 (PMC9767370; doi:10.1371/journal.pone.0277197)
Supplement: S1 Data — (ZIP) [file pone.0277197.s001.zip › Minimal data/S6 Why health care workers don't report AEFIs.docx]

**Name:** 12. Why health care workers don't report AEFIs

**Description:** This folder contains all information on why health workers do not report AEFIs

<Internals\\IDI EPI\\GAEPI_01> - § 1 reference coded [13.61% Coverage]

Reference 1 - 13.61% Coverage

I: and we will like to also know why health care workers themselves do not report the AEFIs when they encounter them

P: for some of them, if, some of them feel I mean reporting, cos most people think ‘oh’ if there’s AEFI then maybe if I report they might think ‘erh’ I caused the problem. So they might not want to report because they fear that their superiors might blame them for the event. Or they might not report because it was reported to them because if you don’t get the, the notification you will not, you will not report. So some of them are not reporting because the events are not coming out to them but others too may not report because, perhaps they think that if it’s reported it might be seen as if they are the cause of the, of the, of the, the, of the event. So those are the two because if I don’t get the notification obviously I, I, I can’t report anything and if I get too I might think that okay yeah, maybe if I report it might be also ‘arh’ I caused, I mean the problem. And also most people, some also always think as if one is AEFI, it’s necessarily because of the, due to the vaccine. So these are some of the things I mean ‘erh’ we are trying to educate the health workers about and those things.

<Internals\\IDI RHMT\\GARI_01> - § 1 reference coded [6.39% Coverage]

Reference 1 - 6.39% Coverage

I: so we would also like to know the reason health care workers themselves who encounter this AEFIs do not report them

P: ‘erh’ like I’m saying, they see it as normal. One, because they feel ‘erh’ it will go by itself. And at times too those involved do not want to come and report. The only thing they tell themselves is I won’t go in for the vaccine again. If I send my child and then this thing happens then I will not send my child again

I: this time it’s the health care workers

P: yeah! Yeah the health care workers, you know, I, I do not know whether psychologically or what some of them even will not tell you that this is what is going to…but before you administer you have to let the person know that. ‘erh’ the person can react, this, that, that. If for any reason, it is this kindly let us know. And some do, some also do not. I will not say all the health workers, we are, we are human. Some do, some also do not

<Internals\\IDI RHMT\\GARI_02> - § 1 reference coded [10.52% Coverage]

Reference 1 - 10.52% Coverage

I: so we would also want to what might be the reason why health care workers themselves are not reporting the AEFI cases.

P: yes. ‘Errr’ the health care workers themselves too, sometimes they too they, they think ooh it’s normal, because normally when we train them and we tell them that fever and those things, they say eih, then we will fill plenty forms. You know, ‘uhuh’! So they too they think it’s normal it’s not anything and of course, sometimes they send the reports, the forms and the feedback is not too encouraging. Most of the times they don’t even get any feedbacks so if it is just fever the woman should just take paracetamol and then that’s it and if it is swelling they think it will recede so they are okay. ‘Uhuh’! So, that is the main reason. Most of them think that it’s a waste of time. Unless it is very serious, normally it is the severe ones that they would want to report. If its paralysis, “err” swelling, convulsion or if there is rumour that somebody has died! Mostly it happens when we are doing campaigns and you hear ‘norr’ they say they took the injection and somebody has died so, then everybody becomes alarmed so the least thing then they will report. That is the reason why most of the ‘erhm’ nurses don’t report these AEF… [Interrupted by interviewer]

<Internals\\IDI RHMT\\NRRI_01> - § 1 reference coded [6.48% Coverage]

Reference 1 - 6.48% Coverage

I: Ok, thank you very much. What might be the reasons why health care workers who encounter Adverse Events Following Immunizations are not reporting?

P: “Hmm”, they are similar problems, they superior sometimes seated up there when there real implementers reports sometimes on AEFIs, it looks like they are not doing well. Is like they follow has not done the right thing that is why these things are occurring.

I: Ok.

P: So definitely when the fellow gets or even when the bring AEFI to him or her the tendency to report it is usually low because he or she thinks that if it is reported she ends up be scolded.

I: Ok ok.

P: So that is one of the reasons.

I: Ok, Do you have more to add?

P1: Is almost the same thing, because our staffs they think giving an immunization to a child and child coming up with an AEFI and you the mother coming to report to the staff means something, it means the staff, she doesn’t know how to give the immunization or she is, she has done something wrong that is why that thing has happened but as we all know we tell them that AEFI is not “arrr”, is not something for the child or the mother, it is something that is supposed to happen and if it happens they should come and report.

<Internals\\IDI RHMT\\NRRI_02> - § 1 reference coded [5.62% Coverage]

Reference 1 - 5.62% Coverage

I: What might be the reasons why healthcare workers who encounter AEFIs are not reporting?

P: They too, is the same, they they may think that, for instance if is an abscess they may be feeling guilty that they didn’t do the vaccination well and that is why the err the reaction or the adverse effects are “errrrrr” occurred and sometimes they themselves if they are not well educated like I said if you are not trained on AEFIs it means that you are naïve, you are ignorant so when there is ‘err’ a reaction you may not report “ahaa” and sometime for fear of blame.

I: Ok.

P: And sometimes too, they also face frustrations, she reports and then you have taken the especially in the severe forms where you have to move from facility to facility with this person and then because is free ‘errrr’ treatment and management people are not willing to support it and then you will get here , you are not given the necessary support and you are with this client you know it can also actually ‘errrrr’ dampen their spirit of reporting.

<Internals\\IDI RHMT\\UERI_02> - § 1 reference coded [6.20% Coverage]

Reference 1 - 6.20% Coverage

M: On the part of the health workers would you say they are the things or there are different reasons why health care workers will not report AEFI’s?

R: In that case, what I said was for the health workers. If it is for the care givers I think it is lack of education. We don’t tell them enough. We are supposed to tell them that if you go and this happens, come back and report to us. We are good at telling them that if you have cold you could give them paracetamol syrup or this or that, you could apply cold compress in case there is an upset, but beyond that we need to tell them to come to us so that we know we also need to tell them the reason why we are asking them to report. Because the fellow is not too sure, so he is likely not to report. My child is fine where will I go? So we need to let them understand that if anything happens, we need to send the report so that they study it to find out, may be they could make their vaccine more effective and friendly to the children so that they don’t get more of such events. So if we don’t tell them the reasons they should report they are likely not to report.

<Internals\\IDI RHMT\\VRRI_01> - § 1 reference coded [18.91% Coverage]

Reference 1 - 18.91% Coverage

I: ok, alright, thank you very much. Emmh, now let’s delve a little bit deeper as far as the reporting is concern. What do you think accounts for the low reporting that we, we. we are witnessing on adverse events?

P: Errh, for the low reporting, like I mentioned, one the staff at the facility level that carry out the vaccinations, errh, their confidence level is low.

I: ok

P: Because some have the feeling, may be if I report something they will feel that I’m not doing the right thing

I: ok

P: because they not trained on it.

I: ok

P: Because they are not trained on it. So I think errh there should be a proper training for all the people that do the vaccinations so that they would be able to report.

I: Ok.

P: Ahaan, because the system is there, the reporting from facility to district, errh sub district to district to region, national. The system is there but sometime how to monitor it because some, some, may be like the clear definition, what to report, some of them may also not be too sure on some of the things to report. So I feel errh a lack of tracing of the staff that actually do the vaccinations on AEFI is one of the reasons we having how reporting rate.

I: ok. Any other please?

P: Emmh and other ones too, is like maybe like the communication aspect.

I: ok

P: That to sensitize caregivers about the AEFIs because all you know, some of the caregivers too if there is AEFI all that they do is…., if is even severe, and they have not reported they feel ok; I have gone to the vaccination session and this happen next time I will not send my child. Because they also don’t know that may be this and this are the things I have to report on.

So the second thing I will think is errh, we should also errh think of sensitizing the caregivers.

I: ok.

P: Ahaan on the AEFI. When they know that, ooh these things can happen, these ones are expected, though they talk to them at the clinics but I don’t think those education is errh, very, very errh, enough or adequate,

I: ok

P: so there should be more or we should step up education on the AEFIs to caregivers.

I: ok. Emmh, does your unit

P: yes

I: have in place the mechanism to monitor AEFI cases in the region?

P: errh, yes, we have a mechanism but hern, with challenges.

I: ok.

P: nhmm, we have the mechanism with challenges.

I: What are they please?

P: Because every, every year, we put in plans for monitoring

I: ok

P: ahaan, but sometimes you, the times you would want to go for the monitoring, the funds are not available for you to go and do the monitoring. So I think that’s one of the challenges.

I: ok. Ammh, could that also be a factor, contributing factor to the low reporting from, from the facilities?

P: That we are not able to do monitoring?

I: yes

P: Errh, yes it’s also part of it.

I: Ok

P: Because if you visit the district that may be you have to get at least one or about 10, a district is supposed to get about 10 and you report only one or two, what are the challenges? If we also visit them and they also look at the sub-district, may be this sub-district you are suppose get at least three in the year. So what are their Challenges? I think the supportive supervision also plays a role.

<Internals\\IDI RHMT\\VRRI_02> - § 3 references coded [12.23% Coverage]

Reference 1 - 4.86% Coverage

I: ok. Thank you very much. Emmh, madam please what do you think could possibly be the reasons why health caregivers are not reporting AEFI to the facilities?

P: ammh, the main reason, may be is errh, they are just thinking we will not errh… they are thinking that maybe they aren’t doing something right at their level,

I: ok

P: though we’ve being telling that we need these things to be able to report to the appropriate quarters for interventions, they still feel erh, it will be fault finding erh, adventure or something, so they don’t to report on these things.

Reference 2 - 4.21% Coverage

I: ok. But the problem might be from the facility?

P: Yes, we filling the form because we don’t see the seriousness of these things.

I: Ok not seeing the seriousness could be part?

P: Yes.

I: and like you said earlier, it could be some kind of fault finding.

P: Ahaah!

I: Any other possible reasons why they may not be reporting?

P: Well, it… if they don’t, don’t have forms available at their level. Sometimes, the forms are kept at the district, they won’t send it to the facility,

Reference 3 - 3.16% Coverage

I: ok

P: it might also be a problem or the forms are there but they don’t orient them well

I: ok

P: on how to fill it.

I: Ok.

P: Ahaan! There is a need for somebody to facilitate this for especially the newly qualified ones.

I: ok.

P: They might not have any knowledge about these things so, it might also cause erh, they not filling or informing the next level.

<Internals\\IDIs DHMT\\GADI_01> - § 1 reference coded [7.09% Coverage]

Reference 1 - 7.09% Coverage

I: So what might be some of the reasons why the health care workers who encounter AEFI may not be reporting?

R: Some of the nurses will be like I have given injection for the past 20 years and nobody has complained so why are now complaining that one too hmmm and also they don’t know the importance of reporting AEFI, they just don’t know the importance so I think those are the two main reasons for not reporting.

<Internals\\IDIs DHMT\\GADI_02> - § 1 reference coded [5.42% Coverage]

Reference 1 - 5.42% Coverage

I: What might be reasons why the health care workers may not be reporting AEFI’s?

R: Maybe they are familiar with what is happening, they are all minor conditions so they think it’s something that the can work with and since there has not been any serious issues I can’t say much.

<Internals\\IDIs DHMT\\GADI_04> - § 1 reference coded [6.82% Coverage]

Reference 1 - 6.82% Coverage

I: So what might be some of the reasons why the health care workers who encounter AEFI may not be reporting?

R: The health workers we need to refresh them periodically, the CHN’s and public health nurses have so much work to do so periodic trainings and refresher and supervision will do and also availability of the forms so that every immunization session it will remind us of the need to report.

<Internals\\IDIs DHMT\\GADI_05> - § 1 reference coded [7.89% Coverage]

Reference 1 - 7.89% Coverage

I: So what might be some of the reasons the health care worker who encounter AEFI may not be reporting?

R: Some don’t see the need others too are like there is apathy in the system, after all what will I get out of it that I have to pick the form, write and all that , they just don’t care because most of them, the CHO’s know that when these events happen the have to report.

<Internals\\IDIs DHMT\\GADI_06> - § 2 references coded [14.94% Coverage]

Reference 1 - 7.60% Coverage

I: What may be the reason why the health workers who encounter AEFI may not be reporting?

R: Maybe because they know how to manage the events if there is fever they know what to do so they don’t have to report but if they have reported then documentation will be present and we know what we are doing and the issues that are coming so we remedies those issues.

I: Is there any other issues?

R: I think because they know the remedies, if the child is having fever I have to do this and if the child has rashes they know what to do or they know the rash could be due to the measles why do we have to report, do you get it we know that when you give a child measles vaccine, the child is likely to exhibit signs and symptoms of measles so when you see rashes it is normal, because you know the processes that is gone through when you see it you are not disturbed compared to someone who doesn’t know you become agitated but if you know it already you won’t be so worried when you see it.

Reference 2 - 7.34% Coverage

I: So with the forms do you send them from the district to the sub- district before they get to the facilities?

R: Yes we give them then they run copies, then now filling the form becomes a problem so when they identify the AEFI cases you have to go from the district to go and fill the form for them.

I: Why are they not filling the forms?

R: They feel they don’t have the capacity to do it but I think they can do it, they will tell you I don’t understand this place, I don’t know this so when there is a client you have to rush there and fil the form, all these cases that we get; measles, AFP and all that we have to go and fill the form. They see the form as complex, it’s just basic information to fill; child’s name, age and all that but they see it as tedious, you know you ladies ( both laughs) writing and filling forms for them it is difficult but we are building capacity in that aspect so we hoping that as times goes on it will be better.

<Internals\\IDIs DHMT\\GADI_07> - § 1 reference coded [4.72% Coverage]

Reference 1 - 4.72% Coverage

I: please having said that ‘erhm’ what might be the reason why health care workers themselves who encounter adverse event following immunization do not report or are not reporting?

P: on themselves?

I: yes they themselves

P: ‘erh well sometimes I think it’s because they do not see it as ‘erh’ necessary yes. So they do not report. And then sometimes people complain also that ‘erhh’ it was insignificant, insignificant

<Internals\\IDIs DHMT\\GADI_08> - § 1 reference coded [10.47% Coverage]

Reference 1 - 10.47% Coverage

I: Okay. We also like to know why is it that the healthcare workers who receive this AEFI, or encounter AEFI are also not reporting them.

P: Oh! most of our, ‘arrh’ for my place they normally report, they normally report because we went through it and director was keen about it because that will help us to know the drug safety…and if there is any condition to that, so director send ‘erh’ hit, hammered upon it very well so we decided to give them, print every, print and distributed some of the list to the people to [inaudible]

I: What am asking is in general not your facility.

P: In general not my facility, at times I don’t know maybe they don’t get the information at hand [phone vibrating in background] so people because of the work load they will say they don’t have forms, they don’t have because the forms at times they have to do photocopies on their own so at times they will say they don’t have forms, ‘erhn’ they don’t have this and that. We know they have so many excuses to [inaudible] so we the supervisor have to be on them to be checking all those things.

<Internals\\IDIs DHMT\\GADI_09> - § 1 reference coded [4.93% Coverage]

Reference 1 - 4.93% Coverage

I: also we want to know what might be the reasons why the health care workers themselves who encounter these AEFIs are not reporting them.

P: hmmm, I think initially they also thought it was of ‘erh’ this thing ‘erh’ ‘erh’ is it the signs that you should expect when you take immunization but ‘erh’ with trainings, I mean during the last ‘erh’ measles ‘erh’ immunization we were told that all those things should be reported. Any immunization that you give and there is an ‘erh’ effect you still have to report it and even with other drugs. All drugs that you give and the person comes with a complaint, you have to document it and then send it to the appropriate office. Yes!

<Internals\\IDIs DHMT\\GADI_10> - § 1 reference coded [10.49% Coverage]

Reference 1 - 10.49% Coverage

I: That’s the next question. We would like to know your suggestions to improving the reporting of AEFIs

P: Yes! So definitely the best thing is training. But I’ve come to realise that most of the times training doesn’t really solve the problem. You train but you make sure that you go down there too, to ensure that the person who is doing or reporting understands what he or she is doing. You see that’s the key if they come for training and they go down and they don’t really understand what they are doing, they would even report but they wouldn’t report [inaudible] so or they won’t even look for certain signs because you know sometimes you, you give ‘erhm’ there is feedback right?…(knock on the door). So sometimes you might give ‘erhm’, ‘erhm’, ‘erhm’ a vaccine then err there is feedback and if you don’t know that even with that feedback you might think that [inaudible] but it could also come from [inaudible] so if you don’t know the signs to look out for; one: you won’t even report and if you don’t know how to report you won’t be able to report and if it’s also not demanded of you, you also won’t report and even if yes it’s demanded of you but we don’t go down there to supervise and to provide what to ensure that the right things we would err they would also not report and maybe the last bit is, if we don’t also give the feedback, you see! Because if I’m always reporting it to you and I’m not hearing anything from you, the encouragement wouldn’t be there

<Internals\\IDIs DHMT\\GADI_11> - § 1 reference coded [5.08% Coverage]

Reference 1 - 5.08% Coverage

I: so now, we would also like to know what might be the reason why the health care workers, the nurses themselves who encounter these AEFIs are not reporting

P: ‘hmmm’ [inaudible] I don’t know, I can’t say much because I know they will, they, they, we orientate them. Any training that we do we chip in some of this things but they find it difficult to do it.

<Internals\\IDIs DHMT\\GADI_13> - § 1 reference coded [8.14% Coverage]

Reference 1 - 8.14% Coverage

I: We would also like to know what could also be the reason why the health care workers themselves who encounter the AEFI’s do not report them or are not reporting them?

P: yeah! I think…if you look at our non-reporting rate, the way we hardly have AEFI’s reports is because, I believe is not because we don’t have AEFI’s occurring but it’s because we are not reporting. Well and health care workers won’t report simply because they would assume that…you know? The paper work, health workers (giggled), I don’t know whether I should say this but we don’t like writing, we don’t like documentation, we don’t like writing report. So instead of writing a long report, someone will come and ask you again what happened, put a summary together. You just write zeros and send it up. Meanwhile, there are AEFI’s that have occurred. Even mothers have brought their children or people have come to complain to them, they will just manage it clinically and then forget about documentation and reporting.

<Internals\\IDIs DHMT\\GADI_14> - § 1 reference coded [10.04% Coverage]

Reference 1 - 10.04% Coverage

I: So having said that what might also be the reason why healthcare workers who encounter AEFIs are not reporting?

P: ‘Hmm!’… I will not say that ‘erhm’ am fully aware that they are not reporting because the AEFI when the fill the form [Door bangs in background] they submit to the disease control officer. So if he is saying he is not receiving, I don’t know if he has told you he is not receiving those forms. Has he said he’s not receiving those forms?

I: Not really.

P: ‘Uhuh’ so you are just assuming, so if you are assuming they are not reporting…’erhm’… I don’t know

I: Could it be staff issues, administrative issues, logistics?

P: The thing is that am not sure if they are not reporting, because they are supposed to re, re, report and the form is submitted to the disease control officer. So I, I am not hundred percent sure that they are not [inaudible] so I can’t say anything

<Internals\\IDIs DHMT\\GADI_15> - § 1 reference coded [9.78% Coverage]

Reference 1 - 9.78% Coverage

I: having said that, so what might be the reason why health care workers who encounter AEFIs are not reporting them?

P: yes, the other aspect too is that, for, they may be thinking that when they continue to report AEFI that means they are not giving the immunisation at maybe the right site or maybe there is something wrong with their approaching or maybe there some, some, they are introducing infection to the children . So these are some of the things which we need to have to clarify them, that, the fact that you report it rather helps to strengthen the system. It doesn’t mean that you are doing something which is wrong. So they need to report but their fear is that when they keep on reporting that, they, their understanding is that they may say that they are not doing the right thing that’s why the children or that’s why they are reporting a lot of AEFI.

<Internals\\IDIs DHMT\\NRDI_01> - § 1 reference coded [4.19% Coverage]

Reference 1 - 4.19% Coverage

I: So, so for you in eeh like with respect to the health care giver it such a person encounters an AEFI what would be the major hindrance that would prevent the person from reporting it to the next level.

P: I don’t think there will be any major hindrance that will prevent the person from --- I myself here have encountered people coming here to report and we actually supported them e yeeh so I don’t think that one there is a hindrance that we will make a health worker not to report

<Internals\\IDIs DHMT\\NRDI_02> - § 1 reference coded [11.66% Coverage]

Reference 1 - 11.66% Coverage

I: Sir, but in the event where eem eeh a parent reports an AEFI to a healthcare worker will there be any reasons why the health care worker may not report this case to the next level?

P: The next thing too that we have to look at eah I think that is probably that some form of systemic failure which we have to address it looks like all along our concentration. I’m not saying that ee hm our concentration on AEFI training have been to the people who give the immunization who are usually community health nurses and field technicians who are not clinicians ok, so when an AEFI occurs and they come the most likely place they will go to would be to the clinician who may not even recognize that it is an AEFI and therefore must report

I: Ook,

P: For that matter I think that eeh our trainings should also take into cognizance and concentrate much also on the clinician so that when a mother or a parent come with the child and talks about the fact that the child developed this after the child was vaccinated then that thing will come to mind and therefore investigations will start otherwise when come what they simply do is treat the child for whatever symptoms the child has come with without actually taking in to eeh cognizance that this thing is an AEFI and therefore should be reported.

<Internals\\IDIs DHMT\\NRDI_03> - § 1 reference coded [6.92% Coverage]

Reference 1 - 6.92% Coverage

I: Ok, so what might be some of the reasons that would account for a health care worker not to report an AEFI?

P: Normally, when you interact with them some of them say that they assume that when AEFI occur it means that the person who is giving the immunization did not do his work well assuming that the way he is supposed to, the angle at which he is supposed to give the vaccination he defaults and then give it the other way so normally their fear is that when you report like that they would think you don’t know the work so this is the main challenge. [Child crying] most of them that I interact with that’s what they say.

<Internals\\IDIs DHMT\\NRDI_04> - § 1 reference coded [21.53% Coverage]

Reference 1 - 21.53% Coverage

I: So what might be the reasons why health care workers who encounter adverse events following immunization are not reporting?

P: Yeah, you know nursing ….. nurses in general……. We don’t want a situation they will say you don’t know your job….. Errrh you this experience nurse see have immunize this child and this thing has happen, the child has (inaudible) abscess, oh no, no, no you no, you didn’t do well. So we, we don’t like that kind of errrh I mean what word what word should I use. We don’t want that kind of wa.a.a way people will say it. Oh you this experience nurse you have done this, so we don’t want somebody to say that oh you don’t know your job. So, sometimes they will even get it, see the abscess alright, see the errrh IE and C very well but try to manage it so that it won’t come out for people to know that it was nurse A or nurse B who made that particular mistake. So sometime we hidden those things just to, errr just to (inaudible) or just to maintain our name but errrh but this time we always made it known to the staff especially those of us at the management level, we tell them that they shouldn’t hide it is nothing, even doctor can ….. give injection and something can happen, Doctor can do operation which he has been doing everyday in, day out and something else can happen is the something we are also doing at the community, we did immunization everyday, but one child …. Can give you trouble. Probably the person was not position well, probably the errrh, after immunization the massaging was not done very well, after immunization the mother also went and apply certain things to the side can cause any some of the things, so they shouldn’t hide, they should just feel free and report that will not disqualify you not being a good nurse (inaudible)

I: So any other reason (inaudible)?

P: Yeah, I think … these are some of the things and you know, the nurses too is not one category at the facility, now we are so many, enrolled nurses are there, community health nurses are there field technicians are there, staff nurses are there I mean the general nurses so now that we are of category and you know we rival each other community nurses says outreach services are their work, errrh enroll nurses say we are clinical nurses, *Soo* and if you are a clinical nurse and you inject a child probably and the child has an abscess you don’t want the community health nurse to say you don’t know your job, just to bring some intimidation to the category, so the person should shield it up and it is a community health nurse, arrh you that is, that is what you have been train to do and you go and give immunization and still the child is having abscess, so you see that there will be some kind of errrh I mean shame in one way or the other on that category. So they don’t want to say, so sometimes because of the category of nursing that we have everybody want to protect the name of the category so sometime they (inaudible) very difficult to report issues that happens during service errrh delivery. [phone vibrating] sorry.

I: Any other reason that may prevent the health care workers from reporting?

P: Emmmm sometimes too …. If the tools are not there (inaudible) so if it is not with them sometimes they just look, especially those who are new, they have never seen the form, they don’t even know you are suppose to report this. So as at when they come and the form is not introduce to them, when they get it, they don’t even know you are suppose to carry it to the next level and if they are not also informed verbally ..... they will sit down .... So as at when they are new nurses, the the form should be given to them. Education on the reporting should be major thing to them and you should be *discussed* even if you are not write officially during workshops and meeting you have to discuss those ones so they will see it very important as at when it happens and they can quickly report, bu, some, unlike the, e ,e monthly reporting tools when you get to their their facilities if you ask them to bring all the reporting tools they can bring them out hat you have given to them and they are all in files, but if that one is not in the files and the person goes through and did not find any reporting for for errh any tool that is suppose to report this event to the next level the should think that is not necessary and and then sit down.

<Internals\\IDIs DHMT\\NRDI_05> - § 1 reference coded [9.75% Coverage]

Reference 1 - 9.75% Coverage

I : So what could be some of the reasons why some Health workers who encounter adverse events following immunization are not also reporting they don't also report them?

P : Emmmm you know emmmm in the administration of ehhh vaccines, it requires some techniques ....... Emmm they fail to report it because one ..... they think the system ...... is going to take them on ..... for such situations, for instance ..... if you administer may be say PENTA and there is a swollen or an abscess .... you noo the fear is that you didn’t do it well that is why but in every human effort no matter what you do ..... At least there will be some situations emmm it is required or it is bound to happen along the line. But the fear of reporting and probable they will be taken on they will not want to report and I think basically that is the reason why .... But in most cases too .... it is not .... that the forms ...... are not available ..... but let me just say that they fear that probably when they report they will be taken on for reporting abscesses or may be adverse events following immunization. Well we have encourage them to do.

I : Any other ..... reasons why they may not be reporting?

P : Emmmm (door knocking) (door opening ) ehhhh if they don't also get the information from the community they cannot also report (door opening) you know information flow is emmmm

I : May be they might have gotten the caregivers have reported but they on their part are failing to

P : Okay, I may also say that May be ehhhhh they may have challenges with filling the form ......... But if you have challenges in filling the form is just the matter of asking or filling it and then bringing it and they will help you do the right thing ...... I think one of the challenges is filling of the form ....... You know the form is such that ..... it needs lots of information. Ehhhhh I want to believe may is a contributing factor.

<Internals\\IDIs DHMT\\NRDI_06> - § 1 reference coded [9.64% Coverage]

Reference 1 - 9.64% Coverage

I : So what could be some of the reasons why some Health workers who encounter adverse events following immunization are not also reporting they don't also report them?

P : Emmmm you know emmmm in the administration of ehhh vaccines, it requires some techniques ....... Emmm they fail to report it because one ..... they think the system ...... is going to take them on ..... for such situations, for instance ..... if you administer may be say penter and there is a swollen or an abscess .... you noo the fear is that you didn’t do it well that is why but in every human effort no matter what you do ..... At least there will be some situations emmm it is required or it is bound to happen along the line. But the fear of reporting and probable they will be taken on they will not want to report and I think basically that is the reason why .... But in most cases too .... it is not .... that the forms ...... are not available ..... but let me just say that they fear that probably when they report they will be taken on for reporting abscesses or may be adverse events following immunization. Well we have encourage them to do.

I : Any other ..... reasons why they may not be reporting?

P : Emmmm (door knocking) (door opening ) ehhhh if they don't also get the information from the community they cannot also report (door opening) you know information flow is emmmm

I : May be they might have gotten the caregivers have reported but they on their part are failing to

P : Okay, I may also say that May be ehhhhh they may have challenges with filling the form ......... But if you have challenges in filling the form is just the matter of asking or filling it and then bringing it and they will help you do the right thing ...... I think one of the challenges is filling of the form ....... You know the form is such that ..... it needs lots of information. Ehhhhh I want to believe may is a contributing factor.

<Internals\\IDIs DHMT\\NRDI_07> - § 1 reference coded [12.94% Coverage]

Reference 1 - 12.94% Coverage

**I:** What might be the reason why Healthcare workers who encounter AEFIs are not reporting?

**P:** Yea, as I said we are reporting because of the possible reprimand. Err… they are not reporting because they know that some of the reports come and they are mild ones and they know that by the vaccine the condition the child is likely to have, I mean fever. So if you come and that is your report that your child has had fever maybe you were even already told that it is likely your child will have fever, so you get the thing. So if the person comes again, so invariably it means I have made the person aware that the vaccine condition would let the child have fever and it would be anything too bad. So when he come back then that reluctance is there but with the advent of this new treatment, we have been made to know that, irrespective of you have already told them of the possible fever and other this things if they come adapt, because of that you should use that opportunity to create a rap ore so that other vaccines that comes for you, they can accept and say that after all I told you that it is likely this child will have fever because of given this particular vaccine, so that is why it comes. However, it will go away, no problem. But you fill the form and assure the person to go because such things may not need any medication and the fever will go away.

**I:** No more about that one. Do you have any more reasons on that?

**P:** Oh the… the…

**I:** Health workers not reporting.

**P:** As for other reasons for not reporting too has been poor documentation in the sense that even the forms, the forms I mean were not there. Forms were there actually, so if the person even wants to report he doesn’t even know the medium through which to report or the forms are not available. We used to have multiple forms. You see that we FDA reporting forms, the generic one, so immediately a lot of them got confused as to which one to report and some of them too look cumbersome but you see initially because you will the case and then a report comes. You have and you are reporting for the surveillance, AEFI surveillance but when you are reporting on the investigation one so the person gets confuse. Then they gave one too that goes with an in depth reporting and so many things. If it was the surveillance then it is just, that it just that is just reporting the condition but when it comes with the investigative one which needs a technical to be on it, so people lump the two together for that matter, seeing that long thing that scares them and they won’t try to start it.

<Internals\\IDIs DHMT\\NRDI_08> - § 1 reference coded [14.69% Coverage]

Reference 1 - 14.69% Coverage

**I:** Ok. What might be the reason why health workers [faint voice at the background]

P: Yes, during outreach, the distance too. Distance of the health facility too, from their house to the facility.

**I:**  What might be the reason why health worker who encounter AEFIs are not reporting? (repeated question)

**P:** Hmm, some of them do not know where, first we used not to do this mass orientation, so some do not know where to report to, and some too feared that when they report they will say they have maybe injected the children wrongly or something. That a few why some of the health workers are not.

**I:** I want more of those.

**P:**  [laughs loudly] [phone rings] so they are not reporting: they don’t know where to report, where to go to and so don’t find it necessary. That is it. There are more but later. [Still laughing]

**I:**  oh!!

**P:** It is ok

**I:** That is what we want to know, so that we come out with the interventions. And you please, if you can add more to that.

**P:** More to some of the staff don’t know?

**I:** Yes, not reporting. They will encounter but will report

**P:** Some forget and some too the work load.as for now the work especially the community health nurses they are the grassroots and the work is too much for them, the forms at the end of the month when they come here with their report you will understand. The forms are too many and you have to analyze all before we input them into the names. So some turn to forget and others don’t know where to report it.

<Internals\\IDIs DHMT\\NRDI_09> - § 1 reference coded [7.90% Coverage]

Reference 1 - 7.90% Coverage

**I:** Thank you very much. To you, what might be the reasons and why healthcare who encounter AEFI are not reporting?

**P:**  Well this can be affected by a lot of things such as some staff don’t actually know anything but AEFI probably they have heard of it but they don’t when to report it and even how to report it. As well as some have not even seen the form, how it looks like and even how to report on it. That is one aspect of it. and then, another aspect some other staff think that when they report an AEFI, the officer they are reporting too may hold them responsible for what has happened to one who took immunization. And for that matter, render them been lacking technical know-how or being negligent to their work. So I think these are some of some the reasons why some staffs are not reporting on AEFI.

<Internals\\IDIs DHMT\\NRDI_10> - § 1 reference coded [16.54% Coverage]

Reference 1 - 16.54% Coverage

**I:** What might be the reason why health worker who encounter AEFIs are not reporting?

**P:** I don’t think healthcare workers don’t report; as I said, AEFI most often than not is well understood by Public Health Nurses and those practicing Public Health and I said we have to work hard, probably should trained those doing consultation at the District, sorry at the hospital level. With those at the Sub-District, they are well vest, so they don’t know that it is the Adverse Effect that is why they don’t report. Even under norms, they won’t go to the hospital, the forms are there. It take any drug, whether it is vaccine or any drug at all, even paracetamol you have to report, but our training is almost like all only the, Public Health people and leave the hospital that is why there are not reporting. If you go and you want, go and get time and go to one of the consulting room and ask the Medical Assistant, he will tell you that I don’t know anything about it. Go to the pharmacy and ask, they would tell you I don’t know anything about it. Yes, and at times too it is so, what do we call it, so frustrating that we have some anti-snake serum, what happens? We will pick a form and fill it and send it to the Food and Drugs Board, they won’t mind you, no follow up, nothing. So that could be some of the things. We have finished.

<Internals\\IDIs DHMT\\NRDI_11> - § 1 reference coded [7.71% Coverage]

Reference 1 - 7.71% Coverage

I: Ok, thank you very much Mummy. What might be the reasons why health care worker who encounter AEFIs are not reporting?

P: “Hmmm”, for the reasons “errmmm” with our interactions, sometimes they think that if the report you might take it as a negligence on their part so a few might try to hide it, let’s take the case abscess formation may be they will try to cover it up such that it wouldn’t be brought to your attention, they think they will be victimized but that is what I think

I: Can you give more examples? More more reasons to that.

P: More reasons?

I: More reasons why health care workers are not reporting, they are under reporting?

P: May be “ermm”, well may be in some (“phone ringing”) in some cases too there is the likelihood, I don’t want to speculate but actually but I think that because people feel they will be victimized and then may be we can’t talk for everyone but I want to belief that some are not also able to recognize that these are “err” adverse event.

<Internals\\IDIs DHMT\\NRDI_12> - § 1 reference coded [11.67% Coverage]

Reference 1 - 11.67% Coverage

I: So mummy, what might be the reasons why healthcare workers who encounter AEFIs are not reporting? We the health worker.

P: We the health worker we don’t report?

I: Why is it that we encounter but we don’t report AEFIs.

P: “hmmm” ok. We encounter and we don’t report, certain times … may be left on to me they don’t come, certain times when th single one that will come he feel reluctant … to to to fill the this thing , to fill the form you just say go, I will do it and that ends. “arrah” certain times too most of them they don’t know they have too especially the newly trained qualified once if they haven’t taken you through. I might not know that I need to fill a form for all you know I think the last time we were someone said she has never seenit. That she has never seen this form so they showed it. It could be that she doesn’t know there is something like that left alone to report. “anhaa” so it could be due to various reasons err reasons and I can remember someone was also saying that the the indicators in the this thing forms is too many, that the form is too cumbersome, madam just writing writing writing, I said you need to document it. Yes, my former station not here someone ever made a comment like that so I feel , so we have various reasons why but the know this things is ignorance, he doesn’t know this things. After all is normal, you go it will go this thing and be free “ahaa”. And some too, they feel that may be is not I mean err is not the severe type if you just say mild and fever you just say go and sponge and give paracetamol, they mother too goes to this I mean some turn to that because is not severe they wouldn’t “ahaaa” report because we know it gives err fever so when we inject, we tell them that when you go the child may get temperature is not every child. So if you get temperature you sponge and give paracetamol so if it doesn’t stop you come back. So when the mother go and sponge and child does not so there is no need for him to come back. You see, so there are a reasons so if he does come he wouldn’t also record it that is so.

<Internals\\IDIs DHMT\\NRDI_13> - § 1 reference coded [3.61% Coverage]

Reference 1 - 3.61% Coverage

I: What might be the reasons why health care workers who encounter AEFI are not reporting?

P: mhm they are not reporting base of maybe they don’t get the report may be they don’t get the report to if get the report and they report may be they don’t also have the forms to fill at their various places but some of them also report.

<Internals\\IDIs DHMT\\NRDI_14> - § 1 reference coded [4.04% Coverage]

Reference 1 - 4.04% Coverage

I: What might be the reasons why health workers who encountered AEFIs are not reporting?

P: sometimes we feel that we don’t want them to know that there a actually people react to those can of thing so sometime that is why or we feel when we report they will say my district has reported that or that or my sub-district has reported this and that.

<Internals\\IDIs DHMT\\NRDI_15> - § 1 reference coded [5.96% Coverage]

Reference 1 - 5.96% Coverage

I: What might be the reasons why health care workers who encountered AEFI are not reporting what might be?

P: that is why am saying most them feel most of the AEFI that we record are not live threaten so they don’t see the need to report they only expect that when some comes and he dying that is where you can report that AEFI.

<Internals\\IDIs DHMT\\UEDI_01> - § 1 reference coded [10.45% Coverage]

Reference 1 - 10.45% Coverage

I: ok so are there any other reasons why healthcare workers too might not report aside...

P: I think that is, basically that is the biggest reason why they don’t want to report because it is like, some of, a similar thing happened in one of our facilities.(ok). There was a nurse who has been working for more than five years, she was having more experience (ok) and she, during her period, she never experienced that but one came from school and she was taken through, you know even after the two years in community health nursing they still have to learn on the job before (yeah) they become perfect so she did an injection an about two weeks, the mother came, the thigh was swollen, very hard so the mother, the caregiver brought the child and reported to the old nurse, the one who has been there for long, and they, they had to sit that nurse down, teach her, I mean, take her through the process all over, they got to realize that she didn’t know how to inject(ok) but that case never got to the district level (ok), we didn’t hear of it until something like that happened at the sub district before we had those information and it was all because they feel like if the information goes out so much and other facility members hears of it, it will go like she is not perfect and all

I: ok

P: that is just the basic reason

<Internals\\IDIs DHMT\\UEDI_02> - § 1 reference coded [10.28% Coverage]

Reference 1 - 10.28% Coverage

I: So what might the reasons why caregivers do not report adverse events following immunizations to healthcare workers?

R: Uhmm just like I just said, they think that it’s expected, they think that they’re self-limiting so if they don’t do anything about it, it will go (Ok). Because if its fever just like most of the immunizations (Ok), eh illicit sometimes just paracetamol can handle the matter (Ok). And they just see it after giving birth to a child you don’t need to (yeah) to report it (Ok), but I think they should be reported. Yeah.

I: Could there be any other reasons why they don’t report aside their mental you know mentality about just giving para, the fever can be subsided? Could there be any other reason?

R: Yea. As for… causes there’re normally many factors. And then eh… they might there should also be a simplified reporting form (Ok). Because if you look at those of us working in this part of the region. You go to a CHPS center and the number of forms that the person has to fill as reports are so many (many). So probably we will probably get a simplified form that will enable them capture the information very easily (Ok). The other thing is that maybe people will always complain that there is workload (Ok), so that if we were able to get focal persons for each facility, I’m sure that could help (Ok), but that is not the case now (Ok). It’s left whosoever is there, they should report. But we need focal persons for each facility who will coordinate all these adverse reactions. I think that could help us.

<Internals\\IDIs DHMT\\UEDI_03> - § 1 reference coded [8.57% Coverage]

Reference 1 - 8.57% Coverage

I: So how about the healthcare workers? What might be the reasons that healthcare workers too may not report an adverse event?

P: Yeah. As for health workers, their reason is that, if it comes to vaccination (Umm), you know as I said (Umm), it’s not only one spect aspect (Uhmm). The vaccine is having, can cause it. Is that not it?

I: Yes.

P: And then… the eh the the immunization that the human being himself giving the vaccination may cause it (Uhmm). So you see that somebody now reporting, maybe the reported name is indicated… he will say that ah, so is it that I don’t know how to vaccinate? You see that some people don’t want to report. To me, that is the way I’m looking at it (Ok). Because if you’re, there is a problem they will say that you’re the one who vaccinated the person. You get it?

I: Yeah.

P: To me, that is what I’m looking at. As well as the drugs. But that is why at times it’s not that, but if they say you’re the one who vaccinated; that’s why I’m saying that it’s not only one cause (O yeah). It could have been that it’s from the vaccine preparation (Uhmm), or the drugs preparation (yeah) as I was saying. And coming to the the the vaccination aspect, it may be you don’t do the As and Bs of what? vaccination(18:26), the site of vaccination can also cause that; the quantity that even you pick can even cause that (Uhmm). You get what I’m saying? (Yeah). And the needle that you even push inside – maybe you picked it long out – wind is blowing dust can put on it; immediately you push it inside somebody, you’re introduce it inside for the person, something inside – it can cause anything. So those things are there. So you have to, that is why I’m saying it may not be you. You may not intentionally did it, but with your process of giving the injection or vaccination (Umm) may result that (Ok). And then because of that, people feel like they should not report; because if I report and they say eh I don’t know how to do the work; I don’t know how to do that (Umm). But if they report it, and you’re the one, you now try to sit up what you feel I couldn’t do (Uhmm) and that thing occurred, you try to sit up and do that (yeah), and then it should not occur.

<Internals\\IDIs DHMT\\UEDI_04> - § 1 reference coded [6.50% Coverage]

Reference 1 - 6.50% Coverage

I: alright, thank you. mmhm sir, what would you also say are some of the reasons why healthcare workers might not report some of these adverse events should they encounter them in their line of work?

P: mmhm, probably some new staff may not be aware of the reporting because we have a very fast flow of personnel in Ghana health service, so some are working they go to school, new staff come in for instance those who have come in in April have not yet received an orientation in the service, the whole of this year I think because those who came in April have not yet receive orientation I think, some came in august, some have entered in October and they have-not had the orientation so it’s just the academic knowledge but these practical things help in redirecting their attention on such reportage.

<Internals\\IDIs DHMT\\UEDI_05> - § 1 reference coded [13.06% Coverage]

Reference 1 - 13.06% Coverage

I: alright, thank you sir, so can you also please help us with what might be the reasons why healthcare workers who encounter adverse events do not report?

P: sometimes their, they think that if I report like I was talking about the proper information or maybe the training given to them, sometimes their afraid, some think that, ooh if I report people will think that I don’t know work, maybe I don’t normally inject well for all they know probably the site, the problem is coming from the drug or the vaccine, it could be that the child is allergic to that particular this thing, vaccine and so sometimes it’s all about the information that they are lacking so that they are afraid and sometimes when they report, maybe they report it to the DHA and or they pass through the channel from the sub-district to the DHA and there is no immediate feedback given to the staffs so when such staffs sees or happen to see something like that later on after reporting the first one they haven’t had any feedback from it, they turn to relax because they see no reason why I should report the earlier, first one no feedback was given, maybe the feedback will come in a way like maybe this is what you should have done and you did not do it, you should have reported it earlier and you did not report it early so I think these are some of the things or reasons why they are not able to submit.

I: has that being a concern for the DHA?

P: yes, it has because it has being long that we actually had the training or refresher training on this

I: and are there any plans on how to deal with this?

P: I think as a district, we are planning to see whether such a thing can happen next year (2018) or if possible but I think on our usual monitoring and on the job coaching those things are happening.

<Internals\\IDIs DHMT\\UEDI_06> - § 2 references coded [14.01% Coverage]

Reference 1 - 8.27% Coverage

I: okay so what might be some of the reasons why healthcare workers who encounter AEFI’s do not report?

P: knowledge is one, inadequate knowledge; they don't attach any seriousness to AEFI, that is one. Two, when the person comes and has no health insurance and the treat or they manage the person, they will have to pay, if the person, and we tell them that they shouldn’t charge anybody for anything, so if they shouldn’t charge anybody and the person come, they will document, for instance, this one I just opened to you, they have, the managed them, you see they manage the cases, now maybe you have to refund the money to them, we don’t or we don’t do that, so how then will the person report again next time that they see another case, definitely he will not like to do that, ahaaa, sometimes too they do they they they they accept the case, they accept the caregivers complain that yes this is AEFI, they will fill the form but because of distance from there to the DHA level, the district level for us to also forward it to region, they will like to keep the form there and will not even do anything with it then when it come to, I have submitted a lot of forms, me in particular fro 2012, I have being submitting AEFI forms, you don’t hear anything, I dealt directly with Food and Drugs Board when they were implementing ...(inaudible word) A and this Rhotarix and Pneumococcal vaccine back then, when you send the forms at least you should hear feedback that what you brought was maybe not AEFI or it was due to this or nothing, you understand that, ahaaa and that will make of alot of meaning but you don’t hear anything from them, (chuckles) at times they feel like ahhaaa there is no importance in reporting.

Reference 2 - 5.74% Coverage

I: so will there be any other thing?

P: yeah there will be a lot of things but just that I I, sometimes times too it’s possible that it can't reach here and we will not submit it to region...

I: what will be the reason?

P: same means, same means at times you have sample here, how will it get to region, only one car in the district, so when the car is going and you are not even in the known so the more the forms delay with you it becomes like “kai”-(chuckle), there is no need I report on it, ahaaa let me leave it like that, or we all come in and technology, technology you see if there is something like offline form that you are filling even over there when they give you, when they call you and give you the information, you can just open and fill it and easy way that they can have it there, maybe if that you are sending it through WhatsApp or through mail or something like that, it will be easy, but they want everything hard copy everything hard copy. When I fill hard copy and if region, my colleague is going to region and am here and its soft copy, I can send to him on WhatsApp and they all, can even forward it to you straight away, but it’s true, the hard copy is only part.

<Internals\\IDIs DHMT\\UEDI_07> - § 1 reference coded [10.43% Coverage]

Reference 1 - 10.43% Coverage

I: alright, thank you for that. mmhm, what would also be some of the reasons why healthcare staff who encounter some of these adverse events do not report.

P: for staff that are directly involved in vaccination, the one giving the vaccination I might think reporting will mean reporting myself. That’s what we think, mmhm because I strongly believe that adverse events following immunization are many, they are not as few as we are seeing now, it just that staff do not want to report them because they think that people might be thinking that they don’t know how to give the vaccination(inaudible voice but noise from the surrounding), if it’s at the clinical side and the person is not I mean the person who is not enrolled in immunization might that mmhm, this one probably doesn’t need to be reported, he doesn’t see the essence of reporting it as an adverse event and might just treat it and let the person go.

<Internals\\IDIs DHMT\\UEDI_08> - § 1 reference coded [4.92% Coverage]

Reference 1 - 4.92% Coverage

I: okay, thank you very much. what might be some of the reasons why healthcare workers who encounter adverse events do not report them?

P: (laughs) mmhm, with that, some do not want to be blamed, they will say maybe its due to their carelessness, they don’t pay attention to what they do, they don’t know anything, so they prefer to keep it to themselves rather than report it to above.

I: will there be other things why healthcare workers do not report?

P: some too if it is something they think they can manage they don’t see it necessary to report (voice herad from outside)

<Internals\\IDIs DHMT\\UEDI_09> - § 1 reference coded [11.40% Coverage]

Reference 1 - 11.40% Coverage

M: What reasons might prevent a health care worker who encounters an AEFI’s from reporting?

R: A health worker

M: Yes

R: For the health worker is just the same, sometimes they overlook it. Like I said earlier on, something like fever, the person will say I have fever and he say do A, B, C thinking that it will go, thinking that he is able to manage it and for that matter. There is no need reporting unless he has done everything and it is not improving, he will now report either than that once they are able to take care of it. There is no need to report it.

M: Are there any other reasons that could prevent a health worker from reporting an AEFI’s?

R: That one actually depends on the person seeing to be his fault like if you are the type that always gives immunization and the place is swelling and all those things. It means there are a lot of implications on your job. It means you are not doing it rightly. That is how come it is so and if it continuously to a point that the person will not be reporting again.

<Internals\\IDIs DHMT\\UEDI_10> - § 1 reference coded [7.42% Coverage]

Reference 1 - 7.42% Coverage

M: So what reasons might prevent a health care worker to report an AEFI?

R: I think they are afraid to report because they feel if they report you will embarrass them you would blame them for what they have done. Also, they are reluctant in reporting. Sometimes, also these days especially those who trained the health care workers have little knowledge on AEFI. These days they are very reluctant unlike those days. There are some people if you ask them the meaning of AEFI, they cannot tell you the meaning. Some of them don’t know the meaning of AEFI and are reluctant to report because they fell like you will embarrass them some don’t report because they don’t get feedback from the district level depending who is working at the district level because some people and even myself I submitted the reports and feedback has not come to me. So I fell reluctant to report next time there is any.

<Internals\\IDIs DHMT\\UEDI_11> - § 1 reference coded [6.61% Coverage]

Reference 1 - 6.61% Coverage

M: What reasons might prevent a health worker from reporting adverse events following immunization?

R: Some of the health workers also think they are just minor adverse events so there is no need reporting them. As I mentioned earlier there is no adverse event which is minor we might regard it as minor but it might be serious on the part of the client we are dealing with and then the number two is if they lack the necessary tools to collect the data at the facility level. If they look into the file and they don’t get they just brush off and it is not followed up later. Number three because we get increasing numbers of health care providers coming in some of them might not have benefitted from the training on AEFI’s. So might not see the necessity to report these adverse events following immunization to the next level.

<Internals\\IDIs DHMT\\UEDI_12> - § 1 reference coded [3.96% Coverage]

Reference 1 - 3.96% Coverage

I: thank you sir, and what might be the reasons why healthcare workers who encounter AEFI’s are not reporting them?

P: yes, it is also on the issue of the knowledge of reporting, others don’t know and don’t have the understanding that this thing is something that I need to eerrh eerrh bring a solution from but rather look for the treatment of that effects and when that goes, that is all but if people are made to know that it may be due to the drug or it may be due to the attitude of the health staff that it happens, it can be corrected, they will be able and interested to forward it for those who need it for action to be taken.

<Internals\\IDIs DHMT\\VRDI_02> - § 1 reference coded [9.84% Coverage]

Reference 1 - 9.84% Coverage

I: So then errm, means that we should be asking the question why health care providers are rather not reporting adverse events. What might be some of the reasons why health workers?...

P: Okay the health workers also not reporting… I feel, Mmm It’s only that they don’t get or they don’t errr get errr the errr reactions cause I interviewed some of them, most often we do interview them so (inaudible)… apart from the minor slight swelling at the sight, apart from that…

I: So basically because they are simple errr to manage

P: Simple… yhh! Manageable ones yeah!

<Internals\\IDIs DHMT\\VRDI_04> - § 1 reference coded [17.30% Coverage]

Reference 1 - 17.30% Coverage

I: Ok Errhm can you please give me some of the reasons why caregivers do not report adverse events to health workers?

P: Care giver as in the the the community level?

I: Yes, the parents of of the infants who come for the vaccination…

P: Yes…

I: Is it…

P… they they they will always report.

I: Okay!

P: I mean from the experience I’ve I’ve had when when the child … reacts beyond the acceptable this thing they will surely report, they will get back to the CHN trying to inform her that oh! This is what happened. What of help can you offer to to to to solve or resolve the condition? But where I had the challenge was like I stated earlier some of them were actually not reporting, the staffs.

I: Okay!

P: Because they feel like it’s an indictment on their part. I mean, I am a nurse when I… It will mean that no! I don’t know the work I’m doing. You understand…

I: Okay!

P: Yes, so I have to come and tell them that no, that it’s not the issue. I think this issue came up during the training. I told them that with adverse events following immunization, there are, I mean varied factors that could result in that, one could be that erhmm technological issues, from the manufacturer. The vaccine could have a problem that when the the the child is vaccinated, the child will react. We also have the individual, I mean errh system you know we are made of diff.. someone will react to certain things, we react to things differently as individuals. That’s the recipient aspect. And then the the the capapcity issue of the staffs. Because it looks like if the the the vaccine is supposed to be given subcutaneously and ended up giving it at the wrong, I mean route of administration, you end up, I mean sometimes too I think the the the one classical one that we investigated that we deal with the the the dosage aspect.

I: Okay...

P: …The staff gave the wrong dosage and the child reacted I mean seriously to it so we have to come in, speak to the mother…they they will always report but where the gap occurs is the this thing level. So in our community interaction too, any engagement we have with the community members, I mention it to them when the… their child they receive the vaccine and the child is reacting to it be it crying, swelling they should quickly inform the nurse and then errh… Fine they will do that like I said maybe the nurse coming to inform us… but anytime they see me too or any of the officers, they should also approach us so we will see how we will we will deal with it.

<Internals\\IDIs DHMT\\VRDI_05> - § 1 reference coded [5.10% Coverage]

Reference 1 - 5.10% Coverage

I: Ooh okay that’s fine, and eee what might be the reason why healthcare workers, healthcare workers who encounter such cases that is AEFI, Adverse Event Following Immunization.. would not report.

P: Well I will think that the person, the healthcare worker thinks that if I report is like I’ve not done my work well that’s the thinking I have because eeeh though you are aware that you are supposed to report but is like when you report then I have not given, do, I have not done my work well that’s why they don’t report, that is the reason I can give, I: the reason you can give P: yea

<Internals\\IDIs DHMT\\VRDI_06> - § 1 reference coded [14.45% Coverage]

Reference 1 - 14.45% Coverage

I: okay! errhmm what might be the reasons why you think care givers are not reporting adverse events to health care workers?

P: errhmm… basically, errhh… I think sometimes, the way it is discussed errhh for instance, if there’s an abscess and sometimes the discussion says maybe the technique for giving the… erhh… whatever…

I: …vaccine…

P: …the vaccine…may be at fault so some of them assume that they… if they report then it shows that I wasn’t good enough.

I: okay…

P: …with my technique

I: Okay…

P: other times, they think it’s a common fever so… (mtchew), it’s not an issue make errh… so much noise about about it so…sometimes…and they think… and that one too… and other times they do other things they errh they…by the time that…urhh so may forget because especially if it’s not as significant errh… an issue, like just a fever or errhh small abscess or something, you think you can manage at lower level. *Vehicle passing*

<Internals\\IDIs DHMT\\VRDI_07> - § 1 reference coded [14.39% Coverage]

Reference 1 - 14.39% Coverage

I: Ok. Alright, so now we should look at it now from the healthcare workers. What could possibly be some of the reasons why they may not be reporting adverse events?

P: Some of them especially may be the newer staff might not be well aware about the processes.

I: Ok.

P: Well of course maybe they were not given an orientation in that area, so it might be due to the training and the high attrition rate of staff. Now the new thing, everybody is going to school, so after three years you go to school so the experience hands are no more on the field doing the work. Most often they are new, where you always have to start from the scratch taking them through the processes.

I: Ok. Any other please?

P: Ooh…

I: From staff point.

P: From staff point?

I: Yes

R: Hmm! Another thing, it could be the… most of the problem we have is the means transport and being able to move to the community to do the investigation. It may happen at time you don’t even have pesewa to buy fuel or anything like that you need to go to the field. We don’t have motor bikes and all those things. They are contributing factors.

I: Ok.

P: If the means of transport were …some, most of the staff would, would want to follow-up on those but if it comes at a times and then there is no money in the confers immediately to buy fuel, then you realized that the enthusiasm might wean or something like that.

I: Ok. Could, could… you know sometimes procedures could also account for some of these conditions.

P: Yea.

I: Erhmm, how do you see staff reporting cases when probably they might be the factor or the reason behind some of these conditions that the client may experience?

R: Yea, and errh, I would not want you to know that I’m inefficient, I did something that was not efficient.

I: Yes.

P: You know so you might just cover it up. Is in the village, nobody will know and keep quiet about it.

I: Ok. So can it also be looked at from the point of logistics?

P: Yea, yea, yea, yea. We always like I’m talking about transportation, you may not always have the needed logistics at that particular time to do the work.

<Internals\\IDIs DHMT\\VRDI_08> - § 2 references coded [13.74% Coverage]

Reference 1 - 6.82% Coverage

I: Ehmm, what do you think could possibly be the reason why ammmh, we are experiencing this low reporting or zero reporting even from the level of caregivers? What could possibly be…

P: Well, my stake on this is that errh, it looks like errh we are not seeing very severe adverse events. They are minor ones, so our staff think that the minor ones are not necessary. They give their paracetamol or whatever that will…. It normally happen, they assured the people so they don’t think that it’ necessary to… even the slightest complain to fill a form and then alert us. They fill that is just a minor adverse event, so there was no need to report on them. I think that’s the main reason why we have the low reporting.

Reference 2 - 6.92% Coverage

I: Ok. Ehmm, again on the side of the health worker, ehm, don’t you think that…. Erhm, you know sometime, some of these conditions could also arise from procedure?

P: yah, yes.

I: So…

P: Not even procedure only,

I: yes

P: how the vaccines are kept,

I: yes.

P: How they are reconstituted, and so on.

I: Yes, so if you are having a case that is as a result of some, somebody’s inabilities and probably negligence and you have this very fellow alerted by the mother, that this is the situation. I think that will be a challenge for the fellow to forward the issue up.

P: Well you are right but, in this case it means… there hasn’t being any such instances that’s why they are not able to report.

I: Ok.

P: Yes.

<Internals\\IDIs DHMT\\VRDI_09> - § 1 reference coded [6.55% Coverage]

Reference 1 - 6.55% Coverage

I: ok, alright. Then again at the level of the healthcare workers, what could possibly be the reason why they are not reporting?

P: ok, ehrr, my idea concerning that is, at their level too, they are also aware that some of the things are expected. If we take ehrr, injection site pain, it is a normal thing so long as your skin is being pricked. Ahaan, you feel pain, ahaan, you may have some redness, you may have some redness and then sometimes the lump. And so with them too, it is possible they don’t see it as so much of an issue.

I: ok.

P: Aside that too, some may ehrr, be of the opinion that they would be blamed for that situation and so instead of reporting, it will be seen as not being efficient. They would rather not be reporting those minor, minor ones except it becomes ahaan, something severe then they, they will have to report.

I: ok. Any other reason why they may not report?

P: Mhmm, that, I think that is the main.

I: ok. Can it also be looked at from the point of staff? Ehm, in terms of capacity, in terms of numbers and work load?

P: For that I don’t, I don’t think so.

<Internals\\IDIs DHMT\\VRDI_10> - § 2 references coded [23.42% Coverage]

Reference 1 - 18.21% Coverage

I: ok, ok, alright, so emmh, what of… that is at the caregiver level. Now within the facility, what could possibly be the reasons why healthcare workers are not or may not be reporting adverse event as and when they, they encounter them?

P: As I said earlier on that errh, for facility level, they do home visits and we are expecting all of them that, when they, visit a home and a mother reports to them, they must also take from there that is adverse event, so they must also create awareness to our level for us to also make errh, a fellow up. So if the facility level, they are not reporting, it implies that through their home visits activities, they are not getting the cases. Situation whereby a mother report to a, a, a care, a health staff and the officer refuse to tell us, so it means the officer is not doing his or her job. But here is the case they are not even getting it. So there is no way there should be a case at the facility level.

I: Ok, couldn’t, couldn’t it also be that, emmh, they see some of these cases as miner cases and so don’t really see the need to, to report those cases to the next level while they can handle that within their facility?

P: Errh, the fact is, we need to understand what we mean by adverse evec, event, what mean by adverse

I: event

P: event. So it means if you really understand the concept, there is no way that you would even say that is a minor issue

I: ok

P: or minor problem which you can easily handle it. It means there is a case and are refusing to. . . And which way are you going to handle it at that moment. The only way you can handle it is to report and we also our follow-up and see whether is the vaccine that is causing it so that, that vaccine would be taken out of the system. So we explain all these to them and I also believe that such a case or cases are not reporting to the facility for them also to prompt our attention.

I: ok, ok, so you quiet sure that our staff out there are, are, are actually not receiving those information that is why we are not getting them reported.

P: Yea, because we do our monitoring, we also errh, receive their report and we must be expecting such a condition should also be report to our office and all the meetings that we do have, we always keep on saying this. So why is not… are they not reporting? So it means they are not getting the cases for them to report.

I: Ok, (*clears throat*), so emmh, again moving a little from away to the reporting system. Is it always the case that they have forms for reporting always available, since where you have lack of forms at a moment to report, that could also account for some people missing out on, on reporting cases?

P: Actually, they… we always make the forms available. They come in, we do a lot of copies here and they will be sent to them. They come for a monthly EPI submission and when they come, we give the forms out. Some even take extra, some do submit without taking any form. So there, there is no way that we may have a shortage of form which they can also blame our region that, they don’t have a form that’s why errh such a case are not reporting to….

Reference 2 - 5.21% Coverage

I: ok, ok, alright, amh, don’t you also think that some staff, ammh, out of fear, you know sometimes some of the conditions may arise as a result of ammh, wrong administration. Ok? And for fear of, I mean fear as in from…, of blame and all those stuff from superiors. That could also account for the no, the no reporting that we are witnessing?

P: Actually for individual differences, it could happen that some people within this, they may hide it but to me, with all the knowledge gain, can you do it? With all the knowledge gain that when such a case occur let us know and we also go through our monitoring, we’ve not even come across any of this. But as I said, individual differences, some maybe thinking that when they report and it means you are not up and during in quote. It could be in the heart of some people but to me, that shouldn’t be the case.

I: ok

P: That shouldn’t be the case.

<Internals\\IDIs DHMT\\VRDI_11> - § 1 reference coded [14.50% Coverage]

Reference 1 - 14.50% Coverage

I: Ok, ok. Now at the level of the health care worker, what could possibly by the reason why some are not reporting adverse events even though they encounter them?

P: Well is, is because once the mother could not come back to report. Because errh the report will be after immunization when the child is pricked and there is pain, the child will just cry and is normal. But when they got home and the pain continue. That’s where they have to come back ,mhm, to report. And may be the education we give them that when there pain, this is what you should do and they also thing they have instructed me to do this when there is pain. So they may not be the need for them to come and then report to us, there is pain.

I: Ok. Could they possibly be other reason why healthcare workers don’t report adverse events?

P: This what I’m saying that, they don’t consider it serious and then also *(coughs)*. May be when they have heavy clinics, when they have clinics they could hardly stop to….. errh there things are not immediate erh, this thing that they observation of…, what they should report immediately. As I said, so that could be the reason. And also if the form is not with them, even where there is a need for them to report, they will not be able to report.

I: Ok. Emmh, emmh, don’t you also think that…., you know some time; some of these cases also result from procedure,

P: mmh

I: ok, in administrating the immunization. It could also results in some of these conditions, so in the event where you have such case, emmh, don’t you think that sometime because of the kind of response they may get from superiors or supervisors, ok, could also be the reason why they may not report such incidence?

P: It is possible.

I: Ok.

P: It impossible.

I: Ok

P: Sometime they may not want to because errh, you the superior officer will query them not in written but explaining to them that errh this thing has resulted because of this and that. Then maybe they want to shy away from that. (Not clear sound 16m: 37 s)

<Internals\\IDIs DHMT\\VRDI_12> - § 1 reference coded [18.02% Coverage]

Reference 1 - 18.02% Coverage

I: Ok. Emmh, please what do you think accounts for the low reporting from the facility?

P: One thing is, if you have not been trained, you would not be able to report. And if you are trained and you have not being errh, would I say motivate, like pushing them to do whatever they are supposed to do, they will not do it. And if they find it not necessary, maybe as if is one of those things like the pain, the swelling, the routine something that they being seeing so they would not attach importance to that thing, so they will not report.

I: Ok. Any other reason why some staff may not report an AEFI case?

I: Those are the few things I can…

I: You know some of the problems could be related to procedure,

P: mmh,

I: ok, where a nurse administers a vaccine the wrong way.

P: Annh, ok the pro, programmatic errors that we normally have. So the person will not be able to.

I: couldn’t that also account?

P: yea, yea, is, is true that if the fault is from me, I wouldn’t report on it,even voice it out for me to come and write it.so that will be part. If instead of mixing a vaccine with a diluent, particular diluent and you have mistakenly taken errh, another diluent to mix. That particular, it can occur, it can occur or even during the process of, I mean errh, the injections, maybe instead of errh, subcutaneous, you give it IM. It can also cause, so definitely the person will not come out

I: ok. Can the causes also be looked at from the point of administrative?

P: Causes of the AEFI?

I: No, the causes for… as in the non-reporting or the, the low reporting of, of condition from staff in the facilities. Can it also be looked at from the point of administrative, in terms of monitoring and those things?

P: Noo, Yes, that’s what I have said that if you’ve been pushed, so like “pushed”, like if you are not going there to monitor whatever they are doing especially at the static or outreach clinic where they are giving the immunization. If you don’t go there, they will do anything and…. It can also be a cause

<Internals\\IDIs DHMT\\VRDI_13> - § 1 reference coded [6.31% Coverage]

Reference 1 - 6.31% Coverage

I: Thank you very much sir eeere [bleating] what might be reason [bleating] why healthcare workers themselves who encounter AEFI are not reporting it [bleating] eeere?

P: That one for us not reporting it if it is within our management level if it manageable within our our our eeeh domain we try to do that one but reporting it to the next level you know it has a lot of process where u need to notify this notify this so if you can manage it you manage it the issue happen when you are unable to manage then you report to the next level for health workers or the district staffs getting AEFI and not reporting it to next level that one then not krachi east here

<Internals\\IDIs DHMT\\VRDI_14> - § 1 reference coded [8.84% Coverage]

Reference 1 - 8.84% Coverage

I: Ok, ok ok what might be reason why healthcare workers who encounter AEFIs are not reporting it?
P: In my personal view I think because the thing is not too serious and too serious that maybe the child will die or might have died and even if the child die kraa they don’t see as an adverse effect [loud noise in background] and also when the child is worm they think is normal thing because they know the immunization will let the child run temperature so they think is one so I think they are also lacking a little knowledge there that even when the child is worm to touch they have tell the caregivers that this the child will run temperature and therefore they should report, here the place will be worm they should report, and penta and nimococa eeii penta and nimococa the child may have some hard area, the place will become hard a little they should report so I think they are not telling the mothers or they the people don’t having the fare knowledge ahhaa about it.

<Internals\\IDIs DHMT\\VRDI_15> - § 1 reference coded [5.56% Coverage]

Reference 1 - 5.56% Coverage

I: Ok what might be the reason why care eehe healthcare workers who enchanter AEFI are not reporting it?

P: That one l, l don’t believe we, we enchanter it and don’t report because anything that comes that we is reported to the district level if their report, because we always ask them don’t you have any AEFIs to report they will say no, it is part of one of the reporting formats.

<Internals\\IDIs FDA\\GAFDA_01> - § 1 reference coded [5.18% Coverage]

Reference 1 - 5.18% Coverage

I: And then the healthcare workers themselves if they receive it the reporting is a problem please ‘erhm’ do you have an idea?

P: No I don’t.

I: Why the, the, the, healthcare workers

P: [inaudible] why they don’t report

I: Yes why the healthcare workers do not report themselves as they should?

P: Am sure that’s why you are doing this study so you’ll find out and tell us

<Internals\\IDIs FDA\\GAFDA_02> - § 1 reference coded [6.20% Coverage]

Reference 1 - 6.20% Coverage

I: Okay, and then the health care workers themselves, we understand that they are not reporting as they should ‘erh’, can you think of any reason why the health care workers are not reporting s they should…after receiving

P: What we, what we, we come across is that in most cases they do not report because they think ‘oh’ we have seen this before; for instance if it’s fever after ‘erh’ BCG or lymphangitis, they say ‘oh’ we have seen this before we are not going to report this. I think that’s the main reason why they don’t report the AEFI. And secondly, some will tell you ‘oh’ we are not aware, we don’t know that we are supposed to report etc, etc, so these are the two reasons why I think…

<Internals\\IDIs FDA\\NRFDA_01> - § 1 reference coded [4.47% Coverage]

Reference 1 - 4.47% Coverage

I: ok, so what might be the reasons why healthcare workers who encounter AEFIs are not reporting?

P: as I said, I think this question has been repeated, as I said, some of them think is, some of the reactions are nor normal because there was a a program and then some of the nurses said, unless the reaction is very serious like collapse or something like that , that is where

I: ok

P: the want to report but we tell them that no, whether is palpitation, is tremor, is even you know running nose, vomiting, dry mouth, whatever, you have to report because we want to know holistically all the adverse events you know associated with the vaccines so that in future we will be able to know some of this reactions which are unpleasant which are associated with the vaccines

I: ok

<Internals\\IDIs FDA\\UEFDA_01> - § 1 reference coded [11.54% Coverage]

Reference 1 - 11.54% Coverage

M: On the part of the health worker what reasons might prevent a health worker who encounters an adverse event following Immunization not to report it?

R: I would say over the years there have been series of training given to a lot of health workers and we expect that they should be able to report all adverse events following Immunization or dispensation of drugs in their duty. However, their reporting is so low or appalling so we want to attribute it to motivation because some of them that they are not being motivated and have to report these things. They don’t take it as their core duty or mandate to report these adverse events to us even though we have institutional contact persons which we have trained to report all these things to be able to pick these things adverse events. They have the forms to complete and forward them to us but we think that they are not being motivated enough so we follow up to a facility, do you have any adverse events that you have complete this form, they haven’t but I think every day there are these things that are happening but they don’t report. So the under reporting for us they should be motivation and package put in place to motivate the health professionals to motivate them otherwise it should be put as a mandate so that their reporting system any time they are reporting to their authority they should include that yes we encountered these number of events over the years and when they see that they would have to look out for these things. Now you would realized that it is not a mandate or enshrine in their day to day activities that is one of the reasons I think.

<Internals\\IDIs FDA\\VAFDA_01> - § 1 reference coded [8.97% Coverage]

Reference 1 - 8.97% Coverage

I: Ok, alright thank you very much. Now to the core issue, the low reporting of, of AEFI; adverse event following immunization. What do you think could possibly the reasons why we have the low reporting? What could account for that?

P: I believe we looked at AEFI as a campaign sort of thing.

I: ok

P: We have not descended further to the routine level.

I: ok

P: Emmh, anti-tetanus is it a vaccine?

I: Yes

P: yes but how do we consider it? As a normal injection or a vaccine? As a routine. We’ve not gone to the level of availing to the routine guide. They are different from the immunization thing.

I; yes

P: As to whether we have availed forms to that level that specifically this is the reason why I don’t believe we have gotten to that. I’ve, I’ve follow-up on this thing sometime and I think procedurally or structurally, we should look at this before we even come to the motivation.

I: ok

P: yea

I: Any other reason you think…

P: The other, other reason is that, still people haven’t gotten to level terms that your reporting does not actually lead to incrimination, where you are liable to punishment or something and that when you do it, is supposed to help the system. It supposed to help re-designing training material, it supposed to help re-design education material. People haven’t gotten to those terms yet because it’s believe that when you do it either you will be castigated, either you will be dented, either you will be seen as a conduit for creating problems for the district or some sort of hierarchy.

I: yah

P: In my region we, we had it that all reports are to go to the office of the regional director of health

I: ok

P: the regional of Ghana Health Service, before they come to FDA for onward transmission to erh, erh Accra. Now it cause the delay, it cause even mix, mix erh, erh, mix-up. It cause the mix-up. Emmh, I can show you a form that came with a different medication as the sample attached to the form and when you looked at the form, now the form is gone, they are to bring the samples and they brought samples that do not have any inclination to the form that was submitted. But when we were going there to collect, when there is any problem the ICP is able to relay it direct. Now I don’t know whether it is felt that the ICP might be reporting on somebody’s non-availability

I: yah

P: to be at work or whatever or something, so they are not… And you know there are hierarchies, and errh, Ghana health service is not FDA,

I: yes

P: so something to come out of there to FDA, the headship must know.

I: yes

P: Structurally, we’ve not looked at this, so even though…. I’m talking about this because I made a… there was a form of scholarship forte ICPs too. So we were supposed to choose and then inform our head office and then they give the scholarship to who we feel. So we looked at the ICP that is mostly reporting. Frequency,

I: ok

P: we realized one gentleman from…. For the sake of whatever let me keep the details. So chose this person to enjoy the scholarship but Ghana Health Service had a different idea, a different opinion. Yea, you see, how can we make a choice within their structure?

<Internals\\IDIs PROVIDERS\\GAPI_01> - § 1 reference coded [4.83% Coverage]

Reference 1 - 4.83% Coverage

I: So what do you think might the reasons the health workers may not be reporting?

R: Forgetfulness, I know that they know they are supposed to report but because nobody is checking, nobody is looking, nobody is asking for the data, like every month somebody is asking if let’s say Ussher is looking for that data then you can report.

<Internals\\IDIs PROVIDERS\\GAPI_02> - § 1 reference coded [10.78% Coverage]

Reference 1 - 10.78% Coverage

I: What might some of the reasons why healthcare workers who encounter AEFI are not reporting?

R: It may be due to it is rare but I don’t know why some workers won’t report.

I: In terms of Logistics and administrative cost?

R: In terms of logistics everything is available, the forms are there, and the vaccines are there.

R: What is the question again?

I: What might some of the reasons why healthcare workers who encounter AEFI are not reporting?

R: Ignorance, that is what I said earlier on that they have to know, that’s the training they had in school and when they come out too they have to go through orientation so what can I say, and poor documentation, they overlook oh as for this one it will go apply this and will not document and proper supervision may help in terms of the direct in-charges.

<Internals\\IDIs PROVIDERS\\GAPI_03> - § 1 reference coded [7.16% Coverage]

Reference 1 - 7.16% Coverage

I: What might be some of the reasons some health workers may not be reporting AEFI’s?

R: There are no issues but for instance if a mother comes for vaccinations and says last month when I came last month, my child had fever but she is ok, its past and the issue has been resolved so they might not see the reason why they have to report but if the mother comes back immediate to report then am sure they will take it over.

<Internals\\IDIs PROVIDERS\\GAPI_04> - § 1 reference coded [7.52% Coverage]

Reference 1 - 7.52% Coverage

I: So what might be the reason why health workers who encounter AEFI may not report?

R: At times they are afraid, I have caused this so we have to educate them it might not be from them at times its manufacturing error so they have to report so we can do investigation into it so we can tell where the problem is coming from because everybody has his or her own allergy so we don’t blame the nurse who is giving that injection. Otherwise they would not report it so they have to talk to them that it is part of the immunization activity so they counter that fear and feel free to report it so that it is investigated into.

<Internals\\IDIs PROVIDERS\\GAPI_05> - § 1 reference coded [10.63% Coverage]

Reference 1 - 10.63% Coverage

I: So what might be the reasons why health workers who encounter AEFI may not be reporting?

R: One because of the numerous works on them so the nurse may think this is not serious and not report it and two no incentive and three can be transportation, the means as in financial, the person goes on outreach, they have to bring the mother and the child for consultation and the person has to use her own money and transport herself and the child to the facility and when the person is going nothing is given to the person do you think if it you, you will do it your little 10 cedi on you , so those are some of the reasons and most of them to don’t have time, after going for outreach they want to go home and do their own stuffs so when incentives for transportation is given to be able to transport or carry out and bring the mothers to the facility and give them more education that rise in body temperature is a serious AEFI or an AEFI that has to be reported I think it will improve upon everything.

<Internals\\IDIs PROVIDERS\\GAPI_06> - § 1 reference coded [23.64% Coverage]

Reference 1 - 23.64% Coverage

I: Okay, so what might also be the reason why ‘erh’, ‘erh’ healthcare workers who will encounter these AEFIs will not report them?

P: They themselves?

I: Yes

P: Sometimes they see it as minor they don’t need to and then sometime too ‘erh’ for a example if am a health worker and then I take immunization and there is swelling I carry it to the next level everybody will think that ‘oh’ this person is ignorant he is useless, so I have to give the treatment myself and then…

I: Could it also be like maybe administrative issues, could it be administrative issues that’s why they don’t report?

P: Hmmmm

I: Or is it staff issues or logistics?

P: Hmm not necessary it becomes personal, it’s becomes personal not necessary administrative or logistics, no.

I: So, so we also want to know how we can improve the overall process of reporting Adverse Events Following Immunization

P: Hmmmm

I: Already you’ve said a lot but if you can add some more.

P: ‘Erhnnn’ some more hmmm, you know lack of knowledge per say ‘nu’ kills a lot ‘erhn’ because the person is going to pass through ‘erh’ this procedure and he doesn’t even know what will happen to him afterwards. Because most of the immunization will get to you without any literature you don’t have any knowledge about it. So if you have access to all things, literature about it, you read little about what you are going to take so you know the side effects and other things so anytime you face anything you know that no! This one is related to this. But if you are ignorant you are only there an then the person comes and then give it to you, you don’t know what you are expecting to receive after you have taken the immunization. So when you educate, when you are educated on it … for example we are going to give CSM, oh, you are only told there’s outbreak here so come and let’s give you the CSM. Hardly will somebody even ask what should I expect when I take the immunization [inaudible] but if you are enlightened about it then you are very cautious or you sit up and expect any light change in you r system. So knowledge, when have knowledge about it will enhance the reportage.

I: Wow that’s, that’s very true, okay this brings us to the end of our interview

<Internals\\IDIs PROVIDERS\\GAPI_07> - § 1 reference coded [8.88% Coverage]

Reference 1 - 8.88% Coverage

I: what may also be the reason why the healthcare worker who encounter these AEFIs are not reporting?

P: the healthcare, like the health worker

I: yes.

P: the health worker?

I: who encounter this adverse events will not report…is, it, could it be administrative issues, or staff issues or logistics

P: [7 seconds] okay, if they are not reporting then they don’t have the logistics to report.

I: hmm, is that all?

P: administrative [inaudible] the logistics will come from administration, so if they don’t have the logistic what should they do…sometimes you don’t have money to print forms [inaudible] I don’t think it’s the caregivers’ problem

<Internals\\IDIs PROVIDERS\\GAPI_08> - § 1 reference coded [7.77% Coverage]

Reference 1 - 7.77% Coverage

I: Okay so having said that then, what might be the reason why caregivers do not report AEFI to healthcare workers?

P: I think most of the time it has to do with fear of being reprimanded like somebody who has given immunization and later on ‘erhm’, them, they reported with swollen ‘erh’ leg at the site or there is a sore or something.’ Erhm’ how come I feel that if she report it they might reprimand or they might take some ‘eih’ ‘erh’ action that might go against her, them so they have this fear that reporting it will, will cause ‘erhm’ bring them problems, they will bring them so they normally try to cover it up.

<Internals\\IDIs PROVIDERS\\GAPI_09> - § 2 references coded [11.58% Coverage]

Reference 1 - 8.02% Coverage

I: so if that’s the case, then what might be the reason why the care givers ‘erhm’ do not come to report AEFIs to the health care workers

P: I can’t really tell. Sometimes I will say; illiteracy is part, others too don’t really have time to listen to what you are saying. Sometimes you can give a health talk, at the end of the day you ask and nobody will be able to give you the exact what you’ve said. Or even just ‘erh, ‘erh’, ‘erh’ half of what you said, they can’t even, they can’t even tell you. So sometimes you know, you can’t really tell or may be what we explained to them they couldn’t get it well ‘eheh’ or even the process that we even gave, maybe they did not even understand. So it’s both side

Reference 2 - 3.55% Coverage

I: we will also like to know some of the reasons why health care workers themselves who encounter these AEFIs are not reporting them?

P: maybe they don’t even know the procedure they are supposed to be using, maybe they don’t know the procedure. Or they don’t even know the importance of reporting the AEFIs ‘mhh’

<Internals\\IDIs PROVIDERS\\NRPI_01> - § 1 reference coded [18.00% Coverage]

Reference 1 - 18.00% Coverage

I: So what might be the reason why health care workers who encounter AEFI are not reporting?

P: Some do not have a fair idea about it and if it is not somebody who is a directly involve with immunization or EPI activities might not know that it is an AEFI

I: So is it about administrative issue that could be accounting to this?

P: Come again

I: could it be administrative issues that account for the health workers not reporting or caregivers not reporting cases, AEFI?

P: About caregivers not reporting some of them might report to like in our case in our health cnter if you are sick you go through OPD. So when you go into the consulting room and the person consulting doesn’t have any idea about AEFI the person, the caregiver will only treat the condition as to whatever will be involve but will not have that idea of may be let me report or tell the person involve on this is AEFI so you can report to the next level.

I: Does logistics also account for this not report the issue?

P: yes, yes because for now I only know that this a form for AEFI that if you happen to meet or get a client like that you report you fill and submit to the district level but I have seen it and certain times too when you even report too when you even report because of lack of feedback you don’t see the need or you don’t know whether it was actually receive or recorded as an AEFI

<Internals\\IDIs PROVIDERS\\NRPI_02> - § 1 reference coded [7.48% Coverage]

Reference 1 - 7.48% Coverage

I: Ok so, would there be any reasons why, I want to know would there be any reasons why a health care giver like a nurse would not be interesting in reporting an AEFI to the next level?

P: Is, is not, there wouldn’t be but because most of us are not aware that such cases supposed to be referred out but in eeh lack of knowledge on it too because may be you take the form filling it! Processing it! So this are some of the basic things that always hinder us from en eeh! Reporting them.

I: So, so for you what would be the main reason that would account for this aside what you have just said?

P: This are some of the reasons I think always hmm

<Internals\\IDIs PROVIDERS\\NRPI_03> - § 1 reference coded [5.84% Coverage]

Reference 1 - 5.84% Coverage

I: What might be the reasons why health care workers who encounter adverse events following immunization are not reporting?

P: Two or more reasons. One will be workload .... is an additional work, if it has a lot of writing, plenty writing to write and then emmm you do that you don't get feedback and them people don't value it's importance emm so they just manage the conditions symptomatically as it comes not to continue worrying yourself and them and may be the last point could also be that oh they hope that ehm they may not think that you didn't do your work well that is why it came about

<Internals\\IDIs PROVIDERS\\NRPI_04> - § 1 reference coded [16.60% Coverage]

Reference 1 - 16.60% Coverage

I : What might be the reasons why health care workers who encounter Adverse Events are not reporting? [Laughing]

P : Like I said nu most of them they know.

I : Am talking of the health care workers?

P : Ehhhh Sometimes they know but ...... errh Okay ( laugh ) (inaudible). Because (coughing) ……Errrh ...... because last time they they were made to understand that is not a, a,a bad thing ehherr. So first I would have said may be they want to report because they think that oh they didn’t do their job well [child playing and making noise] does why the this thing, the effects are coming. So but they were made to understand that is not anything. Fever is fever, if someone get our injection, a fever from an injection is not because you didn’t do your job well does why the fellow has the fever ehherr So I would have said first that was the the fear was so may be they will think that people will think that they didn’t do my work well but now nooo is not the case.

I : So what ?

P : That is what am saying as of now thierr I can’t think of any thing Eherm because you know, the, the AEFI song they ‘v been singing it for a while, they just came they always tell them that oh is not .....when they come here for monitoring they say oh is not a bad thing if you report AEFY AEFI, is just, it just means that even you are even you are even monitoring the client self Errh.

I : So administratively do you think all is Okay?

P : All is Okay.

I : For reporting?

P : Ehm all is Okay!

I : What of staff issues?

P : Staff issues ….Like [nurse picking instruments for work] like I don’t understand the staff issues you are talking about?

I : Staff issues may beee the staff within the staff they also have certain issues that they put up. [ nurse picking tools]

P : Noo Noo [ Noise from consulting room]

I : What of logistics?

P : As for Logistics thirr is some how okay....... Aside from the fact that may be sometime it can be that we will be short of and if is short is short from the District. [ tools falling ] [ baby crying]

<Internals\\IDIs PROVIDERS\\NRPI_05> - § 2 references coded [20.06% Coverage]

Reference 1 - 5.65% Coverage

I: What might be the reasons why caregivers do not report adverse events following immunization to your health care workers?

P: Arr I think is arrh is always people don’t want to be blamed. There is perception that like if something happen, then they come then it will means like you didn’t do the work well is not about only those errh doing the immunization but all of us ..... errrherr like blame shifting so if, one of the this thing to to worked well. We have to take away with that blame shifting. Like, if like someone comes and I immunize the child and the child goes home and they bring back the next day that the child is having fever, the perception ….. not from the parent alone but among the staff themselves, will be that you didn’t do it well …. That’s why the child is having that kind of abscess or fever or whatever. Errherr so is because of this blame game …. That people don’t always want to report on those things.

Reference 2 - 14.41% Coverage

I: Okay ..... ok what might be the reasons why health care workers ..... *who* encounter adverse events following immunizations are not reporting?

P: Errherrr this what I thought who ask me the earlier on. This was what I was explaining. I think is because of what I say nuuu. Blame games like if … I reported I report it usually you know when, when it happens they come they say oh this nurse …. They will just say this nurse if they know your name they will mention if they don’t even don’t know your name they will say this person …. And when they say that … you know .....we like blaming ….. our colleagues too much or each other too much. When they come and they bring that instead us to report and attending to them nun we turn to blame the person, we say oh this person doesn’t know how to do this …. You see so is a stigma …. It has to stigma the one who injected that child or sometime it could even go out arrrhh this person injected this child and the child this was what happen to that child ....... So I think this are the reason why we don’t report and the second one also to be the awareness creation … may be some of us are not aware that we are supposed to reporting those things …. You may have a staff you doesn’t even know what AEFI is ….. in the first place so how do you even report something that you are not even aware. There are also other staff who don’t even know that you have to report it, is something that ..... must be reported. So how will you even report it, when you are not even aware, errherr so these are the I think the issues.

I: *Sooo*, *are* there not issues from administrative wise?

P: No I have not encounter that …… Errrrherr I have not encounter that, but I think the issues with administrative wise should also be that like you should be empowered, we should be trained on it. Errherr that should be the issues with them … *y*es! But I don’t think when it happen that was in June, July, when the case happen …. And the one that I said we reported I didn’t hear them blaming anybody for ..that . Errherr I was even on leave [they rather encourage them to be reporting] *ye*s! So when I came back it took a long time before I heard of it …. From a colleague. Nhmmmm.

I: So with regards to logi logistics, what do you say?

P: Logistics wise! We have the forms, is the human resource that need empowerment …. Yes but the forms are there.

<Internals\\IDIs PROVIDERS\\NRPI_06> - § 2 references coded [4.32% Coverage]

Reference 1 - 2.66% Coverage

**I:** So, what might be the reasons why Health care workers who encountered AEFIs are not reporting?

**P:** like I am saying, I have, it is not one case that I have had, I have had several cases and forms are not there. I hardly sit down to know the steps to follow to report. Sometimes if there can be way to manage it without reporting then I will just manage and go ahead. That is the basic thing.

Reference 2 - 1.66% Coverage

**I:**  Do you have any other reason in addition to that?

**P:** Some of them doesn’t know that even what they are encountering is AEFI, so that is why I said if there are trainings on AEFIs then it is going to improve about on the reporting on AEFIs.

<Internals\\IDIs PROVIDERS\\NRPI_07> - § 2 references coded [11.49% Coverage]

Reference 1 - 9.72% Coverage

**I:** what might be the reasons why healthcare workers who encounter AEFIs are not reporting?

**P:** yeah, like i said earlier, like a clinic of my setting, we see adverse effect to minor, they come with slight body temperature and which we don’t see as anything severe that should be reported and so for a patient as relative reporting to us, we give a minor management and then the patient get well and then we send them home.so just a minor documentation is done in the clinic but we normally do not report outside the clinic, yes.

**I:** could it be administration issues?

**P:** Not at all, we don’t have any staff issue. if there is any staff issue then i think that our category we are able to take good care of that so, we shouldn’t dont have to bother worrying the levels for such cases, yea.

**I:** Could it also ... ok, ok.

**P:** But like i said, in severe case we report appropriately and then we refer for further management.

**I:** Could it be logistic issue too?

**P:** Ah... yes, i think in most cases logistic issues comes and when the... err, we lack drugs sometimes we can ran out of drugs. We don’t have even the basic drugs. say hydro-cortisol for the management of adverse effect that normally comes in or adrenaline. so when it happens like that we won’t have any option but to refer but if we have we think is is appropriate to manage, but we dont think that logistic are normally the reasons why we either dont refer or we report. Either we don’t report or we report. Logistics are the major issue in this case.

Reference 2 - 1.77% Coverage

**I:** But do you have the reporting format?

**P:** I have, we don’t but i think that even if dont have we could always or still report without a form. Yes we can report without a form but indeed we dont have. Logistic but in a case we have one we can still report without the form.

<Internals\\IDIs PROVIDERS\\NRPI_08> - § 1 reference coded [16.46% Coverage]

Reference 1 - 16.46% Coverage

**I:** Mommy, what might be the reasons why Health care workers who encountered AEFIs are not reporting?

**P: [**Laughter] this is a big question. This one dear, what will I say? It is all about the attitude of the staff. They will come but I don’t know why they are not reporting. They will come and they will come because even fever, when the woman comes back with a complain of fever. It is an AEFI, They have to listen to the woman and attend to her but I don’t know why the health workers are not reporting. I know why, maybe we need to still talk to them on the importance of this thing. [Still laughing]

**I:** Could it be administrative issues?

**P:** Administrative Issue? I don’t think. That I don’t think. I don’t think because, if it is part of your job you are giving the immunization and you know that this is a problem. If you immunize and the woman comes back to say that this has happened, you have to…it is part of your work. You have to report and send it forward. You have done your part and so whatever is left is not your this thing again. **I:** So, that one, we say that it is the attitude of the staff, healthcare workers.

**P:** Hmm…I think it is the attitude of staff**.**

**I:**  But it can’t be logistical issues too?

**P:** Logistics Issues??

I: Like if they don’t have the reporting forms.

P: Forms?? No! The reporting forms, as for our Centre here, there are there it appears to be there. As for our reporting forms they are there. We take note of them and if it is left with the last form self we quickly make photocopies to put down. We never ran short. So that is not a reason. **I:** So, how can we improve the overall process of reporting AEFI?

**P:** We have to let the training be frequent and also monitor them to make sure they do the reporting.

<Internals\\IDIs PROVIDERS\\NRPI_09> - § 1 reference coded [7.54% Coverage]

Reference 1 - 7.54% Coverage

I: What might be the reasons why health care workers who encountered AEFI are not reporting?

P: Because of lazy because of busyness at the worksite

I: busy

P: they are busy the for and then 2 the workers the forms are not may be readily available and 3, some of the mothers don’t report don’t tell us after we telling them they should to us they don’t turn up they don’t tell us they don’t turn us they turn to tell us they just always be in the house to manage it themselves

<Internals\\IDIs PROVIDERS\\NRPI_10> - § 1 reference coded [5.39% Coverage]

Reference 1 - 5.39% Coverage

I: What might be the reasons why health care workers who encountered AEFI are not reporting?

P: as it may be due to lack of knowledge on AEFI some may not also see the importance on reporting AEFI and some deliberately may not report.

<Internals\\IDIs PROVIDERS\\UEPI_01> - § 3 references coded [32.96% Coverage]

Reference 1 - 2.18% Coverage

I: ok … so has there ever been a reason where they’ve not reported a case like that, a case of an adverse event following immunization?

P: unless … because for this our setting the moment a child takes a vaccine or immunization and gets home and is having any unusual feeling they will bring the child back to us they will bring the child back to the facility they will do mhm unless they don’t suspect that it’s due to the the drug but the moment it’s even, it happens the if once the know that it’s from the drug they will bring the child immediately to the hospital

Reference 2 - 0.77% Coverage

I: ok so meaning they always come when they detect

P: oh they will come some some will come and we have to reassure them yeah and tell them that they should, they should be calm everything will be ok

Reference 3 - 30.01% Coverage

I: so what might be the reasons why health workers errm health care workers who encounter an adverse event following immunization are not reporting?

P: yeah errrm as am saying you know sometimes errm errm we see that when a health worker errh errh should encounter a child if it’s a minor this thing most often they turn not to report it, uhuh if it’s a a a minor errh this thing but if its severe that’s when most often report uhuh but if it’s a minor this thing, and even if there is a problem we always want to rule other possible causes before we can be sure to report uhuh but most often you know some common presentations, common and mild symptoms so we turn to reassure them and then manage because this thing if it’s not anything serious and you report it raise national interest uhuh meanwhile they will follow up and realize that after all it’s not anything serious you understand? mhm because this this, there was this case about this drug that we give the albendazole and then abemethane imagine the teacher he took the drug today today, and we suspect that adverse events within 30 minutes to an hour you should start manifesting, you understand? but somebody waited aaahhh that very day let’s say around 10 am you’ve given drugs to the students and you took some yourself you wait aaah the following day around 10 then you came here that you are itching you understand uhuh so we also studied the trend and the time so that errh we know that is is we try to be sure it’s certainly due to the drug before we can report we don’t just report every case like that we have to rule out errh

I: ok so errm how about administrative errm issues, is it could it be a reason that health care workers do not report adverse events following immunization

P: administrative issues like?

I: errh I wanted to know what could you know errm errm the reasons that may I mean let a health care worker not to report these adverse events following immunization and per you explanation you’ve given that it if it’s not severe you don’t want to report it

P: mm

I: I want to know if maybe administrative issues too could be the reason why some people may not report it some health care workers may not report this

P: no you know adverse events are cases of interest that everybody is looking up to uhuh so administratively what is it, if the the reporting formats are thereuhuh so if there is a case they quickly alert the in-charge of the facility oh this is this or they might even call (inaudible) I come we will access the case together then we say oh for this case lets fill a form, let’s call the next level and let’s manage it this way then together we manage it

I: ok... so are there any other reasons … because now administratively you said the document I mean the report forms are there and then another way was errm you said was if it’s not serious so we’ve realized that there’s been an adverse event I mean there’s a low reporting of adverse events following immunization so we want to know more or what other causes could errm make this adverse events following immunization not be not to be reported .. as it should

P: yeah (clears his throat) that is … administratively that is if the case that presents (inaudible) proper history is not taken for which you really don’t attribute it to the this thing that is why when the mother come and you are able to take proper history then you are able to diuce that its due to a drug orr its due to immunization then the the the the staff should also be aware that this kind of case I need to report mhm I need to report but if the happen to meet a staff who will not know that this the case that I need to report the staff might just bring the case and (inaudible) uhuh but if you should meet a staff and the staff know that oh for this case it’s a reportable case we have to report on it then the staff reports uhuh so consentizing every staff is also very important so that we will be aware (inaudible) so u just mentioned about staff so could it also be staff issues?

P: yes

I: ok can you elaborate more on that, like what could be the issues with staffs that would errm make some health care workers not to report this

P: …. That’s what am saying uhuh, it depends on the experience of the staff, if the staff is experienced enough to know that for this case I need to report, then the staff will report so but if the staff is not that experienced he might not even report on the case

I: ok so ermm sorry if am sounding a bit repeated erhh repetitive like I wanted to find out when we spoke about the administrative issues you spoke and then said the forms where there so I want to know interms of logistics are there errh could any logistical you know things you know hinder this health workers from still reporting this … aside the fact that their their the forms are there so could logistics be a reason

P: yeah because even if you fill the form you need fuel to deliver the form to the next level and even the calling at the next level you need credit to call next level to tell them this is what we have here that is it so earlier am sure I may have and then your staff strength too

I: your staff?

P: your staff strength atleast some of these cases you need to have … the right person as am saying this facility we do not have a disease control officer but these are cases that the disease control officer is to be doing that running uhuh because and it is so it means that sometime there can be serious pressure … you understand and if the staff strength is not there you, for you to get up immediately and start running without report nu you might say well let me fininishat the end of the day when am done attending to all the clients then I will go, so the staff number is also a factor

I: ok

P: mhm

I: ok... so ermm with that how can we improve errm reporting of these adverse events following immunization among health care workers

P: yeah we need to have adequate staff

I: adequate staff,adequate staff in terms of number and category in terms of number and category uhuh then they should be resource needed resources should be provided then the needed training is (inaudible) should be organized so that they will whip every staff interest … so that every staff really have that interest and be on the lookout for all those things so that together we can work as a teamand is is something that we should even be giving education during our outreach points and then statics and then even during OPD services we should give education to the people so if the staff strength is not there we need the disease control officer need the health promotion officer we need the the community health nurses then the clinical staff uhuh so and then we need the community volunteers so that they can all be on the lookout for all this cases so like the overall process for reporting AEFIs how can we improve on this … how can we improve on the overall you know I spoke about the health care facility here, how can we improve on the overall health care I mean errm process of reporting ,yes so the process of reporting … yeah the overall process of reporting that’s why am saying if the appropriate staff are at post … you understand, so this one is call this case so this a reportable case then the disease control officer can take care of the reporting aspect, the clinical nurses will take care of documentation and the management of the case then at the district level sometimes you might even have a case you are calling the disease control officer you are not even getting him you understand mhm but if there should be an office line somebody should be … uhuh should always be at the office uhuh so you call and you can get the person but if the fellow is somewhere you are calling and you won’t even get the person so that you know the appropriate steps to take

<Internals\\IDIs PROVIDERS\\UEPI_02> - § 1 reference coded [14.41% Coverage]

Reference 1 - 14.41% Coverage

I: Ok. So what might be the reasons why healthcare workers who encounter adverse events following immunization are not reporting?

P: Well, for this my facility we when we encounter we report, but other facility I can’t talk for them (Ok). You understand? (Umm). But here, any adverse we sit for meetings, we talk; anything when you see adverse report, it doesn’t mean that when somebody get adverse effects we don’t know how to immunize or you don’t know your work (Ok). We’re not all perfect (yeah). You don’t know what has happened probably. Probably it might not be the way you immunized the child, but the problem might even come from the vaccine (Ok). You understand? (Yeah). So we talk to ourselves. Don’t feel shy that O because I’ve immunized madam A child and he has come back with adverse effects it will be like they’ll lose confidence in you. No, no, no. So we report…our facility we report.

I: So you you…you said you report, but could there be a reason, maybe administrative issues that would delay or prevent a health worker from reporting an adverse event following immunization?

P: Well, here there are no delay. As I said, you pick a phone, call if it’s like that…you make a phone call and you fill your form. So when immediately you call them too they respond and go ahead just like I called and they gave me go ahead.

I: Ok. But you were talking about maybe within among yourselves as staff you talk to yourselves, encourage yourselves to report

P: Yes.

I: But do you really think there could also be a staff issue that is…could I mean prevent health workers from reporting this?

P: No. there’s no staff issues. As I said, we’re only 3 – the 3 CHOs (Ok) or 3 CHNs; we give the immunization. You understand. So we all know the importance of adverse effects. As I said, maybe it may not be how you’re administering it – how you immunized the child that caused it, but it could even be the vaccine. You understand. So when you report and they’re able to detect it early you see it will even prevent more harm. Uhmm.

I: So I know maybe I’m sounding like I’m repeating some of my questions but I just want to know more

P: No problem. You talk.

I: So in terms of logistics you mentioned earlier that maybe you take a phone and call (yeah), but do you think logistic issues could also prevent health workers from reporting?

P: No, I don’t think. For us, there is no any logistics really that will prevent us from reporting.

I: So how can we also improve adverse events following immunization reporting among healthcare workers?

P: Among healthcare workers?

I: Yes.

P: That’s why the other explanation, I made mention that we need to talk to ourselves. It doesn’t mean that when there is an adverse effect, you don’t know your work. You understand? (yeah). It may not be how you immunized the child or how you administered the vaccine to the child, but probably the fault might be coming from the vaccine itself. You understand?

I: So aside that explanation, is there any other way to improve the reporting?

P: The reporting?

I: Yeah.

P: That’s why I say don’t feel shy to report (Ok). You understand. Because we’re all human – we’re not perfect, and anything could have happened (Ok).

<Internals\\IDIs PROVIDERS\\UEPI_03> - § 2 references coded [14.29% Coverage]

Reference 1 - 6.97% Coverage

I: ok so errmm what might be the reasons why health care workers who encounter adverse events following immunization are not reporting?

P: mmm some will all some will say some will say that maybe they when they report and then there is an investigation maybe they at the end, they will be implicated because some of the adverse effects could be the ne the negligence of the health staff ehuh because it could be where the position you have you were not supposed to immunize there then you immunize there or the particular vaccine supposed to be given to a different side and you have given it to a different side or even how the potency of the drug it could be that the drug have expired and you have not check and you use so sometimes some fear that maybe at the end it will it will bounce back to them so that’s why some of them the for the overlook some of the some of the adverse events and they don’t want to report but its better you report so that even at the end you will now learn lessons on it or even learn more about it, it could be that the even that it is not your fault it’s the vaccine that is not good because if you are when we give the immunization we write the batch number and every vaccine have its batch numbers so sometimes that way if you even report they can track to get the batch number then it could be that the fault is coming from manufacturer not you some sometimes we pursue that maybe it’s our fault so reporting that means you are reporting yourself so these are some of the perceptions some of the health staff have and then they don’t report

Reference 2 - 7.31% Coverage

I: ok … so are there any other reasons?

P: … and some also say they report and then they don’t get feedback from where they have send the report so that one also make them lazy too

I: ok how about administrative issues could it be a reason
P: administrative mmm it could be a factor assuming they have to send the report to the next level there is no fuel,they person is not also having money with her or him then it becomes a problem

I: mmm ok how about staff issues

P: staff issues is also one because sometimes they can say ooh so far as this person is not there then because they won’t they person can come even the wont the person can come and then if they think this particular staff is not there then they either send the woman to go back or they can take the thing and even now waiting till you come before the thing can be forward

I: ok

P: so staff attitude can also be a factor

I: mmm

P: but it varies that’s why I said

I: mmm ok any other?

P: mmm I think these are the things that

I: how about logistics

P: logistics, maybe shortage of the (knocking) the reporting form

I: form ok

P: can also come in.

I: ok

P: because it’s just a matter of getting the information and other things it will now get to if it will now get to maybe they need other investigation fine

I: mmm

P: but the first start you if you have the reporting form then the there is no staff who is not will not will complain will say he doesn’t have, he or she doesn’t use. You can’t be going to farm without your hoe.

I: mmm

P: so pen shouldn’t be a problem.

I: yeah (laughing)

P: uhuh

I: ok so are there any other you want to add to the reasons?

P: no

<Internals\\IDIs PROVIDERS\\UEPI_04> - § 1 reference coded [30.82% Coverage]

Reference 1 - 30.82% Coverage

I: okay, my next question is similar to the above, so I want know what might be the reasons why caregivers do not report AEFI’s to healthcare worker?

R: either they always think it is too sample and so it’s not necessary especially the fevers, they always feel that it will go away so the follow is not serious

I: is that all please?

R: yeah, to mine this thing that is how I see it

I: what about the administrative issues regarding that

R: well, at the level here like I said, all the unit they know that this booklet is there and if you get a case, it is just the matter of coming to pick it and report on. So management is not holding this book at a place where they cannot find it and even at the immunization site, they have their forms, the forms are there we photocopy it for them. So every staff so administratively I don’t think there is something hindering them from reporting

I: oo okay, what about staff issues resulting in healthcare workers not reporting AEFI’s

R: staff issues, even if you are one staff and you are there and the case come, you are to report. It doesn’t add up anything. Okay, some too may thing like just like I said, sometimes they just think it is not a serious case. So they come they are advising them so that if it is fever they try to reassure them. I think that is it.

I: okay, with regards to logistics what can you say about that

R: logistics as I said the forms are there and that is the only thing, whiles the forms are there that’s the logistics for reporting

I: thanks for response, in your own words what do you think need to be done to improve AEFI’s among healthcare workers?

R: okay, we have to let them understand that once the come to report, we should also report we shouldn’t wait and say that this is serious and maybe this not. It is important that, once the come back to report we should also report. May be during the trainings we should intensify and let them understand that every report that comes they shouldn’t judge which one is serious and which one is not.

I: is there anything to add?

R: that’s all I have to say

I: madam in your own words, how can we improve the overall process of reporting AEFI’s

R: ahh maybe we should add it to our monthly report so that every month it should be part of our reporting system. Now that we are dealing with the DHIMS, I don’t know if there is a portion for us to report but if there is not, they should create one so that we can be reporting there monthly

I: Madam my last question, can please tell me other issues that you would like me to know with regards to reporting of AEFI’s in general?

R: as I said maybe the electronic reporting if they can widen the scope, so that, the staff can also know the format of reporting. I think that is it.

<Internals\\IDIs PROVIDERS\\UEPI_05> - § 1 reference coded [5.74% Coverage]

Reference 1 - 5.74% Coverage

I: alright sir, thank you, so what might be the reasons why care eerrh, healthcare workers who encounter AEFI’s are not reporting them?

P: if they are not reporting they may not, they may be is part of ignorance otherwise if it is part of structured for them to report, is is it should be reported because some if some of the forms (greeting from a client) is very important.

I: okay sir, mmhm apart from structured way of reporting would you think that there might be having some untold fears, administrative issues or something?

P: mmhm, am not quiet too sure about what administrative fears would be because if you are administer any vaccine or therapy and there is any adverse effect I don’t think there is fears to report. After all, I think that adverse effects are either accidental or natural phenomena because it can happen as an accident, even the best driver can get an accident with his car, is that not it? So it’s normally we have to it should be official. Nobody should be afraid of reporting, if the thing you have done it and it occurred as an accident, why won’t you report but you know sometimes it may be true that the fear of the authorities but I am not tempted to belief in that.

<Internals\\IDIs PROVIDERS\\UEPI_06> - § 1 reference coded [7.20% Coverage]

Reference 1 - 7.20% Coverage

I: so what might be some of the reasons healthcare workers who encounter AEFI’s are not reporting them?

P: mmhm hummm, I will be surprise if somebody a healthcare provider gets this and will not report because a form is already given you. Anytime you get an adverse reaction report to us and if somebody doesn’t report then then then there is a technical issue that I may not be able to tell, maybe is a funding issues. If you have not given the person credit to call you, you have not given the person fuel for these activities, how will the person get to you, you understand. It can be logistics problem too it can be logistics, you understand, there is no fuel well, you have not given them credit, if I don’t have credit now you know somebody is working but he has a family, you know in Ghana the dependency ratio is high and so you are already having financial crisis you understand, so you need to call, you need to travel by a motorbike using fuel if those things are not there what happens, it means our resources is one of them

I: will there be challenges administratively? Will there be any administrative issues that you think probably is the reason why others still will not want to report?

P: administrative, even if it is administrative it has to boil down to the resources that am talking about, yes if it is even administrative maybe resources that’s why I was saying that the last time what I wanted us to do is that, it shouldn’t be only the disease control officer monitoring the whole community, we should have one staff monitoring these communities, we have another staff monitoring this community even though the disease control officer is the overall head but everybody will take part and everybody will be working. If you receive a call I have only one community, when my volunteer calls me I can go rush there but if you have so many communities, 15 communities supervised by only one person you see you can see how tedious it is and it is because of resource constraint (laughs heartily)...
