# Supplementary material for: Optimising reporting of adverse events following immunisation by healthcare workers in Ghana: A qualitative study in four regions
Source: PLoS One. 2022 Dec 20;17(12):e0277197. doi: 10.1371/journal.pone.0277197 (PMC9767370; doi:10.1371/journal.pone.0277197)
Supplement: S1 Data — (ZIP) [file pone.0277197.s001.zip › Minimal data/S2. AEFI data storage.docx]

**Name:** 08. AEFI data storage

**Description:** This node contains all information on how AEFI data are stored

<Internals\\IDI EPI\\GAEPI_01> - § 1 reference coded [8.44% Coverage]

Reference 1 - 8.44% Coverage

I: so we will like to know how the AEFI data is stored. Whether it’s in a paper form or electro… [Inaudible]

P: it comes first as paper form and then we create a data base for it… and we now send that paper, a copy of that ‘erh’ I mean paper to the food and drug’s authority that’s where they finally end up and they work on it. So here, we have ‘erh’ database with the underlying lists of all the ‘erh’ reported AEFIs *to us* and then I mean ‘erh’ we share it with FDA plus the hard copies that have been received. Some regional officers send them, they scan and send, send to us. Others too post them to us so there are different but eventually it comes as either scanned or hard, hard copy to us then we put the key core variables into our, our, our database

<Internals\\IDI RHMT\\GARI_01> - § 1 reference coded [1.66% Coverage]

Reference 1 - 1.66% Coverage

I: so what about, how is the AEFI data stored?

P: oh, we have a form. It’s just like a line list. We have a form and then we have line listing then we also have ‘erh’, ‘erh’ software on those forms, those line listing and they store it there

<Internals\\IDI RHMT\\GARI_02> - § 1 reference coded [4.61% Coverage]

Reference 1 - 4.61% Coverage

I: we would like to know, how is the AEFI data stored? Whether paper form, electronically, software*?*

P: it’s paper! It’s paper. Normally they don’t bring it to my office. We take it to Uncle Atoo’s office ‘uhuh’. So that’s the disease control office. So that is where they keep the forms. I don’t know whether they keep copies but when they bring the forms we also forward it to headquarters and then to ‘erhm’… standard’s… authority. ‘*Err’* and sometime ago I think they said the forms were also with the pharmacist or so, so I don’t know, it becomes difficult. so

<Internals\\IDI RHMT\\NRRI_01> - § 2 references coded [7.04% Coverage]

Reference 1 - 5.41% Coverage

I: Ok, can you please add more. How is the AEFI data stored?

P: As for storage, hard copies you can always find them in files.

I: Ok.

P: But you can find mostly, they are stored. What do use? What program do you use?

P1: Programs?

P: How do you store?

P1: The data is captured, it depends on the level in which if it is at the regional level how is our data store, we have soft copies and we have the original “errr” the hard copies too stored in files in a hard copy and the soft copies is also stored in the PC.

I: Ok, soft copies.

P1: We don’t know storage as in? May be if you.

I: Weather in pen drives, now we can store in iCloud or is the hard drives, external hard drives like the soft copies.

P1: From where to where? If is at this level?

I: Yes, at the regional level.

P1: “Yaah”.

I: When you receive the data.

P1: When we get the data, they bring the hard copy we have a software that has been given to as by National.

I: Ok.

P1: Which is inputted and the hard copy is filed.

Reference 2 - 1.62% Coverage

P: That’s (not audible) inadequate.

I: Were the reports sent by paper or it was electronic?

P1: They usually will bring the paper, they fill the form. The facilities this level at their level it will be difficult transferring electronic copies to us, they bring as the hard copies then we input it.

<Internals\\IDI RHMT\\NRRI_02> - § 4 references coded [6.14% Coverage]

Reference 1 - 1.70% Coverage

I: How is the data stored?

P: “errer” ‘hmmm’, I will not be able to to to to say how it is stored for now, like I said my training has been some time ago and of recent I don’t know. You know with this “errrrr”, when the forms are filled you know, those days I was working at the district level, we keep a copy.

Reference 2 - 1.84% Coverage

I: Ok.

P: We keep a copy and we submit a copy, so at the DHMT level we keep a copy and submit the other one to the higher level, so from that level regional to national level and to the “errrr” FDA level I don’t know how it is stored. (Laughing) but at the district level I was working in the District and then we just keep a copy.

Reference 3 - 1.30% Coverage

I: Ok.

P: “Aaaa”, so that in case they now come back for a down “errrr” stream follow up we will be able to trace “errrr” to the facility of reporting and then to the community of reporting and then to the client “errrr” who reacted.

Reference 4 - 1.30% Coverage

I: So is the report always electronic or is paper?

P: Well, those days we were having it in paper but this time we can decide to actual “errrr” make it electronic but the time that I was (laughing) I was privy to, it is paper. Is paper.

<Internals\\IDI RHMT\\UERI_01> - § 1 reference coded [4.37% Coverage]

Reference 1 - 4.37% Coverage

M: so how is the adverse event following immunization data stored?

R: that much I don’t know I think it would be the EPI coordinator that would be in that position to tell because we don’t collect the forms or information as regional public health nurse but for the EPI board they collect and I think they transmits to food and drugs authority. That is what I think but I don’t know whether that is the case I actually will not be able to tell.

<Internals\\IDI RHMT\\UERI_02> - § 2 references coded [6.56% Coverage]

Reference 1 - 4.01% Coverage

M: How is AEFI data stored?

R: AEFI data I think it is part of the surveillance programme, the later part of it because those days we used not to put AEFI’s on the surveillance. But as surveillance revolves overtime it came as part of the surveillance programme. So it is supposed to be reported to the surveillance officer using the integrated diseases surveillance system but as I said it is not very regular. As I said, sometimes we have campaigns mass vaccinations against a particular preventable disease then the data is also reported and that one comes to the EPI coordinator who shares with the surveillance officer and then they are forwarded to the FDA. So we have routine and special programmes like mass campaign.

Reference 2 - 2.56% Coverage

M: So is the data stored in paper form or electronic form?

R: It is supposed to be both because for us in Ghana health service what we do is dime data based and that is electronic but we still have the hard copies that we fill and file and scan to the top. That is core evidence because for electronic it is just aggregate data you can talk about the numbers but the specific information regarding the event you cannot find that in the data base so we use both.

<Internals\\IDI RHMT\\VRRI_01> - § 1 reference coded [1.78% Coverage]

Reference 1 - 1.78% Coverage

I: emmh, how is the data stored?

P: Errrh, like I mentioned, we have a data manager

I: ok

P: at the public health unit,

I: ok,

P: so it’s sent to him and he stores them. He create a folder for them.

I: Electronically?

P: Yes, electronically, so he stores them. And he also forward to national.

<Internals\\IDIs DHMT\\GADI_01> - § 2 references coded [9.00% Coverage]

Reference 1 - 3.87% Coverage

I: So how is the AEFI data stored?

R: The reporting, I have 5 sub districts under me, so when you report, you do the photocopy and keep the copy but electronically, we have the DHIMS that is entered into so electronically yes.

Reference 2 - 5.13% Coverage

I: So you have access to that information?

R: I have access to that information as a district disease control officer, but when we send the reports we send the in hard copy but with the DHIMS its just records the number but the details will be on the form you filled so you can know the batch number.

<Internals\\IDIs DHMT\\GADI_02> - § 1 reference coded [2.66% Coverage]

Reference 1 - 2.66% Coverage

I: How is the AEFI data stored?

R: I cant say much because we have not had cases but I know there should be aprocess of storing the data.

<Internals\\IDIs DHMT\\GADI_03> - § 1 reference coded [4.57% Coverage]

Reference 1 - 4.57% Coverage

I: So how is the AEFI data stored?

R: So usually my former district we use to keep the hard copy on a file so we have a file where the disease control keeps a copy and the district pharmacy also keeps a copy because he is the main focal person for taking he forms and forwarding to FDA, so that is how it is stored hard copy not softcopy.

<Internals\\IDIs DHMT\\GADI_04> - § 2 references coded [2.63% Coverage]

Reference 1 - 1.81% Coverage

I: So how is the AEFI data stored?

R: We are to send it to the next level so the storage am not too sure.

Reference 2 - 0.82% Coverage

I: Is it paper based?

R: Yes it is paper based.

<Internals\\IDIs DHMT\\GADI_05> - § 1 reference coded [2.72% Coverage]

Reference 1 - 2.72% Coverage

I: How is the AEFI data stored?

R: I don’t deal directly with it so I can’t tell but the pharmacy should have a reporting system.

<Internals\\IDIs DHMT\\GADI_06> - § 1 reference coded [1.54% Coverage]

Reference 1 - 1.54% Coverage

I: How is the AEFI stored?

R: We have the DIMS but we have the hard copy as well, we have the EPI we have to fill a form, sometimes because we don’t have the form to support what we are dealing with.

<Internals\\IDIs DHMT\\GADI_07> - § 1 reference coded [5.96% Coverage]

Reference 1 - 5.96% Coverage

I: please having said that about the form, we would also like to know how the adverse event following immunization data is stored. Is it a paper form or electronically software?

P: ‘erh’ in our setting, it is stored ‘erh’ in a paper form yes, because of the way our system is, it is not too reliable nowadays. So once you have, even if ii is stored electronically, you have to have the paper backup ‘uhuh’. So that if you lose information. Yes. So there is a file [claps] for that one which is kept at the disease control office, yes.

<Internals\\IDIs DHMT\\GADI_08> - § 1 reference coded [1.45% Coverage]

Reference 1 - 1.45% Coverage

I: So how, how is the AEFI data stored? Is it in the paper form or…

P: In a paper form, in a paper form.

I: We are almost done.

P: In a paper form.

<Internals\\IDIs DHMT\\GADI_09> - § 1 reference coded [2.54% Coverage]

Reference 1 - 2.54% Coverage

I: so please how is the AEFI data stored? Is it in a paper form or is it electronically?

P: in the paper form ‘hmm’ in the paper form. I don’t know if now there have put it on DHIMS [door opens in background] but it’s a form that you fill then they collect everything and then forward it to, I don’t know whether standard’s board, somewhere! ‘hmm’

<Internals\\IDIs DHMT\\GADI_10> - § 1 reference coded [1.18% Coverage]

Reference 1 - 1.18% Coverage

I: So the next question says ‘erhm’, how is the AEFI data stored? Is it in paper for or a software?

P: Yes, it’s paper. Because it’s a form we fill so we keep a copy

<Internals\\IDIs DHMT\\GADI_11> - § 1 reference coded [5.80% Coverage]

Reference 1 - 5.80% Coverage

I: okay. Now we would like to know, how is the AEFI data stored? Is it in a paper form or electronic form, software we would like to know? Electronic form, software, we would like to know

P: what I know is hard copy form that we have [talking in background] ‘hmm’ but I don’t know whether it reach the district, okay…when it reach the district level they…do electronic before the ‘erh’ hardcopy go to the metro

<Internals\\IDIs DHMT\\GADI_12> - § 1 reference coded [3.82% Coverage]

Reference 1 - 3.82% Coverage

I: So please how is the AEFI data stored is it paper form ‘erhm’, electronic, softare?

P: yes. We have one in the paper form. You know, the hard copy then you have to make sure you put it on the electronic copy. They gave the electronic version when we were doing ‘erh’ this thing, AE [inaudible] measles SIE way back; I think two, three years ago.

<Internals\\IDIs DHMT\\GADI_13> - § 1 reference coded [6.14% Coverage]

Reference 1 - 6.14% Coverage

I: we will also like to know how the AEFI data is stored

P: AEFI data is stored on files in….at the various levels. So if you come to the district health directorate, you would find AEFI forms on files. We have AEFI FILES on…in the disease control office. In our files, there is one for AEFI’s. So you will find all the forms here and we would have sent copies to the region. So we have hard copies. And then also if you go on DHIMS, you would find our reports there.

I: is it software?

P: the softcopies of our report

I: the DHIMS, is it a…

P: the DHIMS is internet based. So you will find it online

I: we will also like to know if your district has received AEFI report in the last year?

P: This year….yes!yes! At least we’ve had one, at least

<Internals\\IDIs DHMT\\GADI_14> - § 1 reference coded [3.78% Coverage]

Reference 1 - 3.78% Coverage

I: So please the AEFI data how is it stored? Is it in paper form or electronically?

P: [Participant fanning with a paper in background] They have a form, they have a hard copy that they must submit ‘uhmm’ so after they have submitted the hard copy I don’t know what the disease control officer how he forwards it [Talking in background]

<Internals\\IDIs DHMT\\GADI_15> - § 1 reference coded [6.28% Coverage]

Reference 1 - 6.28% Coverage

I: okay, so please, how, how is the AEFI data stored? Is it the paper form or electronically?

P: yes, we for now all those that I have recorded I have it on a data, on a [chuckled] hard copies and then the soft copies too is there on an excel sheet but there is no erhm we, we use, we pick the various parameters on the form to create something on excel sheet “uhuh” but it’s not “standardized” form that national or region has come out that this is a soft copy so anytime you report AEFI enter into it and then we also submit the hard copies to the region.

<Internals\\IDIs DHMT\\NRDI_01> - § 1 reference coded [4.72% Coverage]

Reference 1 - 4.72% Coverage

I: how do you store your AEFI data?

P: mostly is with the disease control officer, mostly whatever case that we get relating to that, if the health worker that is may be the case is reported to we get the disease control officer informed about it so after every process he keeps the data.

I: you don’t know whether it is in electronic form or paper form.

P: Anyway what I know of is he paper form, the form that we fill but the electronic I really don’t know whether he *does* enter it in a this thing as for that one I don’t know much about that.

<Internals\\IDIs DHMT\\NRDI_02> - § 2 references coded [3.28% Coverage]

Reference 1 - 1.38% Coverage

I: Sir, another case is about AEFI data how do you store them when you receive them.

P: I think the disease control unit should have a file for filling.

Reference 2 - 1.91% Coverage

I: Mmm but you don’t know whether it takes paperless eh paper form or ----

P: is paper is paper.

I: No electronic form?

P: I don’t know! I think if it is reported in [DHIMS] then it will be otherwise is paper.

<Internals\\IDIs DHMT\\NRDI_03> - § 2 references coded [8.03% Coverage]

Reference 1 - 3.76% Coverage

I: In respect to data collection like on AEFIs how do you store them?

P: Data collection on AEFI!

I: AEFI that is those which have been reported to you…

P: Normally, yaah normally when they report you know I said we have a coordinator here so is through coordinator that I channel the information so that the forms are sent to the region.

Reference 2 - 4.27% Coverage

I: So when you take them do you store them in a data form. What do you call it electronic form or paper form?

P: No! No!! No!!! normally some of the AEFI at times the staff himself send to the region because I directed them where in case I am not there normally I don’t stay in case I am not there they know where they will forward what is it the form to that is the EPI office in Tamale.

<Internals\\IDIs DHMT\\NRDI_04> - § 1 reference coded [2.69% Coverage]

Reference 1 - 2.69% Coverage

I: How is the adverse events following immunization data stored in your district?

P: Arrrh (inaudible) it is like I said earlier on they report it if if the form is filled it is given to the disease control unit, even if I receive it and he is not there when he comes because they have the files with them so they have copies to file with them and probably the,e, sub-copies if they have any other thing they can enter, they have a template that they enter and that one is with the Disease control unit. Nhmmm. These are the things that we do it.

<Internals\\IDIs DHMT\\NRDI_05> - § 1 reference coded [6.00% Coverage]

Reference 1 - 6.00% Coverage

I : Is it stored in paper form or electronic ......... at your district level?

P : I think our level ......... ohhh is both ...... both in the sense that ...... they will use the had copy to report ..... I think the we we even if they get one within a month ... it has to be reported immediately and then the transmission of the form to the next level but it is entered on the monthly immunization report form wherrrre it goes into the Dims .... But if there is no report probable it will be zero reporting for may be a particular month for number of ehhhh if there is none but where you have may a number of cases is reported and I think it goes into the Dims, apart from that I don't think we have any software ..... where specifically but like I said I remember some years back some three four years ago a woman came from ehhh CDC Atlanta and installed in fact I think it was it was on a trial bases and that you can just enter the information, you can even print it out and what have you but it ended there like that because it was installed on my personal computer in fact the computer had a problem and we lost every thing but for the district this is how it is done..... Yaah!

<Internals\\IDIs DHMT\\NRDI_06> - § 1 reference coded [6.37% Coverage]

Reference 1 - 6.37% Coverage

I : Emmmm how is the adverse events following immunization data stored?

P : Emmmm.

I : Is it stored in paper form or electronic ......... at your district level?

P : I think our level ......... ohhh is both ...... both in the sense that ...... they will use the had copy to report ..... I think the we we even if they get one within a month ... it has to be reported immediately and then the transmission of the form to the next level but it is entered on the monthly immunization report form wherrrre it goes into the Dims .... But if there is no report probable it will be zero reporting for may be a particular month for number of ehhhh if there is none but where you have may a number of cases is reported and I think it goes into the Dims, apart from that I don't think we have any software ..... where specifically but like I said I remember some years back some three four years ago a woman came from ehhh CDC Atlanta and installed in fact I think it was it was on a trial bases and that you can just enter the information, you can even print it out and what have you but it ended there like that because it was installed on my personal computer in fact the computer had a problem and we lost every thing but for the district this is how it is done..... Yaah!

<Internals\\IDIs DHMT\\NRDI_07> - § 1 reference coded [3.33% Coverage]

Reference 1 - 3.33% Coverage

**I:** How is the AEFI data stored?

**P:** As I said, it is stored electronically, I mean through the electronica line system, once the form comes we enter it into the electronic license and if we store it that way fine. At least we now have in electronically, I mean apart from the hardcopy. As for the hardcopy at time you come, I want to trace some of the things and it is difficult but when electronically stored this thing particularly the net, how is that how do you call, the hard drive or maybe just on excel or this thing is not enough. You can put on maybe the I-cloud, yes or we input it into the limbs, it is permanently stored and retrieved at any time.

<Internals\\IDIs DHMT\\NRDI_08> - § 1 reference coded [4.65% Coverage]

Reference 1 - 4.65% Coverage

How, place (clears throat) how is the AEFI data stored

**P:** The data?

**I:** Yes the forms

**P:** The district control unit when they bring it or when someone gets AEFI, where events, we go to the disease control unit.

**I:**  Is hard paper form or is electronic?

**P:**  Paper, paper form, like this and they fill it and bring it

**I:** Ok

**P:** They will fill it and send to Disease Control Unit or they call the Disease Control will tell them to pick a form and bring it to them.

<Internals\\IDIs DHMT\\NRDI_09> - § 2 references coded [3.19% Coverage]

Reference 1 - 2.05% Coverage

**I:** How is the AEFI data stored?

**P:** For now we store them locally in our application file. Because we have a case base form so when they report, we have to file the form and submit another to the next level.

Reference 2 - 1.14% Coverage

**I:** Paper form or electronically?

**P:** Paper form, the electronic is a website so we don’t have access to repository.

<Internals\\IDIs DHMT\\NRDI_10> - § 2 references coded [5.20% Coverage]

Reference 1 - 3.05% Coverage

**I:** Doc., Please how is the AEFI data stored in your district?

**P:** The what?

**I:**  The AEFI data.

**P:** Data?

**I:**  Yes.

**P:**  Come in, it is superb. We are following what is supposed to be done. We fill them. We have never recorded any feedback.

Reference 2 - 2.15% Coverage

**I:**  Is it stored, paper base or…

**P:** Yes, paper-based reports but we enter it into our computer or is it (inaudible)

[Flipping of papers] So those are some of the things.

<Internals\\IDIs DHMT\\NRDI_11> - § 2 references coded [4.63% Coverage]

Reference 1 - 1.85% Coverage

I: Thank you very much mummy, we are almost done, how is the AEFI data stored in your district?

P: Is, one is the online that we can refer too and the other is the hardcopies that we , we keep copies of and then forward err, the rest to.

Reference 2 - 2.78% Coverage

I: Ok, the electronic, you send it by mail or?

P: “Yaah”.

I: And after wards, how do you store it? Just is it in your computer, hard drive, is it the pen drives or you store it in the cloud, external hard drives, how? (“Clearing of throat”).

P: The hard copies is filed in an “aaaa” file but the err soft copies are saved and stored on our computers.

<Internals\\IDIs DHMT\\NRDI_12> - § 1 reference coded [2.94% Coverage]

Reference 1 - 2.94% Coverage

I: Thank you very much. So how is, how is the AEFI data stored? Is it hardcopy like paper or is electronic?

P: “ah” is hardcopy.

I: Is it the only way you store it?

P: That much I can’t this thing, “hmm” because that much I can’t. they just put it in this thing and forward it to the err appropriate quarters. The disease control unit. Alhasan, how do you store it? Paper work or electronic? AEFI reports. We file.

I: Ok.

P: The hardcopies, so at the facility level too. Ah 8second “mmm” 10second so that is it.

<Internals\\IDIs DHMT\\NRDI_13> - § 1 reference coded [3.60% Coverage]

Reference 1 - 3.60% Coverage

I: How is the AEFI data stored?

P: mhm they fill they have some form which they fill and they send it to the region but we also have copies here at this district level we have copies and they the rest to the region

I: It is stored in a paper form or electronic [ ] phone belling

P: we stored in a paper form [ ] phone belling

<Internals\\IDIs DHMT\\NRDI_14> - § 1 reference coded [4.65% Coverage]

Reference 1 - 4.65% Coverage

I: How is the AEFI data stored?

P: the storage like when you got to the field and you have something like you need to fill a form and bring it to the district so the district will send it to the region

I: Is it stored on paper form or electronic

P: those days we were doing it in paper form but now electronic is now on top and you after completion of everything you put it in the electronic form.

<Internals\\IDIs DHMT\\NRDI_15> - § 2 references coded [4.76% Coverage]

Reference 1 - 3.08% Coverage

I: How is the AEFI data stored? [ ] Baby scream

P: the data usually the AEFI is reported together with other conditions so when you report on those one the AEFI is part

Reference 2 - 1.68% Coverage

I: Is it store in paper form

P: on files

I: or electronic

P: on paper and then electronic

<Internals\\IDIs DHMT\\UEDI_01> - § 1 reference coded [2.27% Coverage]

Reference 1 - 2.27% Coverage

I: ok so how is the Adverse Event Following Immunization data stored?

P: ok because arm I, my office don’t deal directly with the form so the report when, normally when they report it goes to the disease control officer (OK) and he takes charge of that so I don’t really know much.( TIME)

<Internals\\IDIs DHMT\\UEDI_02> - § 2 references coded [3.76% Coverage]

Reference 1 - 1.63% Coverage

I: So the data; how is the adverse events following immunization data stored?

R: Oh we you know when we have it, we fill a report and send it (Ok) but the disease control officer also collect these things (Ok). Eh so we have the forms with us.

Reference 2 - 2.13% Coverage

I: Ok. So is it stored electronically or just paper

R: it’s hardcopy.

I: Hardcopy

R: Yeah hardcopies.

I: You don’t have electronic version of the data?

R: No, no we don’t have electronic versions.

I: Ok. Does it mean that it’s only the paper version that you have for now?

R: Yeah. That is that is what we have.

<Internals\\IDIs DHMT\\UEDI_03> - § 2 references coded [5.72% Coverage]

Reference 1 - 2.02% Coverage

I: Ok. (laughs). Ok. So how is the the data, the adverse events immunization data stored?

P: Ehh. Thank you very much. What I do or we do here is that, I have a file here (Umm). Immediately they report from the sub-district (Umm)… and then bring the hardcopy here, quickly, I do photocopy, send one to your facility, and go and file it (Ok). I will also file one here (Uhmm) and photocopy another one and add to the right original (original one) one and also forward to the region. That is the way I have been doing it.

Reference 2 - 3.70% Coverage

I: Ok. So meaning it’s in a hardcopy form?

P: Yes.

I: Ok.

P: Exactly. Because the the form they give it to us, the software way nu you cannot enter data (Umm). You cannot enter anything on it.

I: Ok. And the softcopy (yes) it’s the same? Ok.

P: You try to enter something, you know when you design it and you can enter something on it, you will enter (Umm), but if you cannot enter, meaning you only print the hardcopy, when you come, that is the only thing that we can we usually do.

I: Ok. So I’m talking about when you’re going to store the data, aside the files like the hardcopy (Umm) you put in the files, do you store it electronically too?

P: Ehh. That is what I just said (Umm). I said the electronically (Umm), for example then we have to do photocopy (Ok). You get what I’m saying? (Yeah). Then you create a folder as a AEFI and put them there just like the this thing, because you cannot enter any this thing (thing) inside (Umm).

<Internals\\IDIs DHMT\\UEDI_04> - § 2 references coded [14.47% Coverage]

Reference 1 - 8.57% Coverage

I: alright sir. After these your forms are filled, how do you store the data on adverse events?

P: INFACT we have a copy at the, with the EPI coordinator and a copy sent to the region. We normally send the original to the regional health directorate, we make a copy to keep and the regional health directorate also forwards it to national but then they give copies to the food and drugs authority in Bolga, there is an office there and that is just the channel. When it is on general medications and we fill the form in fact we receive acknowledgement letter, we have a lot of such letters acknowledging having received the report we have made and when there is, there is a technical working group that sits on and an expert committee and when they come out with the findings they usually communicate back to the reporter. So to me it encourage a lot because I have had several of that and it encourage a lot, reporting (clears throat) and I think it’s a good one, if your email is there they send you a mail and that helps motivate the people to report more.

Reference 2 - 5.90% Coverage

I: and having had the benefit of feedback, does that trickle down to the facility level where staff who have encountered these have reported.

P: yes, any staff who reports adverse events in general not necessarily the EPI in general when the food and drugs authority is reporting back, they be giving us a feedback, they give the feedback to the one who fills the form, the reporter, they give the feedback to the district director, they give the feedback to the institutional contact person, so about 4 copies are always sent and that helps a lot. I used to be an institutional contact person before, a district hospital before a district director and some of the forms we filled, I was here when those feedback were sent to me.

<Internals\\IDIs DHMT\\UEDI_05> - § 2 references coded [4.33% Coverage]

Reference 1 - 3.41% Coverage

I: thank you very much, so how is the adverse event following immunization data stored?

P: eerrh when they get it eerrh, when it is recorded at the community; when I talk of the community level I mean facility level, it is reported to the DHA, the disease control they coordinate it so they have a file that they keep all those ones, so when they also receive the report from the field then they also forward it to the region for them to take further action on it.

Reference 2 - 0.92% Coverage

I: would you know if it’s only hard copy or I mean, it is also transmitted electronically?

P: am much aware of the hard copy.

<Internals\\IDIs DHMT\\UEDI_06> - § 2 references coded [1.77% Coverage]

Reference 1 - 0.46% Coverage

I: okay so when you, how is the AEFI data stored?

P: we create a file, put the hard copy there

Reference 2 - 1.31% Coverage

I: is that the only mode of storage?

P: yeah, yeah that’s the only mode. Mmhm DHIMS, DHIMS will just capture on the EPI reporting form, you have something like AEFI, how many AEFI’s and you report but talking about the detail of the patient, that one is AEFI form itself.

<Internals\\IDIs DHMT\\UEDI_07> - § 1 reference coded [2.57% Coverage]

Reference 1 - 2.57% Coverage

I: alright sir. Thank you so how is the adverse event data stored?

P: for us we keep the copies of the forms in files and then for the..... numeric’s that one is part of the monthly reporting forms and it’s entered in DHIMS.

<Internals\\IDIs DHMT\\UEDI_08> - § 1 reference coded [2.42% Coverage]

Reference 1 - 2.42% Coverage

I: okay, so how is the AEFI data stored, when you record cases how is the data stored?

P: that I cant tell, I think the disease control will be the best to tell.

I: okay, you have never encountered ...?

P: I was a community health nurse but I have never had any abscess or anything.

<Internals\\IDIs DHMT\\UEDI_09> - § 1 reference coded [3.45% Coverage]

Reference 1 - 3.45% Coverage

M: How is AEFI data stored?

R: For the storage it is the disease control officer that is coordinating that but the reports and everything goes to them directly.

M: For instance, the data that is picked on AEFI. Is it on paper or electronic form?

R: It is on paper that is brought here and we collate and send

<Internals\\IDIs DHMT\\UEDI_10> - § 1 reference coded [5.34% Coverage]

Reference 1 - 5.34% Coverage

M: So how is AEFI data stored?

R: Well I think what we have currently is hard copies of AEFI’s reports and EPI report there is a certain place that indicates AEFI on monthly basis.

Let’s say in the beginning of the month if somebody had AEFI, you have to wait till the end of the month because our reports come monthly. So we usually traced them from our monthly reports here. Even if I am entering on dimes here and there is any year it means I will have to enter it on the dimes.

M: Do you people have any electronic way of storing AEFI data?

R: No unless it is in other component but AEFI to be specific we don’t have electronic data.

<Internals\\IDIs DHMT\\UEDI_11> - § 2 references coded [9.11% Coverage]

Reference 1 - 4.76% Coverage

M: How is an adverse event following immunization data stored?

R: We for now just have copies of these adverse events forms that are brought from the local level. We have copies here. Other copies are sent to the region and to the food and drugs board. But I think that we should create a template that we can simply put them in because these are leaflets they can easily fly over and you will be looking for it and you won’t get it but when we have a reporting tool but as and when we get we just keep updating so that anytime we need this type of information we open a folder and retrieve it.

Reference 2 - 4.35% Coverage

M: Do you people have an electronic way of storing data on adverse events following immunization?

R: I think that is what I just mentioned I think it is also depends on the individual programmes that comes in. some of the programmes that come provide electronic form to put in the data while you forward the hard copy to them but others don’t so if all could just have one electronic form irrespective of the programme that is running that when we record this adverse event then the data should be ported to that and we have it stored somewhere.

<Internals\\IDIs DHMT\\UEDI_12> - § 1 reference coded [2.28% Coverage]

Reference 1 - 2.28% Coverage

I: okay thank you how is the AEFI data stored?

P: mmhm like I mentioned, it is it is part of the EPI, a column is in it and so it is stored in a hard copy and then also we enter it into DHIMS so at the end of the year the hard copies are compiled into the annual report and its’ also stored in other way, so we have the hard copy, you have the soft copy and DHIMS.

<Internals\\IDIs DHMT\\VRDI_01> - § 1 reference coded [3.66% Coverage]

Reference 1 - 3.66% Coverage

I: that is great. How is AEFI data stored?

(3seconds)

P: As for that I can’t a,a, I, said there are eeeh reporting form, there are reporting forms for so how it is stored I know when it comes the disease control officer collate them and there should be a data somewhere to lay hand

I: and do you have electronic installation of it

P: Yes I think so but the eer cumulative something, as for that I can’t say much (flipping paper) I: what (Phone ringing) P: but there, there should be a stored data you know a, a data kept so far as (Phone ringing) AEFI is concern

<Internals\\IDIs DHMT\\VRDI_02> - § 1 reference coded [6.45% Coverage]

Reference 1 - 6.45% Coverage

I: Okay, that’s fine. Errm… How’s the data on adverse events following immunization stored? Is it stored on paper form or electronically?

P: Errr It’s stored on paper form and errm… Actually the disease control officer takes care of that one.

I: Okay!

P: ahaa so actually in fact, I’m not fully aware if it’s in the (inaudible) as a reaction (not sure) not seen a form.

<Internals\\IDIs DHMT\\VRDI_04> - § 1 reference coded [4.83% Coverage]

Reference 1 - 4.83% Coverage

I: Okay! How is the adverse event following immunization data stored? Is it paper form or it’s electronic?

P: It’s it’s we we we have the monthly reports, I think these are even some of the copies the immunization report. We enter it electronically as well.

I: Okay!

P: Because when we have the paper we enter into the district health information management system, the DHIMS.

I: Alright, errhm…

P: we we also have a form, that we fill…

I: At this level…?

P: Yh, at this level

I: Oh okay!

P: and then we submit to region and then…

I: Okay…

P: I remember national… I think somewhere either last year or early this year, I wasn’t, they didn’t meet me but they met my other colleague, around.

<Internals\\IDIs DHMT\\VRDI_05> - § 1 reference coded [6.58% Coverage]

Reference 1 - 6.58% Coverage

I: Alright thank you, then at your district alright, how do you store AEIF data

P: In fact (flipping papers) when eeem since I came I can’t remember eem any AEFI reported but will happen is that after is reported the disease control officer takes it, we have files for these things put it in eem put a copy then also forward a copy to region so I think

I: I had wanted to ask whether you do it in a paper form or electronic storage kind of

P: No it, it will be in a paper form, as I have, as I have said this is my fourth year in the district. I have not receive any AEFI eeeh, am not aware of any they’ve not reported any but I will think that it will be a paper eeeh , a for, a form is filled, is signed, a copy is send to region and a copy is kept.

<Internals\\IDIs DHMT\\VRDI_06> - § 1 reference coded [3.24% Coverage]

Reference 1 - 3.24% Coverage

I: erhmm… how’s the adverse event following immunization data stored? How do you store it?

P: yh! The disease control…

I: ...is it paper or…?

P: Yeah! It’s paper, it’s paper and then they key it into their…

<Internals\\IDIs DHMT\\VRDI_07> - § 1 reference coded [11.25% Coverage]

Reference 1 - 11.25% Coverage

I: ok. Ehmm, now again on the reporting, when the report comes, ehmm, how is the information… do have a storage system that you have in place at the district where you, you keep such information for record purposes?

P: Of err like errh adverse events?

I: Yes please.

P: you keep the result, the report in the file.

I: Ok, you mean a hard copy?

P: Yea,

I: ok.

P: because often is the… you will fill everything, is in hard copy, forward and I think the response too will be… in a, either sometime they will just inform you, call to inform you. Scarcely would you see hard copy anyway.

I: Ok, ok, ok. So ehmm, again with respect to the reporting, is it the case that…. Ok, what is the link between the district, the directorate and the Food and Drugs Authority as far as the AEFIs are concern?

P: Yea, I think they have a system in place and I think some years back, we would even be called to Ho, we will meet with the Food and Drug Board, they tell us when they are coming. So I think yearly, they do some kind of training where they ask for a particular staff who would be responsible for reporting on this adverse events following drug administration. So they go to those meetings and then they are given some training as how to make those reports. .

I: So you mean there is a focal person..

P: in the district.

I: in the district as far as the Food and Drugs Authority is concern?

P: Yea.

I: Ok. So how have being your working relation with those individuals?

P: Eei, normally they will report to you.

I: Ok.

P: And then before they send the form, I think you have to sign the form or something like that, before they even send it.

<Internals\\IDIs DHMT\\VRDI_08> - § 1 reference coded [3.52% Coverage]

Reference 1 - 3.52% Coverage

I: Ehmm, please when they report, how is the information stored?

P: Ooh! You know, is aaa… there is a numbering system, from the district, the number whether is one or the second in a year or something, is numbered before is send to Accra and a copy is kept in a file at the office here.

I: Ok, is it a hard copy?

P: Yes, yes hard copy. Yes, yes I think hard copy.

<Internals\\IDIs DHMT\\VRDI_13> - § 1 reference coded [5.54% Coverage]

Reference 1 - 5.54% Coverage

I: Ooh ok oh ok eere how is the AEFI data’s you, you receive stored?

P: The data for AEFI is normally on the EPI forms the immunization forms that is where the community health nurses or administrators at facility record,

I: Ooh ok.

P: Oo we depend on that data and conduct our follow up to find out eere what they have done on it.

I: Oh ok, ok eeere so eere is this data’s’ you collect on the paper stored on the paper form alone or electronically too they are stored?

P: Is on the paper and transferred to the DHIMS electronically [bleating] we enter it on to the DHIMS.

<Internals\\IDIs DHMT\\VRDI_14> - § 1 reference coded [5.03% Coverage]

Reference 1 - 5.03% Coverage

I: How is the AEFI data stored?

P: Data stored?

I: Yeah,

P: Mmm their data’s are stored like the way we store our data.

I: You mean?

P: On the hard copy,

I: Yeah.

P: Ennn hard copy l don’t know how the disease control,

I: Hard copy you mean you mean the paper form?

P: Ehenn,

I: So you don’t do electronic?

P: l don’t do it here so l think the disease control people are best to answers.

I: To answer ok ok

P: Mmmm that one, l don’t know after the hard copy whether they have the software to enter so that region will see or l don’t know.

<Internals\\IDIs DHMT\\VRDI_15> - § 2 references coded [3.90% Coverage]

Reference 1 - 2.22% Coverage

I: erre how is AEFI data stored?

P: When we take it from the facilities we keep a copy at the district level and then send a copy to the region level

Reference 2 - 1.68% Coverage

I: So you receive it in paper form or?

P: In paper form actually.

I: Ok so you don’t do electronic

P: No, no no

<Internals\\IDIs FDA\\GAFDA_01> - § 1 reference coded [1.45% Coverage]

Reference 1 - 1.45% Coverage

I: Okay, and please how is the AEFI data stored… data at your unit?

P: We have both hard and soft copies.

<Internals\\IDIs FDA\\GAFDA_02> - § 1 reference coded [4.40% Coverage]

Reference 1 - 4.40% Coverage

I: So now let’s move a little to the data itself. Please how is the data stored?

P: ‘erm’ at the moment the data is stored in ‘erhm’ an online data base that we call, the safety watch system. So the AEFI data is stored in the safety watch system.

I: so strictly electronic form?

P: It’s electronic form.

I: Okay

P: The paper hard copies are there, but the paper copies are translated into electronic form and that’s what we use and these reports are sent to WHO database [inaudible]

<Internals\\IDIs FDA\\NRFDA_01> - § 2 references coded [5.64% Coverage]

Reference 1 - 4.78% Coverage

P: you are welcome

I: how is the Adverse Event Following Immunization data store?

P: ok, to what I know is you know the public health workers after collating the take it to the disease control so that the also collate and then later on they, their transported or transferred to “ehhh” the headquarters of the National Pharmacovigillance center which is at the FDA, the FDA also collate it and then also send it, also store and then send it to the International Vigi-Base which is International rug monitoring center which is in Uppsala, Sweden such that if there is any severe or serious Adverse drug reaction that will be alerted to the WHO and then the WHO will also discuss with the manufacturing company and the will try to see if the can reformulate or do something such that in future this reaction can be minimized or averted.

Reference 2 - 0.86% Coverage

I: ok, is it stored with paper or is electronic?

P: I think we have both paper now and then electronic

I: ok

P: yes

I: ok, ok, thank you very much

<Internals\\IDIs FDA\\UEFDA_01> - § 2 references coded [7.48% Coverage]

Reference 1 - 5.11% Coverage

M: How do you store data of adverse events following Immunization?

R: We over here, when we pick the forms we look at the forms to see may be if there is hospitalisation and we have to make a follow ups and all those things. Apart from that when these forms are sent to Accra there is a data base where they keen in all these into the data base and a feedback is generated given to whoever send the report. Many at times the feedback report is send to us and we distribute them. If you have reported on an adverse event you need to know that yes it has been given attention and whatever the outcome is, has come out of the technical advisory committee is also communicated to us and we also communicate back to them.

Reference 2 - 2.37% Coverage

M: Is there an electronic way of storing your data?

R: No from my region we don’t store the report we forward it. But there are steps put in place so that we here can also keep it, so that it can get to the head quarter but as at now we send the hard copies of whatever we received to the headquarter where it is keyed electronically.

<Internals\\IDIs FDA\\VAFDA_01> - § 1 reference coded [1.84% Coverage]

Reference 1 - 1.84% Coverage

I: Ok. emmh again concerning the reporting, when you receive the report and for onward transmission to the national, do you, do you keep copies at the region and in what format do you save the records?

P: What we realized is, there are times that the evaluation as is carried out at the national level, there are…. there may be writings that are not clear, there may be information that may not be clear so they get back to us with the serial number. So if we don’t keep copies it will be difficult for us to find out,

I: to trace.

P: yah, to trace, so we keep copies on in a file. We make photo copies

I: ok

P: and then keep it in hard copy.

<Internals\\IDIs PROVIDERS\\GAPI_01> - § 1 reference coded [3.65% Coverage]

Reference 1 - 3.65% Coverage

I: How is the AEFI data stored?

R: Are you asking about the physical form

I: What you get

R: Usually in the adverse event reporting form, we have a copy that the facility will keep and one that is taken away and I think the facility keeps two copies.

<Internals\\IDIs PROVIDERS\\GAPI_02> - § 1 reference coded [2.51% Coverage]

Reference 1 - 2.51% Coverage

I: How is the data stored?

R: We have the DHIMS, hardcopies too and softcopies as well, I keep it on my pen drive and the desktop as well. We keep hardcopies in a file for safety measures.

<Internals\\IDIs PROVIDERS\\GAPI_03> - § 1 reference coded [2.86% Coverage]

Reference 1 - 2.86% Coverage

I: How is the data for the AEFI stored?

R: After filling the form we have monthly reports where we put the number of AEFI’s that one we enter on the computer and DHIMS.

<Internals\\IDIs PROVIDERS\\GAPI_04> - § 1 reference coded [1.38% Coverage]

Reference 1 - 1.38% Coverage

I: So how is the data stored?

R: We have DHIMS where we store it and we have the hard copy and keep it in a file.

<Internals\\IDIs PROVIDERS\\GAPI_05> - § 1 reference coded [1.63% Coverage]

Reference 1 - 1.63% Coverage

I: So how is the data stored?

R: We have a form after it is filled we photocopy and keep one but we don’t have a computerized system of storing the data.

<Internals\\IDIs PROVIDERS\\GAPI_06> - § 1 reference coded [2.39% Coverage]

Reference 1 - 2.39% Coverage

I: Please how is the AEFI data stored?

P: ‘Uhrr’ that one

I: is it in a paper form or a software?

P: No, no, we, we because of the vicinity we only communicated it to them that this the problem we have encountered ‘erhnn’.

<Internals\\IDIs PROVIDERS\\GAPI_07> - § 1 reference coded [1.98% Coverage]

Reference 1 - 1.98% Coverage

I: so having said that, so how is the AEFI data stored? Is it by paper form or electronically

P: no, DHIMS.

I: okay, so that’s software

P: yes

<Internals\\IDIs PROVIDERS\\GAPI_08> - § 1 reference coded [5.78% Coverage]

Reference 1 - 5.78% Coverage

I: Okay, so please how is the AEFI data stored?

P: We have a monthly data like a form that we fill every month then we capture the Adverse Reactions at the column, so after we have done that we send it to the records where they enter it into the district health management information system

I: Okay, so that’s like a software?

P: Yes a software that we keep a copy of the data

I: Do you store, do you store in paper form too

P: Yes we have a ( not concluded)

<Internals\\IDIs PROVIDERS\\GAPI_09> - § 1 reference coded [2.01% Coverage]

Reference 1 - 2.01% Coverage

I: how is the AEFI data stored? Is it in paper form or electronically?

P: okay. I know it’s in a paper form. If it’s electronic, that one I don’t know. [Door close n background]

<Internals\\IDIs PROVIDERS\\GAPI_10> - § 1 reference coded [1.73% Coverage]

Reference 1 - 1.73% Coverage

I: okay, alright! So ‘erhm’ how is the AEFI data stored? Is it in a paper form or electronic software or?

P: for our, for my health centre, it’s in the ‘erhm’, ‘erhm’ paper form. Yes! [Talking in the background]

<Internals\\IDIs PROVIDERS\\GAPI_11> - § 1 reference coded [3.69% Coverage]

Reference 1 - 3.69% Coverage

I: so please how is the AEFI data stored?

P: data stored?

I: yes!!

P: we have forms that we fill. So any case that you encounter, you fill that form and at the end of the month we report it to the district

I: so it’s usually a paper form or electronic form?

P: It’s a paper form

I: only

P: yeah

<Internals\\IDIs PROVIDERS\\NRPI_01> - § 1 reference coded [1.71% Coverage]

Reference 1 - 1.71% Coverage

I: So the AEFI what do you called it the AEFI cases the forms that you filled you compete how you do store them?

P: We have files.

<Internals\\IDIs PROVIDERS\\NRPI_02> - § 1 reference coded [4.48% Coverage]

Reference 1 - 4.48% Coverage

I: How is the data concerning AEFIs: how are they stored? When you like when you gather them how do you store them? Like the one that you encountered, the records regarding that one how was it store it?

P: aah ok we fill them, we have files so all our data and then our reports are filled.

I: So that means they stored in electronic form?

P: Not necessarily electronic but paper form.

<Internals\\IDIs PROVIDERS\\NRPI_03> - § 1 reference coded [2.98% Coverage]

Reference 1 - 2.98% Coverage

I: How is the adverse events following immunization data stored?

P: Usually .... I don't store ..... copies I just fill them and send them...... Emm because I fill one copy, I don't have a photocopier here where I can a photocopy, so just fill one copy and sent it to the next level I don't keep copies.

<Internals\\IDIs PROVIDERS\\NRPI_04> - § 1 reference coded [1.79% Coverage]

Reference 1 - 1.79% Coverage

I : How is the Adverse Events following immunization data stored?

P: Mmmm we just file it errh like the [Child crying]

I : Is it in paper or electronic?

P: In paper.

I : In paper?

P : In paper.

I : Okay

P: in Paper.

<Internals\\IDIs PROVIDERS\\NRPI_05> - § 1 reference coded [1.45% Coverage]

Reference 1 - 1.45% Coverage

I: How is the .. adverse events following immunization data stored?

P: Arrh okay, I don’t store it so I wouldn’t know. I don’t know.

I: The storage is it in paper form, or electronic form?

P: In paper form ....... yes in paper form.

<Internals\\IDIs PROVIDERS\\NRPI_06> - § 1 reference coded [1.86% Coverage]

Reference 1 - 1.86% Coverage

**I:** How is the AEFI data stored?

**P:** We just have our hard copies, when we errr…complete form or maybe write your details, you take a, a, a copy of it, send one and leave one here and so…

**I:** So in short, electronically or with hard paper, just the hard paper?

**P:** Yeah, yeah.

<Internals\\IDIs PROVIDERS\\NRPI_07> - § 1 reference coded [3.14% Coverage]

Reference 1 - 3.14% Coverage

**I:** Ok. How is AEFI data stored?

**P:** In our settings we don’t normally store any data, we don’t store any data, and we only take note of people who have come with adverse effects. That’s all

**I:** Is it stored in paper form?

**P:** Ok....yes

**I:** Or electronically?

**P:** no, we don’t store in electronic, we don’t have any electronic gadget. We normally have a book we rule in, that is the name column, we have the vaccines that was given and the adverse effect the person is complaining of... yea

<Internals\\IDIs PROVIDERS\\NRPI_08> - § 1 reference coded [2.43% Coverage]

Reference 1 - 2.43% Coverage

**I:** How is the AEFI data stored?

**P:** We store it by giving a copy to them and also keeping a copy in the file at the facility**:** Here we have a file for it and we keep a copy of the report in it

**I:** Is it electronically or is paper based?

**P:** It is paper based.

<Internals\\IDIs PROVIDERS\\NRPI_09> - § 1 reference coded [1.64% Coverage]

Reference 1 - 1.64% Coverage

I: How is the AEFI data stored?

P: we have a file that an arch file that we store it inside….talk cream

<Internals\\IDIs PROVIDERS\\NRPI_10> - § 1 reference coded [1.95% Coverage]

Reference 1 - 1.95% Coverage

I: How is the AEFI data stored?

P: we stored it on our hard copies in our AEFI files

<Internals\\IDIs PROVIDERS\\UEPI_01> - § 1 reference coded [3.15% Coverage]

Reference 1 - 3.15% Coverage

I: ok so errm like you mentioned earlier you said you have a folder you put some of these you document these things there so how is the AEFI data stored?

P: these cases their also stored you know we do monthly DHIMS entry

I: ok

P: so it’s entered into the DHIMS report

I: ok

P: so besides the folder will be there, its entered into the DHIMS report and then we will also have copies of the reports that we keep

I: ok so the DHIMS is it a soft copy or a hard copy I mean electronic

P: it’s a software electronic it’s a software this thing

I: ok

P: so that every report, all the various reports that we write on monthly basis are entered into the DHIMS

I: are there any other ways you store this data aside the DHIMS and you keeping copies are there any other ways?

P: no apart from that there is no any other way

<Internals\\IDIs PROVIDERS\\UEPI_02> - § 1 reference coded [2.81% Coverage]

Reference 1 - 2.81% Coverage

I: Ok. So how is the adverse event following immunization data stored?

P: Data store?

I: Ehm.

P: As we say, we have files for them. You can see this one, this is the file. So when we have any these things then you keep it in the file,

I: Ok. So aside the file, do you have any other…maybe electronically?

P: Yes. For the monthly vaccination report nu, that one when you go to DHIMS it’s there (Ok). So when you have a case and you report, you go to the DHIMS too you report it there.

I: Ok. Any other?

P: Well so far this is the only two places that we do, but if the district have any other way of doing it I can’t tell.

<Internals\\IDIs PROVIDERS\\UEPI_03> - § 1 reference coded [4.26% Coverage]

Reference 1 - 4.26% Coverage

I: ok … ok …. So how the adverse events following immunization data stored?

P: mmh ... the format as I said you will send a copy and then a copy is left with us so that’s how we it is happen so that maybe if you come and we say we have reported this what we have sent we should have a copy with ourselves so that it will be there that we have we have sent this we have we have recorded this adverse (inaudible) this case and then we have forwarded it to the next level but we have our copy so that they will also know how they will also store it to the highest level

I: ok … so meaning it’s in a paper form but do you store some too electronically

P: electronically … is now that we have a computer but we use not to have an electronical garget to store them so it’s just recently that we a common PC that we started putting our data there but not even all we are yet putting some of the other data there so

I: so have stored some of this AEFI data

P: not yet

<Internals\\IDIs PROVIDERS\\UEPI_04> - § 1 reference coded [4.10% Coverage]

Reference 1 - 4.10% Coverage

I: okay, my next question is on how you store your AEFI data?

R: so we have a booklet which is carbonated, so when we fill the form we tear the form and the carbonated one is left for the facility.

I: so do you store your data electronically?

R: no, but okay I think this last one, i think we did it through the internet but I don’t know whether I stored it or not.

<Internals\\IDIs PROVIDERS\\UEPI_05> - § 1 reference coded [3.92% Coverage]

Reference 1 - 3.92% Coverage

I: okay, thank you. How is the AEFI data stored?

P: I think the event we have to file it here then we have to send it to the district or where the the this thing our officers are. I don’t even know whether we still have the document about that because we used to file them in the immunization file but I don’t know whether it’s available.

I: how is the data captured?

P: you mean the data for the... that is by filling the forms, if we document and fill the forms, one is stored here one is sent out. That is how we capture the this thing for the report.

I: will it only be done on the paper or you store some electronically?

P: no as for the electronics storage you know we have not developed up to that level, because we don’t even have that facility here, we don’t have facility so we cannot talk of electronic one.

<Internals\\IDIs PROVIDERS\\UEPI_06> - § 1 reference coded [1.81% Coverage]

Reference 1 - 1.81% Coverage

I: How is the AEFI data stored?

P: come again.

I: How is the AEFI the data that you gather from your work how is the data stored?

P: mmhm, the storing of the data you know its suppose to go into our reporting form and our reporting forms when we prepare the forms the the reports we send a copy to them and leave a copy over here so we have a file that these copies can be inside or will be inside.

I: so it means you only keep hard copies?

P: yes hard copies because we don’t have a soft copy report
