# Supplementary material for: Optimising reporting of adverse events following immunisation by healthcare workers in Ghana: A qualitative study in four regions
Source: PLoS One. 2022 Dec 20;17(12):e0277197. doi: 10.1371/journal.pone.0277197 (PMC9767370; doi:10.1371/journal.pone.0277197)
Supplement: S1 Data — (ZIP) [file pone.0277197.s001.zip › Minimal data/S9 Suggestions to improve AEFI reporting_health workers.docx]

**Name:** 16. Suggestions to improve AEFI reporting_health workers

**Description:** This node contains all information on suggestions to improve AEFI reports.

<Internals\\IDI EPI\\GAEPI_01> - § 1 reference coded [12.96% Coverage]

Reference 1 - 12.96% Coverage

I: please doc can you let us know your suggestions in improving the reporting process of AEFIs

P: yeah, one I mean ‘erh’ would be to build capacity among the staff themselves not only the vaccinators but you know among the clinicians. If a clinician takes a history, [inaudible] and asked if a child comes maybe with some whatever condition because AEFI will come as a condition; whether it’s a fever or something, so if you then take vaccination history then I will also know maybe a temporal effect, maybe this thing came after the vaccination so clini, the clinicians must be trained, vaccination trained and then also general community awareness must be made so that at least if I bring my child for vaccination services and there is something happening after vaccination, I should be able to I mean [inaudible] no matter the way I see, I see it. It’s left to the system to I mean determine whether whatever is occurring is actually from the vaccine or not but you just I mean report. So I think ‘erh’ capacity building I mean on the part of the clinical and public health teams and also creating more community awareness on AEFIs I mean ‘erh’ will be also help

<Internals\\IDI RHMT\\GARI_01> - § 1 reference coded [6.16% Coverage]

Reference 1 - 6.16% Coverage

I: we wanted to know if, your suggestion for improving the process of reporting of AEFIs

P: yeah, one more thing too is ‘erh’ a lot of people do not report the [inaudible] I feel it is because they have to do some follow ups and they don’t have the means to do the follow ups . I do not know, I am not at the district level, I do not know what is pertaining there. You ask them, they cannot tell you this, that, that, that. But I have feel like they have to set some funds aside if there is anything, they use to do the follow up because if the person hasn’t got money to do the follow up, the person will sit down and we even instruct: take the ‘erh’ person to this side, go to this side, go to and then because of the follow ups some also refuse to even fill the form. They will not report because they know when they report, they will have to do the follow ups so they will not report at all.

<Internals\\IDIs FDA\\NRFDA_01> - § 1 reference coded [7.21% Coverage]

Reference 1 - 7.21% Coverage

P: you understand

I: ok, please let me know your suggestions for improving the process of reporting of Adverse Events Following Immunizations?

P: I think there should be constant touch or constant training with health, field workers or health workers who undertake this activity and then there should be public education also for the clients or the patients or the children or parents of the children to know, you know some of these things, so that they will also help in reporting

I: ok, ok. Is there any other you can add to that?

P: look, periodically, there should be seminars you know

I: ok

P: to both health workers and general public so that everybody will be aware that there is the need to report.

I: ok

P: so that we will be able to improve on the vaccines that are given

I: ok

P: I mean the quality and safety of the vaccines

I: ok

P: if we are able to report on this, future you know all the reactions can be averted because you will factor all these things in the manufacture of the vaccines such that may be we will not be able to eliminate them but will be able to reduce them drastically to the bearers minimum.

I: ok

P: such that you will take the vaccine may be you will not feel any reaction or effect

I: ok, thank you very much

<Internals\\IDIs PROVIDERS\\GAPI_01> - § 2 references coded [7.66% Coverage]

Reference 1 - 4.04% Coverage

I: How can you improve upon reporting among caregivers?

R: How to improve is to impress on the CWC in-charges or the attendants to explain to the mothers the reactions and what they should expect and when they come for their next visit they should ask so that they can record it.

Reference 2 - 3.62% Coverage

I: So how can we improve the reporting among health workers?

R: If we organize even a one day workshop, some few hours to talk about it, how important it is to do the reporting even if that is expected then they will know the importance of reporting.

<Internals\\IDIs PROVIDERS\\GAPI_02> - § 3 references coded [16.10% Coverage]

Reference 1 - 5.84% Coverage

I: How can we improve the reporting among care givers?

R:As I said documentation is key, we have to put down every information so at the end of the month we compile, sometimes to when we have a case and there is no doctor at first we have to report or even call the disease control officer on phone and we have any challenge and is not abreast with such challenges, we should communicate and if possible report to the appropriate quarters.

Reference 2 - 6.61% Coverage

I: So how can we improve reporting among the mothers?

R: Some of the mothers leave outside the communities, since they are far away in case we give them our contacts they can call us but if that fails then we have to tell them to that if after immunization something happens they should report to any nearest CWC but there is no CWC session there then they should go to the facility and send the child records card and inform the doctor about the history of the child instead of keeping the child.

Reference 3 - 3.65% Coverage

I: So how can we improve reporting among health workers?

R: The reporting format should be made available everywhere, every corner that gives out services so that it will help to have it on time in terms of reporting and also we should encourage them to document and submit.

<Internals\\IDIs PROVIDERS\\GAPI_03> - § 2 references coded [9.63% Coverage]

Reference 1 - 3.84% Coverage

I: So how we improve reporting by caregivers?

R: So continuous orientation and also educate the mothers about the signs and symptoms and tell in case you see this sign go to the hospital and report so those are the two issues.

Reference 2 - 5.79% Coverage

I: So how can we improve reporting among health workers?

R: Emmmm, orientation, reorientation on what to expect when you give this vaccines and then continuous quality data reporting so in their report, there is a form where they enter the number of AEFI cases they saw so if they are vigilant on that they will be able to get cases on that.

<Internals\\IDIs PROVIDERS\\GAPI_04> - § 2 references coded [15.90% Coverage]

Reference 1 - 9.69% Coverage

I: So how can we improve reporting among caregivers?

R: There should be something like posters so we post them at the immunization Centre so when the others come and read they will know that this is something that and they see any situation like that they would report it, because not all the nurses give such education, sometimes if they come to report the nurses will be like if I report they will know that am the one but there is poster there then when the mother can read and know about it, they would just report, they should also do announcement and they should talk about it because you might give the vaccine to like 5 people and just two might react to it so we need those information to find out if it’s from the vaccine or the children have some allergies so they can do research about it.

Reference 2 - 6.20% Coverage

I: so how can we improve reporting of AEFI among health workers?

R: That’s what am saying we should let them know the importance of adverse event following immunization and not to put blames that you have caused it at times the way they will talk to you , when you do such things next time when the nurse encounter that kind of problem he/she will just keep it to himself/herself, we make be part of the work so that if they encounter it they can report, they should not hide it so we can also help to improve it.

<Internals\\IDIs PROVIDERS\\GAPI_05> - § 2 references coded [17.82% Coverage]

Reference 1 - 10.84% Coverage

I: Can you give suggestions to improve reporting of AEFI?

R: I think that you know the work is becoming tedious for the nurses not tedious as such but plenty because they are doing family planning, immunization, home visits, outreaches in the communities so they tend to ignore AEFI’s so I am suggesting that for us to improve reporting lets attach an incentive to it, AFP I discussed with my director that after every mass immunization lets reserve some money and we should attach some amount to each case as an incentive so that we can improve on our case detection rate so with AEFI we can do the same because my nurses tell me that every time they give vaccination 90% of the children come out with fever and it is normal and not a serious AEFI, I tell them to fill the form and bring it but they don’t but if we say if any nurse an AEFI this amount will be given to them we will improve reporting. We always think they are not serious AEFI’s so we won’t report but will only report those that will bring us problems.

Reference 2 - 6.98% Coverage

I: So how can we improve reporting by the caregivers?

R: We can only improve by telling them the paracetamol syrup alone …… education is the key and the education should be shifted from give paracetamol syrup after your child has temperature and tell them that we want to improve upon the vaccines that are given to children so even if your child runs temperature you should report. So basically it’s all about education, education, education and sometimes too we can attach some incentive to it, you know Ghana when we say do something for free they won’t do it but if something is attached they will report so I think education and incentives are the best.

<Internals\\IDIs PROVIDERS\\GAPI_06> - § 2 references coded [18.83% Coverage]

Reference 1 - 10.36% Coverage

I: Okay, so what do you think we can do to improve the reporting of adverse events in general?

P: In general okay, ‘erhh’ when we want to improve upon it the first thing is, the recipient or the client have to get inside into whatever they are going to get. So health education is very, very necessary before we even ‘erh’ carry out that programme. Let them know what you are going to give to them the advantages and the disadvantages the consequences of it and then you give them, you give them raw picture of what they should expect after they have taken the immunization. For example if you have given the immunization on somebody’s shoulder and then the person after two or three days started to have, run ‘erh’ stomach you hardly relate it to maybe the immunization, so let the person know all the consequence or side effects of the immunization that you going to give to the person and then the person will appreciate it when he sees any he will come back and report.

Reference 2 - 8.47% Coverage

I: Thank you. But we also want to know to improve adverse event ‘erh’ following immunization reporting among ‘erh’ healthcare workers themselves?

P: ‘Erhmm’…okay, that, that one too will also…we, we cannot leave health education too aside. You have to give them some training, workshop on that and then some people think that ‘erh’ if he goes out to give immunization and there are complications it means that the healthcare worker himself didn’t administer it well so even if somebody comes back to report to him or her he feels reluctant to carry out, to carry it up to the next level because there will be lot question for him to answer so he will only sit on it and then hide it. But if he is made aware of the consequence by all means he will be able to report it to the right channel.

<Internals\\IDIs PROVIDERS\\GAPI_07> - § 2 references coded [10.04% Coverage]

Reference 1 - 4.10% Coverage

I: so how can we improve ‘erh’ AEFI reporting by caregivers?

P: by educating them so, ‘erh; education should still [inaudible] and then monitoring, supervision, yes. We should supervise them well. We shouldn’t leave them alone to do their own thing [door opens in background] [inaudible] monitoring.

Reference 2 - 5.94% Coverage

I: having said that, how can we improve AEFI reporting among healthcare workers?

P: [9 seconds] by giving them all the logistics they want

I: is that all?

P: and as I said, you do the supervision and check whether they have all the logistics. You the supervisor going, you should check whether they have it ‘erhm’ if they don’t have it you have to support them or get means of helping them to get it. Yes. [Door opens in background]

<Internals\\IDIs PROVIDERS\\GAPI_08> - § 2 references coded [15.14% Coverage]

Reference 1 - 8.33% Coverage

I: Having said that then how can we improve Adverse Events reporting by the caregivers?

P: So to improve we need to get a training we are, we, they need, the staff needs to be trained on how to identify and report Adverse Reaction I think that will, will [inaudible]

I: What about the caregivers how can we improve them, like improve the process of them reporting more.

P: The mothers?

I: Yeah

P: So we, we have to take it on ourselves to, to educate them we have to let them know the reactions, some of the Adverse Reactions that they may get from the immunization so that when they get it they may report to us

I: Is that all?

P: Yeah [inaudible] [4 seconds]

Reference 2 - 6.81% Coverage

I: So now we will like to know how we can improve ‘erh’ AEFI reporting among healthcare workers?

P: healthcare workers, I think we have to like I said the training is one of them. Then from there we have to be …, like to understand like understanding each other when it comes to cases like that maybe the person reporting it to the next level, the one who is at the next should be able to understand or should take measure that might not put off the, the person who is reporting so that next time you get the case they will be able to report it.

<Internals\\IDIs PROVIDERS\\GAPI_09> - § 2 references coded [7.10% Coverage]

Reference 1 - 2.24% Coverage

I: so if that’s the case ‘erh’, how can we improve the adverse event reporting by care givers?

P: continuous education during home visits and when they come for CWC as well and during outreach also

Reference 2 - 4.85% Coverage

I: so then, how can we improve AEFI reporting among health workers?

P: current refresher courses for all health workers where the forms will be out, you’ll be told when you see this, when you see that. The reason why I’m saying refresher courses is, some maybe on maternity leave, others may go to school. New staffs may come, so at least once a while when there is a refresher courses, there is update on whatever we are doing.

<Internals\\IDIs PROVIDERS\\GAPI_10> - § 3 references coded [15.68% Coverage]

Reference 1 - 6.36% Coverage

I: so with that, how can we improve AEFI reporting by caregivers?

P: oh! We have to intensify our health education; we have to conduct durbar and then our one on one counselling uhuh! And this is the case. We take care of ‘erh’ two hundred clients a day and we are how many? I’m not always around with them and they are three; one will be doing weighing, documentation, giving injection, so the counselling aspect, if we see that the one who is plotting the weight sees that oh the child is below the minus two SD score, that is when the one will say that uhuh! “Maame wo, wo, erh, erh, erh wo ba no, aden nti na ne weight no aye sei” but going to give a talk on AEFI, let’s be frank it, it, it will be difficult because we are not many. The staff strength is very low, very, very low

Reference 2 - 5.30% Coverage

I: okay, so we will also want to know what might be the reason why health care workers themselves who encounter AEFI are not reporting.

P: ‘mhm’ I oh! They are reporting [laughs]

I: could it be administrative issues, could it be staff issues or

P: oh, they are reporting

I: could it be logistics?

P: it could be staff issues, or it could be that, we would, would go for outreach, we are not having, we don’t go along with some of the forms, so you will see it and document it somewhere. You will come back; you are so tired you forget. ‘Uhuh’! So it’s also part

I: is that all?

P: that’s all. But my people they are doing well. They are reporting.

Reference 3 - 4.02% Coverage

I: oh okay. Having said that, we will like to know how we can improve the AEFI reporting among the health care workers

P: …’erhm’, then one, we should have refresher training for them, so that, they will know how important it is for us to report the AEFI and also ‘erhm’ anytime they are going for outreach, they should carry some of the forms along so that immediately they get to know this is AEFI they fill the forms. We do the following processes and then is being reported.

I: is that all?

<Internals\\IDIs PROVIDERS\\GAPI_11> - § 2 references coded [6.81% Coverage]

Reference 1 - 4.53% Coverage

I: so having said that, how can we improve AEFI reporting by care givers?

P: then we should intensify the education to them and the side effect or what can happen so when it gets in well maybe they can always come

I: is that all?

P: yeah! Then you tell them, reporting will help improve on the vaccines and other things so that the… will know we are doing a good job

Reference 2 - 2.28% Coverage

I: so now how can we improve this AEFI reporting among health care workers?

P: we have to intensify the training. Tell them the importance it can bring to us so that they will be alert.

<Internals\\IDIs PROVIDERS\\NRPI_01> - § 2 references coded [7.16% Coverage]

Reference 1 - 1.48% Coverage

I: so how can we improve AEFIs reporting by care givers?

P: *Caregivers* continue eeh education by health workers

Reference 2 - 5.68% Coverage

I: So how can we improve AEFI reporting among health care workers?

P: That is why I was saying we need capacity building health caregivers should be their capacity should be built through workshops and any means available.

I: Is that the only way that we can improve it?

P: Then to the parents caregivers or caregivers the parents like they should be given adequate education on AEFIs if not they do not see it necessary reporting.

<Internals\\IDIs PROVIDERS\\NRPI_03> - § 2 references coded [4.78% Coverage]

Reference 1 - 2.80% Coverage

I: Okay, how can we improve adverse events following immunization reporting by caregivers?

P: Is continue education, telling them to report anytime they have any complain or anything that arises after the immunization they should let us know....... Nhmm I think that is what we can do.

Reference 2 - 1.98% Coverage

I: How can we improve adverse events following immunization reporting among health care workers?

P: Emmm may be sensitization, more education and training.

I: Anything again?

P: Nhmm that is all

<Internals\\IDIs PROVIDERS\\NRPI_04> - § 2 references coded [8.19% Coverage]

Reference 1 - 4.53% Coverage

I : Okay we are almost getting to the end of our interaction. How can we improve adverse events following immunization by care givers? [Talking from the reception]

P : That one through what [talking from the reception] through sensitization, through our durbars and our meetings we can tell them, they should tell whenever someone has any, errr ehrn they inject, when they come for immunization and later they are having some reactions they should come to the facility. Emmm ..... Jus ehh that one thierr is just sensitization. [Talking from the reception]

Reference 2 - 3.66% Coverage

I : How can we improve Adverse Events reporting among health care workers?

P : That one like the training's will also do ehhh Sometime when you train staff, that what I say even though they night have knowledge but it awakens peoples ehher errr err interest in staff. For example like [ inaudible] like this, most at times some people knew but [ inaudible ] but when the training was going on people were reporting even they themselves said it ehherr.

<Internals\\IDIs PROVIDERS\\NRPI_05> - § 2 references coded [9.81% Coverage]

Reference 1 - 4.52% Coverage

I: Okay, so how can we improve …. Adverse events following immunization reporting by caregivers?

P: By *caregivers*?

I: Yes!

P: The awareness creation or education. We should always educate them and when they come we shouldn’t we the nurses too, we shouldn’t try to be hard on them. When they bring a child that we came and you injected my child and when I … got back home, they this was what was happening to my child, we have to receive them well attend to them ( inaudible) let them go and report it errrherrr so the awareness creation and our treatment to the caregivers. Errrherrr if you are treating them well and they also have the awareness and or they are educated on it, I think it will help ….. Improve .... the reporting of AEFI.

Reference 2 - 5.30% Coverage

I: How can we improve adverse events following immunization among health care workers?

P: Among health care work, health care workers, train us lets gon the go training …. Does the most important thing then you provide all the logistics needed for it …. To be reported. Errherr I think if we do this things.

I: Any other way to … improve the reporting?

P: Any other way to improve, I think we should also like at our level …. That one should be at our level if like we are all trained …. On it we can even take like select one of us …. To be the reporting officer to the Technical Officer within like the staff. Errrhrrr so like you responsible for this thing. So always make sure that like what you for TB and the rest nuu when it always ask for it and when you find one you report to the Technical Officer who will then take it from there to the district level. Yeah!

<Internals\\IDIs PROVIDERS\\NRPI_06> - § 2 references coded [11.17% Coverage]

Reference 1 - 7.42% Coverage

**I:** [Laughter] How can we improve AEFI reporting by caregivers? The caregivers, how can we improve upon how they report to us. **P:**  For…them, sometimes they don’t even know, so we have let them know, like I am saying, because of the staffing, these Enrolled Nurses and Staff Nurse, I have seen in a situation. Sometimes I go out and also ask. Maybe when this fellow comes to do the immunization here what did he tell you about the vaccines? And that they just came and they just came and collected their books, write and recorded and those that were having them they gave it back to them. We don’t let them know, err…the outcome of the vaccination or immunization. So the basic thing is we should let the caregivers know that the immunizations are not 100% secured, that when you give there will be nothing after the administration. So if we let them know that at least there will be something after it then they will be able to record and report. So that is basically the main thing. If caregivers are aware of the adverse effects then they will be able to report as to also know how to manage it.

Reference 2 - 3.75% Coverage

**I:**  We are almost done. How can we improve AEFI reporting among Healthcare workers? Among we the Health workers, how can we improve it? **P:**  The basic thing is I know that if there is this, it means that or maybe after immunization if a mother or caregiver comes to complain that this, this, this had happened then it might be AEFI then I have to also train my colleagues staff to know that, that is what goes in but we don’t do. That is the basic thing. If we were doing what we were supposed to do I don’t think this will have been any serious matter.

<Internals\\IDIs PROVIDERS\\NRPI_07> - § 2 references coded [11.41% Coverage]

Reference 1 - 6.03% Coverage

**I:** How can we improve AEFI reporting caregivers, by parents, how can we improve them?

**P:** I think err... we can improve that through education, yes. We need to give appropriate education when they are in for vaccination or for immunization. We have to let them know that, when they get home and they get any adverse effect they should return back to the hospital. That is the only way we can do it but then, the other plat form we can we can use is our meetings. We normally have quite a number of meetings, we have mother-to-mother support group and we also have father-to-father support group, we have attire based groups and then we durbars once a while we normally organize. We channel informations like this through these programmes where we educate the public that after immunization if they see any adverse effect that they think the child was not suffering from they should report it to the hospital and they will do appropriately.

Reference 2 - 5.38% Coverage

**I:** How can we improve AEFI reporting among health care workers?

**P:** Yes among health care workers there are quite a number of strategies we can put in place. First of all a training should be organized. I think we should have a training and then the staff can have a clear view of the whole is about and then an appropriate or an information is given regarding the reporting. Particularly how to do the reporting. The reporting format could be given out and a facilitator leads the staff through how to fill it and how the information should be tracked and where the form should be sent appropriately, yes. apart from that too we can also in our local level, in the facility level, we can encourage each other that in a case of adverse effect , whichever the case is , an appropriate reporting should be done to the right authority yea.

<Internals\\IDIs PROVIDERS\\NRPI_08> - § 2 references coded [7.07% Coverage]

Reference 1 - 4.45% Coverage

**I:**Ok.How can we improve AEFI reporting by caregivers?  **P:**  We have to encourage them after the immunization. We the health workers should encourage them. If you go and there is something happening come back, we will attend to you and when they come back, we should make sure we listen to them and have time for them and what is supposed to be done ,and if we can manage it at our level, we manage it and if we can’t we refer. So that, that person will get the correct care.

Reference 2 - 2.62% Coverage

I: How to improve the AEFI reporting among Healthcare workers.

P: Yeah, if like I said, we should let the training be there to sensitize them on that area to know that it is a serious part and also to monitor their activities and let them report. I think that is all that I have.

<Internals\\IDIs PROVIDERS\\NRPI_09> - § 2 references coded [11.34% Coverage]

Reference 1 - 6.78% Coverage

I: So how can we improve AEFI reporting by caregivers?

P: As I said when they come they tell us exactly what is wrong with the child and they must come back with the child weighing card for us to be able to check the date and the vaccine that were introduced to the child they must not replace they must not misplace the card they must not make it lost. If that one happen we cannot do that, so the mothers must take care of that

Reference 2 - 4.56% Coverage

I: How can we improve AEFI reporting among healthcare workers?

P: They should make sure that they will they should fill a form for each AEFI been it fever or any other thing they should report and they must make sure that all the reporting forms are readily there before the client come.

<Internals\\IDIs PROVIDERS\\NRPI_10> - § 2 references coded [6.15% Coverage]

Reference 1 - 2.13% Coverage

I: How can we improve AEFI reporting by caregivers?

P: to intensity our education to on AEFI

Reference 2 - 4.01% Coverage

I: How can we improve AEFI reporting among health workers?

P: by organizing training on AEFI with health workers and also providing the the basic logistics for reporting AEFI

<Internals\\IDIs PROVIDERS\\UEPI_02> - § 1 reference coded [3.23% Coverage]

Reference 1 - 3.23% Coverage

I: So how can we improve adverse events following immunization reporting by caregivers? How can we improve the reporting of from caregivers on adverse events?

P: So more counseling need to be done (Ok) on it. So when you talk to them, counsel them because it’s high time the education…you give somebody health education, the person has heard but he’ll not do it. So now we’ve moved from educating them to counseling – counseling one-on-one just as we’re sitting (yeah). Understand – when you talk to the person and the person understand, so when you tell them, they go, anything they come back and tell us (Ok). So as we’re doing that here, we’re getting the results – anything concerning that they come to report to us.

<Internals\\IDIs PROVIDERS\\UEPI_03> - § 2 references coded [5.99% Coverage]

Reference 1 - 2.54% Coverage

I: so how can we improve adverse events following immunization reporting by care givers?

P: mmm more counseling that’s effective counsel counseling then also getting volunteers the community volunteers involved so that when they also because their they volunteers are living with the mothers so it’s getting to the volunteer house may be shorter than coming to this comparing distance so when they can move to the volunteer and the volunteer can yeah the we have each other contact so they can easily contact us so that that reporting can be done faster and then effective

Reference 2 - 3.45% Coverage

I: so how can we improve the adverse events following immunization reporting among health care workers?

P: mmm that is why (inaudible) my earlier statement I said job training on for all staff. some of them they will say I wasn’t trained. and that’s why they will not report. but if in the job training you are trained, you know that if this particular if a client come with ABCD. this is what I should do I should not be waiting for maybe the focal person or the one that represented you for the training to do that job alone.so that one should be one, two will be … staff turning a new leaf to work.because if you are committed to your duty then there is no need this being a part of you shouldn’t carry it out. so these are the major ones commitment and then the job training.

<Internals\\IDIs PROVIDERS\\UEPI_04> - § 1 reference coded [4.34% Coverage]

Reference 1 - 4.34% Coverage

I: so in your own way what do you think can be done to improve AEFI reporting by caregivers?

R: we have to intensify our education to caregivers on ...a...a...a the side effects of every drug. We have let them understand that even though the treatment is for this but if there are other things cropping up they have to come back, so intensifying health education on adverse drug reactions.

<Internals\\IDIs PROVIDERS\\UEPI_05> - § 2 references coded [3.98% Coverage]

Reference 1 - 1.62% Coverage

I: okay thank u sir so how can we improve AEFI reporting by caregivers?

P: mmhm by educating them, if we educate the caregivers they will comply. They will understand and comply if we leave them and we don’t give them the education, I mean the more you educate them the more they will get to know if they are not educated how will they know

Reference 2 - 2.36% Coverage

I: alright sir, thank you. How can we improve AEFI reporting among health workers?

P: by educating all the health workers, involving all health workers when we are going in for immunization. We have to involve everybody to know that as far as vaccines are concern, there may be untold effects that will require us to report so we have to involve them. We should not exclude, we should not take a handful and leave a handful. Everybody must be involved since this is the work that all of us do.

<Internals\\IDIs PROVIDERS\\UEPI_06> - § 2 references coded [9.51% Coverage]

Reference 1 - 5.32% Coverage

I: Okay, thank u, how can we improve the reporting AEFI by caregivers?

P: pardon me again

I: how can we improve caregivers reporting AEFI should they find them on their wards or on their kids to health workers?

P: this is how, that is why maybe we should improve the communication skills with caretakers or care providers, you see when you are able to talk to all those you rendered services to them the caretakers or the caregivers, they will be able to report and why do we forget we forget because there is no column for advice given to somebody you understand, if there is a form and every parent you meet and give to the ward, then you document what advice you given to the person I don’t think you will be able to forget this thing but if it is oral, so you speak to them orally when everyone you get you speak to them orally, there is the tendency that you will forget of it and number two there should be enough funds too, if you give your number out to them, you can give your number out to all the caregivers, any adverse reaction even if they can’t come they should call you and you will come, so if this can happen why won’t it be improved, we do home visits why don’t they come and we do home visits because we think that we need to improve the system and some people naturally they don’t hospital environment so we have to go to the communities so we can still give them our numbers, ask them to call us when , anytime they should see an adverse reaction it will also help

Reference 2 - 4.19% Coverage

I: alright alright, so please how do you think we can we improve AEFI reporting among health workers?

P: okay supervision is also one of them, if I’m suppose to report to the directorate and they have given me forms, yes at the end of the day, the district should call me or even everyday in the evening, how many sub-districts do we have, we have about seven or eight sub-districts, in the evening the one in-charge the focal person at the district health directorate can also call, oooh today have you received any report on adverse reaction? Will you receive and say I have not receive, you cannot say that and so the district focal person will also need to do some work on that and so either by calling all those heading the sub-districts and so that they will also forward the reports to them even though I don’t have credit to call you, if you call me wont I give you understand (laughs again) so the focal person at the directorate so need to do some work and that’s where the improvement will come even though staff commitment too is also one I will think that we have to commitment on the part of the staff and supervision (claps hands) will have to move together.
