# Supplementary material for: Optimising reporting of adverse events following immunisation by healthcare workers in Ghana: A qualitative study in four regions
Source: PLoS One. 2022 Dec 20;17(12):e0277197. doi: 10.1371/journal.pone.0277197 (PMC9767370; doi:10.1371/journal.pone.0277197)
Supplement: S1 Data — (ZIP) [file pone.0277197.s001.zip › Minimal data/S4 Suggestions for training.docx]

**Name:** 10. Suggestions for training

**Description:** This node contains all information on suggestions for training on AEFIs

<Internals\\IDI RHMT\\NRRI_01> - § 1 reference coded [3.72% Coverage]

Reference 1 - 3.72% Coverage

P1: I think orientation as he mentioned, would have been the best way to get all the staff at the same level, I remember 2015 we went to “errrr”, Dodowa for a workshop was to cascade down to “errrr” the lower levels, it never came.

I: Ok ok.

P: “Yaah”, that was in 2015.

I: 2015.

P1: All the regions were assembled there, they took as through with ADFI to sensitize or orientate our staff, why is it that they are not reporting, we have talked “aaaaa” since 2015 and is still like that, we will talk talk with interviews everything and is still like that, we haven’t orientated, we haven’t put the staff down this, this, this, you see and if we we it was a nice program but it got stacked somewhere.

<Internals\\IDIs DHMT\\GADI_02> - § 1 reference coded [9.75% Coverage]

Reference 1 - 9.75% Coverage

I: Are there challenges regarding AEFI that we should know about?

R: Sometimes we don’t know where the forms are lying, documentation is very poor or may I say people keep moving the frontline workers keep changing their post and the junior ones may not be conversant that something like this exist, if there is a system where we do refresher training for the graduates but funds are not coming to do training. As at when if there is an issue then we do on the spot training and that will not be the best.

<Internals\\IDIs DHMT\\GADI_03> - § 1 reference coded [5.06% Coverage]

Reference 1 - 5.06% Coverage

I: Is there any other suggestions?

R: Definitely as new people come in, there is the need for reorientation or training for staff so I would say regular training on AEFI form because every now and then we get new staff coming in so I would say regular basis, there should be that orientation so that every point in time everyone is informed on the need to complete the forms.

<Internals\\IDIs DHMT\\GADI_07> - § 1 reference coded [13.75% Coverage]

Reference 1 - 13.75% Coverage

I: Please we would also like to know your suggestions for improving the process of reporting adverse events following immunization?

P: ‘erh’ improving the process is ‘erhm’ about continually reminding ‘erh’ staff and providing the necessary things for them to record it. For instance, they must have the forms, when they get the forms, they must understand the reporting and also I think refresher trainings will be fine. As we, as we receive new staff into the system, erh’ it should be one of the areas especially if they are immunization and so on, it should be one of the areas that we that we draw their attention to before they start work and the in all our reviews I think we should make it a point to ‘erhm’ drum it home ‘uhuh’. There are some people who may not be confident in filling forms yes. Filling forms is not easy ‘uhuh’ so there is the the need for us to improve on their confidence so that they can be bold to fill the forms and fill it well. That’s understanding how to fill the form, it means there should be a job aid, ‘hmm’? Attached to the form so that if they don’t understand any particular aspect of the areas, they can check from the job aid and fill it. So the form must go with what? A job aid. ‘uhuh’

<Internals\\IDIs DHMT\\GADI_11> - § 1 reference coded [12.08% Coverage]

Reference 1 - 12.08% Coverage

I: okay. So then we will like to know, from what you just said, will we would like to know the best way to provide training on AEFI

P: ‘hmm’ it can be at the district level

I: is that all?

P: I know that national will train, then region also will train [flips sheet of paper in background] then, will ‘erhm’ national will train people to train region, region also will train people to train district then district also will train their sub-districts

I: so we will like to know, how best do you think the training on AEFIs can be done?

P: how best?

I: yeah.

P: it’s should be done at the district level so that ‘erhm’ many…nurses or people who are working with the immunization at where there is ‘erh’, people are giving immunization, majority can be trained but if they want to train at the regional level it will be few people that will be trained.

<Internals\\IDIs DHMT\\GADI_14> - § 1 reference coded [2.99% Coverage]

Reference 1 - 2.99% Coverage

I: Please can you let us know how, like the best way to provide training on AEFI?

P: The best way to provide training [yawns] organization has implications, money so if those who are responsible *think* they have money that they can put in purposely for AEFI why not…

<Internals\\IDIs DHMT\\NRDI_02> - § 1 reference coded [2.45% Coverage]

Reference 1 - 2.45% Coverage

I: So I would like to know how best we best we can provide trainings on AEFIs to the healthcare workers.

P: I will I will suggest that the training eeh can *be* at the facility level -- it should be at the facility level

I: Facility based?

P: yeah facility based training.

<Internals\\IDIs DHMT\\UEDI_09> - § 2 references coded [11.09% Coverage]

Reference 1 - 6.24% Coverage

M: What would have been the best processes to provide trainings on AEFIs?

R: The best processes would be like any other workshops or training that is been organized and getting all those who are going to be working or concern are the best way. But I know even if it done, it is done to those directly giving the services like the facilities, whereas we here also need to be part of it. It is always limited number that is given like if they say they want to train five people. They would want to give priority to those who are directly involved in the process.

Reference 2 - 4.85% Coverage

M: You made mentioned of training downwards. What exactly do you mean by that?

R: Like I said if there is a training right now, they would just say they want five people from the district and we know that those who are directly involved are those at the facilities and they would be given priorities to be trained. But if it is like to train A, B, C and the district is involved at least they can send some people from the district too.

<Internals\\IDIs DHMT\\UEDI_10> - § 1 reference coded [3.42% Coverage]

Reference 1 - 3.42% Coverage

M: So how best can we provide AEFI training?

R: I think we need to take it as a main concern as other programmes because it is very important but it is much neglected and I think we should have a special training for that one. In the district here, we also need support even though we are technical officers we have knowledge. We need technical officers that are much based on AEFI’s to come and take us through.

<Internals\\IDIs DHMT\\UEDI_11> - § 1 reference coded [8.51% Coverage]

Reference 1 - 8.51% Coverage

M: Can you please let’s know your suggestions for improving the process of reporting adverse events following immunization?

R: My suggestion is continue training of all the health providers on adverse events following immunization from the district level down to the CHPS compound level. Also, refreshers training to some of the volunteers who are supporting us because some of these things are picked up by them when they come across them; they call the health staff to follow up. So I think continue refresher training of these events is very key. Number two provision of the necessary tools for these data to be collected and also we the health authority should also insist on every health care provider to try as much as to fill every adverse event and not to consider some as minor, others as severe because we don’t sit to wait for clients to come with severe adverse event like Stephen Johnson syndrome then we have failed. It is a dangerous adverse event that would occur to a child. We should consider all adverse events very important whether minor or severe.

<Internals\\IDIs DHMT\\UEDI_12> - § 2 references coded [5.84% Coverage]

Reference 1 - 3.63% Coverage

I: thank you, so with that can you please let me know how best to provide training on adverse events following immunization?

P: mmhm, okay I suggest programme organized within a period specifically on how to identify such, record, report and submit, formal training need to be done on it that will help us because you are not working alone, you are working with people, you receive from people and also submit, so downwards if they don’t have knowledge on it, it becomes a problem, you are not the directly with the people aaaha so those who have to submit to you need the training.

Reference 2 - 2.21% Coverage

I: will that be the only suggestion you want to give?

P: that may not be the only suggestion, the other best way is is is maybe its part of EPI, its part of any other programme, it can be added into it and then eerrh eerrh eerrh people will understand and other channels of just passing it through to the people to understand so relating it to the work.

<Internals\\IDIs DHMT\\VRDI_07> - § 2 references coded [17.06% Coverage]

Reference 1 - 13.80% Coverage

I: ok. Alright, please do you have any suggestions you think we, we… when adopted can help improve upon the reporting of AEEI?

P: AEFI from the district?

I: Yes

P: It all needs err regular training of the staff. Like I said initially, most staff are now on the field just about three years.

I: Ok.

P: And they have no …some have not experience such effects before,

I: Ok.

P: So we need lot of orientation and then training the newly posted staff about the reporting systems.

I: Ok.

P: We often do it but like human institution you may get one or two people who might have forgotten or maybe lazy,

I: ok.

P: Would not want to take up those task etc. Or maybe where I’m operating is a small place and nobody will border me so, you could just allow the reporting those things to go.

I: Ok.

P: Yea but so we need regular errh… At least if it’s yearly or once in every three as you come to brief them ehmm. . . This reporting, but I know is something that the disease control unit does.

I:ok, ok. Erhmm, you talked, you just talked about training, erhm, what kind of training do you think the staff would need with respect to this whole issue? How should the training be?

P: Yes, you see normally like Avemectin distribution, we will do training, you train volunteer etc, so at each of these trainings, you tell them about the… what to lookout for.

I: Ok.

P: The adverse events that could happen or side effects of those drugs. You give them out, so when they spot any such thing, they bring them to you. Not too long ago, about two months or a month back, we did these gifts, given err this-thing to errh… Yes, there were series, one or two reported cases of adverse effect that we have to send to the facility. And that was the place where the boy, my nutrition officer came to tell me that… Well you said such things are free but then, you know health insurance, you buy your drugs and do everything, so some of the facilities were not willing to just treat and let go of the money and things like that.

Reference 2 - 3.27% Coverage

I: ok. Now again about the training, should, should such training be facility base or at the district level or how should it be?

P: Errh, for a training like that, it will be time consuming moving from facility to facility but if you bring together at the district and then get training…

I: But given the fact that, errh we want to have an all –inclusive training, how is that possible?

P: Yea, you organise one for the health staff and then another for the volunteers. I: Ok.

<Internals\\IDIs DHMT\\VRDI_08> - § 1 reference coded [15.64% Coverage]

Reference 1 - 15.64% Coverage

I: By way of training, do you think there is something…

P: errh, by way of training, what it also means is that, until we continue to periodically update their knowledge on it, they will forget and think that oh is something of the past and they will never report on it. But if we continue on a regular basis, errh remind them. Because you know arrh a lot… the turnover is very high now. You train somebody today after three years, he going to school, next time he becomes a midwife. About immunization he has forgotten about it and new people have taken over, so if you don’t re-train those people, it means they will not be… they will be ignorant about these adverse events. So even whether. . . even if the midwife is there but know anything about it but is not directly involve in the immunization ,she may not have the opportunity report on any of, of these forms. And the new ones that have come, they don’t have any idea. They have not be sensitized on it, they don’t have any information on it. Meaning they will never report and now even people two and half years… even the three years will not even reach before they will be forcing that they will go to school. So we need periodic update on the information on this.

I: So what kind of training do you subscribe to? Is it facility level training or…?

P: well, I think it could be on the job training.

I: Ok.

P: On the job training so that… because is not… I don’t think is anything so difficult that the district level staff cannot do, but if we’ve just been empowered and reminded that it’s necessary to do this on a periodic basis, so that know body will forget about it.

<Internals\\IDIs DHMT\\VRDI_09> - § 1 reference coded [35.96% Coverage]

Reference 1 - 35.96% Coverage

I: ok

P: I don’t think so because the same staff who is doing the immunization, is the same staff who is reporting. So if he or she has the time to report on all vaccinations given, that too fills on the immunization form indicating AEFI, whether is serious or not serious,

I: ok

P: that should not be an issue.

I: ok,

P: nhm

I: ok. Ehmm, what about also looking at it from the point of ignorance on the part of the healthcare worker?’

P: ok, ignorance, well, I will… that I’m not very certain about because…

I: Yes, I say so because, you know the turn over that we have in the system,

P: nhm

I: where we have a lot of people going back to school,

P: yea

I: and then we have news crop of people in the system who may not be introduced to some of these things.

P: ok

I: couldn’t that also be a factor?

P: ok, ok, I think there is some truth in it.

I: ok.

P: nhm, that is also possible. Is also possible (*someone talking)* and so for, for me personally as the EPI coordinator, with our training needs for next year, which we were made to compile, I factored some of those things in

I: ok, ok,

P: so that if we have that opportunity by bring all of them together, we would go through immunization in general and would touch on all those things.

I: ok

P: but that notwithstanding, even with the little… the one that was reported, ehrr, last month as I was telling you, as I got there, I had to sensitize and educate the staff on the condition. We went through the filling of the forms,

I: ok

P: ehm, I think three, three CHNs who were present as at the time I went. We went through it again and so… that’s also part of it.

I: ok. Amhm, please how available are the forms at the facilities?

P: Yea, the forms are available. Only that sometimes because they don’t make use of it, they don’t become conversant with it and some people may not even know where they placed them.

I: ok.

P: As a matter of fact, when I was informed of that case, when I went they couldn’t… well they said they don’t have the form.

I: ok

P: so I gave them enough anyway. When I came back to… with our people who are here, we have an RCH here, I went to them to… they are a bit ehrr about six or seven or so. I took all of them through it again and then gave them the, the forms.

I: ok, ok. So couldn’t that also be part of the reasons why some may not be reporting?

P: Ehrr, as a matter of fact, the one who reported was not having a form.

I: ok.

P: ok but she reported.

I: ok

P: Now…

I: through a phone call?

P: Yes, this with errh, our people here, is over a month,

I: ok

P: is over a month when I had the interactions with them. I gave them the forms but since then errh, not one case was also reported.

I: ok,

P: ahaan, we had a lengthy discussion, they asked questions especially on this issue of fever because on the AEFI form, we have fever there.

I: yes

P: Ahaan! And so with them, fever is normal so long as especially PENTA is concern. And so would you have to be filling AEFI forms for every child that you give PENTA to? It was a concern, but we agreed that we all know fever errh, presents after PENTA.

I:yes.

P: Ahaan, and so that should not be so much of a big deal order than that, every child that receives PENTA, we are going to fill AEFI form for.

I: ok. What about the FDA, Food and Drugs Authority forms? Do you have any forms

P: yes

I: front their point?

P: yes.

I: So how is the communication like with respect to their forms in the district?

P: aaah, in the district here, (*someone talking*) as at the time I came as a matter of fact, we had my colleague here informed me. We had one errh, adverse event as a result of the Avemetin.

I: Ok.

P: Ahaan, so in collaboration with the FDA, I had to…. she had to fill the form, they had to take some sample to the national level and all that. But as at the time I came here, any direct link with FDA, I’m not even sure if they have any

I: ok

P: office in town.

I: Ok. But are those forms also available at the facilities?

P: The FDA forms?

I: Yes please.

I: That I can’t tell.

I: Ok.

P: I can’t tell because my… at my former place, it was the pharmacist who was handling the

I: FDA

P: yes, the FDA forms.

I: ok. and that isn’t the case over here?

P: errh, here, the pharmacist is gone to school but we have errh, a staff who is errh, taking care of the office.

I: Alright, ok, amhm, so when those forms are filled, to your desk for onward transmission, how are they stored?

P: We file them.

I: you file them?

P: yes.

I: hard copies?

P: Yes.

I: ok, ok.

P: But we also have the soft copy.

I: Ok. Ehmm, what suggestions do you have, that you think when adopted can help improve upon the reporting of AEFI?

P: Improve on the reporting of AEFI, I think that some, some of the, the erh, signs and symptoms should be re-looked at.

I: Ok.

P: Because as I mentioned in the case of fever for PENTA, it is the normal thing

I: ok

P: and so the staff who know that with PENTA, the child is going to have fever, and yet on our AEFI form, it is stated you have to errh, indicate if the child has fever and that also qualifies for AEFI. I: yes

P: it will mean that, they would be filling more than errh, necessary forms and so they will not even fill it at all. So long as it’s just fever.

I: yah

P: If it goes beyond fever and may be an abscess, errh, a lump, something of that sort, they may report it. A rash or anything of that sort, they may report but something like just fever, errh, pain. Ehmm, I foresee is one of the reasons why they don’t even report it.

I: ok, any other thing you think we can, we can do to improve upon the reporting?

P: ehm, it is also on my part as the district EPI focal person to be constantly reminding them of it. I think that is also one of the things that we could do.

I: Ok. Any other thing?

P: I think that is basically, what I think we should do.

I: Do you have any other thing you want to say with respect to AEFI?

P: With respect to AEFI?

I: Yes.

P: Ooh nothing more, nothing more.

<Internals\\IDIs FDA\\NRFDA_01> - § 2 references coded [7.15% Coverage]

Reference 1 - 2.58% Coverage

I: so how best can your department receive training for (AEFI) Adverse Events Following Immunization?

P: we are FDA, we closely, you know are stakeholders with EPI

I: ok

P: so what EPI do, we complement it, I hope you understand. So we want to make sure that the vaccines that are given out are safe you understand. So therefore we are a stakeholder here. So we will be very much interested in taking part in any training that EPI wants to turn out.

Reference 2 - 4.57% Coverage

I: ok. But what can you do in your capacity so that you, your department can get the training?

P: oh, periodically, as a regional head, am very much interested in the reporting of adverse drug reaction and then reporting of AEFIs, so periodically we do what we call in-training. So I have some slides I talk about the reporting of Adverse drug reactions without leaving AEFIs because the go hand in hand

I: ok

P: so of cause, almost every staff in our department knows something about the reporting of AEFIs and the periodically also every Monday we go to meeting at the Regional Health Directorate (RHD) where we talk about AEFIs, for instance some of the Disease Control Officers you know even reports to us we talk about some of the reactions people are getting and we all brainstorm on that.

<Internals\\IDIs FDA\\VAFDA_01> - § 2 references coded [1.84% Coverage]

Reference 1 - 0.68% Coverage

I: ok

P: and before we started, I was talking about if it could be…. even the training of the ICP could be taken as a continuous Professional Development and then given some credit hours which cause them to be force to be trained (Laughs).

Reference 2 - 1.16% Coverage

I: ok

P: They will force the system to train them because they will gain the errh, credit units for their…. for consideration for their errh, errh, promotions and all that. But for now as flat as it is, you have to go chasing people, you have to call and then if your time or your date actually falls on the date of another program, then it means you may lose them. You may not have them, I mean simply put.

<Internals\\IDIs PROVIDERS\\GAPI_01> - § 1 reference coded [10.76% Coverage]

Reference 1 - 10.76% Coverage

I: Can you give any suggestions to improve upon the training you had?

R: For reporting online, it means if a facility doesn’t have access to internet then the cant fill and submit the form, and we were made to understand that it is ok to bring the blue form, hopefully the one day training you realize you have to go through a whole lot, different aspects of it and even the internet was a problem it took us some time before we were able to go through but I think they should improve on the internet system because it’s not everyone who is internet inclined, the information was too much and if it could be made simpler like the blue form but honestly I haven’t touched it since I came back and I don’t even remember but since we had it its ok.

<Internals\\IDIs PROVIDERS\\GAPI_02> - § 1 reference coded [10.22% Coverage]

Reference 1 - 10.22% Coverage

I: Can you give me some suggestions to improve reporting among health workers?

R: Documentation is key, you work without documentation its like you haven’t worked, it affects the client as well as the reporting system you wouldn’t know what is happening in the system, we have to do good documentation we don’t have to overlook things, abnormal cases even if it is boil to know whether it is …… or common boil or AEFI, then we have to keep on with the workshops, refresher courses and those who are coming into the system even though they have been trained in school when they come out we have to add it to their orientation and then there should be proper supervision at the post, there should also be posters around on AEFI, it should be catchy so everybody will see.

<Internals\\IDIs PROVIDERS\\GAPI_03> - § 1 reference coded [4.76% Coverage]

Reference 1 - 4.76% Coverage

I: Are there any suggestions to improve upon trainings on AEFI?

R: Like I said we don’t wait for 5 years for training, every year if there are no trainings we have orientations, so things are not like new we know and its periodic and there no issues or challenges with trainings

<Internals\\IDIs PROVIDERS\\GAPI_04> - § 1 reference coded [10.39% Coverage]

Reference 1 - 10.39% Coverage

I: Can you give me some suggestions to improve training on AEFI?

R: If we can have one particular form because at times you go to the pharmacy they have different form, so if the form can be one, I don’t know if there is a difference but that will help and also there should be training for the nurses that when they give injection and they see those sign and symptoms they shouldn’t think it is normal, it is not when the person cannot walk that it is an AEFI but redness and abscess are all AEFI’s and they should report that is why we miss a lot of AEFI’s, you give ROTA and someone might run diahorea for three days and you think it is normal, if they know that they are to report such cases they should know that its not when the person is paralysed that its serious to report, they are supposed to know the signs and symptoms of the injections they give.

<Internals\\IDIs PROVIDERS\\GAPI_05> - § 1 reference coded [3.86% Coverage]

Reference 1 - 3.86% Coverage

I: What are some of the suggestions to improve training?

R: I think with the training too, at least every year there should be training on AEFI, it brings about awareness to the health workers that this thing is been taken seriously then they know the national level is involved but we sit for a long time without organizing a training the nurses will also relax.

<Internals\\IDIs PROVIDERS\\GAPI_07> - § 1 reference coded [7.33% Coverage]

Reference 1 - 7.33% Coverage

I: And please, we would like to know your suggestions to improving the training of adverse, the training on adverse events following immunization

P: oh! this tie I think we will train according to categories of staff, so that they can understand it well. Because, last time it was all jammed up. Questions cannot be… it’s like only those people [inaudible] the doctors, the physician or the [chuckled] the young, the junior ones cannot ask more questions but when it’s done according to the categories of staff, I think it will be okay.

<Internals\\IDIs PROVIDERS\\GAPI_08> - § 1 reference coded [17.03% Coverage]

Reference 1 - 17.03% Coverage

I: So now we will like to know how, can we improve the overall process of reporting Adverse Events Following Immunization?

P: Overall process?

I: Yeah.

P: We need to start with training. Training is the very, the basic thing that we need to do, then the ‘erhm’ system that we follow to report should also be improved, then we should also have feedbacks from the level , like where it,it, it is referred to we should have it back from them what was done and how the case went.

I: Is that all?

P: Then education too we have to make sure we educate mothers on identification, because they are supposed to report to us for us to take it from there. So if they are able to identify the, the effects, the side effects and they, they come then we will be able to report it.

I: Okay, so we are, we are almost done with the ‘erh’ ‘erh’ interview; before we go we will like to know if there is any other issue you will like us to know with regards to reporting of AEFI in general.

P: [talking in background] Yes is, we need training we have to get for, for example last, the whole last year we didn’t get any training on and we are having new staff who have no idea of ‘erhm’ Adverse Reactions. So if we are able to train more staffs to be able to identify and report the Adverse Reaction it will be able to help us improve the reporting system of adverse ‘erhm’ cases.

<Internals\\IDIs PROVIDERS\\GAPI_09> - § 1 reference coded [17.06% Coverage]

Reference 1 - 17.06% Coverage

I: we will also like to know your suggestions for improving the reporting of, ‘erh’ in training of adverse events following immunization

P: oh, I will say that, you see some of the things when you are in school you learn it but once you come to the field and start practicing, you may not even see some at all. So once you come to the field there and you see it…once a while there should be some refresher courses and when there is the change in vaccines we want earlier training rather than when the vaccines are being done, they will be rushing to do training. Sometime you will not be able to grasp everything and the shortage of vaccines too sometimes it’s a really a problem for us. You don’t even know the kind of explanations we should give it to the mothers. Now, there is another problem where we are to give the measles and the, let’s say ‘erh’…is it the meningitis? Yes, alongside with age, one and half year and we give the same measles with the yellow fever. At times you realise that when you open the measles, you give the yellow fever, and the, those coming for the, let’s say the Men-A ARE more than those coming for the measles and the yellow fever;so you realise that you have to open extra one and at the end of the day, there will be some wastage which we don’t like it. ‘eheh’ so if we could a specific one; if it’s measles *two* which will go with the ‘erhm’ the Men-A and we have the measles one which will go for the yellow fever. I think we will prefer that just to limit the wastage

<Internals\\IDIs PROVIDERS\\GAPI_11> - § 1 reference coded [4.53% Coverage]

Reference 1 - 4.53% Coverage

I: please we would like to know your suggestion to improve the training on AEFI [music playing in background]

P: [music playing in background] improve the training? Maybe it maybe increase maybe twice a year or because nurses come in and out. Some are going on transfer and new ones are coming. So if it can do it twice a year, it will be okay [tweeting in background]

<Internals\\IDIs PROVIDERS\\NRPI_01> - § 1 reference coded [1.74% Coverage]

Reference 1 - 1.74% Coverage

I: So could you please let us know how best we can provide you with training AEFIs?

P: Through workshops

I: Any other?

P: Coaching

<Internals\\IDIs PROVIDERS\\NRPI_02> - § 1 reference coded [4.26% Coverage]

Reference 1 - 4.26% Coverage

I: So any suggestions as to how we can improve the training?

P: Hmm I would suggest the because most health staff did not know the what goes into adverse events following immunization so if such a training would, so we could reminds each other of if you see the case how do you manage it. How do you conduct the case at our level before you refer that one could help.

<Internals\\IDIs PROVIDERS\\NRPI_06> - § 2 references coded [12.96% Coverage]

Reference 1 - 8.43% Coverage

**I:** Ok. Please let us know your suggestions for improving training on AEFI?

**P:** In fact, ah…my suggestion about improving the training on AEFI, I sitting here personally, at least there should be training held based on only AEFI, it is very important. You see, sometimes because of the nature of our work, they will come here because, I am not a clinician, even when they come to report to the clinicians instead of them to alert us to know as to this is what is happening, they would just take the opportunity and do what they want to do and the womens go. So sometimes that is why we don’t get, there are AEFI cases but because of the structure of the work the clinician will take the opportunity to do all these things. Even I have being telling them that whenever they see anything like that, they should find out even if women comes with a fever; ask whether in two or three days has she received any immunizations. That one can also be, especially the pricking and PCV will increase the temperature of the children but they don’t do so I will say, they should not limit it to only the field workers. The Staff nurses and the Enrolled nurses even the clinicians in particular should also be taken through AEFI and that’s going to help enough.

Reference 2 - 4.52% Coverage

**I:** Do you have any more to add to that? More suggestions

**P:**  Hmm, …more suggestions, I will also say, sometimes they also have to help us to also train our volunteers, because these days the volunteers if you even go to call them for anything they looking for money. If you say, thanks to them, it doesn’t, they don’t appreciate it. So you know they are in the community with the people so the volunteers actually they do … I have to, I have to even my volunteers here, I have to praise them for that, they are doing marvelously well, so if we get the chance and we get the things to train them at least I think I will help case enough to be able to cover such cases.

<Internals\\IDIs PROVIDERS\\NRPI_07> - § 1 reference coded [4.62% Coverage]

Reference 1 - 4.62% Coverage

**I:**  Please let us know your suggestion for improving on training on AEIFs.

**P:** Alright i think it will be very important to have an expect personnel who should organize maybe a brief training for, I should say all staffs because for CHIP compound, every staff there is involved in vaccination, almost every staff. It might be direct but to some extend they are. So if training can be organized for all the staff, weather a local one that will be done in the facility, or at the district level that will help a lot. And so for a few who have possibly had the training could extend it to the other staffs who haven’t gotten the opportunity so that it should be able to manage and also to be able to report appropriately.

<Internals\\IDIs PROVIDERS\\NRPI_08> - § 1 reference coded [4.78% Coverage]

Reference 1 - 4.78% Coverage

**I:**  Please let us know your suggestions for improving training on AEFI?

**P:**  So, we... there…, I have only one suggestion if like in every year at least we should have a training at least once on AEFIs so that, maybe the new staff that are coming here will also know something about it. But when it delays, maybe the new ones, they don’t know anything about it and they will get into contact with cases like that and will not know how and what to do, if the old ones are not around, it will never be reported.

<Internals\\IDIs PROVIDERS\\NRPI_09> - § 1 reference coded [6.04% Coverage]

Reference 1 - 6.04% Coverage

I: Please let us know your suggestions for improving training on AEFI

P: Nurses shouldn’t be lazy in filling the forms and 2, forms should be readily available, and then 3, the form must be a booklet from so that it will be carbonated, so that when you doing it its should get a copy to be onward submitted then all cases must be submit by investigated and treated free of charge.

<Internals\\IDIs PROVIDERS\\UEPI_01> - § 1 reference coded [1.52% Coverage]

Reference 1 - 1.52% Coverage

I: ok. Are there any other ways you think would be best to provide this training

P: yeah they can roll it …. they can roll it .. maybe they normally they can invite a cross section from the district then a cross section from the sub district then all the facilities within the sub district will come together then they will train them

I: ok

P: uhuh

I: any other?

P: yeah basically, basically

<Internals\\IDIs PROVIDERS\\UEPI_02> - § 1 reference coded [9.51% Coverage]

Reference 1 - 9.51% Coverage

I: So can you let us know your suggestions for improving training on adverse events following immunization?

P: Well…my suggestions, yes I’ll suggest that…maybe we just organize a training specifically for adverse effects following immunization (ok). With that…most of us…I’m saying most of us because I’m not the only CHO in Ghana here or Bongo, or this thing. Because there are certain things that maybe you may be looking at it that it’s not adverse effect, but it’s; so because of that you see that we’re always complaining we’re not reporting, we’re not reporting, we’re not reporting; because yes, you may see an adverse effect, but it’s like…it’s part of the side effects of the drug so let it be (Ok). You understand

I: Yeah.

P: Whether it’s an adverse effect, you understand. So I think if they just organize trainings specifically for that (Ok) see, they’ll go details (Ok) into it (Ok) so everybody will know much about it; so that when you see certain things you’ll not overlook them.

I: Any other suggestion?

P: Any other suggestion? So since Zokor here (laughs) it’s only Gambrongo (13:26) this facility that you’re coming. So I think it’ll be good you go to the other facilities so that they’ll all benefit from it (Ok). You understand; so maybe if you’re to organize training, the training shouldn’t be limited to the selected facilities that you’ve taken, but it should be…at least cover majority of the facilities so that it’ll help us [talk]…so that…, so as I was saying, the training shouldn’t be limited to the selected facilities (Ok), at least it should be, it should covered…should I say all the facilities (yeah), so that we all have much idea (Ok) on the adverse effects; so that it’ll help us…reduce or prevent them, or be able to detect them in time (Ok) so that action will be taken.

I: Any other you like to add?

P: Well, so far this is okay. If you’re able to do this, it’ll help community…it’ll help those who render services, especially those who are much involved in immunization…in Bongo, in this my facility, Bongo, and then what? Ghana, the whole nation (Ok). It’ll help us all.

<Internals\\IDIs PROVIDERS\\UEPI_03> - § 1 reference coded [2.76% Coverage]

Reference 1 - 2.76% Coverage

I: it was one day ok can you let us know you suggestions for improving training on adverse events following immunization

P: … mmm my suggestion is that sometimes it’s not necessary calling just few staff to a particular place to train but even on the job training so that when they come to the various facility level so that even those who are not even more into immunization can also get some idea about it so that wherever they find themselves when they experience something like that they won’t be “nino” in it mhm and that is where majority of even the staff can get to know much about it so job training is very crucial

<Internals\\IDIs PROVIDERS\\UEPI_04> - § 1 reference coded [3.05% Coverage]

Reference 1 - 3.05% Coverage

I: okay, so can you please let us know your suggestions for improving trainings on AEFI’s

R: {laughing} hmm, with the immunization I think maybe, if we make it like quarterly whereby they can always bring us together to refresh us on that and also on how to fill the form.

<Internals\\IDIs PROVIDERS\\UEPI_05> - § 2 references coded [9.00% Coverage]

Reference 1 - 6.27% Coverage

I: okay ,so can you please let us know your suggestions for improving trainings on AEFI’s.

P: well my piece of suggestions or advice will be tailored towards the type of vaccine that is to be given, because almost all drugs and vaccines have their different eerrh they have their different untoward effects or adverse effects so we cannot be on only one dogmatically depending on which somebody has ever given somebody ATS and the fellow collapse, so and you you may not expect that, that will happen, because ATS is also a vaccine that is given to children and then BCG we are told that HIV clients may react to BCG, I don’t know whether am right, so if a child is born and the child has gotten contracted the HIV from the mother and you don’t know and give and something happens, you may not know! So it is structured most of the training are structured the vaccine in question but I mean since we are dealing with multi vaccines now, it’s good to mention in the passing various adverse effects that may be expected in almost all the vaccines that we see and we give examples of them, because we even have five in one vaccine, so it is not only one it would be all the symptoms may be many and varied according to sort of vaccine the this thing, the vaccine that is given and the antibody immune system reactions.

Reference 2 - 2.73% Coverage

I: will there be more suggestions you would want us to know?

P: mmhm, well intermittently every because we are we continue to immunize, we would say that every quarter it would be good to talk about or to mention them so that they will be reminders, because if we are reporting quarterly, everything you know we come together quarterly to try to see what the performance of each district is so whilst we are talking about reconciling our immunization coverage’s and we also make mention of some of the pertinent adverse effects that we will may be meeting as a reminder.
