# Supplementary material for: Optimising reporting of adverse events following immunisation by healthcare workers in Ghana: A qualitative study in four regions
Source: PLoS One. 2022 Dec 20;17(12):e0277197. doi: 10.1371/journal.pone.0277197 (PMC9767370; doi:10.1371/journal.pone.0277197)
Supplement: S1 Data — (ZIP) [file pone.0277197.s001.zip › Minimal data/S1 How best to provide trainings.docx]

**Name:** 02. How best to provide trainings

**Description:** This node contains all information on the best ways to train health workers on AEFI reporting

<Internals\\IDI RHMT\\NRRI_02> - § 2 references coded [9.52% Coverage]

Reference 1 - 6.56% Coverage

I: So how best, please let me know how best to provide training on AEFIs?

P: You see, this question not very good for my level or for any unit because am not the, the person or my unit is not the unit that coordinates Adverse effects. It is the EPI expanded program on immunization or surveillance unit that actually coordinates the training of this and if they are coordinating then all RCH staff or maternal and child health staff because we are directly linked to vaccination because the community health nurses we immunize and vaccinate “err” children and mothers at that level so if it is, it is not me who coordinates but the coordinate and either invite district staff like those people I have mentioned, the staff involve in immunization, the disease control officers, the community health nurses especially all those who matter in vaccination is the EPI or surveillance unit at at at regional level the coordinate the training of these people “ahaaa”, so that is why am not able to say much about training. “Ahaaa”, they are those who coordinate and I know series of trainings has been organized “ahaa” but I can know when and how many times because I was not the person who was coordinating it.

Reference 2 - 2.96% Coverage

I: Thank you very much.

P: Yes, And so sometimes when these trainings are on and then you are aware of it, you can decide to go and be an observer to listen. In times pass, public health nurses were invited at the national level together with regional teams to be three, that is why I said some time ago I enjoyed the training I was invited and I was among the regional team that went for the training years back but of late, it is the EPI and surveillance units that coordinates trainings and I know trainings have been going on this issue.

<Internals\\IDI RHMT\\UERI_01> - § 1 reference coded [15.89% Coverage]

Reference 1 - 15.89% Coverage

M: can you let us know how best to provide training on adverse events following immunization?

R: well I think on the job training is the best where you go with the forms and let me say simulation exercises and use that to teach the staff how to do it. For instance, we can do a role play, somebody comes in as somebody who is coming to report an adverse event and then we ask the staff to go and complete the form and go through the process because sometimes you talk, talk and people go home and forget when they do the activity I think it will help them to remember.

M: what do you mean by simulation exercises?

R: what I mean is that we will pause I don’t know what to say again we will pretend that somebody has had an immunization and he has adverse events and has come to report. So we all sit around and then we see how the service provider will do the completion of the form and what they would do next so that if there are gaps then we can fill them in and that is what I mean.

M: in the health delivery system we have care givers and health care workers. What reasons might prevent a care giver to report an adverse event following immunization?

R: in the first place if the care giver was not told that if anything untoward happens they should report then they wouldn’t even know that they should report. One other thing I think is lack of knowledge both on the side of the health care provider and the care giver because if the health care provider doesn’t know that there is something like that an adverse event which should be reported or communicated that to the care giver then they will not report.

<Internals\\IDI RHMT\\VRRI_01> - § 1 reference coded [16.01% Coverage]

Reference 1 - 16.01% Coverage

I: ok

P: because they not trained on it.

I: ok

P: Because they are not trained on it. So I think errh there should be a proper training for all the people that do the vaccinations so that they would be able to report.

I: Ok.

P: Ahaan, because the system is there, the reporting from facility to district, errh sub district to district to region, national. The system is there but sometime how to monitor it because some, some, may be like the clear definition, what to report, some of them may also not be too sure on some of the things to report. So I feel errh a lack of tracing of the staff that actually do the vaccinations on AEFI is one of the reasons we having how reporting rate.

I: ok. Any other please?

P: Emmh and other ones too, is like maybe like the communication aspect.

I: ok

P: That to sensitize caregivers about the AEFIs because all you know, some of the caregivers too if there is AEFI all that they do is…., if is even severe, and they have not reported they feel ok; I have gone to the vaccination session and this happen next time I will not send my child. Because they also don’t know that may be this and this are the things I have to report on.

So the second thing I will think is errh, we should also errh think of sensitizing the caregivers.

I: ok.

P: Ahaan on the AEFI. When they know that, ooh these things can happen, these ones are expected, though they talk to them at the clinics but I don’t think those education is errh, very, very errh, enough or adequate,

I: ok

P: so there should be more or we should step up education on the AEFIs to caregivers.

I: ok. Emmh, does your unit

P: yes

I: have in place the mechanism to monitor AEFI cases in the region?

P: errh, yes, we have a mechanism but hern, with challenges.

I: ok.

P: nhmm, we have the mechanism with challenges.

I: What are they please?

P: Because every, every year, we put in plans for monitoring

I: ok

P: ahaan, but sometimes you, the times you would want to go for the monitoring, the funds are not available for you to go and do the monitoring. So I think that’s one of the challenges.

I: ok. Ammh, could that also be a factor, contributing factor to the low reporting from, from the facilities?

P: That we are not able to do monitoring?

I: yes

P: Errh, yes it’s also part of it.

I: Ok

P: Because if you visit the district that may be you have to get at least one or about 10, a district is supposed to get about 10 and you report only one or two, what are the challenges? If we also visit them and they also look at the sub-district, may be this sub-district you are suppose get at least three in the year. So what are their Challenges? I think the supportive supervision also plays a role.

<Internals\\IDI RHMT\\VRRI_02> - § 1 reference coded [6.74% Coverage]

Reference 1 - 6.74% Coverage

I: In terms of training, what kind of training do you think will be effective for them? How should the training be like?

P: Errh, we have a lot of forms in the system, so first the availability of the form is crucial. If they are there, then we need to go through this thing for them regularly.

I: Ok.

P: Emmh, in the DIMTHS, we have this thing, so we also look at some of these things through the DIMTHS and then we call them. That’s may be through phone call, we inform them that you are supposed to do this if you have done it will go against you. We alert them and then we can also have a league table for all the districts, as you report, we record.

I: ok

P: At the end of the day can motivate them by giving some prices to encourage others also to follow suit.

I: Ok.

P: Yah.

<Internals\\IDIs DHMT\\GADI_05> - § 1 reference coded [19.40% Coverage]

Reference 1 - 19.40% Coverage

I: Can you give some suggestions to improve training?

R: Ermm at the district level I don’t know the in-service coordinator but at the facility level I know the in- service coordinator so it’s like on our appraisal it is stated your weaknesses and your strength to upgrade yourself so when they see your appraisal forms yearly they will see the needs of the staff and will know whether the person needs training or not in the unit, pick it out so when there is a training they include you but unfortunately when there is a training they select the people to go and the category of staff to involve so if you don’t read on your own, do intense search and all that you might not know what is going on. Those who get the opportunity to go for the training when they come they disseminate the information, they organize some small in-service training for those who were not able to attend I think when that is done it might help.

<Internals\\IDIs DHMT\\GADI_09> - § 1 reference coded [4.69% Coverage]

Reference 1 - 4.69% Coverage

I: please how will, we would like to know the best way to provide trainings on adverse events?

P: normally ‘erh’ I think may be when you do it on the site ‘erh’ on the site training I mean we go to the facility and then train. But once a while we also bring them, we can, we can train the leaders in may be a formal way and then the, the staff we go to them, ‘hmm’ during your supervision you sit them down and then talk to them about it. Because it’s only when you get them in smaller’erh’ numbers that they will really understand than the whole sale one. They come and sit here, talk, talk, talk and then they go and do a different thing hmm

<Internals\\IDIs DHMT\\GADI_10> - § 2 references coded [10.28% Coverage]

Reference 1 - 4.55% Coverage

I: So we will like to know, why have you not received any trainings?

P: We, we receiving the trainings or we conducting the trainings?

I: Like ‘erhm’ receiving trainings

P: Well with receiving the trainings, I because then with receiving the trainings, it will have to come from the top so with that I cannot really tell. But I’m sure that ‘erhm’ probably if they have a schedule they would probably according to districts so if there is a schedule like that definitely it will come up or if they should come round and ‘erhm’ probably train us on some something concerning vaccinations, Immunisations definitely they will chip it in. yeah!

Reference 2 - 5.73% Coverage

I: So please having said that we will like to know the best way to providing training on AEFIs

P: Ok, for me, if ‘erhm’ I think that it can be provided in ‘erhm’ in two layers but one at the district level. You see! Where we bring those the in-charges of the sub-districts, the public health nurses and also ‘erhm’ ok obviously the district director and the nurses at the upper (inaudible) and then when we finish we would now go down because we realise that we have a lot of CHNs we can’t kind of lump them all together. So if it’s done at two levels ‘erhm’ it is better. Then I think we can reach them better because they are the ones providing the service “ahaa”, so it will be more detailed in that training; that’s the training “erhm” that would involve the CHNs or those who, who do the vaccinations.

<Internals\\IDIs DHMT\\NRDI_02> - § 1 reference coded [2.45% Coverage]

Reference 1 - 2.45% Coverage

I: So I would like to know how best we best we can provide trainings on AEFIs to the healthcare workers.

P: I will I will suggest that the training eeh can *be* at the facility level -- it should be at the facility level

I: Facility based?

P: yeah facility based training.

<Internals\\IDIs DHMT\\NRDI_04> - § 1 reference coded [3.42% Coverage]

Reference 1 - 3.42% Coverage

I: So how best can …. Emmm adverse events following immunization training be given?

P: ( inaudible) for, for IEA for this particular activity it can happen at any point in time. IE and C, IEA it can happen, can happen even if you are the best nurse, you may give an injection and something can happen so it needs….. well the the staff need to be updated and also need to oriented especially when they are coming new. So those who are coming new to join the system to be train on that one those who already there if there are new things they need to be updated. So refresher training for existing staff and then new ones should also be trained. These are the things I think we can do the training.

<Internals\\IDIs DHMT\\NRDI_07> - § 1 reference coded [2.06% Coverage]

Reference 1 - 2.06% Coverage

**I:** Please let me know how best to provide training on AEFI?

**P:** As for best training at times, the best I should say is that for every surveillance activity or immunization, there is a RPI Programme, and these should be a component of AEFI. Aside that then they can, they should be a formalized AEFI training. But however, all other surveillance hands were on EPI Programmes there should be an AEFI training.

<Internals\\IDIs DHMT\\NRDI_14> - § 1 reference coded [5.21% Coverage]

Reference 1 - 5.21% Coverage

I: If yes please let know how best to provide training on AEFI

P: *am* when we are doing training on AEFI you should let the people know what AEFI is so that when they see it they should handling it as appropriate and also before they even go out for the field they also need at least to carry something that can when even it comes they can be able to start there before they bring the person to the health facility excuse me [] phone call 15 seconds

<Internals\\IDIs DHMT\\NRDI_15> - § 1 reference coded [2.19% Coverage]

Reference 1 - 2.19% Coverage

I: let us know how best to provide training on AEFI

P: Well how best than I can just say it should just be a topic on it

<Internals\\IDIs DHMT\\UEDI_01> - § 1 reference coded [7.22% Coverage]

Reference 1 - 7.22% Coverage

I: so what might, can you let us know how best to provide trainings on adverse events following immunization.

P: aaa, as in, is it the processes or, what goes into the training?

I: yess everything that has to do with the training on adverse events

P: I think, the process of reporting, yes, should be focused on more because me myself I didn’t really know the process of reporting because I, I never witnessed one and for all you know there are a lot of community health nurses whom I reported into the service before them (ok)who have also never witnessed them before (ok) so right now if they, they, they go for outreach and they get such a case(I- COUGHS) they will keep it to themselves ( ok), because they don’t know how to report it (report it).They will probably pick a phone and call the district disease control officer that this is what has happened but to report it formally to, that way they won’t do it

<Internals\\IDIs DHMT\\UEDI_02> - § 1 reference coded [6.36% Coverage]

Reference 1 - 6.36% Coverage

I: Ok. So can you please let us know how best to provide training adverse events following immunization? You were talking about the funding. Can you please let us know how best you can provide training on adverse events following immunization?

R: Yeah, as I said eh… you know, the thinking is that when it comes to adverse reactions people think that they’re all expected (Umm). You see? But as I said earlier, some of them we might not know about them. So we’ll need to do it on regular basis (Ok). Even those of us who are trained will need some refresher training quarterly. And then eh we’ll also try to train people on the job to let them know that they have to document everything that happens after immunizations. Because the perception may be that they are expected, so they won’t have children (yeah). But that is not good because you realize it’s not everybody that will get these adverse reactions (yeah) when they’re all given the vaccine.

<Internals\\IDIs DHMT\\UEDI_08> - § 1 reference coded [4.67% Coverage]

Reference 1 - 4.67% Coverage

I: okay so since you have not had any training can you tell us in your view how best to improve training of AEFI’s to you?

P: with that eerrh, we have eerrh district plan that everybody brings what he/she intended doing in a year so what we do is that we add those things, give it to director then he will lobby for funds from assembly to see if they can assist us to train our staff on those things. If they are not involved to, what we do is we report to our superior at the region if a training can be done on such things. Thus what we usually do.

<Internals\\IDIs DHMT\\UEDI_09> - § 2 references coded [11.09% Coverage]

Reference 1 - 6.24% Coverage

M: What would have been the best processes to provide trainings on AEFIs?

R: The best processes would be like any other workshops or training that is been organized and getting all those who are going to be working or concern are the best way. But I know even if it done, it is done to those directly giving the services like the facilities, whereas we here also need to be part of it. It is always limited number that is given like if they say they want to train five people. They would want to give priority to those who are directly involved in the process.

Reference 2 - 4.85% Coverage

M: You made mentioned of training downwards. What exactly do you mean by that?

R: Like I said if there is a training right now, they would just say they want five people from the district and we know that those who are directly involved are those at the facilities and they would be given priorities to be trained. But if it is like to train A, B, C and the district is involved at least they can send some people from the district too.

<Internals\\IDIs DHMT\\UEDI_10> - § 1 reference coded [3.42% Coverage]

Reference 1 - 3.42% Coverage

M: So how best can we provide AEFI training?

R: I think we need to take it as a main concern as other programmes because it is very important but it is much neglected and I think we should have a special training for that one. In the district here, we also need support even though we are technical officers we have knowledge. We need technical officers that are much based on AEFI’s to come and take us through.

<Internals\\IDIs DHMT\\UEDI_12> - § 2 references coded [5.84% Coverage]

Reference 1 - 3.63% Coverage

I: thank you, so with that can you please let me know how best to provide training on adverse events following immunization?

P: mmhm, okay I suggest programme organized within a period specifically on how to identify such, record, report and submit, formal training need to be done on it that will help us because you are not working alone, you are working with people, you receive from people and also submit, so downwards if they don’t have knowledge on it, it becomes a problem, you are not the directly with the people aaaha so those who have to submit to you need the training.

Reference 2 - 2.21% Coverage

I: will that be the only suggestion you want to give?

P: that may not be the only suggestion, the other best way is is is maybe its part of EPI, its part of any other programme, it can be added into it and then eerrh eerrh eerrh people will understand and other channels of just passing it through to the people to understand so relating it to the work.

<Internals\\IDIs DHMT\\VRDI_07> - § 1 reference coded [3.27% Coverage]

Reference 1 - 3.27% Coverage

I: ok. Now again about the training, should, should such training be facility base or at the district level or how should it be?

P: Errh, for a training like that, it will be time consuming moving from facility to facility but if you bring together at the district and then get training…

I: But given the fact that, errh we want to have an all –inclusive training, how is that possible?

P: Yea, you organise one for the health staff and then another for the volunteers. I: Ok.

<Internals\\IDIs DHMT\\VRDI_12> - § 1 reference coded [11.71% Coverage]

Reference 1 - 11.71% Coverage

I: ok. Alright you mentioned of training for the community health nurses, what kind of training do you errh, will you subscribe to for them? How should the training be like? Should it be facility based or?

P: Ooh yes, no, no, they should be brought to the district, all them because when you do it facility base, sometime somebody might have another issue to bring out but it might not be peculiar to that facility, so they will not bring it out but if we bring then all together, so this one will bring a case, another person will bring another case, so we all thrush it out and that will go down well than the facility base. So it should be district base. Maybe in batches so that all of will be trained because what we experience is, when you train one person, the other person will say aarh they’ve trained you, so you have to do it. ahaan, and in the other case, somebody will say aan, ok, the person will go back and what she or he has been taught, she will not teach the others so the information will be left with him alone so we need to… in batches. It will be errh, I mean one day refresher or training session not elaborate days, so many days. One person will come, other person from all the sub-districts, all the facilities will, I mean be trained. All the members of the facility or the CHNs will be trained.

<Internals\\IDIs DHMT\\VRDI_15> - § 1 reference coded [7.04% Coverage]

Reference 1 - 7.04% Coverage

I: Emm have you, have you not receive any training please lets us know why, how best to provide training on AEFI?

P: Yeah we have not received any training, any formal training so if, if we are ready to, to to have any training if there’s a training, an information for training we are ready to actually take it

I: So what, what am asking again is please let us know how best to provide on AEFI like for you people

P: We will lease with region

I: Ok

P: Mhm we lease with region

<Internals\\IDIs FDA\\NRFDA_01> - § 2 references coded [4.55% Coverage]

Reference 1 - 2.31% Coverage

I: ok. But what can you do in your capacity so that you, your department can get the training?

P: oh, periodically, as a regional head, am very much interested in the reporting of adverse drug reaction and then reporting of AEFIs, so periodically we do what we call in-training. So I have some slides I talk about the reporting of Adverse drug reactions without leaving AEFIs because the go hand in hand

Reference 2 - 2.24% Coverage

I: ok

P: so of cause, almost every staff in our department knows something about the reporting of AEFIs and the periodically also every Monday we go to meeting at the Regional Health Directorate (RHD) where we talk about AEFIs, for instance some of the Disease Control Officers you know even reports to us we talk about some of the reactions people are getting and we all brainstorm on that.

<Internals\\IDIs FDA\\VAFDA_01> - § 1 reference coded [2.83% Coverage]

Reference 1 - 2.83% Coverage

I: ok

P: Yes. There’s being times that we’ve tried doing programs and it’s gone that way. So looking at it, I believe procedurally and structurally, some recognition will have to be put in the system. Likewise even FDA. If somebody is an ICP because we also… we are also contact persons, we are the focal

I: persons

P: yes. Attritions and transfers come in, I mean who then is designated to take the place? if we could consider… we should have some structure apart from the procedures and then protocols that we have, we should have a human resource structure to actually look at this and then… For instance, FDA is also controlling blood and blood products, who is the ICP at the blood bank? It supposed to be that biomedical guy. Erh, we’ve got to also look at errh, what sort of training, will it be the same as the medication? Is not. It will be a different thing, biologicals. So the trainings will be different. So I believe there should be a human resource structure to take care of this.

<Internals\\IDIs PROVIDERS\\GAPI_01> - § 1 reference coded [10.76% Coverage]

Reference 1 - 10.76% Coverage

I: Can you give any suggestions to improve upon the training you had?

R: For reporting online, it means if a facility doesn’t have access to internet then the cant fill and submit the form, and we were made to understand that it is ok to bring the blue form, hopefully the one day training you realize you have to go through a whole lot, different aspects of it and even the internet was a problem it took us some time before we were able to go through but I think they should improve on the internet system because it’s not everyone who is internet inclined, the information was too much and if it could be made simpler like the blue form but honestly I haven’t touched it since I came back and I don’t even remember but since we had it its ok.

<Internals\\IDIs PROVIDERS\\GAPI_02> - § 1 reference coded [10.22% Coverage]

Reference 1 - 10.22% Coverage

I: Can you give me some suggestions to improve reporting among health workers?

R: Documentation is key, you work without documentation its like you haven’t worked, it affects the client as well as the reporting system you wouldn’t know what is happening in the system, we have to do good documentation we don’t have to overlook things, abnormal cases even if it is boil to know whether it is …… or common boil or AEFI, then we have to keep on with the workshops, refresher courses and those who are coming into the system even though they have been trained in school when they come out we have to add it to their orientation and then there should be proper supervision at the post, there should also be posters around on AEFI, it should be catchy so everybody will see.

<Internals\\IDIs PROVIDERS\\GAPI_08> - § 1 reference coded [3.76% Coverage]

Reference 1 - 3.76% Coverage

I: So having said that we will like to know the best way to provide training on Adverse Events?

P: Best way to provide training?

I: Yeah

P: The best is to organize it very often at least so that all staffs will be able to participate and get fair idea on how to identify and manage Adverse Effects.

<Internals\\IDIs PROVIDERS\\GAPI_09> - § 1 reference coded [17.06% Coverage]

Reference 1 - 17.06% Coverage

I: we will also like to know your suggestions for improving the reporting of, ‘erh’ in training of adverse events following immunization

P: oh, I will say that, you see some of the things when you are in school you learn it but once you come to the field and start practicing, you may not even see some at all. So once you come to the field there and you see it…once a while there should be some refresher courses and when there is the change in vaccines we want earlier training rather than when the vaccines are being done, they will be rushing to do training. Sometime you will not be able to grasp everything and the shortage of vaccines too sometimes it’s a really a problem for us. You don’t even know the kind of explanations we should give it to the mothers. Now, there is another problem where we are to give the measles and the, let’s say ‘erh’…is it the meningitis? Yes, alongside with age, one and half year and we give the same measles with the yellow fever. At times you realise that when you open the measles, you give the yellow fever, and the, those coming for the, let’s say the Men-A ARE more than those coming for the measles and the yellow fever;so you realise that you have to open extra one and at the end of the day, there will be some wastage which we don’t like it. ‘eheh’ so if we could a specific one; if it’s measles *two* which will go with the ‘erhm’ the Men-A and we have the measles one which will go for the yellow fever. I think we will prefer that just to limit the wastage

<Internals\\IDIs PROVIDERS\\GAPI_10> - § 1 reference coded [7.49% Coverage]

Reference 1 - 7.49% Coverage

I: so having said that, we will like to know how best to provide trainings on AEFI

P: How best? (Baby crying in the background)

I: yeah! We can provide training on AEFI [baby crying at the background] maybe for your facility

P: oh me I think that err every, from right from OPD to the RCH, every staff should be trained on AEFI, because sometimes the mothers go home and come back and report at the OPD. They don’t come back and report to the RCH staff so when they also have knowledge about AEFI they wouldn’t be treating the fever but they will request for the child’s ‘erhm’ weighing card. Child, that is the child’s health record booklet, check the last time the ‘erhm’ mother had immunisation, so that they can rule out certain things ‘uhuh’. So I think the whole staff should be trained on AEFI because it’s not in, it’s not on only vaccines that there’s AEFI. Other medications too are there, there is AEFI ‘uhuh’.

<Internals\\IDIs PROVIDERS\\NRPI_01> - § 1 reference coded [1.74% Coverage]

Reference 1 - 1.74% Coverage

I: So could you please let us know how best we can provide you with training AEFIs?

P: Through workshops

I: Any other?

P: Coaching

<Internals\\IDIs PROVIDERS\\NRPI_02> - § 1 reference coded [7.37% Coverage]

Reference 1 - 7.37% Coverage

I: So how eeeh what would be the,

you know may be next time of there is going to be training how what form would you like it to take?

P: Emm mostly I would prefer that they should just our management should just designate a person to go to the facility level like when they came to us personal like this so that we see may be you have a peculiar case at the facility you wanted to but because you are a combined staffs you don’t want to - us you come like this if all my colleagues were to be here…

I: For you, you would have preferred something like facility based training.

P: Yes, that one always eem …

I: it will help?

P: M mm

<Internals\\IDIs PROVIDERS\\NRPI_03> - § 1 reference coded [5.18% Coverage]

Reference 1 - 5.18% Coverage

I: Please, let us know how best to provide training on adverse events following immunization.

P: For the facility to train itself?

I: Ehmm how, the question is, please let us know how....best we can provide training on adverse e.. events following immunization?

P: Ehmm the best thing is to ehm call organize a workshop and invite us then we shall be there to ……… participate.

I: Any other way?

P: Ehmm the other way can also to become organize ... Ehm may be training in the first facility ..... for all the staffs.

<Internals\\IDIs PROVIDERS\\NRPI_04> - § 1 reference coded [4.09% Coverage]

Reference 1 - 4.09% Coverage

I: So how best .... can we..... ehhhm provide you training on Adverse Events following Immunization?

P: Mmmm mostly like the normal tranings they just do, if if that one too is done like that you know some times when a training is done it awakens people errh errh [phone ringing] this thing on issues ..... Ehher somebody might have a small knowledge on it alright but she won’t put it into practice but if a training is is done on it you see that it awakens the fellow so he wants to take up take it up.

<Internals\\IDIs PROVIDERS\\NRPI_05> - § 1 reference coded [6.05% Coverage]

Reference 1 - 6.05% Coverage

I: Let me know how best ....... we can provide you training on adverse events following immunization?

P: ( inaudible) I think errrh you have to errrh laze with the District ..... Errrh Health Committee ….. errrh or the District Management Team to try find out maybe appropriate day then you organize all the staff even. It should be at the district level, so that we all attend. It could be a one day or two day thing, then you train the whole group on it. Errrhrrr emphasis the importance of it ...... eerrh, I think does how is suppose to suppose to be done.

I: Is there any other way you can think of to receive the training?

P: Errh any other *ray*, way apart from organizing one at the district level, let me say the can be, okay you can also select a few probably just do a regional thing at the regional level. Select people from the regional level they will go and when they receive the training they will come back and train the rest of the group. Yeah you can also do it that same way.

<Internals\\IDIs PROVIDERS\\UEPI_01> - § 2 references coded [4.05% Coverage]

Reference 1 - 2.53% Coverage

I: ok … errm please let us know how best to provide trainings on, on Adverse Events Following Immunization

P: …….. Yeah I… think …. this errh this training on this it should be something that should be done on week I mean on yearly basis every year at least they should reecho or there should be that errrh errh training for all staff on the adverse events following immunization, uhuh so that every staff should be abreast uhuh maybe if there are any new developments at least we should all be aware uhuh so that at least we can all respond appropriately when we encounter such cases

I: ok …… so yearly?

P: yeah every year it should be done on yearly basis

Reference 2 - 1.52% Coverage

I: ok. Are there any other ways you think would be best to provide this training

P: yeah they can roll it …. they can roll it .. maybe they normally they can invite a cross section from the district then a cross section from the sub district then all the facilities within the sub district will come together then they will train them

I: ok

P: uhuh

I: any other?

P: yeah basically, basically

<Internals\\IDIs PROVIDERS\\UEPI_02> - § 1 reference coded [9.51% Coverage]

Reference 1 - 9.51% Coverage

I: So can you let us know your suggestions for improving training on adverse events following immunization?

P: Well…my suggestions, yes I’ll suggest that…maybe we just organize a training specifically for adverse effects following immunization (ok). With that…most of us…I’m saying most of us because I’m not the only CHO in Ghana here or Bongo, or this thing. Because there are certain things that maybe you may be looking at it that it’s not adverse effect, but it’s; so because of that you see that we’re always complaining we’re not reporting, we’re not reporting, we’re not reporting; because yes, you may see an adverse effect, but it’s like…it’s part of the side effects of the drug so let it be (Ok). You understand

I: Yeah.

P: Whether it’s an adverse effect, you understand. So I think if they just organize trainings specifically for that (Ok) see, they’ll go details (Ok) into it (Ok) so everybody will know much about it; so that when you see certain things you’ll not overlook them.

I: Any other suggestion?

P: Any other suggestion? So since Zokor here (laughs) it’s only Gambrongo (13:26) this facility that you’re coming. So I think it’ll be good you go to the other facilities so that they’ll all benefit from it (Ok). You understand; so maybe if you’re to organize training, the training shouldn’t be limited to the selected facilities that you’ve taken, but it should be…at least cover majority of the facilities so that it’ll help us [talk]…so that…, so as I was saying, the training shouldn’t be limited to the selected facilities (Ok), at least it should be, it should covered…should I say all the facilities (yeah), so that we all have much idea (Ok) on the adverse effects; so that it’ll help us…reduce or prevent them, or be able to detect them in time (Ok) so that action will be taken.

I: Any other you like to add?

P: Well, so far this is okay. If you’re able to do this, it’ll help community…it’ll help those who render services, especially those who are much involved in immunization…in Bongo, in this my facility, Bongo, and then what? Ghana, the whole nation (Ok). It’ll help us all.
