# Supplementary material for: Optimising reporting of adverse events following immunisation by healthcare workers in Ghana: A qualitative study in four regions
Source: PLoS One. 2022 Dec 20;17(12):e0277197. doi: 10.1371/journal.pone.0277197 (PMC9767370; doi:10.1371/journal.pone.0277197)
Supplement: S1 Data — (ZIP) [file pone.0277197.s001.zip › Minimal data/S3 Training on AEFI.docx]

**Name:** 09 Training on AEFI

**Description:** This node contains all information on all trainings related to AEFIs.

<Internals\\IDI EPI\\GAEPI_01> - § 1 reference coded [5.28% Coverage]

Reference 1 - 5.28% Coverage

I: sir, we will like to also know, has your unit received any trainings on AEFI?

P: yes.

I: please in a few words can you please describe the different types of trainings. If…like how long ago was the last…

P: we had a one week training. At least we have ‘erh’ three FDA trained ‘erh’ all the regional teams and also ‘erh’ WHO also had some training but ‘erh’ but then we are yet to train the district level and sub-district ‘arh’ I mean level staff and then [inaudible]

<Internals\\IDI RHMT\\GARI_01> - § 1 reference coded [8.49% Coverage]

Reference 1 - 8.49% Coverage

I: we will also like to know if your unit has received any training on AEFI

P: for the AEFI, I, I, I will not say a formal training. For example, ‘erhm’ during mass immunization like national immunization days ‘erhm’ we used to ‘erhm’, when I talk about mass, it’s just one particular antigen we use it, for example; yellow fever ‘pam, pam, pam, pam, pam, pam, pam’ and then this one will react because I have even forgotten national will also report to if there is any adverse events, they also report to WHO. It goes to food and drug’s board, they also have to, every, all these people are involved. They will all come in, they are all parties to it because, we want to know whether the vaccine because, normally a vaccine comes when you read the ‘erh’ inscriptions will tell you oh, these , that, that, that, that but how many people read the inscription embossed in the ‘erh’ package of the ‘this thing. So one thing people needs to do, they have to read to know that when you give it it’s either this, that but when it’s this then its serious then we have to know if either the vaccine itself is not good or the vaccine is good but it has been exposed, you understand because it needs to be preserved at a certain temperature.

<Internals\\IDI RHMT\\GARI_02> - § 1 reference coded [5.43% Coverage]

Reference 1 - 5.43% Coverage

I: we would also like to know if your unit has received any trainings on reporting of AEFIs

P: yes, severally. Mhm!

I: so we would want to know what different types of training have you received.

P: ‘uhm’ I…I, I can’t remember but what I know is that normally when we are going to do ‘uhm’ …’erh’ national immunisation programmes, yes, we are all taken through the process of AEFI and then also the front line nurses are also taken through. We teach them what they are supposed to do on the field and if there is any ‘uhm’ introduction of new vaccines, they take us through what we are… expecting, what we anticipate, and when it happens what we are supposed to

<Internals\\IDI RHMT\\NRRI_01> - § 1 reference coded [5.64% Coverage]

Reference 1 - 5.64% Coverage

I: Ok, ok. Has your unit receive any training on AEFI?

P: People have attended several, when you say my unit, my unit as in this office?

I: Yes, the

P: Or my unit as in the region? My unit as in this office.

I: Yes please.

P: Yes, people have, people have, and it cuts a broader perspective, am talking of people into EPI activities, people into surveillance activities at the regional level, yes because “errmm” before mass immunizations is one of the topics that is usually treated AEFIs is one of the concerns that is raised but now it has gotten eventually into routine reporting.

I: Ok.

P: That is why is one of the, it is even on the, CD CD2 form.

I: Ok.

P: You know as a reportable event.

I: But in terms of the region, your catchment.

P: “Yea” we have officers in all the districts, so apparently we community nurses and what have you. So apparently and this trainings are not just centered for regional level staff, they are centered for staffs up to the sub-district level or even CHPS level.

I: Ok.

P: So everybody has.

<Internals\\IDI RHMT\\NRRI_02> - § 2 references coded [3.00% Coverage]

Reference 1 - 2.08% Coverage

I: Has your unit receive any training on AEFIs?

P: “Hmmmm”, my unit, “err” I don’t understand, my unit if its unit here you know I am just here I don’t have a big unit you know at the regional level we don’t have a unit but if you talking about “ayii”, persons then I can say in times pass I receive a training on it but not of late, I have receive a training about 20 years ago.

Reference 2 - 0.92% Coverage

I: So.

P: Or is it 20? No about lets say10 to 15 years ago.

I: Ok, Why has your unit not received any training?

P: “Hmmmm”, I don’t know, I can’t explain this.

<Internals\\IDI RHMT\\UERI_01> - § 2 references coded [6.17% Coverage]

Reference 1 - 3.29% Coverage

M: So has your unit received any training on adverse events following immunization?

R: my unit as RCH

M: yes madam

R: no I mean quite a long time I can’t remember the last time we receive training but base on my previous working life and previous training I know but if there are any updates I will not be able to tell those updates.

Reference 2 - 2.88% Coverage

M: can you tell us any possible reasons why your unit has not had any training since that time?

R: well I don’t know but what I know is that it has to do with funding issues when there is no money and they are specify about who should come because of limited funding. That is what I will say.

<Internals\\IDI RHMT\\UERI_02> - § 1 reference coded [0.54% Coverage]

Reference 1 - 0.54% Coverage

M: Has your unit received any training on AEFI’s?

R: Yeah we have received training severally

<Internals\\IDI RHMT\\VRRI_01> - § 1 reference coded [2.06% Coverage]

Reference 1 - 2.06% Coverage

I: ok. Alright ammh, whiles you were talking, you spoke about training, emmh when was the last time you had a training, I mean at the regional level on adverse events following immunization? Have had a training of such nature?

P: Yea, usually ehmm, I think this year around errh April or May when there was introduction of this errh PCV multi dose.

<Internals\\IDI RHMT\\VRRI_02> - § 1 reference coded [1.64% Coverage]

Reference 1 - 1.64% Coverage

I: Ok, ok. Emmh, please has… *(Her phone ringing),* please your unit received erhm, training on adverse events following immunization?

P: Yes, I’ve attended quite a number at the national level.

<Internals\\IDIs DHMT\\GADI_01> - § 1 reference coded [3.65% Coverage]

Reference 1 - 3.65% Coverage

I: Has your unit received training on AEFI?

R: Normally it is not training on AEFI alone but any time there is training on EPI we have an aspect on AEFI but not particularly AEFI alone (phone rings in background)

<Internals\\IDIs DHMT\\GADI_02> - § 1 reference coded [2.95% Coverage]

Reference 1 - 2.95% Coverage

I: Has you unit received training on AEFI?

R: Am just one month old here and can’t say much the disease control officer might give you that information.

<Internals\\IDIs DHMT\\GADI_03> - § 1 reference coded [2.44% Coverage]

Reference 1 - 2.44% Coverage

I: Has you unit received training on AEFI?

R: I just resumed office so I don’t know much of what has gone on the disease control officer might be able to give you that information.

<Internals\\IDIs DHMT\\GADI_04> - § 1 reference coded [1.59% Coverage]

Reference 1 - 1.59% Coverage

I: Has your unit received training on AEFI?

R: Ermm, maybe yes I think there was a workshop.

<Internals\\IDIs DHMT\\GADI_05> - § 2 references coded [4.04% Coverage]

Reference 1 - 1.07% Coverage

I: Has your unit received training on AEFI?

R: No.

Reference 2 - 2.97% Coverage

I: So why haven’t you received?

R: We have not been called, but if there is a training and I will be called fine and if am not involved too.

<Internals\\IDIs DHMT\\GADI_06> - § 1 reference coded [4.46% Coverage]

Reference 1 - 4.46% Coverage

I: Has your unit received training on AEFI?

R: Not specifically on adverse events but we do trainings and chip those things in but anytime we have training on EPI because it’s part of what expect to arise we chip in, we let them understand what we meant by AEFI, you know there is this fear that when they report AEFI it means they are not working, they are not doing well so we have that perception so we have to disabuse their mind from that so tell them when you report its shows you are working and if there is a problem with the vaccine you can easily identify and solve it.

<Internals\\IDIs DHMT\\GADI_07> - § 1 reference coded [3.68% Coverage]

Reference 1 - 3.68% Coverage

I: so please has your unit received any trainings on adverse events following immunization?

P: yes we have. Our disease control officers have received and we have in ‘turned’ trained our officers on the, on the ground especially the community health nurses who go ‘erh’ especially during the introduction of the [inaudible] ‘hmm’

<Internals\\IDIs DHMT\\GADI_08> - § 1 reference coded [4.12% Coverage]

Reference 1 - 4.12% Coverage

I: ‘Errh’ please has you unit received any trainings on AEFI?

P: Yes, last year we received, last year we were having training on men, meningitis so we took ‘errh’ it was in addition to the training materials, it was packaged, AEFI package was inside so we were, the nurses that came were, we run through those things with the form. We distributed the form to them and we went through it for them to get better understanding

<Internals\\IDIs DHMT\\GADI_09> - § 1 reference coded [2.20% Coverage]

Reference 1 - 2.20% Coverage

I: please has your unit received any training on AEFI?

P: [3 seconds] we normally receive it when there is going to be like ‘erh’ expanded programme on immunization. Yes! Whenever we have meeting with them too we stress it, we stress on it, we tell them to fill them because it’s very important, yeah.

<Internals\\IDIs DHMT\\GADI_10> - § 3 references coded [8.28% Coverage]

Reference 1 - 2.94% Coverage

I: Please has your unit received any training on AEFIs?

P: ‘Erhm’, I would say that I’ve been here for just ‘erhm’ about five months. Yes! so if, I’ve not seen any like particular training on it. But anytime we are having ‘erhm’ DHMT meetings, anytime we are having DHMT meetings ‘erhm’ it is chipped in. you know (clears throat) as to what they are to err what err report because sometimes…( knock and door opens)

Reference 2 - 0.79% Coverage

I: So that means it’s a no for the adverse event trainings?

P: No, no. trainings ‘dier’, it’s a no….not at all

Reference 3 - 4.55% Coverage

I: So we will like to know, why have you not received any trainings?

P: We, we receiving the trainings or we conducting the trainings?

I: Like ‘erhm’ receiving trainings

P: Well with receiving the trainings, I because then with receiving the trainings, it will have to come from the top so with that I cannot really tell. But I’m sure that ‘erhm’ probably if they have a schedule they would probably according to districts so if there is a schedule like that definitely it will come up or if they should come round and ‘erhm’ probably train us on some something concerning vaccinations, Immunisations definitely they will chip it in. yeah!

<Internals\\IDIs DHMT\\GADI_11> - § 1 reference coded [4.39% Coverage]

Reference 1 - 4.39% Coverage

I: okay. Please has your unit received any training on AEFI?

P: no!

I: no, then we would like to know; why hasn’t your unit received any training on AEFI?

P: ‘hmm’ maybe, it’s orientation but not ‘erh’ training per say. So that one it’s above us because if we want to do training now there is funds involved.

<Internals\\IDIs DHMT\\GADI_12> - § 1 reference coded [4.13% Coverage]

Reference 1 - 4.13% Coverage

I: So has your unit received any training on Adverse Events Following Immunization?

P: ‘Erhm’ we don’t have one training like this, like the one that is taking place on the second year of life, you chip it in but It’s not a training on its own on AEFI. For some reasons when you are doing malaria training like this, AEFI will come but we’ve not had a training on AEFI solely.

<Internals\\IDIs DHMT\\GADI_13> - § 1 reference coded [4.39% Coverage]

Reference 1 - 4.39% Coverage

I: Please we will also like to know if your unit has received trainings on AEFI

P: AEFI specific training? I would say not really. We usually have AEFI trainings together with when there is a campaign. When there is NID or when there is vaccination campaign or anything of that sort when we are going to administer de-wormers and stuff like that and the people on the ground are trained we add AEFI training to it. So we don’t have AEFI specific training but anytime we do campaigns we do have training in AEFI added onto those trainings

<Internals\\IDIs DHMT\\GADI_14> - § 2 references coded [9.20% Coverage]

Reference 1 - 6.64% Coverage

I: Okay, okay, please has your unit received any training on Adverse Events Following Immunization?

P: [Giggles] I was asked this question before and my answer, answer was that there was never a training *specifically* that on this day come and sit here for training on AEFI but if there is a programme [inaudible] let’s say ‘erhm’…polio those days we were having polio mass immunizations, we were having the cevaries the [inaudible] cancer. During the training we talked about AEFI but not a training structured purposely for AEFI. That one I hve neer sat into any programme like that before.

Reference 2 - 2.56% Coverage

I: If that’s the case can we know why you people have not received training on

P: I don’t know. That ‘erhh’ training specifically not for any other thing but all the resources being channeled toward AEFI training I don’t know.

<Internals\\IDIs DHMT\\GADI_15> - § 1 reference coded [3.92% Coverage]

Reference 1 - 3.92% Coverage

I: Please has your unit received any “erh” training on adverse events following immunization?

P: not training per say specifically to AEFI but, we don’t organise one training specifically for AEFI but it’s more or less integrated training. So anytime I have opportunity to train them on expanded programme, new updates we add the AEFI form to them

<Internals\\IDIs DHMT\\NRDI_01> - § 1 reference coded [6.41% Coverage]

Reference 1 - 6.41% Coverage

I: Thank you very much so at this at this moment I want to just find out eeh how is eeh have you receive as a unit receive any training concerning AEFI?

P: Quite well w’vebeing any time we have any programme EPI may be at the region or they hammer on it sometimes our District director is also a nutrition eehm a disease control officer and so mostly those things he has encouraging staff to report on it don’t afraid and maybe you will report on it and you will be blamed is not a blamed game thing but then when you give a child an immunization and anything happen, and they come don’t hide it just report into the highers so that they will take actions not they wouldn’t blame for what has happen so they are been encourage to report on it.

<Internals\\IDIs DHMT\\NRDI_02> - § 1 reference coded [3.32% Coverage]

Reference 1 - 3.32% Coverage

I: That will do sir, thank you very much so, has anybody eeh your facility here received training on AEFI.

P: Yes! Yeah training has been done hmm I think that it was done eeh at the regional level. I don’t remember the district itself a yaah training are sometimes eeh usually during HIVs as part of the training of staff eeh AEFI is added so training has been done.

<Internals\\IDIs DHMT\\NRDI_03> - § 1 reference coded [5.79% Coverage]

Reference 1 - 5.79% Coverage

I: Have you received any, eeh has your unit here received any training on AEFI?

P: AEFI eeem I remember that was some years back now we have AEFI coordinator in the district here because normally we do programmes and then we assume that injections given to children at time they may come down with some AEFI signs and symptoms. There was a one person called to the region to train so that he is the AEFI coordinator so that in case anything when they report to us we report to him the form he fill then send to the next level.

<Internals\\IDIs DHMT\\NRDI_04> - § 2 references coded [5.41% Coverage]

Reference 1 - 3.20% Coverage

I: Has your unit received training on adverse events following immunization?

P: Arrrrh, Nooo ..... No in the sense that is a specific training for that particular IE errr IEM and CE but there was a training that we were given to staff ….. oon MMCH ehmm maternal health activities but we incorporated those activities, those incidences in that training but is not a training for IEA specifically so no in a sense but yes because we always incorporate it to (inaudible) the training we are doing now before the end we cheap in some of those things because is the same nurses who are there to benefit the training so that’s what we always do. Nhmmm.

Reference 2 - 2.22% Coverage

I: Why have you not received .... any training?

( 3 seconds )

P: Aiii this one can I, can I, can I respond, because I don’t know why probably there is no funding or may be the programme has not, the programme at the regional or national level has not schedule training for that one or of course may be am not aware but I, I may relate it to funding or if we also have our own funding we could call our staff and give training on that one specific.

<Internals\\IDIs DHMT\\NRDI_05> - § 1 reference coded [0.68% Coverage]

Reference 1 - 0.68% Coverage

I: Okay, sir has your unit receive any training on AEFI?

P : Yah, that was some time back about two three years ago ......... Yah.

<Internals\\IDIs DHMT\\NRDI_06> - § 1 reference coded [0.68% Coverage]

Reference 1 - 0.68% Coverage

I: Okay, sir has your unit receive any training on AEFI?

P : Yah, that was some time back, about two three years ago ......... Yah.

<Internals\\IDIs DHMT\\NRDI_07> - § 1 reference coded [0.64% Coverage]

Reference 1 - 0.64% Coverage

**I:** Ok, Ok, Has your unit received any training on AEFI

**P:** Yeah, yeah, we have received training, we have received training.

<Internals\\IDIs DHMT\\NRDI_08> - § 1 reference coded [1.76% Coverage]

Reference 1 - 1.76% Coverage

**I:** Has your unit received any training in AEFI?

**P:** AEFIs, is in service training. Like when they come newly, newly posted these things we orientate them but formal training, no

<Internals\\IDIs DHMT\\NRDI_09> - § 1 reference coded [2.25% Coverage]

Reference 1 - 2.25% Coverage

**I:** Has your unit received any training in AEFI?

**P:** Err… Specifically no, but there has being programs that has a component of AEFI in it. Though it may not do, go deep in the AEFI but it touches the surface so do I say yes, yes.

<Internals\\IDIs DHMT\\NRDI_10> - § 1 reference coded [5.75% Coverage]

Reference 1 - 5.75% Coverage

**I:** Has your unit received any training in AEFI?

**P:** Not the office per say but the Directorate.

**I:**  Those working in the Directorate here?

**P:** Yes, they have received any training on; there are a lot of trainings at the regional level. They have received training but most of the training with it at the regional level and not at the District Level but I feel with time it is high time we do it at the district level too. Everything is now at the regional level.

<Internals\\IDIs DHMT\\NRDI_11> - § 2 references coded [4.41% Coverage]

Reference 1 - 1.13% Coverage

I: Ok, Thank you very much. Has your unit receive any training on AEFI?

P: “Errr”, personal me no but my staff have received training in AEFI.

Reference 2 - 3.29% Coverage

I: Ok.

P: Are you asking in respect to which dates?

I: Not, not dates mummy.

P: But then by virtue of our training, everybody has a gist of what AEFI is.

I: Ok.

P: Not necessarily calling you to a formal kind of this thing but have all been trained in one way or the other.

I: “Hmm”.

P: “Hmm” not necessarily calling you to come for a separate workshop but workshops of EPI usually we will touch on AEFIs.

<Internals\\IDIs DHMT\\NRDI_12> - § 1 reference coded [4.07% Coverage]

Reference 1 - 4.07% Coverage

I: Ok. Has your unit receive any training on AEFI?

P: “hmmm” …you mean here?

I: Public health unit, the Metro public health unit.

P: Public health, Metro public health unit? 3second “hmmm” ok.

I: You and your staff.

P: Me and my staff, me and my staff “eyiii”. I don’t think we have may be some time, “eemmm” because err but most at times when we do any err when we get any training “haaa” may be like we use to 2YL this thing, they do I mean also discuss it but to just structure this is AEFI training no but any other training we do be it may be a durbar or this training at or when job they talk about it but structured training this thing I haven’t had that this thing may be so this is I mean I don’t have this thing.

<Internals\\IDIs DHMT\\NRDI_13> - § 1 reference coded [2.74% Coverage]

Reference 1 - 2.74% Coverage

I: Has your unit received any training on AEFI

P: several times several times most of the community health nurses and then the field technicians they get training on that and any time there is going to be on immunization they take them on this effect

<Internals\\IDIs DHMT\\NRDI_14> - § 1 reference coded [2.99% Coverage]

Reference 1 - 2.99% Coverage

I: Has your unit receive any training on AEFI

P: yes our unit mostly let me see when was that if most of the new antigen they brought they do tech us how to go about it we have to tell the mothers allow them to come if probably if fever is more than 3 days.

<Internals\\IDIs DHMT\\NRDI_15> - § 2 references coded [6.19% Coverage]

Reference 1 - 4.89% Coverage

I: Has your unit received any training on AEFI

P: not really but sometimes when we have programmed like immunizations AEFI is one of the topics that we are taken through incase during that exercise we encounter such a problem than we know how to handling that condition

Reference 2 - 1.30% Coverage

I: Has your district unit received AEFI reports in the last year

P: yes

<Internals\\IDIs DHMT\\UEDI_01> - § 2 references coded [2.48% Coverage]

Reference 1 - 1.22% Coverage

I: ok, the normal forms, so has your unit received training on adverse events following immunization?

P: no, no, they haven’t received anything like that.

Reference 2 - 1.26% Coverage

I: ok, do you happen to know why you haven’t received training on adverse events following immunizations?

P: no, not really, I don’t have any idea, (inaudible)

<Internals\\IDIs DHMT\\UEDI_02> - § 2 references coded [12.06% Coverage]

Reference 1 - 4.86% Coverage

I: Ok. So has your unit received any trainings on adverse events following immunization?

R: Well, I have.

I: You have.

R: I have and then eh… because of my background as a pharmacist I have also read a little bit about it (Ok). But there are plans to really train the entire staff because when it comes to adverse reactions, eh everybody is a stakeholder (yeah). So long as you work in the facility, somebody could be vaccinated, gets home, come back to the facility; if there is no pharmacist or doctor or nurse, any staff available should be able to deal with the matter (yeah). Yeah. So that is it. So we plan to train but I think we need some resources. We cannot bring people together without giving them certain things.

Reference 2 - 7.19% Coverage

I: Yeah, that’s true. And so should I take it that your unit has not been trained, you have been trained

R: I have been trained but eh we’re yet to train all staff in the district.

I: Ok. So meaning you haven’t been trained yet, I mean your unit has not been trained yet.

R: Yeah. The district has not been trained.

I: Can you please tell me why you’ve not received any training?

R: Hmm. Eh the reason. I think the reason is as said, it’s a very important training that we need to do (Ok). It has to do with some funding. So funding is the main thing (Ok). But it’s very important for us to train the people (Ok). And besides, you know all of us are health professionals; those who do the immunization directly, they let me say they’re familiar with these reactions (yeah). So even though they haven’t had a formal training from the district (Umm), in one way or the other, they’ve had it in their various eh trainings (Ok) eh in school. So basically it’s just left for me to be able to provide just maybe lunch, snack, and the training materials (Ok) for it to take place.

<Internals\\IDIs DHMT\\UEDI_03> - § 2 references coded [5.31% Coverage]

Reference 1 - 2.48% Coverage

I: Yeah. Ok. So has your unit received any training on adverse events following immunization?

P: Our unit?

I: Yeah.

P: Yes! I will say yes in the sense that, at times when we go for eh…like EPI, eh workshop, or we may go for let’s say that TB. Some training you know because we give drug, some, those things are also definitely comes. So when it comes to like that they add. So I will not say that I never received training (Ok). Training does not mean that you should go to class room before you’re trained. So training, it mean that whatever that somebody have been able to you knowledge, and you understand it, its part of training.

Reference 2 - 2.83% Coverage

I: Ok. Ok. So like you mentioned you went to the EPI training and other trainings (yeah). Ok… can you please describe the different types of training you were just talking about on adverse events following immunization?

P: Yeah. We, that’s what I’m saying. Because it come with this thing, they will take us through how to report, reporting is there (Umm), how to get the cases. Just like what I’m saying (yeah), the signs and symptoms you pick to do, so they will take us through that (Ok). So from there, then the timing and all those things, and then later on you try to find out, what how is the situation. Whether the person is recovering or not recovering (Ok). So those things, they will let us know those things (Umm).

<Internals\\IDIs DHMT\\UEDI_04> - § 1 reference coded [5.20% Coverage]

Reference 1 - 5.20% Coverage

I: thank you very much sir. Would you say your district has received any training on adverse events following immunizations?

P: I should say yes, you know adverse events following immunizations usually concentrating on EPI but the trainings that have gone on reporting of adverse events eventually cover up for the immunization as well, so yes even in the course of this year there was a training at Ex-Tee (a hotel) on generally reporting of adverse events by the food and drugs authority and some sub-districts of this district were invited, I remember very well I think Basuende was invited, some of the sub-districts were part of it. yeah

<Internals\\IDIs DHMT\\UEDI_05> - § 1 reference coded [1.10% Coverage]

Reference 1 - 1.10% Coverage

I: okay, thank you. Has your unit received training on adverse events following immunization?

P: aaaah yeah but it has being long. It has being long.

<Internals\\IDIs DHMT\\UEDI_06> - § 2 references coded [1.15% Coverage]

Reference 1 - 0.86% Coverage

I: thank you very much, has your unit received any training on AEFI’s?

P: (breathes out heavily) training geared towards AEFI particular or you go for workshop and AEFI comes in?

Reference 2 - 0.29% Coverage

I: any? Where ever there is a mention of it?

P: I think yeah

<Internals\\IDIs DHMT\\UEDI_07> - § 1 reference coded [5.20% Coverage]

Reference 1 - 5.20% Coverage

I: thank you very much sir. has you say your district has received any training on adverse events following immunizations?

P: there was a training and usually its added to any form of training that has to do with vaccination its always part and parcel of that (loud voices of people talking in the background), the last EPI training we had for staff, I think last year or early this year, the adverse reaction was part of it. Reporting of adverse reaction.

<Internals\\IDIs DHMT\\UEDI_08> - § 2 references coded [7.02% Coverage]

Reference 1 - 3.30% Coverage

I: okay so has your unit received any training on adverse events following immunizations?

P: that one I think the disease control unit can tell, I cant tell on that.

I: so your office as public health officer ....

P: mmhm, no since I came there hasn’t been any training like that.

I: how long have you been in the district or in that office?

P: that’s 2015, I took over from somebody.

Reference 2 - 3.72% Coverage

I: okay, okay okay so would you have an idea why you have not being trained on adverse events.

P: most of eerrhh the children that come to our facility they are usually attended by clinicians or the community health nurse, so with the community health nurse am sure they might be trained but its the disease control officer that can tell, I cant tell much about that but with the midwife, I don’t think any training on that has been done.

<Internals\\IDIs DHMT\\UEDI_09> - § 2 references coded [3.56% Coverage]

Reference 1 - 1.86% Coverage

M: Has your unit received any training on adverse events following immunization?

R: Not to the best of my knowledge. Over the years what we have been using that is it.

Reference 2 - 1.70% Coverage

M: If no can you advance reasons why you have not had any training?

R: Well that one I wouldn’t actually know the reasons why we have not been involved.

<Internals\\IDIs DHMT\\UEDI_10> - § 3 references coded [7.85% Coverage]

Reference 1 - 2.22% Coverage

M: Has your unit received any training on AEFI’S?

R: Not AEFI’s to be specific, but there are other programmmes that AEFI’s are embedded in it. For instance, in surveillance training, when we go for surveillance training these are some of the things we look out for.

Reference 2 - 1.58% Coverage

M: But you have not had any training by AEFI’s.

R: Not specifically unless there is any other trainings and is a sub of what we are training, but there is not specific training for AEFI’s.

Reference 3 - 4.06% Coverage

M: Can you advance reasons why training has not been organized specifically for AEFI’s?

R: I think for now what we always expect is ideally if you are calling for a training it means you have a lot of funding to do that and once there is no funds, and two it is not something that is too much or too serious for us to organize a special training for AEFI’s even though it is expected. But any time we organize NTD’s training there is a component of adverse drug reactions and those things.

<Internals\\IDIs DHMT\\UEDI_11> - § 1 reference coded [3.52% Coverage]

Reference 1 - 3.52% Coverage

M: Has your unit received training on adverse events following immunization?

R: Yah we have received some form of training. Usually, when we have national exercises we include in it adverse events following immunization training to the health workers and also to the volunteers who come in contact with these parents in their homes. So some form of training has been given to the staff of DHMT and the various levels of health care delivery

<Internals\\IDIs DHMT\\UEDI_12> - § 3 references coded [5.96% Coverage]

Reference 1 - 0.89% Coverage

I: alright, thank you very much; please has your unit received any training on AEFI’s?

P: for within the year or two not specifically on that.

Reference 2 - 2.57% Coverage

I: okay, why haven’t you received any training?

P: okay it is it is eerrh the adverse events eerrh following immunization I think it’s part of the EPI so like the time that we were doing the polio immunization national NID’s you see there used to be frequent training on it, aaaha, so the small I can say is during the SMC which we it is packaged part of it that you explain, but formally on adverse events, no.

Reference 3 - 2.50% Coverage

I: okay, so are you suggesting you have not received any training and you don’t have any idea why you haven’t had any trainings?

P: well I said formal training eerrh I have not received (slamming of door in a distance) I think it is one of the programmes that officers need to take on because those above need to train us on it so the more we know about it the more we are able to capture and report.

<Internals\\IDIs DHMT\\VRDI_01> - § 2 references coded [11.69% Coverage]

Reference 1 - 5.37% Coverage

I: That’s great, has you unit receive any training on AEFI?

(4seconds)

P: eeeh not eeeh, you know eeeeeer I will say yes, yes because eeer we recently have eeer a training on EPI and anytime you know because (car horn pee pee pee) they are not, I don’t see whether they don’t see the need, some, let me put it that way not all so is like sensitization continuou goes on anytime there is a forum or they meet eeer review meetings when the need arise we always mention that or inform the staff there, so I will say we are informed about it, my unit is informed about it because I have myself and other two CHNs working (inaudible) and they even go to the field dough they are here to support, they go to the field when the need arise so they also interact with us so we are aware the unit is aware of this and partakes in that.

Reference 2 - 6.31% Coverage

I: so you are saying eeem training of AEIF is always chipped in other training

P: Yes, when the, as I said is like some are not doing, some, some don’t, let me put it some don’t take it, some don’t take serious so they need to be prompted always reminded of, it need to be reported kind of thing so that they, they don’t take it as is just one of those things yeah that’s what am trying to say.

I: okay, so whenever there is training AEFI is chip in

P: yeah mm all problem areas are chip in including AEFI, (3seconds) that is the only way and means we can get them because there may not be any money allocated to say come solely for AER, AEFI training eheeh so we integrate such eeeh I will say orientations maybe they were, some were, are not aware their senior colleagues are trying to put them right but you know to do it better and since we continue to update the system maybe new things has come we need to you know disseminate information through those eeeh meetings.

<Internals\\IDIs DHMT\\VRDI_02> - § 2 references coded [5.08% Coverage]

Reference 1 - 2.27% Coverage

I: Okay! Has this unit received any trainings on adverse events… following immunization?

P: Mmm Since I’ve come I’ve not I’ve not…

Reference 2 - 2.81% Coverage

I: Oh okay! Can you please tell us why you have not received any training? Is there any particular reason they’ve given…[pauses] for the long break?

P: That mmm…

<Internals\\IDIs DHMT\\VRDI_04> - § 1 reference coded [1.41% Coverage]

Reference 1 - 1.41% Coverage

I: Okay, errhm has your unit received any trainings on adverse events?

P: It it was a general training, I think I… I attended that was in in 2015, somewhere June, June CHO (not too sure) training in 2015.

<Internals\\IDIs DHMT\\VRDI_05> - § 1 reference coded [2.36% Coverage]

Reference 1 - 2.36% Coverage

I: Thank you, has your unit received any training on AEFI?

P: Well, eeeh am a public health nurse we have been trained long time ago but we just had eee second year of life immunization training and during the, we discussed the new EPI policy so we discuss the AEFI also.

<Internals\\IDIs DHMT\\VRDI_06> - § 1 reference coded [5.87% Coverage]

Reference 1 - 5.87% Coverage

I: erhmm has this unit received any training on adverse events? Cars passing and honking

P: yeah! They’ve had some training; I think twentyyy…errr sixteen or so. Errrh… They…they had some training…and some time when errrh new vaccines are coming in, they train the people but sometimes because parturition and errr new staff being posted and all that, sometimes, some few people…

<Internals\\IDIs DHMT\\VRDI_07> - § 2 references coded [9.33% Coverage]

Reference 1 - 0.99% Coverage

I: Ok. Ahmm, has your facility received any form of training on adverse event following immunization as a district?

P: As a district?

I: Yes.

Reference 2 - 8.34% Coverage

P: Can I remember something like that? I think sometime, but not in my… before I came here,

I: Ok.

P: I think there was an exercise like that where I had to send one of my boys in collaboration with the Food and Drugs Board.

I: Ok.

P: mhmm, you know some of the events, we report straight to them.

I: Ok.

R: They also have a format which we use especially when it’s drugs,

I: Ok.

R: Adverse events following drugs, we report through the standard board. And they often… Sometime they have a bulletin that once awhile they come back and they tell you about the sample you sent, and what was the outcome etc. I could remember my last district, when I read the bulletin, I realized that, that particular incident that happened in my district was featured in the bulletin.

I: Ok. Erhm, but currently, your current post, your current district, you’ve not received any training of…

P: I think there was one, I didn’t go. I had to send somebody, I think from the health centre or so. I sent somebody from the health centre for one like that. I think it was the same Food and Drugs Board, but I can’t remember the exact… what they went to do exact but I think there was a request or something like that. I can’t remember.

<Internals\\IDIs DHMT\\VRDI_08> - § 1 reference coded [3.50% Coverage]

Reference 1 - 3.50% Coverage

I: Alright, ehmm, so when was the last time you had a training on adverse events for staff?

P: For, for, for EPI, I’ve being here for the past 3 years. I can… But for Adverse Events for all the general medications from Food and Drugs Board, we had.

I: Ok.

P:Yea but for EPI specifically, I think there was one. I need to find out from the disease Control officer.

<Internals\\IDIs DHMT\\VRDI_09> - § 1 reference coded [7.52% Coverage]

Reference 1 - 7.52% Coverage

I: ok, ok. Ehmm when was the last time your unit receive training with respect to AEFI?

P: With respect to AEFI.

I: Yes.

P: I will say…

I: at the district level,

P: at the district level, ok let me ehrr, erhmm, I think in October. It wasn’t specifically on immunization per say,

I: ok.

P: but we had a regional training towards the mass drug distribution of Avemetin. (*some one talking at the background*) And so part of it also looked at adverse event in general.

I: yea, yea, ok.

P: the drug, and then vaccine. So it was look at in general. Ahaan, so same thing when we were also doing the training for the health staff who would supervise the distribution as well as the community directed distributors. We also mentioned some of the adverse events.

I: ok

P: but of course because we were not dealing with immunization here, it wasn’t related, specifically related to immunizations so it was generally on adverse event that may occur after

I: the drug

P: the drug administration.

I: ok, So ahmm, with respect to adverse event following immunization, when was the last time you had a training for the staff on the field?

P: No, I actually came here onnn, this march.

I: ok.

P: Mhm this march.

I: ok.

P: No training of such as, has been done.

<Internals\\IDIs DHMT\\VRDI_10> - § 2 references coded [1.96% Coverage]

Reference 1 - 0.83% Coverage

I: Ok, ok thank you. Amh, has your unit received any training on adverse event following immunization?

P: Errh, actually, errh, I must say no,

Reference 2 - 1.13% Coverage

I: ok

P: In such a way that if we have any other training and we chip in is different

I: ok

P: but solely for this particular adverse event following immunization, I may say at the moment, no.

<Internals\\IDIs DHMT\\VRDI_11> - § 1 reference coded [2.84% Coverage]

Reference 1 - 2.84% Coverage

I: Alright ok. Emmh, Madam, emmh has your unit received any training on adverse events following immunization?

P: Yea, is errh always form part of our, our orientation on eerrh programs especially when there is going to be any immunization program. Like national immunization days, when new vaccines are introduce and then we are oriented. It always forms part of it. It always forms part of it.

<Internals\\IDIs DHMT\\VRDI_12> - § 1 reference coded [2.61% Coverage]

Reference 1 - 2.61% Coverage

I: ok, ok.. Has your unit received any training on AEFI?

P: Nnnh, yes when like… the last time we were trained on this measles rubella, we were taking through AEFI and the Food and Drug Board who came once, and to organise this AEFI something workshop, some half day meeting be that I was part.

<Internals\\IDIs DHMT\\VRDI_13> - § 1 reference coded [2.44% Coverage]

Reference 1 - 2.44% Coverage

I: Ooh ok thank you sir eerre has your unit receive any training on AEFI?

P: Yes we have been receiving training during eere EPI anytime we are coming to do national immunization we receive AEFI training we go through the form as usual.To update ourselves.

<Internals\\IDIs DHMT\\VRDI_14> - § 1 reference coded [1.53% Coverage]

Reference 1 - 1.53% Coverage

I: (laughing) has your unit received any training on AEFI?

P: mmm some [loud conversation in background] some few some months ago mhum.

I: some months ago.

P: mhumm.

<Internals\\IDIs DHMT\\VRDI_15> - § 1 reference coded [3.66% Coverage]

Reference 1 - 3.66% Coverage

I: Eere has your unit receive any training on AEFI?

P: Mhm following immune [cell phone rings] actually following immunisation no but for other services like mass drug distribution yes we, we do have T.O.T and then we bring it down to district level.

<Internals\\IDIs FDA\\GAFDA_01> - § 1 reference coded [13.13% Coverage]

Reference 1 - 13.13% Coverage

I: Okay, ‘erhm’ please has your unit received any training on AEFI?

P: Yes we have.

I: And ‘erhm’ please ’erhm’ can you describe the different types of trainings on AEFIs you have received?

P: The last training we were facilitators, that’s why am looking at you ‘erhm’

I: Okay

P: So as in?

I: So the last, you provide training most of the times?

P: Yes we provide training most of the time.

I: Okay, okay so let’s look at the trainings you have provided ‘erhm’ can you describe the last training?

P: The last training we had, was for which group of people?...we usually train the health workers in hospitals on AEFIs , on the different types, how to report… what to report.

I: And, and, , a how long ago was that?

P: Last year

I: Last year, and please what was the duration of this training?

P: ‘Erhm’ for the hospitals one hour if it’s an intensive AEFI training like we had for our technical advisory committee that was a five day training.

<Internals\\IDIs FDA\\GAFDA_02> - § 2 references coded [2.73% Coverage]

Reference 1 - 1.20% Coverage

I: okay, please has your unit received any trainings on AEFIs?

P: Yes, we receive trainings [giggles], we provide trainings on AEFIs

Reference 2 - 1.53% Coverage

I: You, you receive and you provide?

P: Yes! because this is more this is we are the national level so we trains regions and then the regions train the districts on AEFI.

<Internals\\IDIs FDA\\NRFDA_01> - § 1 reference coded [2.08% Coverage]

Reference 1 - 2.08% Coverage

I: has your unit received any training on Adverse Events Following Immunization?

P: yes, I have received a lot of training on Adverse Events Following Immunization because I work with FDA, we have a whole department called safety monitoring department and we are that is our mandate to collate Adverse Events Following Immunization and then Adverse Drug Reactions

<Internals\\IDIs FDA\\UEFDA_01> - § 1 reference coded [3.46% Coverage]

Reference 1 - 3.46% Coverage

M: Has your unit received any training on adverse events following Immunization?

R: Yes, we have been receiving trainings from our head quarter level severally on how to fill the form, we also do education for health care professionals on how to fill the forms, the type of adverse events they should expect and how they should categorized them into minor and serious ones and all that we have been doing that. Even this year we have offered training for some health care professionals.

<Internals\\IDIs FDA\\VAFDA_01> - § 1 reference coded [1.88% Coverage]

Reference 1 - 1.88% Coverage

I: Emmh, again now at your level, as far as your staff are concern, have they receive… when was the last time they’ve some form of training on Adverse Events Following Immunization?

P: The last time was this year. Myself too, we all were gathered at Dodowa,

I: ok

P: and were given… with all the ICPs from Volta

I: ok

P: and then we were given tip bits on errh….,

I: adverse event following immunization

P: adverse event following immunization, yea. So I will say my staff here, by designation, what I do is to designate one person who is responsible for this particular thing

I: ok

P: every year

I: ok

P: So you also…. everybody here knows about AEFI

<Internals\\IDIs PROVIDERS\\GAPI_01> - § 1 reference coded [0.81% Coverage]

Reference 1 - 0.81% Coverage

I: Have you received training on adverse events?

R: Yes

<Internals\\IDIs PROVIDERS\\GAPI_02> - § 1 reference coded [0.85% Coverage]

Reference 1 - 0.85% Coverage

I: Has your health facility received training on AEFI’s?

R: Yes

<Internals\\IDIs PROVIDERS\\GAPI_03> - § 1 reference coded [0.88% Coverage]

Reference 1 - 0.88% Coverage

I: Has your facility ever received training?

R: Yes

<Internals\\IDIs PROVIDERS\\GAPI_04> - § 1 reference coded [2.76% Coverage]

Reference 1 - 2.76% Coverage

I: Has your health facility received training?

R: As for training it’s not just on AEFI, normally when there is training about immunization then we talk about AEFI but not just specifically for it but it is combined with others.

<Internals\\IDIs PROVIDERS\\GAPI_05> - § 1 reference coded [0.58% Coverage]

Reference 1 - 0.58% Coverage

I: Has your facility received training on AEFI?

R: Yes

<Internals\\IDIs PROVIDERS\\GAPI_06> - § 2 references coded [5.24% Coverage]

Reference 1 - 1.14% Coverage

I: Okay, so please has your health facility received any training on AEFI?

P: No, no training, no training

Reference 2 - 4.11% Coverage

I: If no, then why has your facility not received any training?

P: ‘Errhn’ because here is not a target, here we hardly give people immunization unlike other health facilities where they care for the whole community. When there is outbreak of any disease people come there for vaccinations and other things but here we don’t do those things so I don’t think there is any need for that.

<Internals\\IDIs PROVIDERS\\GAPI_07> - § 1 reference coded [3.33% Coverage]

Reference 1 - 3.33% Coverage

I: okay, has your health facility received any training on AEFI?

P: and then the forms too are filed [talking in background]

I: ‘erh’ has your health facility received any training on adverse events?

P: yes, yes

I: okay, ‘erh’

P: this year

<Internals\\IDIs PROVIDERS\\GAPI_08> - § 2 references coded [7.23% Coverage]

Reference 1 - 2.71% Coverage

I: So please has your health facility received any training on Adverse Events Following Immunization? The, this facility ‘erhm’ have you people received trainings on Adverse Events Following Immunization?

P: No please

Reference 2 - 4.52% Coverage

I: [talkaing in background] So ‘erhm’ if no, why is it the your facility has not received any training on Adverse Events Following Immunization?

P: Ah, okay i think it’s from the people organizing them they normally come around and ask us about the training needs, the things that we need to know, but from last year they didn’t provide any training [inaudible].

<Internals\\IDIs PROVIDERS\\GAPI_09> - § 1 reference coded [1.15% Coverage]

Reference 1 - 1.15% Coverage

I: so has this health facility received any training on adverse event following immunization.

P: yes.

<Internals\\IDIs PROVIDERS\\GAPI_10> - § 1 reference coded [2.67% Coverage]

Reference 1 - 2.67% Coverage

I: and has your health facility received training, any training on AEFI?

P: no

I: no?

P: yes

I: if no, we will want to know why your health facility hasn’t received any training on AEFI

P: maybe it’s in the pipeline. Yes, it’s in the pipeline. Maybe next year, the region can decide that we should have training on AEFI. Yes!

<Internals\\IDIs PROVIDERS\\GAPI_11> - § 1 reference coded [1.75% Coverage]

Reference 1 - 1.75% Coverage

I: so please has your health facility received any trainings on AEFIs?

P: yeah, that’s what I said; from the district, they come and train us.

<Internals\\IDIs PROVIDERS\\NRPI_01> - § 2 references coded [2.16% Coverage]

Reference 1 - 0.80% Coverage

I: Has your health facility received training on AEFI?

P: No

Reference 2 - 1.36% Coverage

I: Why has your facility not received any training on AEFI?

P: I can’t tell I don’t any idea about that

<Internals\\IDIs PROVIDERS\\NRPI_02> - § 1 reference coded [1.20% Coverage]

Reference 1 - 1.20% Coverage

I: So has your health facility received any training on AEFIs?

P: As I said earlier yes we did receive.

<Internals\\IDIs PROVIDERS\\NRPI_03> - § 2 references coded [7.99% Coverage]

Reference 1 - 1.72% Coverage

I: Has your health facility received any training on adverse events following immunization?

P: No I can remember I can't remember .... Ehm ever been receiving any training.

Reference 2 - 6.27% Coverage

I: Does that mean you have not received any training?

P: For myself?

I: No! Your facility.

P: My facility in general, I can't tell specifically because ehhh I came to take over from somebody and I don't know whether he was trained before or some of the staff were trained before.

I: So, your three years of being here no training?

P: No, for my three years of being here no.

I: Why has your facility not received any training on adverse events following immunization?

P: Ehmm, for that one I can't tell (laugh) ehm(laugh) I can't tell because ehmm ..... may be those who are to organize those training haven't invited us yet.

<Internals\\IDIs PROVIDERS\\NRPI_04> - § 2 references coded [3.19% Coverage]

Reference 1 - 0.88% Coverage

I : Has your health facility received any training on adverse Events following immunization?

P : No.o.o No.o

Reference 2 - 2.32% Coverage

I : Why has your facility not received any training on Adverse Events Following immunization?

P: I think that one theirr we can’t answer it, you know the traning and all these things are organized by the District and the Region so they will know why......( coughing) ( paper flipping)

<Internals\\IDIs PROVIDERS\\NRPI_05> - § 2 references coded [1.75% Coverage]

Reference 1 - 0.81% Coverage

I: Has your health facility receive any training on adverse events following immunization?

P: No,no since I came here …. I have not.

Reference 2 - 0.94% Coverage

I: Why has your facility not received any training on adverse events following immunization?

P: I wouldn’t know (laugh) to be frank ...... I don’t know.

<Internals\\IDIs PROVIDERS\\NRPI_06> - § 2 references coded [2.68% Coverage]

Reference 1 - 1.16% Coverage

**I:** OK, Has your health facility received any training on AEFIs?

**P:** I received it in the region but not in the facility here. So I don’t know whether maybe they have ever.

Reference 2 - 1.52% Coverage

**I:** Hmm, So you went on behalf of the facility

**P:** Facility, yeah and when I came I also took them through. Normally, when you go, you represent the facility, and when you come you also take them through what you went and did.

<Internals\\IDIs PROVIDERS\\NRPI_07> - § 2 references coded [5.87% Coverage]

Reference 1 - 2.13% Coverage

**I:** Has your health facility receive any training on AEFI?

**P:** Aaa... I should say, no. we have not have a direct training on it. we are always encouraged that in the case of any adverse effects we should report, especially serious cases but for adverse effects management, no, no specific training of such nature has been organized.

Reference 2 - 3.74% Coverage

**I:** Why has your facility not received any training on AEFIs?

**P:** That is the big question, because trainings are normally organized by the district management team. They normally determines the kind of training to be organised.so we expect that they should be able to determine why such and such trainings. But i can basically say that since reporting are not been done regularly because like i said initially, some of the adverse effects are manageable on the group. So since we don’t report, the DHMT do not see that challenge to the staff so they also stay silent towards that.

<Internals\\IDIs PROVIDERS\\NRPI_08> - § 1 reference coded [0.96% Coverage]

Reference 1 - 0.96% Coverage

**I:** OK…Has your health facility received any training on AEFIs?

**P:** Yes…we have received training on it.

<Internals\\IDIs PROVIDERS\\NRPI_09> - § 1 reference coded [1.03% Coverage]

Reference 1 - 1.03% Coverage

I: Has your health facility received any training of AEFI

P: yes

<Internals\\IDIs PROVIDERS\\NRPI_10> - § 1 reference coded [1.58% Coverage]

Reference 1 - 1.58% Coverage

I: Has your health facility received training on AEFI

P: no please

<Internals\\IDIs PROVIDERS\\UEPI_01> - § 1 reference coded [8.23% Coverage]

Reference 1 - 8.23% Coverage

I: earlier I asked you if your health facility has received training on AEFIs and you said no

P: no you know these, is one of the basic packages in our various training

I: ok

P: I actually when we started work we have not had any refresher training or we have not had any formal training on AEFIs

I: ok can you please tell why you’ve not had training, do you know why this training

P: you know this training are not just organized at the local base. This trainings are always determined at the national level

I: ok

P: so at the national level they see that there should be a training on this or a workshop on this then they roll out the program for every region then for every district.ok so you think because they have not been able to come up with a plan that it has to happen that is why you’ve not been trained yet.yes But by then during our routine programs it’s something that we always stress that every staff should be on the alert to observe for adverse events and report

I: ok

P: mhm

I: so with this is it only your facility that has not been, has not received the training or there are other facilities around

P: I wouldn’t, I wouldn’t say it’s only my facility

I: ok

P: because if there is a training then every facility staff are supposed to participate even if not all they can select one or two person from the facility, you go for the training then you will come and then share it with all the other stuff

I: ok

P: uhuh

I: so from, from the reason you gave meaning this is something that is being organized from the higher level and so do you happen to have you errh ever asked why training like that has not been organized for you people or has there ever been a peculiar reason why the training has not been organized for you people

P: I per say I have not asked

I: ok

P: but as I said we are always on the lookout because those allergic reactions they are not things that are strange to us their things that we encounter on daily basis as we work

I: ok

P: uhuh so if there is any this thing, any update sometimes they just update us the district disease control officer they can do updates meetings for us

<Internals\\IDIs PROVIDERS\\UEPI_02> - § 2 references coded [4.54% Coverage]

Reference 1 - 1.72% Coverage

I: Ok. So has your health facility received any training on adverse events following immunization?

P: We’ve not had training following on that.Because maybe mostly some, when you go for some workshop then they’ll just talk that it’ll come in how you’ll do about it; but training on…just to call training on adverse effects that is following immunization, no – we’ve not had on that.

Reference 2 - 2.82% Coverage

I: Ok. (Clears throat). So do you happen to know why your facility has not received any training on adverse events following immunization? (Coughs).

P: Well…I I don’t think, and even the whole of Bongo I’ve not seen them calling for any facility on training because if there is a training on that, they’ll definitely call us because our facility to render service. But as I said, it’s other trainings – when we go for other trainings then they bring those on board (Ok). So maybe I guess maybe it’s because of that that they’ve not just called for a training specifically for that; but if they’re yet to call (Ok), I can’t tell.

<Internals\\IDIs PROVIDERS\\UEPI_03> - § 1 reference coded [0.47% Coverage]

Reference 1 - 0.47% Coverage

I: ok … so has your health facility received any training on adverse events following immunization

P: yes

<Internals\\IDIs PROVIDERS\\UEPI_04> - § 1 reference coded [0.77% Coverage]

Reference 1 - 0.77% Coverage

I: has your health facility received any training on AEFI’s?

R: yes.

<Internals\\IDIs PROVIDERS\\UEPI_05> - § 1 reference coded [1.35% Coverage]

Reference 1 - 1.35% Coverage

I: alright, thank you sir. Has your health facility received training on AEFI’s?

P: yeah as I said all those of us involved in that exercise, we are all trained, all the health staff like the nurses, the community health nurse and the dispensary, we are all involved in the training.

<Internals\\IDIs PROVIDERS\\UEPI_06> - § 1 reference coded [2.07% Coverage]

Reference 1 - 2.07% Coverage

I: okay okay, has your health facility received training on AEFI’s?

P: mmhm just as I said during the SMC, the one who was in-charge of it that is that disease control officer he was part of the training and the volunteers who were suppose to do the work aaaha just as I said the disease control officer together with those who were to do the exercise SMC work, that’s the volunteers, they had the training, they had some training and I want to believe it captured the adverse reactions that they should be expecting when they are giving the children their the the the the drugs.
