# Supplementary material for: Optimising reporting of adverse events following immunisation by healthcare workers in Ghana: A qualitative study in four regions
Source: PLoS One. 2022 Dec 20;17(12):e0277197. doi: 10.1371/journal.pone.0277197 (PMC9767370; doi:10.1371/journal.pone.0277197)
Supplement: S1 Data — (ZIP) [file pone.0277197.s001.zip › Minimal data/S10 Suggestions to improve over all reporting.docx]

**Name:** 17. Suggestions to improve over all reporting

**Description:** This node contains suggestions on how to improve general reporting of AEFIs.

<Internals\\IDI EPI\\GAEPI_01> - § 1 reference coded [18.63% Coverage]

Reference 1 - 18.63% Coverage

I: okay. We are almost done but before we conclude we will like to know if you can please tell us if there is any other issue you will like us to know with regards to the reporting of AEFIs in general

P: [inaudible] reporting of AEFIs ‘erh’ is not as we all want it to be I mean ‘erh’ the reporting rate I mean is low and this’s also more important at the time that EPI is achieving very high successes actually in immunization. Diseases are really going down or [inaudible] so now what people might see are the, I don’t know…adverse events come from after immunization, but if the diseases are not there [inaudible] so if I think ‘erh’ it’s very important that as a country, as a service, as a programme we focus as to how to strengthen the AEFI I mean ‘erh’ this thing to ensure that we can pull the situations as and when I mean ‘erh’ they occur without letting [inaudible] disease allow AEFIs now to become the I mean the champions. Probably people are not seeing measles, they are not seeing polio, diphtheria; what they might see is some fever [inaudible], some swelling following maybe a vaccination which might be giving a very big picture as if that one is more serious than the disease measles or the disease polio-myelitis. So I, I will think that ‘erh’ there should be a well prepared system all over the country [inaudible] all levels and also educate the broad masses about I mean ‘erh’ AEFIs, what they should do after vaccination in case any event occurs actually in their ward. No matter how mild, whatever it is they look at it because some, some, some things can begin mildly and end up I mean [inaudible]] so they should just report to their various sites.

<Internals\\IDI RHMT\\GARI_01> - § 1 reference coded [4.45% Coverage]

Reference 1 - 4.45% Coverage

I: so we are almost done with our interview. The last thing we want to find out if there is anything you want to tell me in *general* with response to the ‘erh’ , regarding adverse effect following immunization

P: yeah, if really we want people to report AEFI, then funds should be set aside. That the, region this is your funds for AEFI, if even you will not give it to the region at a go, we inform the regions that there is funds set aside. Anybody who re, any district that reports we just give it to you to do your follow up. I think it will, people will gather this thing to do it.

I: is that all?

P: for, that is all that I can say for now

<Internals\\IDI RHMT\\GARI_02> - § 2 references coded [21.53% Coverage]

Reference 1 - 6.86% Coverage

I: so having said all these, we will like to know your suggestions for improving the process of reporting AEFI

P: uhuh, yes. I think that, ‘erhm’, you see most of the time too ‘erhm’ getting access to the forms is also a problem. So if the forms are made available in all facilities whether we are doing any program or not, the forms are there. And periodically they are ‘erhm’ refreshed on how to fill the forms and also when they fill the forms and they send it, feedback should be given to them, feedback should be given to them to know that oh this thing that we sent, you know, this was the results and then also ‘*erhm’*, periodically the staff should also be ‘erhm’ refreshed and reminded about AEFIs and then how they, they can report. And maybe if the process is not too cumbersome for them, I think it will also help.

I: is that all?

Reference 2 - 14.68% Coverage

I: before we finish we will like to know if there any other issues you will like us to know with regards to the reporting of AEFIs in general

P: ‘erhm’, for AEFIs, I think we still need to’ erhm’ talk about it, because it’s very important if we want to make meaning of the services that we give out. So periodically, like I said before, we need to bring the front liners together and then you know remind them about AEFIs, how the process is and how they can, no! How important it is because I know it will help us to you know, know most of the things about the vaccines that we are giving out, if there is any problem, we report and corrections will be done. But if we keep quiet over it, like currently there is no campaign or national programme going on but, ideally anytime they are going for immunisation session, they should go with their forms. If there is any problem they have to fill and bring it. But if it is not, it’s not a campaign, everybody is sleeping and we forget about it, you know. So we need to get people, you know awaken to know that, even though if there is no, it’s when there is a campaign that headquarters and everybody you know is talking about it. And most of the time too they don’t even give us ‘erhm’ [chuckled], very difficult to get this ‘erhm’ antidote for them. When we are doing a campaign, we will realise that they will give hydrocortexin may be one, one vial to a whole team or the whole district to go and use. So people also don’t see the use. So if we want to you know get the ‘err’, AEFIs and then know if there are any problems with any vaccine then you also have to sit up. Right from headquarters, to region, to the district and to the facilities. So that we will make sure that the right thing is done and get the right information about the vaccines.

<Internals\\IDI RHMT\\NRRI_01> - § 4 references coded [31.39% Coverage]

Reference 1 - 9.00% Coverage

I: Ok, ok, “Arrr”, please let me know your suggestion for improving the processes of reporting AEFI?

P: I think it about orientation, orientation of our staff. What is the question? The process of?

I: Suggestion for improving the processes of reporting AEFIs.

P: Is about orientation, to every health worker who matters, like she said, AEFIs is not due to anybody’s carelessness in as much as this job is concern. So that when people have it, I think the most important thing that is that we talk we accept it as a normal happening and then we treat the health workers as such as humanly possible as we think we can rather than scolding them, for me that is the number one thing that prevents me from reporting AEFIs, the idea that if I report it I will be asked questions, how did it happened? Why did I make it happened? And a whole lot of things.

I: ok.

P: So if we can give that orientation to our staff which we have been doing oh, we have been doing it but you see change does not come in a day, change will normally take some time to occur.

I: Ok.

P: So I belief that giving orientation and psyching ourselves up, am talking of those of us who have control over a number of people who work under us and making us accept whatever I mean sometimes are minor I belief over 90% of the cases is not usually the faulty of the health worker but then it is the reaction of the vaccine that has been introduced to the, the reaction of the body to whatever. So definitely you can temperature rise and all of those things happening that should be made to feaster among “eerr” what we are telling our health worker so that they will also be free and feel confident to report when they find any of them.

Reference 2 - 3.73% Coverage

I: Ok, Thank you very much. Has your regional unit receive AEFI report in the last year?

P: She has the report. But I belief, I belief the have reported but as to the numbers that’s what the problem is and as to the number of districts reporting that’s where the problem also is. Whereas some districts will religiously try to report to the next level, others, I don’t know whether to say they don’t see the need or simply put they are not seeing but as for the question or they are not seeing that one is out of the way.

I: Yes.

P: But I think is the edge to really report it taking into consideration some of the earlier things that we have reported, we have spoken about is what would make the trick.

Reference 3 - 14.94% Coverage

I: Ok, Do you have more to add? Please tell me other issues you would like me to know with regards to reporting AEFIs in general?

P1: AEFI.

I: Other challenges.

P1: Other challenges, “hmnm”, “yaah” the other challenge I will like to put to whoever, National or whoever is “errr” you by asking about the way the data is generated at this level, soft either soft copy or hard, it will sort of demand at least “errr” “errr” a computer or something basically for that if you fill AEFI itself is important, let us have something “err” a computer, or something device devoted for that “err”.

I: Ok.

P1: (Laughing) for that purpose “errrrr”.

I: Ok.

P1: Unlike, it comes a computer is there been used for all other programs and then you key inn, you want to update something or you want to do something.

I: Ok.

P1: Then you have to sort of let that person be free or is free before he puts it for you. But AEFI as it is, we will want it to “errm”, from district level to region or to national and to be softcopy we have to have a computer for that. I didn’t know.

I: Do you have more to add?

P1: (Laughing).

I: Please sir, can you add more?

P: Other challenges?

I: Yes, any other issue concerning AEFI, any other issue.

P: For me I think, from the previous responses I think some of them have come up.

I: Ok.

P: May be a lot of them.

I: But we want all of them.

P: (Laughing) that is the others that are not coming now. Because as in the challenges as in how it can improve, we spoken about the need to, the other challenge will be orientation of even our village volunteers may be the CBS volunteers.

I: Ok.

P: Because we have, we have all of a sudden seen a dip in reporting from the community level.

I: Ok.

P: That is because, even the common most important tool for the CBS volunteer to report on is not in the system, we can’t provide it that is as in the register.

I: Ok.

P: We can’t provide, so sometimes it makes also the issue of may be even reporting because this village volunteers are better placed to have more contacts with community members than we do.

I: Ok.

P: And they are usually the front liners when it comes to reporting from that level upwards.

I: Ok.

P: So when he does not even have that that tool that I will refer to as the motivating tool.

I: Ok.

P: Because that is what he or she works with.

I: Ok.

P: Where the follow doesn’t have access now to it to do some of the normal reporting then it poses a problem, so that makes it that dents our image as service. How come that all of a sudden for so many years about 3 years we can’t print this to ginger up the volunteers because that is what makes keeps them working but were it is not available then it serves as a de-motivative factor for the system.

I: Ok. Thank you very much.

Reference 4 - 3.72% Coverage

P1: I think orientation as he mentioned, would have been the best way to get all the staff at the same level, I remember 2015 we went to “errrr”, Dodowa for a workshop was to cascade down to “errrr” the lower levels, it never came.

I: Ok ok.

P: “Yaah”, that was in 2015.

I: 2015.

P1: All the regions were assembled there, they took as through with ADFI to sensitize or orientate our staff, why is it that they are not reporting, we have talked “aaaaa” since 2015 and is still like that, we will talk talk with interviews everything and is still like that, we haven’t orientated, we haven’t put the staff down this, this, this, you see and if we we it was a nice program but it got stacked somewhere.

<Internals\\IDI RHMT\\NRRI_02> - § 2 references coded [28.03% Coverage]

Reference 1 - 20.81% Coverage

I: Please let me know your suggestions for improving the process of reporting AEFTs?

P: For me, the strategy is simple, we should train people, hum train people and especial mostly people who are directly ‘errrr” “hmm’ managers of vaccine, people who are “ayiii” ‘mm’ vaccinators, all people involve. Disease control officers, surveillance officers “errrrr” EPI staffs, community health nurses, public health nurses all those matter in “errrrr” dealing with vaccine and vaccine preventable diseases and management “ayii” given of care, given of vaccination they should all be properly trained and as they are properly trained, the have to be “errrrr” periodically monitored, monitored frequently and reminded to report adverse effects. And the community level too, you know and durbars, and radio discussion we have to discuss these things so that people will be aware that vaccines are supposed to protect them but once in a while you may get somebody getting a reaction so if it so happens they shouldn’t sit at home, they should report this. So our education should go on widely and then individually. For instance if am vaccinating ‘errrr’ your child I should educate you that because every drug has its adverse effects and then that is why ‘errrrr’ vaccinator are advice always to read the literature, “ahaaa” the literature of the manufacturer so you let this person know that am given this child this vaccination in case you realize ABC in the child, come back and then we give you treatment you know is an adverse effects. If that is not done they wouldn’t know but if this is done they will report so as individuals and as “errrrrr”, public as a whole, we all need to be educated on vaccines and its “eff” and their adverse effects so that the reporting can flow and then “errrrr” as managers we need to also to do what? To monitor and track all these things and also do we even get feedback, we are not getting feedback with the few cases we even reported, who has investigated, what have they seen, it is actually an adverse effect or just a co-incidental “errrrr” condition that the child or the person acquired because I can be harboring some some some some “errrrr” con disease condition in my body, you know but is not showing at the time that I am been vaccinated, then after vaccination for instance if I have malaria and then here is a high fever due to malaria, it could not be due to the vaccine so those people who are also helping as to investigate. They should give as feedback so that we are aware but if “errrrr” we are reporting and we are not seeing any action from the upper level, it means that we are wasting our time, you know sometimes that is why people will not even report. “Ahaaa” you understand. After all even if we report, what is it, is not the fever, let’s manage our thing, is not rashes let manage get some corticosterone “errrrr” cream for this child and things like adrenaline “errrr” hydrocortisone you keep giving and then you also “errrrr” relaxed you know. So we need feedback “errrr” we need feedback. So trainings, education, feedback from investigation, you know and then “errrr”, when people report, they should rather be encourage to do more instead of may be aportion some blames and things like that is know and then we should also be in the position when people react “errrr” I mean to be able to allay their anxiety and that kind of thing. We don’t pray for the worse once that can result to deaths but in case of the severe once we need to be what “errrr” ready to allay the families anxiety, we should be supportive, I mean we shouldn’t demand money to manage these cases. We should actually go right away and then manage these people get them errs sate instead of experiencing complication and deaths if not it can even mad our vaccination program you understand.

Reference 2 - 7.22% Coverage

I: Thank you, we are almost done, please tell me any other issues that you would like me to know with regards to reporting AEFTs in general?

P: “Hmmm”, I think that “errrr”, we need to also do what “errrr” continuously at all levels be reminding ourselves about AEFIs and then “errrr” when we are doing that then we know that it will be like part and parcel of us that we will not see it as “errrr” something that we should actually play down with because if at all level we continuously remind ourselves, we continuously monitor and like we are here and we see monitors come down from the higher level to monitor about AEFI, and then onward we also pick it up, we also go down to the lower level you know it keeps, it keeps us been reminded and then we feel that this is something we should pick it up and report for action. “Hmmm”.

I: Thank you very much. But do you have any issue concerning vaccine safety? Any other issue concerning vaccine safety in your region?

P: Vaccine safety in my region, apart from the power off, the occasion power off, well I don’t know, I just want to say that “hmmm” I don’t have any big issue but “errr” people giving vaccination should, should keep on be (noise) the should always be updated when there are new new new issues coming up, new policies, new vaccines the should be updated.

<Internals\\IDI RHMT\\UERI_01> - § 2 references coded [14.39% Coverage]

Reference 1 - 3.81% Coverage

M: what would be your suggestions to improving the process of reporting adverse events following immunization?

R: my suggestion is that we should make sure that all the staff who are doing the vaccinations are trained on the job, then we make the forms available and then we continuously carry out refreshers and should also monitor and supervise the work they do so that they can report.

Reference 2 - 10.58% Coverage

M: what other issues would you like us to know with regards to reporting adverse events following immunization in general?

R: well I don’t know what to say but I think that if the logistics are available and readily handy for people to use it will also go a long way to help in reporting of adverse events following immunization. For instance the forms I know it is usually carbonated one where you tear and leave a copy. I don’t know the last time forms were supplied. So if they forms are there, they supervisors in the sub-district levels should make sure that any time staff are going out for immunization, they carry some along and there should be some at the facility level and then we should also embark on education to tell people that if anything happens and they think it is related to the vaccination they should come and report. I mean once it happens when they come, there will be some counseling and if it has nothing to do with the vaccine care givers would be told to alleviate their fears but it has something to do with the vaccines they will be taken care off.

<Internals\\IDI RHMT\\UERI_02> - § 1 reference coded [16.41% Coverage]

Reference 1 - 16.41% Coverage

M: what other issues would you like me to know with regards to AEFI’s in general?

R: I think the issue of AEFI’s has to be taken very seriously. With regards to the fact that several vaccines are introduced day in, day out, the roll out of vaccines is quite rapid , knowledge on vaccination and immunization among care givers is quiet good- some of them are able to tell you much about the vaccines, the time they are taken and all that, the side effect and those things. So if we have to link it to the fact that side effects will be reported in the contest of AEFI’s. They are found wanting. So sensitization is key, to be able to sensitize care givers and health workers alike. So they will know what exactly it is and what to do. We need to also know that at the points of suspicion you don’t assign any causing just like in any surveillance issue. If you begin with suspicion it becomes a probably case. Then it goes into confirmation you don’t begin thinking that it is confirmed, No. So at that level you should be able to open the net wide so that you can catch the events or be sensitive so that if it is there you catch. We are not talking about specifics here at that level it is not necessary. So training has to be taken across board. I am even thinking they should have a focal person at each level for AEFI’S because of the importance we attached to it and because of the fact that the reporting is low, feedback is not forthcoming and all that. We need to also find out because at the central level they should be able to give us concrete information regarding some of the decisions taken. They should be able to tell us periodically that yes we have been able to receive 30 cases of AFFI’s with regards to this vaccine so that when they experts committee sat on it they realized that some of these were unrelated and some were coincidental. This number or proportion was due to human error or may be vaccine was requisition mistake by the health worker or something and these were actually associated with the vaccine and because of that we have taken this action or that action but if we don’t hear that then I actually don’t see anything concrete in it because it is like reporting for reporting sake but the most important about surveillance is that information for action. The data we generate feed into the information and that is used to take public health action. So in the absence of this I don’t know what it means, so sensitization, training and feedback very essential. You would have realized that it is intersectorial nature. It is not an all health worker thing. Ghana health service is there, FDA is there. In the experts committee level there are other experts there. So it is much wider than a health thing. So we should be able to collaborate and state clear the roles the stakeholders would need to play and all that. Otherwise it may go there and one agency is left to take care of everything, may be that is making us not get the feedback.

<Internals\\IDI RHMT\\VRRI_01> - § 2 references coded [26.16% Coverage]

Reference 1 - 23.23% Coverage

I: ok

P: because they not trained on it.

I: ok

P: Because they are not trained on it. So I think errh there should be a proper training for all the people that do the vaccinations so that they would be able to report.

I: Ok.

P: Ahaan, because the system is there, the reporting from facility to district, errh sub district to district to region, national. The system is there but sometime how to monitor it because some, some, may be like the clear definition, what to report, some of them may also not be too sure on some of the things to report. So I feel errh a lack of tracing of the staff that actually do the vaccinations on AEFI is one of the reasons we having how reporting rate.

I: ok. Any other please?

P: Emmh and other ones too, is like maybe like the communication aspect.

I: ok

P: That to sensitize caregivers about the AEFIs because all you know, some of the caregivers too if there is AEFI all that they do is…., if is even severe, and they have not reported they feel ok; I have gone to the vaccination session and this happen next time I will not send my child. Because they also don’t know that may be this and this are the things I have to report on.

So the second thing I will think is errh, we should also errh think of sensitizing the caregivers.

I: ok.

P: Ahaan on the AEFI. When they know that, ooh these things can happen, these ones are expected, though they talk to them at the clinics but I don’t think those education is errh, very, very errh, enough or adequate,

I: ok

P: so there should be more or we should step up education on the AEFIs to caregivers.

I: ok. Emmh, does your unit

P: yes

I: have in place the mechanism to monitor AEFI cases in the region?

P: errh, yes, we have a mechanism but hern, with challenges.

I: ok.

P: nhmm, we have the mechanism with challenges.

I: What are they please?

P: Because every, every year, we put in plans for monitoring

I: ok

P: ahaan, but sometimes you, the times you would want to go for the monitoring, the funds are not available for you to go and do the monitoring. So I think that’s one of the challenges.

I: ok. Ammh, could that also be a factor, contributing factor to the low reporting from, from the facilities?

P: That we are not able to do monitoring?

I: yes

P: Errh, yes it’s also part of it.

I: Ok

P: Because if you visit the district that may be you have to get at least one or about 10, a district is supposed to get about 10 and you report only one or two, what are the challenges? If we also visit them and they also look at the sub-district, may be this sub-district you are suppose get at least three in the year. So what are their Challenges? I think the supportive supervision also plays a role.

I: Ok, thank you very much. What do you think, again what do you think we can do in general to, to improve upon the reporting of AEFI?

P: Ok, errh to me, what we can do, just like other surveillance systems, you take like AFP, Measles, Yellow fever, other surveillance system like these, errh we need to first train, do the training of regional officers, district officers and sub-district facilities officers, then supportive supervision at all levels; national, region and districts levels. Then communication, communication errh, like the districts, now we have a lot of radio stations just to help districts to organize discussions. I know a lot of programs sponsor discussions on radio, so if the sensitization is there, we can incorporate AEFI monitoring, but some sponsors too, if they sponsor a program, they don’t allow the staff to put in other areas

I: ok

P: in their program but some are flexible. But if AEFI system can also… or the EPI system can also look at it, support districts to errh, advocate on the need to report AEFIs at the district levels.

I: ok

P: then we build on that one to do… demand errh, to request for other demand, like to bring people for vaccinations, and other things. I think it will help.

Reference 2 - 2.93% Coverage

I: Please do you have any other issues you think is important to…

P: The AEFI?

I: Yah with respect to the AEFI?

P: Yah, like you mentioned that do we give feedback to the district or the facilities, and I mentioned that we have not be getting feedback from national. If there would be a system when they go, national look at them, which ones are serious, which are not serious, and they give feedback. If it’s serious they should give feedback, even if it’s not serious they should give feedback.

<Internals\\IDI RHMT\\VRRI_02> - § 4 references coded [35.00% Coverage]

Reference 1 - 3.16% Coverage

I: ok

P: it might also be a problem or the forms are there but they don’t orient them well

I: ok

P: on how to fill it.

I: Ok.

P: Ahaan! There is a need for somebody to facilitate this for especially the newly qualified ones.

I: ok.

P: They might not have any knowledge about these things so, it might also cause erh, they not filling or informing the next level.

Reference 2 - 2.49% Coverage

I:ok. In view of these possible causes, what do you think we can do to improve upon the reporting of AEFIs?

P: We just have to keep doing our orientation to the newly ones that come into the system and giving updates to those who are already aware and they are not doing it.

I: Ok.

P: Yes

Reference 3 - 5.43% Coverage

I: Any other suggestions you have that you think we can bring on board to help improve upon the reporting?

P: These are the two key areas, just the training, informing them how to go about it. Is…., the matter is if they don’t know how to go about it. They just sit down and, and look on. So I will suggest that, there should be constant training for the newly qualified ones and then refresher for the others, or reminder or we also have to intensify our supervision.

I: ok

P: If we get to the facilities we should enquire about these things and find out why they are not doing them, then we can take it from there.

I: Ok.

P: Yes

Reference 4 - 23.92% Coverage

I: Thank you. Is there any other issue you may want to say with respect to AEFIs?

P: I think the general public also need education.

I: ok.

P: Yes, so that if a child is given any immunization at any level, errh they need to observe that child or the… even if it’s an adult, ahaah, they need to observe the situations and then inform the nurses or the health workers that are around. It is they that are supposed to report first. Like this outbreak that we are all facing now, if they don’t report at the facility, we can also not pick the disease as early as possible and treat. So the main intervention will come from, how they are reporting.

I: ok

P: yes

I: Ok. Lastly, erhm, what is the link between your outfit and the Food and Drugs Authority with respect to adverse event?

P: I think the meeting that we held, not the recent one. There was a meeting before that, they were also part of it. There was a complete team from Ho, the Volta Region and other regions. We met at Dodowa and we were supposed to comeback as a team and do the training and we did that. That was about two years ago before the recent training that we had.

I: ok

P: So there is this relationship between us and the Food and Drugs Authority. And then we, we are doing our reviews, we invite them;

I: ok

P: ahaan, both the half year and then annual. They are always at our….

I: Ok.

P: Yes.

I: Alright, please do you have any other thing to say?

P: Well, we… what we are concern about is the EPI in general, we have a lot of err problems, the cold chain. Some of the facilities don’t have proper fridge, vaccine fridges to store their vaccines.

I: ok

P: so you need to go from one end to the other and then, may from the field back to wherever the chain might broke, be broken and then we have these adverse events. So we need to store emmh, proper vaccine fridges at all the facilities to get potent vaccines for our clients.

I: Which actually implies that logistics

P: yes

I: might be a major contributing factor?

P: Yes, Yes.

I: Ok and that is some of the situation we have out there,

P: mmh

I: Is that the case?

P: Yes, it is.

I: ok

P: When you go out some are broken down, they need repairs. We don’t even have…, as a nation we don’t have enough of the fridge mechanic to do the repairs.

I: Ok.

P: Yes, we are even lucky we have one in the Volta region but other regions, is only headquarters that goes there to do erhm, the repairs, so the advocacy now is, they should recruit emmh, competent

I: ok

P: fridge mechanics who could help us in terms of the broken ones, if we don’t even have the money to buy new ones.

I: Ok.

P: Yes.

I: Any other thing please?

P: We continue the updates and then the trainings, like I mentioned, we will do our bit to help the, the staff to do their best.

<Internals\\IDIs DHMT\\GADI_01> - § 2 references coded [22.34% Coverage]

Reference 1 - 15.00% Coverage

I: So can you give us some suggestions to improve the reporting of AEFI?

R:I think training in terms of reporting of AEFI should be done regularly not to higher authorities but to the community health nurses directly because when people come for training they tell them to go and tell the others but they don’t go and tell them so go and sit in their offices and the knowledge is with them only it doesn’t go down , so training should be done directly with the Community health nurses because they give the injections, they are with the others and they see the children directly , so training should be direct. And education, public education on reporting of AEFI should be intensified we don’t know the reason why we are to report AEFI, whether that particular batch of vaccines is causing that AEFI so that it can be withdrawn but we don’t understand the importance generally.

Reference 2 - 7.34% Coverage

I: Are there any other issues about AEFI in general that you would like us to know about?

R: As I said there should be public education on the importance of reporting AEFI’s and training should be done directly, you call for training and administrators are the ones coming, the CHN’s should be the ones involved because they do the direct jobs to have the training.

I: Are there any other issues?

R: No, I think I have said all.

<Internals\\IDIs DHMT\\GADI_02> - § 3 references coded [36.22% Coverage]

Reference 1 - 12.09% Coverage

I: Can you give us some suggestions to improve the overall process of reporting of AEFI?

R: Now we have apps, Ghana health service should have a platform where you can easily dock in and if there is any report it should be referred to the sub district and steps will be taken that’s the only way I think to improve reporting now we technologically inclined paper work is no more part of the system, because the mother might call you at night and say y child is sick what should I do, you need prompt attention, that’s my personal suggestion I don’t think it’s an additional cost you just download the app and its n your phone.

Reference 2 - 9.75% Coverage

I: Are there challenges regarding AEFI that we should know about?

R: Sometimes we don’t know where the forms are lying, documentation is very poor or may I say people keep moving the frontline workers keep changing their post and the junior ones may not be conversant that something like this exist, if there is a system where we do refresher training for the graduates but funds are not coming to do training. As at when if there is an issue then we do on the spot training and that will not be the best.

Reference 3 - 14.38% Coverage

I: Are there any other issues?

R: There are no issues since there are no cases but one issues is the availability of the forms, even normal paper for work is not there how much more forms even though they are rare but important to follow.

I: Are there any other ways of improving AEFI?

R: There is a lot of information the GHS website but a lot of the people don’t go there but if the information is put there and we continue making people aware, and if you are in doubt you should just go there and should always be updates or hotlines where you can call for health information, even if it’s not on the internet you could still get access to it and read. We don’t have money for monitoring and supervision and as and when we get we will work.

<Internals\\IDIs DHMT\\GADI_03> - § 2 references coded [28.46% Coverage]

Reference 1 - 11.11% Coverage

I: Can you give me some suggestions to improve the overall process of reporting of AEFI?

R: Maybe if the form is simplified, if we are able to simplify the form then it might make it easier for the health worker to probably be able to complete in a shorter time and they will not find it burdensome, so I think the form has to reviewed so it captures the key information needed and that will make it user friendly and the staff would complete it.

I: Is there any other suggestions?

R: Definitely as new people come in, there is the need for reorientation or training for staff so I would say regular training on AEFI form because every now and then we get new staff coming in so I would say regular basis, there should be that orientation so that every point in time everyone is informed on the need to complete the forms.

Reference 2 - 17.35% Coverage

I: So is there any other suggestions or issues you would like us to know?

R: Well I think it’s just the reporting so once the reporting is done adequately that should improve the system other than that I don’t have any major comment but actually we do have few adverse events following immunization in my former district but some of them have been quite significant, as I indicated if the form is made a bit simpler with few indicators on it that might help to fill out the form and then making the form available which is the responsibility of the district directorate to do, so as much as possible we make sure that the forms are available and expect that events like that occur there would be reporting and the other thing is that what happens to the care giver who doesn’t come to the CWC in the first place but goes to a facility to report, we had a number of private facilities where we leave the forms with them it only to go back and they tell you they don’t know where the forms are so I think going forward the clinical side significantly brought on board especially the outpatient department so that when the care giver comes with report of AEFI they will be very much aware and fill out the form and it will go a long way to improve the system, involving the clinical team.

<Internals\\IDIs DHMT\\GADI_04> - § 4 references coded [27.59% Coverage]

Reference 1 - 9.09% Coverage

I: So can you give us some suggestions to improve the reporting of AEFI?

R: The very first thing I will say is the do periodic sensitization of health workers so that it will become a culture to let the mothers know that is you see an adverse event please come back and also people in position as me should do monitoring and be sure that they are doing what they have been taught and also educate the mothers, it should be part of the counselling session and if there are difficulties in filling the form too it should be addressed.

Reference 2 - 6.58% Coverage

I: Are there any other issues and challenges about AEFI that we should follow up?

R: With the challenges I think we have to be proactive about adverse events and then we need to write the batch numbers so we can easily identify and then we also have to educate our mothers more to identify, if they are not aware they might not be able to report because it might become something else.

Reference 3 - 8.90% Coverage

I: So what about the challenges?

R: As I said there should be refresher training that everybody is in the known but if you come and ask me I might have so much on my mind and might not be able to tell. We should be all conscious about it because we need to be trained and refreshed we will report and supervision should be more, your supervisor should be like every month where is your adverse event reporting form and if they don’t understand anything they should ask questions that’s the only way we can get the cases.

Reference 4 - 3.02% Coverage

I: Do you have supervisors who are to go for these visits?

R: Yes , am supposed too but it’s not all the time, I think when we do these A,B,C,D we will do our supervision well.

<Internals\\IDIs DHMT\\GADI_05> - § 3 references coded [35.97% Coverage]

Reference 1 - 19.40% Coverage

I: Can you give some suggestions to improve training?

R: Ermm at the district level I don’t know the in-service coordinator but at the facility level I know the in- service coordinator so it’s like on our appraisal it is stated your weaknesses and your strength to upgrade yourself so when they see your appraisal forms yearly they will see the needs of the staff and will know whether the person needs training or not in the unit, pick it out so when there is a training they include you but unfortunately when there is a training they select the people to go and the category of staff to involve so if you don’t read on your own, do intense search and all that you might not know what is going on. Those who get the opportunity to go for the training when they come they disseminate the information, they organize some small in-service training for those who were not able to attend I think when that is done it might help.

Reference 2 - 5.69% Coverage

I:Can you give us some suggestions to improve reporting?

R: I think we should continue to educate them, sensitize them and also the in-charges, they have their conferences once a month and their management meetings and it will come to a point they will be all used to it.

Reference 3 - 10.88% Coverage

I: Are there any other issues concerning AEFI that you think we should know about?

R: My only problem is that, drugs have side effects and they should expect them, so if we see that are we to classify it under AEFI or we have to report on it or we see it to be normal, every drug has an information sheet where it will tell whether you will have nausea or vomiting so that where do we draw the line, would we classify that under AEFI or just a normal side effect. This has to be clarified so reporting can be done well.

<Internals\\IDIs DHMT\\GADI_06> - § 4 references coded [41.81% Coverage]

Reference 1 - 7.79% Coverage

I: Can you please give us some suggestions to improve reporting in general?

R: Reporting in general we see AEFI reporting as a negative report, it’s not positive there is something wrong, so people fear to report they may even identify some but they say oh I won’t report so we have to boost their confidence, there is no punishment for reporting. There is nothing to fear, it’s part of the process to identify challenges so that we can put in measures to check those things, if let’s say every vaccine and the type of AEFI to expect and also every drug and it’s AEFI so if we build their confidence aspect; you see the point, then they will also be willing to come forward but if we make the thing look like fault finding then why should I report but if we build their confidence level in that aspect and the have the capacity to be reporting, am sure they are getting the cases but the fear is that if they report plenty they will say am reporting, so they won’t report at all. That is the situation that I see.

Reference 2 - 7.32% Coverage

I: So what are some of the challenges you face with reporting?

R: You know it has to do with the capacity of the staff it has to do with the understanding of what they are doing and the importance of reporting on these findings and we try to make them understand but over the years they have been doing the same thing, people are used to some way of doing things then it becomes difficult but you know gradually with time they will change, it will take some time but as we go and talk to them it will get better and with time with some understanding and involvement, let them see you as a colleague, when you go to them and chat with them they will ask questions and when you explain to them they tend to be like ok so this is the reason why you should be doing A,B,C,D then they start. So we hope that if we are really able to reinforce our visits to them sit down and have discussions with them we hope they will really come out and start reporting.

Reference 3 - 19.36% Coverage

I: So are there any other issues about AEFI that we need to know about?

R: You know before this whole EPI thing became accepted, people were having their own how should I say it their doubt, but caregivers have come to understand that EPI is something that is helping their wards because seriously we could all see paralysis is 0 we don’t see children with that again, measles is almost 0 you don’t see a lot of measles again although the result of EPI is not tangible and you cannot feel it but when you go to the bottom level you can really know that this things are happening and they are positive and to me what we can do to reinforce the reporting so the care givers appreciate it and one thing too is that we don’t show concern, when you show concern to somebody that person also will pull up, but when the mother comes to the hospital for vaccination and she goes home and it is ended it means that am selling and you have come to buy and you are gone, if she comes to the clinic and you give vaccination then you take the persons contact then you call her ‘Aunty Adowa’ how is the child doing, then you alert her and she tells you(speaks in local dialect) when I brought the child he has temperature, fever and all that then you write it down then you report but we don’t have that personal contact you feel she has come for my service and she is gone but if you should have the contact and after they have come and gone you call to find out to check if the place is swollen because the mind might not be there and it is not everything you will see, so then she might tell you I touched the child and there was pain and the place was swollen then you can report so there should be that contact of the caregivers and I think when we do that we will make head way.

I: Is there any other issue?

R: No, unless you have some questions for me but I think the best approach is to have a good relationship with the care givers and when we do that we will get all the necessary information that we need from them, when we do those things even the defaulting rate will reduce and the mother will always be ready to come and see you, if you are concerned they will always want to come and ask you of your advice. So we can make it a policy that we should have the numbers of all the clients that you offer services to so you can do a follow up not only by going to their houses but by phone call too, so when you call to check on the child it is also follow up so I think when we do that it will go a long way to help.

Reference 4 - 7.34% Coverage

I: So with the forms do you send them from the district to the sub- district before they get to the facilities?

R: Yes we give them then they run copies, then now filling the form becomes a problem so when they identify the AEFI cases you have to go from the district to go and fill the form for them.

I: Why are they not filling the forms?

R: They feel they don’t have the capacity to do it but I think they can do it, they will tell you I don’t understand this place, I don’t know this so when there is a client you have to rush there and fil the form, all these cases that we get; measles, AFP and all that we have to go and fill the form. They see the form as complex, it’s just basic information to fill; child’s name, age and all that but they see it as tedious, you know you ladies ( both laughs) writing and filling forms for them it is difficult but we are building capacity in that aspect so we hoping that as times goes on it will be better.

<Internals\\IDIs DHMT\\GADI_07> - § 1 reference coded [13.36% Coverage]

Reference 1 - 13.36% Coverage

I: please we are almost done, the last question says, if there any other issues you will like us to know [door opens in background] with regards to reporting of adverse events following immunization in general

P: come again

I: if there is any other issue you will like us to know

P: hmm

I: ‘erh’ with concerning ‘erh’ reporting of adverse events following immunization in general

P: ‘erh’ I think it is a very, it is a very important erh programme that we need to take serious but I also think that if we make it electronic, ‘hmm?’ from the point of service delivery, it will, it will help. So that the person doesn’t need to come and do that paper work and leave it and lose it ‘uhuh’ ‘uhuh’. So if it is included in maybe in our phones and so on so that it is delivered to those who work at the lower level, then they can enter it straight away into the system; the DHIMS

I: Please is that all?

P: yes that is all. ‘erh’ ‘erh’ I think the immunization programme has come to further boost the use of the adverse drug reaction ‘erh’ adverse events and so it is not only done during immunisation. It is also done in all the other clinical this thing so ‘erh’ ‘erh’ it fits in very well ‘erh’

<Internals\\IDIs DHMT\\GADI_08> - § 2 references coded [14.43% Coverage]

Reference 1 - 7.33% Coverage

I: And please we would like to know you suggestions for improving the process of reporting AEFI

P: Okay my suggestion is that, first and foremost I think I went for a training at food and drugs board on this AEFI and they were having a format that is like having original copy, duplicate and triplicate on the form so whenever they fill instantly you get the same information on, it’s like a carbonated paper so it helps for the writing. At times the work load will be high on them that when they fill one they forget to fill the others to let u have, so if we have carbonated paper like that when they fill it they, maybe the colored copies, this color copy is for region, this color copy is for district will let them let us have it immediately ‘uhuhm’.

Reference 2 - 7.10% Coverage

I: So the last question says, if you could please tell us if there are any other issues you will like us to know [participant sighs] with regards to reporting of AEFI in general?

P: In general is what we are talking about. If someone reports to you and the person receives feedback it gin, it motivates the person to keep on reporting because the person will say that “oh, when I report they act on it they do this, they do this” so the feedback is needed and the way we report the format the way I was taking about it, if we can get duplicates and other things so when they write it once they will just tear the copies or the colors needed for the next level for it’s onward transmission. So I, I don’t have anything to say again.

<Internals\\IDIs DHMT\\GADI_09> - § 2 references coded [21.44% Coverage]

Reference 1 - 7.22% Coverage

I:so please we would also like that, like, like you to tell us your suggestions for improving the process of ‘erh’ reporting adverse events following immunization

P: ‘erh’ sometimes I think what happens is, sometimes the patient comes with those signs and symptoms and they are asked to pay. I mean if it’s something that they come and it’s free people will be willing to come. But when you come you have to go through the process and sometimes you pay. Because sometimes, I quiet remember some time ago they said we should ‘erh’ compile the list of all those who come and then send it to, to region for reimbursement but nothing was coming. So when they come ‘oh! Maame you go and see the doctor and let doctor do something for you and that has been the system. So it’s not encouraging people to [inaudible] yes. Because if I tell you that if you get the symptom come and then you come and I am not able to do anything for you, then the next time I will not yes encourage you to come. Hmm.

Reference 2 - 14.22% Coverage

I: having said that, if you could please tell us any other issues you will like us to know with regards to reporting adverse events in general.

P: ‘hmmm’ like we were saying if we send it to ‘erh’ the, the appropriate quarters they should also give us feedback so that we can use that one to also educate the mothers. Yes. As for education we do it. Whenever we are ‘erh’ giving immunization, we tell them the signs, whatever the person is expected to see. But the person comes in, there is not proper, I don’t know the proper treatment then the person will not come again. Yes. And, I think what we have been seeing too sometimes they are very minimal, they are not very serious but exception of the one I mentioned, the only one that I have really seen was very serious but the other ones may be the fever and all those things, those ones are treatable, so we just tell them but really ‘erh’ if they can do something about the payment, how the follow up when they come with the complaint or something. Yes. And then the training, we have to ‘erh’ really make it a point that we will emphasize on the training ‘eheh’ the community health nurses are the first point of contact. Yes.

I: the front, the front line nurses

P: the front line nurse, the front line nurse, yes. So we can also give them some training ‘hmm’, though they, they have the idea, periodically we should be reminding them so that they do the right thing. Yes. In fact, for a long time, you will not see any form being filled but I know that people come with all sorts of complaints, but nothing much can be done about it. Yes. The thing is, she goes and says I didn’t receive it what will you say. But me, I always tell them the slightest thing, if you even ‘erh’ they gave and the nail got broken, they should report it maybe that person is different. Yes. So… The suggestion maybe just training and then feedback and then something for payment for the treatment when they come.

<Internals\\IDIs DHMT\\GADI_10> - § 1 reference coded [10.92% Coverage]

Reference 1 - 10.92% Coverage

I: So we are almost done. The last question says ‘erhm’, if there are any other issues like you would like us to know with regards to the reporting of AEFIs in general.

P: General issues?

I: Yeah general issues on reporting of AEFIs.

P: Yeah the, the general issue I would just say is the, the frequency. I think we have a lot of mixed err, err reports, yes because some of the things are assumed and there is, and I think one of the issue too could be that ‘erhm’ sometimes the, the, the nurse would have to actually tell or inform the parent that try and look out for A,B or C. you see! If you don’t do that, then the parent too will not probably notice it. If you don’t know what you are looking for you will not find it. So if they are able to tell the patients or the mothers that, if you go out, if you go back home look out for A, B or C, then they will be able to pick those signs and even tell them ‘erhm’ the times you know that these occurs, those err effects occur then when you anytime you see anything just rush back. If you encourage that reporting from the mother or the mothers then I think we will be able to pick up a lot more cases and if we ensure that we also ‘erhm’ you know monitor the activities of the nurses a lot in terms of ‘erhm’ vaccinations and [inaudible] adverse events, I’m sure we will be able to get a lot more information from them and maybe with periodic ‘erhm’ review of the forms. Yes! Because almost all the time may be something new might happen. Yes! So when the forms are reviewed [inaudible]

<Internals\\IDIs DHMT\\GADI_11> - § 2 references coded [10.89% Coverage]

Reference 1 - 4.15% Coverage

I: so then ‘erhm’ we would like to then know your suggestions for improving the process of reporting AEFIs

P: we will still educate them [inaudible] so that they will do it.

I: is that all? ... I’m sure there is, you can give us more than that

P: ‘erhm’ and also we monitor their activities.

Reference 2 - 6.74% Coverage

I: so we are almost done. The last question says, if there are any other issues you would like us to know with regards to reporting of AEFIs in general.

P: no

I: oh! You can give us something. In general, how can we do or to help…how do you call it? The reporting of AEFIs in general.

P: how that one, there should be training

I: is that all?

P: yes!

I: okay then…

P: and then the feedback too should come early; from the national to region to district to the grass root.

<Internals\\IDIs DHMT\\GADI_12> - § 2 references coded [27.38% Coverage]

Reference 1 - 11.25% Coverage

I: so having said that we would like to know your suggestions for improving the process of reporting AEFFIs

P: yes, I think we, we even this afternoon what we said was our communication, we realized there are communication gaps ‘uhuh’ So in addressing some of the communication gaps, mothers should also be, they should communicate to mothers, mothers should know it is from day one that they give the injection up to the twenty-eight days ‘uhuh, so that is what we told them. So, but we think that one will help. Some of them are saying that they don’t understand the language and we are saying they should learn because anywhere you find yourself working at least familiarize yourself with…there are some one or two languages that you have to learn to help you do that. Then I think the second option too was because they have the youth employment people attached to them and they are coming from Ada here. They understand the language, so you can tell them what you want to say in English they translate it in like dialect.

Reference 2 - 16.13% Coverage

I: but before we go, we would like to know, is there any other issue you will like us to know with regards to reporting of AEFIs in general?

P: ‘erhm’ oh, Well we are trying, like I said there is no particular…you know some of these things because of the staff alteration so you train people, people move out, new people come so that’s why I said that you know when we are doing training like that we don’t have it so when we are lucky and region say oh! Do this then we have to make sure we don’t do one thing but you add other things. So that is what basically we are doing to make sure we help the district reporting these kind of…I even told them it’s even beyond, the AEFI is not solely on the EPI thing. It could be prescribers giving somebody a malaria drug, the person reacted. Like last I went out and I saw somebody, I was going somewhere and I saw the person, a pregnant woman who has taken off all the dress she was using the hand to scratch. So I have to bring the woman to the facility. So I was telling them that AEFI go beyond…fine, you are doing EPI but when you go on outreach ‘erh’ the home visit, don’t, don’t limit yourself to only the immunization AEFI but you should go ahead and look up to the extent where the prescribers or doctors have given medicine and people are reacting. So I asked whether the forms are available, they said all the facilities said they have. So I have about, I have got about two CHPs zones to just send the forms to them.

<Internals\\IDIs DHMT\\GADI_13> - § 1 reference coded [13.46% Coverage]

Reference 1 - 13.46% Coverage

I: ok we will also like to know your suggestions for improving the reporting of AEFI’s

P: as it is now, it think it’s ideal if you have an AEFI, you fill a form and if you look at the form…the reporting form, everything on it is important, everything is critical. So that, you will be able to reach the mother, you will be able to find where she is and try and you know…even do causality, establish causality and say well, this thing is because of this vaccine. And AEFI reporting is to help make the system better and it’s a safety measure and it’s to make sure that we are all safe as we administer vaccines to the populace so everything on the form is important. I don’t know what we can take out. And when we have incidence occurring on the job we need to report to our supervisors. So the next person up, you need to report to that person and that person in turn also reports. You know, to the next higher level. So for instance, if something happens at the sub-district level, it comes to district level. We also don’t sit on it. We also have to send it to regional level and I know the regional level also sends it to the national level and that’s how come, we are able to put things together and have a complete report for say the country. So it’s important that we report. I don’t see which part of this process that is unnecessary. You know! I don’t see how we can simplify this. But I think it all boils down to supervision. Supervisors should make sure that their subordinate officers are doing what is expected of them. We shouldn’t have any problem if supervision is…you know! Optimal.

I: is that all?

P: yeah! That’s what I will say

<Internals\\IDIs DHMT\\GADI_14> - § 3 references coded [18.75% Coverage]

Reference 1 - 3.05% Coverage

I: So we are saying in general why do you think they might not report?

P: Why they might not report then you want me to guess [inaudible] I wouldn’t want to guess…

I: Okay then we would also like to know your suggestions

P: I told you I cannot stay in heat for so long…

Reference 2 - 9.41% Coverage

I: So please we would like to know your ‘eih’ suggestions for improving the process of reporting Adverse Events Following Immunization

P: Well to, to , new, new, new, new, new, nurses come out after training so I think newly posted nurses must be given some orientation, newly posted nurses those in the system already they know. The newly posted ones must be given some orientation on the importance of AEFI and the need to, to report them.[participant fanning herself with a paper in the background] And they should, their in-charges those who are there with them must go to the field with them and be there to supervise and make sure that adverse events are being reported and that the form should be available. If the forms are not there it will be difficult for them to even if somebody comes to report, so the forms must be there.

Reference 3 - 6.29% Coverage

I: So we are almost done with our interview but before we go, we would like to know if there are any other issues you will like us to know regarding the reporting of AEFI in general.

P: As I have already said the newly posted nurses must be given some orientation and the in-charges must make sure that they are on the field with them so that ‘erhm’ there will no missed opportunities and the form that they use to report them must be readily available.

I: Is that all?

P:[Participant fanning] And they should be committed, they should learn to be committed.

<Internals\\IDIs DHMT\\GADI_15> - § 2 references coded [18.77% Coverage]

Reference 1 - 12.23% Coverage

I: oh, okay. We will also like to know your suggestions for improving the process of reporting AEFIs

P: okay basically, one: we need to, there should be forms available. Okay! For immunisation, day in, day out new updates are coming so if you don’t get any opportunity to send these updates to them it becomes a challenge. So there should be forms for training which when we are doing the training AEFI form will also be part of it. There should also be funds “erhm” available at the district level for effective supportive visits to the CWC site. It’s not forth coming. It was recently that an M chain fund is coming but that one is once in a quarter. So all of us can move out once in a quarter which is not the best. Then thirdly, the, the community health nurses we need to advice ”erhm”, “erhm” let them know actually that the fact that they are reporting is rather helping to strengthen the system. We need to let them do away, to alleviate that fear that when they are reporting, oh! When you are reporting, then that means that I am not doing whatever I’m supposed to do right.

Reference 2 - 6.54% Coverage

I: okay, We’ve come to the end of our interview but before erhm we conclude finally, we will like to know if there are any other issues you will like me to know erhm regarding the reporting of AEFIs in general.

P: mhm, basically, it seems I have said, have said it all. There is no any major issue apart from what I have said because one good thing about mine district is that, the moment they get something like that, they record a case like that they call for more technical assistance even from the technical level and then from public health level too and that’s a good thing.

<Internals\\IDIs DHMT\\NRDI_01> - § 2 references coded [18.72% Coverage]

Reference 1 - 6.66% Coverage

I: So what would be your suggestions as to how we can collectively improve the reporting of AEFIs?

P: Me I think the continues counselling and education on AEFIs will help because something that should be done every day during immunization session or after before you give you let the mother understand that I’m giving the child this this and after that this this is what and what you may experience. But at times the mothers you even educate them and counsel them after that you ask her to repeat what said and some of them will sit there and they can’t even remember what you said or you were telling them but then I think we continue with our education on it and encouraging mothers to come and report back whatever that has happened we will be able to get good results.

Reference 2 - 12.06% Coverage

I: mm mm we are at the last stage and I just want to know what are the issues that you would like to add to what we have just discussed generally. Any additions?

P: Quite well, my issue that I have is that sometimes to staffing in the district is very poor, we don’t have enough staff so sometimes the work pressure on staffs too we go to – this thing you are alone you have to carry so many activities and those things sometimes is is also something that may be make them not to do effective education let me put it that way, they do but maybe it won’t be that effective m hmm so our staffing [babies crying] in the district we don’t have [crying] most of, mostly community health nurses are not there, you go to a particular this thing and the people there so I think the staffing if we are able to get enough staffs then the work too will…

I: For you, the staffing is your major problem

P: eeh if we get staffs and hmm and the logistics too to run the activities. Talk of motor bikes and other… [crying]

I: You at this unit, I know that you deal a lot with immunization issues.

P: yes so does why am saying the logistics too -----

I: Is a major hindrance …

P: There are times we run short of junior staff we don’t have them to go out to work with vaccines are not there so sometimes the logistics human resource, the logistics and other --- all those things sometimes hinders our activities

<Internals\\IDIs DHMT\\NRDI_02> - § 2 references coded [30.82% Coverage]

Reference 1 - 7.55% Coverage

I: hmm ok, thank you very much for that, eeh I would like to know how what are some of the suggestions that you would like to give apart from what you have just said in improving the reporting situation.

P: I think the suggestion, we have to also may be concentrate more on the education , more education on the parents because they are the first people who will have to recognize and then eeh report it.

I: Ok

P: So we - it looks like we haven’t done much in terms of actually eem eeh educating *the* the parents the education has mostly been to our staff

I: Ok

P: But the first person to recognize the AEFI is the parent. If the parent doesn’t report there is no way the staff will even know that this eah thing has come up, so apart from the trainings and whatever we give to our staff we should also concentrate much on the parents.

Reference 2 - 23.27% Coverage

I: Aah thank you very much Sir, we are almost at the last part of our --- our interview at this point I would just want you to throw some eeh more light at eeh what you think about the whole issue regarding AEFI. So for you what generally would you like to eeh tell us about it.

P: Well, u hu AEFI is is eem is something we need to actually pay much attention to because it it it makes us understand what the vaccination actually eeh does eem if we are able to understand it very and form vey scientific opinions about it then some of these wild myths that some people have about what immunization can do or cannot do will be able to eem eah samon them I mean some of these wild rumors because I quite remember when I was working in Yendi district as a disease control officer eeh that was at the time that we were eem polio was actually at its pick and we were --- the polio eradication programme was at its pick.

I: Ok

P: There was one child that we [caughs] we we who had polio I mean and then during the investigation we got to know that the child never eem had any vaccination and when they further probed the child at that time the child was about 8-9 years old the who had the polio he was already walking before he got the polio so when we investigated we got to know that that particular man’s children none of them have ever been immunized and apparently the reason was that one of the children was ever immunized I think in the past and the child got a reaction and they concluded that that man’s children don’t like white man’s medicine and that was it none of the children after that one ever got immunized but this was probably an issue of AEFI the child reacted to the vaccine be maybe it was an AEFI and because that time the understanding wasn’t there people didn’t understand it very well they took a superstitious position and then decided that this woman or this man’s children don’t take white man’s medicine and that was it. So I think that this AEFI , it will let us know about vaccines and therefore whatever happens can be explained scientifically, instead of… because at that time knowledge about AEFI wasn’t that much as it is now and therefore eeeh people could not even explain to the man what the issue was.

I: They only had to re-enforce his believe

P: yeah he believe, so eeh that would let us… the issue of AEFI would let us know more about it but eeh It should also be that whenever reports are made the feedback should be as quick as possible because when you send reports and you don’t get feedback we don’t get motivated to continue to do it.

<Internals\\IDIs DHMT\\NRDI_03> - § 3 references coded [28.23% Coverage]

Reference 1 - 10.67% Coverage

I: So for you how do we improve the reporting of this AEFI?

P: Normally any time that they get any chance as for AEFI the region are always on us because our reporting is low so now any reporting or anytime I get opportunity concerning about training or any general, I talk to them about it to them that AEFI doesn’t mean that you are not qualified we wanted to know whether the vaccine is having a problem, or the injection has expired and you are giving it. so normally when it happen that they should be able to report they shouldn’t think that they are the person who have caused it they are the people who have cause it but then it could be other factors so we wanted to ascertain whether there are factors that are causing that AEFIs so they shouldn’t think that because though they can make a mistake and it will result to that but is good that they should report it doesn’t mean they don’t know the work so wherever I get the plat form I normally tell them this.

Reference 2 - 14.76% Coverage

I: Yoo ---- I just want to know where there any general concerns that you will like me to know about eeh AEFI?

P: Eheh the AEFI the only problems I am having is that most of the staff as I have said, they thought or they are thinking when it happen and you report you don’t know your work that is what we have to do public education on it for to get to know that that’s not the case

I: Ok!

P: That is not the case that one if you are able to work on it they accept it that then most of it because there is immunization you will give and the child will cry even there is a column for it that you have to tick and then so that after the immunization the child body temperature was hot the mother should come you see the thing too is not instant the person will go to the house and go and notice it in the house by time the person who is doing the immunization might have gone so one thing too I have seen the mother should be educated that we give the immunization to your child and you go home and there is a problem come and report to us when we do that we will get them because is not an instant when you give immunization the woman just carry the child to the house and you be there may be three what is it after three hours two hours you start experiencing by that time the nurse might have left the community and is back to his station.

Reference 3 - 2.80% Coverage

I: For you the lack of eeh no under reporting is based on eeh the education?

P: That is the education aspect of it because some of the women don’t know that when immunize a child, the immunization is supposed to do something to you, this you understand.

<Internals\\IDIs DHMT\\NRDI_04> - § 2 references coded [20.16% Coverage]

Reference 1 - 8.65% Coverage

I: Thank you very much please let me know your suggestions for improving the process of reporting adverse events following immunization?

P: [door knocking] for this … issue I always emphasis day in day out we train new staff, we post new staff, we receive new staff. This new staffs ..... should always be updated …. And should be informed and those who are there already we should urge them to continue reporting. Even if it is one within a year why, why not. Even if it is zero zero let us know that it is zero zero. But the old ones are not practicing and you know nurses …. (laugh) we copy from each other. When I come to this facility A, what they are doing is very difficult for you to change me from doing that one probably it will be very difficult, I don’t want my in charge to say I have gone contrary to his directions or her directions, so I follow what he is doing. If he is the, if the new person is coming to bring the new form errrh the form to report when the in charge was not doing it or the old staff were not doing it then it will be another thing again. So sometimes lets try as much as possible to emphasis on the new ones who are coming. Train them of the importance of reporting including this event and those, the existing nurses should have all the tools with them and we also urge them to report as quickly as possible and when they report we shouldn’t blame them, because if I report one and you insult me to the level as if I didn’t go to school is better, the next time, I won’t report (inaudible) probably my colleagues are shielding them up you are not aware … I have reported and you are insulting me for doing this and doing that next time I will not report, this are some of the things. I think when we do that one it will help.

Reference 2 - 11.51% Coverage

I: Okay we are almost at the end of our interaction. However I will like you to tell me any other issue that you will like me to know with regards to reporting adverse events following immunizations in general.

P: Ahh I think with the AEFI, it is ….. it is very, it is very important in our …. EPI services. Because if a mother …. Bring a child…. After immunization the mother is not feeling comfortable at home, the child too is not also feeling comfortable it is very difficult for, for, for the, for the mother to come back again or those closer to her, she may be giving them wrong information that they shouldn’t come, they shouldn’t go, when they go this one will happen. So we are entreating all our staff to report ….. A.E yeh AEFI as at when it happens. Them bring the client if the need arises then when the client gets here we as the DHMT manages the case with the medical officers if it needs medical att, attention and if it is also something that we can manage at our local level we do it and then let them go. So …… it is very important and I think that ..... we at the DHMT level nurses who are coming in and those who are already there (inaudible) I mean AEFI. So that they will know the importance of it and then the forms, the reporting tools should be sent to all facilities including CHPS compounds, because they go into the communities day in and day out and they are always with the children. so as at when the issues are coming they can quickly refer arrrh I mean report and then intervene will be put in place to admonish them. But if we leave them and say ohh report, without following it up. They look as if they arrrr I mean is not important and that is most of the things that is also driving some of our mothers for coming for EPI services because, your mother today and she is not feeling comfortable at home she won’t come back again. Probably the mother was not even given education or counseling as to what will happen after taking the immunization, probably fewer and those things (inaudible) given immunization, arrrh I mean counseling, so I think that we have to make sure that our staff are always doing the counseling ….. giving them information enough information not just information to, for them to know that they an easily come back as at when there is problem after immunization. And that will help us.

<Internals\\IDIs DHMT\\NRDI_05> - § 2 references coded [21.50% Coverage]

Reference 1 - 8.64% Coverage

I : Okay, thank you sir. Please let me know your suggestions for improving the process of reporting adverse events following immunization?

P : Yeh I think ehhh, ......... One of them is that we just have to allay their fears that or let them understand that, .......... In whatever you do ........... Or out of ten you can get one ........ Adverse events following immunization which no fault of yours. Because if the child is running temperature after immunization is considered an adverse event following immunization, you have to report! because you don't know what probably is causing the what is it call the ehhhhh the con the situation. And then I think we need to continue to engage our staff ahhh in most of the meetings that probable we organize we still have to continue keep reminding them to report and to also let them understand the fact that if it is reported too and you report no body is going to take you on is just going to help the system work better. And I think more to the point ahhh we need to also ahh may be do on the job coaching with regards to filling of the forms, because when you bring people together there are tensions and the concentration is not there, they think that is that easy but when they get to their level they may find difficulty in filling it but I believe that with our monitoring visits whether we go as a group or you go as an individual ..... you can take the opportunity to just take them through ... or you can even let them take you through .... were they have challenges then you help them out ... So the job coaching also help.

I : Any other suggestions?

P : Hmmmmm well I think may be basically for now emmmm this is what I think will help.

Reference 2 - 12.86% Coverage

I : Ehmm this is the last part of our interaction, I will like you to tell me any other issue regards to reporting on adverse events following immunization that you want me to know in general?

P : Well what I will say is that ehhmm ...... Ehhh ...... it is part of .... the reporting system and it is also important that we report ehhhm ehh but as you and I know ehh reporting from the facilities to the next level is a challenge. You know health workers ehhh for whatever reason ( clap) do not want to report. In fact sometimes we have to go to the extent that even if it is emmm you know and some of them like I said because they don't want to report of for fear that they will take me on or for whatever may be ehhhhh the sys or the communities will not trust them thinking that enow is because of their enow action that is why the child is suffering but it isn’t the case some go to the extent that you take paracetamol give paracetamol to the child enow which of course is not the ideal because there could be some form of interference well we should we all know that the basic ehhmm ehmm immunology or ipimology that we learned when a foreign material goes into the body of course there will be swelling, there will be redness, there will be pain these are things that they are suppose to do but they don't do it So of course is mandatory we report but they don't do it, so may be that is what I can say for now but we will continue doing our best in making sure that we tell them that they do the reporting .... because it is important and in fact I think even for now with the latest update ..... is like we are suppose to even report a number ..... either we will be appraised or something like that appraise (inaudible) in fact some bo usually let me say the on going feedback we get is that ... we are not reporting, may be you are there and the regional level calls you or we get a call from National that you not reporting on AEFIs. But there are several of them occurring you know base on that we also tell the facilities even though we are already telling them because it has become part of the system the reporting system we suppose to report. It isn’t that they don't occur they do but we think sheee some of them are minor though we have minor and you know emmmm major ones or may the serious and or mild ones but they are all AEFIs we are suppose to report. So I think basically this is what I can say unless otherwise you want me to say more or may be I have not responded well and you still have to ask more (smile).

<Internals\\IDIs DHMT\\NRDI_06> - § 1 reference coded [7.94% Coverage]

Reference 1 - 7.94% Coverage

I : Okay, thank you sir. Please let me know your suggestions for improving the process of reporting adverse events following immunization?

P : Yeh I think ehhh, ......... One of them is that we just have to allay their fears that or let them understand that, .......... In whatever you do ........... Or out of ten you can get one ........ Adverse events following immunization which no fault of yours. Because if the child is running temperature after immunization is considered an adverse event following immunization, you have to report! because you don't know what probably is causing the what is it call the ehhhhh the con the situation. And then I think we need to continue to engage our staff ahhh in most of the meetings that probable we organize we still have to continue keep reminding them to report and to also let them understand the fact that if it is reported too and you report no body is going to take you on is just going to help the system work better. And I think more to the point ahhh we need to also ahh may be do on the job coaching with regards to filling of the forms, because when you bring people together there are tensions and the concentration is not there, they think that is that easy but when they get to their level they may find difficulty in filling it but I believe that with our monitoring visits whether we go as a group or you go as an individual ..... you can take the opportunity to just take them through ... or you can even let them take you through .... were they have challenges then you help them out ... So the job coaching also help.

<Internals\\IDIs DHMT\\NRDI_07> - § 2 references coded [17.47% Coverage]

Reference 1 - 5.89% Coverage

**I:** Please let us know your suggestion for improving of reporting AEFIs.

**P:** yea, one – we should make the form to be highly and readily available and the filling should be simpler. The form should be simple after all we are just I mean, we should, once we are talking about salient points and avoid the things the person even reporting will not understand. They used words, the words that the person even does not understand. Since it just a report let just make it simple, one page and they are ready to report. And then as I said [Clears throat] constant reminder and feedback and the person also should get feedback. If you report and you don’t get feedback just because they are waiting for the one that is serious before to normally follow up. And we are saying that in surveillance ones the person does some kind of surveillance performance of reporting the minuet information let the feedback come that yes, we has heard of the but if you don’t do that, and you are only looking for and because you are only looking for the unusual ones, so that is the one you follow up and it defeats the purpose of the reports. So feedback is also important so feedback aware.

Reference 2 - 11.59% Coverage

**I:** we are almost done. Please me other issues that you will like me to know with regards to reporting AEFIs in general.

**P:** AEFI in general, I mean is something which official should just continue to be in place because we have , we are aware that it is in place to help the manufacturer, it helps the program manager and then the helps the system and then it helps great cordiality between the care givers and the clients. And then it binds, because it shows a care and I mean a level of care. You have given something to somebody and you becoming even interested in the aftermath of that thing. So that alone, we even have being telling that when you build trust with the client, you will see, they will now stick to the system and for that matter the programme as it continues, and then they continuously be your client. So that is it.

And other hand, the documentation, the forms, I mean they should all the time be available, however you will see the constraints as we sit here they don’t even have paper to this thing, you may even have a softcopy of the form and you want to print it out but you can’t because there is no stationery. What do you do? Yea, so those things are there and that one is beyond the control. At a time we have had instances we have to go and take old programme form or tally sheet to turn the back and print something. Al these things and movement, in case they even report you also need to move to the next level to even be able to once in a while see the clients who reported but the means of transport I mean, we need fuel and this is non-existing. You saw me, that is my own car and this morning they just called, that vaccine fridge is not working, I have to use my own vehicle took it up to *Vittin* to go and take the thing. From here to that place is over 8km; you will go, I had to just go and check it at my own expense. You see these are some of the things full when some these things come we don’t have, the office has not given me fuel, we have to use your own car and don’t have fuel will you go, no. so those are some of the things. So logistical constraints, I mean other things, stationery as i said, fuel, transport and all those things for stop. As for the human resource they are there. It is not a problem. We have enough human resource. That is it.

<Internals\\IDIs DHMT\\NRDI_08> - § 1 reference coded [4.79% Coverage]

Reference 1 - 4.79% Coverage

**I:** Mommy, we are also done please tell me another issue that you would like me to know with regards to reporting AEFI in general

**P:** Other issues

**I:** Yes mom

**P:**  Hmm, issues like?

**I:** That affects the reporting of AEFIs, AEFIs.

**P:** That is why am saying I am going backwards the only problem is the reporting some don’t know that they are to report as i said it earlier, some don’t know so the trainings and in service. Training on the job training will do. On the job training

<Internals\\IDIs DHMT\\NRDI_09> - § 3 references coded [25.25% Coverage]

Reference 1 - 7.48% Coverage

**I:** Please Sir; let me know yours suggestions for reporting the process the process of reporting AEFI.

**P:** Alright thank you very much for this opportunity. [laughs] if there could be a good avenue to orient new staff who come into the system on almost all the schematics areas including AEFI, which CPI is there, AEFI with be tackled, but for some time now, I think we lack resources too to orient staff when they report to the district, and some of them too, those, those who think they will be victimize when they report on AEFI, I think they need encouragement. We have to let them know that, reporting AEFI is now something to push them to corner but to rather to help build the system so that our caregivers can also enjoy the service we are giving to them yeah.

Reference 2 - 12.45% Coverage

**I:** We are almost done Sir. Please tell me another issue that you would like me to know with regards to reporting AEFI in general

**P:** We, there was a form in the system that we were using. It wasn’t quiet long, a program came and they had to change the form and another immunization program and they have to change the form. So it got a time, those who weren’t actively part of the immunization programme didn’t know much about the change of form so when there is a case, you will see different facilities reporting AEFI using different forms. So we recommend that when there is a change of form communication should be effective, shouldn’t be based on a program. They should let us understand that this is the final AEFI form or currently this is the AEFI form we are all using. Because, for some time your get confuse, you don’t know whether it is because of the program why awe have changed the form or this is the generic form we are using in the system for now. Another thing I will say that, I think, we wish we get more resources to orient staffs on AEFI because to them there is a stigma. They think when they report they are going to be victimize or something, so I think we have to orient them more frequently so that they know it is part of the work do or are doing.

Reference 3 - 5.32% Coverage

**I:** I think you are giving good suggestions, could you continue with that one.

**P:** (Laughs) thank you very much. I take that as a complement. But like I said, More training will enlighten them to know that it is a core activity that we have to partake in because we have to also let them know that AEFI will help us to follow up on which vaccine is doing well and which vaccine is also causing this kind of events. So that the manufacture will all use that to improve on their production, yeah. So it all boils down to training, yeah. Thank you.

<Internals\\IDIs DHMT\\NRDI_10> - § 2 references coded [10.73% Coverage]

Reference 1 - 6.11% Coverage

**I:** Please let me know your suggestions for improving the process of reporting Adverse Effects Following Immunization.

**P:** The only improvement is to expand the training and awareness to everyone, yes and even including the clients. I am telling you, even health worker don’t know because we do our trainings and thinking that Adverse Effects is only this thing, vaccine, no but if we left those people to know that is the only thing to expand. We don’t expand the training we can’t improve it.

Reference 2 - 4.62% Coverage

**I:** Please tell me another issue that you would like me to know with regards to reporting AEFI in general

**P:**  In general, we have a protocol and a form we designed, I can’t change it. Probably, if they want me to modify it, I will modify it. I am not a policy maker. It is a policy; so generally, I have so little to do there. And I can’t design one for my district. No, No.

<Internals\\IDIs DHMT\\NRDI_11> - § 4 references coded [15.92% Coverage]

Reference 1 - 7.59% Coverage

I: Thank you mummy, please let me know your suggestion for improving the reporting of AEFIs?

P: The process in “err” improving the “err”, one of it I will say is for us to re-take it to our health staff not necessarily training but may be some form of on job for as to probe farther for our education to be intensity, our education on AEFIs to be intensified. If these are done at the child welfare sessions it will help in a long way to improve the under reporting.

I: Please add more suggestions mummy.

P: And then, other the other err suggestions I can add is the EPI unit within the District health directorate, there is “emmm”, what we can do is to let them go and do some kind of on job training or if is possible we do a general orientation on only AEFI possibly when we organize trainings and then we duel so much on EPI and then may be we chip in this particular section we should have rather a full day or a half a day program for it for the emphasis to be felt.

Reference 2 - 5.39% Coverage

I: Ok, Thank you very much mummy, we are almost done, please tell me any other issue you would like me to know with regards to reporting AEFIs in general?

P: “Hmmm”, “Yaah”, the err issues that I think err you should take home or one, will have to be in the area of capacity building of health staff I think this thing if is well done it will go a long way to improve err the reporting and the second issue has to do with feedback, if that can be improved, it will edge as to do more for instance is I send a case and the I get a feedback from especially from the higher level, I will be motivated to do more but usually when you send and then there is no feedback in a way is demoralizing.

Reference 3 - 1.21% Coverage

I: Ok.

P: Then err, aside the capacity building, what I think we should also do is to plan this thing how do I even call it, err visits, visits study tours.

Reference 4 - 1.73% Coverage

I: Ok.

P: If we have study tours for health staff to go and under study what others have done to improve upon the reporting, it could also help and then “emm”, I think basically these two things I can think of for now.

<Internals\\IDIs DHMT\\NRDI_12> - § 2 references coded [21.78% Coverage]

Reference 1 - 8.58% Coverage

I: Please let me know your suggestions for improving the report AEFIs?

P: “Hmmm” “eemm”, they just have to I mean train us, we need awareness creation. When we have you seen, we are trained and make sure that all people who deal with err immunization should know that if you inject a child this is the procedure you have to go through. I think the training, continues training and refresher is in you can I mean strengthen it.

I: Do you have more to add to that?

P: “Hmmm”, to to *to* the suggestion?

I: Yes.

P: “eemmm” we also need to be doing follow up. When we monitor, round when we go on monitoring and they are doing it we have to sit down see that they inject and hear what they say whether the advice them or they counsel them of that so that this thing and if you is not confident sure whether they enter it. So when we always monitor you know or visit them they know that oh if we don’t do this then may be they will come back and this think. So we need to also intensify our monitoring system.

I: Ok.

P: And also make sure that err what did they say the forms are available in all facilities. If is there we keep on ringing ringing the bell with the training and their knowledge, they will this thing and in then with the communities too we need to inform them. Let the community members know that if a child is given immunization and this and this whether a mild or not the fellow should report, if we carry out may be group discussions, durbars into the this thing. I think we can make a heard way so

Reference 2 - 13.20% Coverage

I: Please tell me other issues that you would like me to know in regards to reporting AEFIs in general?

P: Other *issues*?

I: yes.

P: Other issues like what?

I: That impedes the reporting of AEFIs?

P: Impedes the reporting?

I: Yes.

P: “ hmmm”, number one is err means of transport, someone will be at err where? Dungu, if she get AEFI mama this thing, if she calls how to bring it a problem. “ wey mee” certain times you even have means petrol to bring is a problem too… to, to, to bring it. I think basically and the filling too is always a problem like I said. Certain times they feel reluctant too to fill the forms.

I: Ok, but are there any other issues concerning vaccine safety in your, your, your Metro Metropolitan?

P: Concerning vaccine safety?

I: Vaccine safety.

P: “*hmm*” alright, the vaccines are welled stored even though we don’t have a this thing here, what do they say? You see we are squeezed here our this thing is burnt, so we don’t have our cool room, he is manning our this thing. We kept it at a sub-district, so he is always there but the fridge’s are working well.

I: Ok.

P: And you walk into a facility, just some a few the CHPS compounds, some of them don’t have vaccine “*ah*” to store their this thing so it was is always a challenge for them because when they have a clinic they will move, pick after that they have to send them back other than that, that’s the challenge they have other than for safety is always this thing, safe.

I: Ok, thank you very much mummy.

P: you are welcome.

I: Just express yourself with any other challenges you might want be facing?

P: Any other challenges we might be facing? 4second with regards to AEFI?

I: Immunization, the immune, the vaccinations.

P: The immunization, the vaccination, solo shots is always our problem. Certain times we have the vaccines, no solo shots to give so what do we do. That’s a big challenge which will be, we used to be erratic supply of this things. Certain times too, some vaccines certain times it got short, just about some few months ago we had, we run short of yellow fever and the this things other than that (inaudible) so that’s our most challenge is the solo shots shortage. And then the facilities too, means of transport to go and carry out their err their out-reach services so that’s it.

<Internals\\IDIs DHMT\\NRDI_13> - § 2 references coded [12.49% Coverage]

Reference 1 - 3.71% Coverage

I: Please let me know your suggestions for improving the process of reporting AEFI

P: I think the training should be a continues thing it shouldn’t be something that should be just be a one month or one days business anytime because staff go staff come so all the time they should be training on it for people to know the essence of report

Reference 2 - 8.78% Coverage

I: Please tell me other issues that you would like me to know with regard to reporting AEFI in general

P: I think there is there is enough room for reporting the adverse event because we have forms and they are always trained but what I said the training should always go on for people health workers to know that is very necessary for them to report and then the care they will give because it also depends on the care that they will give them will let them report because at times when they come and give them some care and you are charging them for next time they wont come so it think these are some of things, there should always be drugs available to (inaudible) these things even though some of their NID when we are doing they come out some of the drugs to (inaudible)these of those things we need

<Internals\\IDIs DHMT\\NRDI_14> - § 2 references coded [10.96% Coverage]

Reference 1 - 4.43% Coverage

I: Please let me know your suggestions for improving the processof reporting AEFI

P: my

I: suggestion

P: my suggestion is that aa they should trained the health workers to understand actually what AEFI is so that when they see it reporting it will help us to even improve they should not think that if they report it might be that you don’t know how to give antigens that its why

Reference 2 - 6.52% Coverage

I: Please tell me other issues that you would like me to know with regards to reporting AEFI in general.

P: [] in general that is why initially I said even though we are not reporting it but that doesn’t mean that we should stop there we should still continue training staff and reminding them so that anytime even if it not there EAFI EFI we can still attend to the client and let him understand that yes that it’s not it that is not thedrug or that is not the antigen but we should still continue to remind and continue on training we should not stop it there

<Internals\\IDIs DHMT\\NRDI_15> - § 2 references coded [14.59% Coverage]

Reference 1 - 4.31% Coverage

I: Please let me know your suggestions for improving the process of reporting AEFI

P:I think health personnel are supposed to, we the district will have to lay more emphasis on the need to report any AEFI whether live threatening or not

Reference 2 - 10.28% Coverage

I: So Please tell me other issues that you like you would like me to know with regards to reporting AEFI in general

P: other issues I think most of the health workers that are posted to this district some will just come and then they are taken to a fill- (cannot hear word well) facility and to go and manage it on their own [ ] crying scream and maybe they may not more knowledge on these AEFI but maybe if they had been posted to work under somebody that person might at taken them through let them know the importance of reporting AEFI there they will take it up

<Internals\\IDIs DHMT\\UEDI_01> - § 2 references coded [19.17% Coverage]

Reference 1 - 10.71% Coverage

I: so can you please let me know your suggestions for improving the process of reporting, AEFIs

P: maybe (clears throat) to me I think during our trainings, subsequent trainings, we should let the health workers know that it’s part of the, the, the work. As far as injections are concerned, there will definitely be abscess and all those things are bound to happen (yeah) so when it happens, they shouldn’t feel like it is their mistake or it’s their fault, they should feel free and report (ok) so that the, the, the, the, whatever will be done to such a client will be done in time before it gets too bad (yeah) that is, the most thing we are supposed to do.

I: any other suggestion?

P: Apart from that I don’t think there’s any other

I: so, educating them, then then

P: Yeah, the health workers to know, because I think, what the, the reason why they don’t report is that they feel like they should never inject somebody and for the person to get a condition after the injection

I: ok

P: They want everything to be perfect which is not done, there is no human institution that is (yeah) Ahaa, not having an error. So when an error occurs they should be able to accept it and report

I: ok

P: if they believe that its part of the work, whenever it happens they will report (ok) but where they get to feel like is, is a mistake it will not be reported

Reference 2 - 8.46% Coverage

I: ok can you please tell me other issues that you would like me to know with regards to reporting of adverse event following immunization in general.

P: oo, (laughs)

I: (laughs)

P: as for that, any other issue pertaining to the reporting

I: yes in general

P: I think maybe the issue that I see that is going way ward for now is the reporting forms

I: ok

P: because I know, that is what I know anyway but I don’t, I’ve, i have never seen the form myself (ok) I know that there’s an official form purposely for reporting that (ok), if it could be made readily available for all our facilities (ok) because there are some of our facilities i visit, you would even ask them and they they will tell you they have not even seen the form before (ok) it means they don’t even have copies (ok) if it could be made readily available for our community health nurses to be aware both the general side and it should just be readily available so that when, as and when it happens, you take the details of the person, the caregiver and forward it to the next level that will be good.

<Internals\\IDIs DHMT\\UEDI_02> - § 2 references coded [12.58% Coverage]

Reference 1 - 5.75% Coverage

I: Ok. Can you please let me know your suggestions for improving the processes like reporting an adverse event following immunization?

R: My suggestions

I: Yeah.

R: Yeah. As I said ehm we will need a regional focal person (Ok), then we’ll need district focal persons (Ok), we’ll need facility focal persons, and we need to get a schedule of reporting… because as for adverse reactions, we need to report them (yeah), and there’re are a lot of them out there that we’re not reporting (Ok). So we need to get timelines, maybe quarterly for people to report, and then eh probably eh ensure that people really report (Ok). Ehe I think that’s how we should do it.

I: Ok. Any other. You’re saying

R: And then and then and the training that I said, we need to do the training. We need to do the training quarterly so that people will be reminded always (Ok). Yeah.

Reference 2 - 6.83% Coverage

I: So can you please tell me like other issues that you would like me to know with regards to reporting adverse events following immunization in general? (6 seconds).

R: Yeah, what can I say? I would… the others issues I think I have said most of them – the fact that we need to… I think I was using some paper somewhere (Ok) and I think it’s not Ghana alone – for many many countries (Umm), they’re not being reported at all (Ok). And if you look at issues about fake medicines and other things around us. I think it’s something we have to take more seriously (yeah). Yeah. So for me, I think… we’ll need to engage collaborators especially the Navrongo Research can be a very good (Ok) partner (Ok) to deal with this issue. So I think we should form a (noise) partnership and be more serious (Ok) with adverse eh (events) events reporting (Ok). Yeah.

I: Ok. Any other thing you want to add to all that we have spoken about?

R: *Ah* no I think it’s Ok, I’ve spoken enough (laughter). I’ve spoken enough, I think that’s it.

<Internals\\IDIs DHMT\\UEDI_03> - § 2 references coded [10.28% Coverage]

Reference 1 - 8.79% Coverage

I: Ok. So can you please let me know your suggestions for improving the process of reporting adverse events following immunization?

P: Hmm! Toh, that that, my suggestion should have been Hmm. It’s our people under the ground (Umm). That is where the problem is (Umm). You see, if the people have the training as we said because I will say that it’s the training, mostly I would have say that they should have had enough training (Umm). Just like what we are saying. If each district are having at least, maybe sometimes it’s just some of these exercises that we come to do training (Umm). Maybe we just go to a region, we’ll also come and just say it. But like maybe the suggestion should be like people, the drug the drug, the Food and Drugs board should also come. Like maybe some of these training that maybe we sometimes have a chance. But the region sometimes come down. Why not, maybe one person should join the region people and also trickle down to the level. So the people should not be seeing the same faces everyday (yeah). You get it? (Umm). Every day the same person, the same and then be talking of the same thing (Umm). Somebody different come and they see a different person also talking of the same thing (Umm), they will know that actually it’s serious (Umm Ok). It’s just like what I’m saying, maybe we’ll try to explain that it’s not the matter of the person that don’t know how to give the drugs, or to do the injection and all those things, so that we’ll all understand, and report appropriately.

I: (coughs). Any other suggestions?

P: Umm. The suggestion too… like another thing is that, I see the form is loaded (Ok). Ber you ever seen the form?

I: No, not yet.

P: Ok when you go, that is what I’ll tell you. Try to see how the form is loaded (Ok). And you know most people immediately they see a form loaded like that, that one alone scares you to report (yeah). After all the form is loaded. Immediately the person sit down to struggle ahhh to fill the form nu, if I leave it what will not happen? Who will come and ask me? So that is another another problem (Umm). The form is loaded too much.

I: Ok. Any other that you think of? Any other suggestion?

P: Now dier I don’t think I (laughs) I don’t have any suggestion to add.

Reference 2 - 1.48% Coverage

I: (It’s true). Can you please tell me like other issues that you would like me to know with regards to reporting AEFIs in general? (5 seconds).

P: Any other issue. I’ve already declared all the issues there, so is it any there me I don’t have any general issue (Umm). Issue is an issue. Issue is an issue (it’s true).

I: (laughs).

P: So the issue have been given out (Ok), Umm.

<Internals\\IDIs DHMT\\UEDI_04> - § 2 references coded [23.72% Coverage]

Reference 1 - 5.06% Coverage

I: okay sir and what would be your suggestions to improve the reporting of adverse events?

P: we were actually, we organise our orientation, it was suppose to be effective from tomorrow but the regional team is not available so we are shitting it forward as and when they will be available to come because it’s an annual affair, we do it for all the new entrance and people working in other districts who have been transferred here or other regions so that they get the benefit of what we want to do so in such orientation, then the information is given, the forms are introduced to them and that is how they will get report.

Reference 2 - 18.67% Coverage

I: okay, and so generally what would be some of the issues that you think that could be taken into consideration to be able to improve the attitude towards reporting of adverse events on side of caregivers and then aaahh healthcare workers.

P: I think, it’s constantly reminding the healthcare workers that any product we give for consumption could potentially cause an adverse event and as soon as we hear there has been an adverse event, we need to report. So it should be a continuous thing. It’s not when people go for the meetings and come you see the adverse events being reported and with time they die down obviously we are very fluid as I said, so go to school so if one person went for the training and starts reporting alot of adverse event, when the person moves away, then it dies down and that is one of the challenges. When you realise like the reporting, the food and drugs authority, Upper East does well when it comes to reporting of adverse events and mostly it’s revolving around on few individuals so if those individuals are away then it turns to slow down the reportage in those particular facilities.

I: so would there be any other suggestion that you would want to give on improving the reportage?

P: no I just think that once people are constantly reminded, when we go on monitoring we should find out if they have the forms and find out how many they have reported because it’s bound to occur and probably that constant reminder would let us report more, I think that is the main thing.

I: so out of this conversation will there be any or issue concerning adverse events that you would like us to know about since you are in this area of work and probably you have encountered different scenarios.

P: yeah, there’ve been several cases reported, we had somebody reporting Stephen Johnsons’ syndrome, that was not in this district anyway, an adult who took Efpak two tablets and four tablets of paracetamol at the same time. Yeah that was a 54year old male and it occurred, it was reported and it was acknowledged, yeah and the suspected medications, paracetamol was one and athesonate amodiaquin had been taken in so virtually everybody was thinking of athesonate and amodiaquin but the feedback from the food and drugs authority indicated it was the paracetamol that caused it.

<Internals\\IDIs DHMT\\UEDI_05> - § 2 references coded [16.65% Coverage]

Reference 1 - 8.35% Coverage

I: so please let us know your suggestions on how to improve the reporting of adverse events following immunizations?

P: okay, thank you, with this one my own opinion is that I will suggest that funds will be made available for staff especially the refreshers to be trained on the reporting then they should also be, all is about the funds, then there should also be commitment commitment on the side of the staff and commitment on the programme officers so in such that when, eerrh there should also be immediate feedback to staff that happen to report such con... eerrh cases because once you the person report and you are able to give feedback, the person will be okay, yes the people have actually appreciated what I have done and so the next time they record another one they quickly report it again. All has to do with the funds to train and give refresher training to the staff and I possible they can (cough) also integrate it fully in the educational curriculum at the school level so that especially the community health nurses so that when they come out then they will have adequate knowledge about problem or the reporting system.

Reference 2 - 8.31% Coverage

I: okay so please tell me any other issues that you want us to know regarding the reporting of adverse events following immunization as a public health nurse?

P: okay like I said earlier on, the reporting system need to be strengthen, eerrh, you know in Ghana here people believe in responsibilities given to them, so I will suggest that, going forward the system should make a provision for staff refreshed regularly, at least if not every quarter, at least every year it should be done once, then people who are also given the training when they get the training and also come back should also do the same to their colleagues at the facility level so that in their absence there shouldn’t be any disconnection but there should be continuity of the reporting this thing to go on, (cough) then if there is any update on the reporting format, it should quickly be made known to the staff especially so that when they get it they will know much about it and I will also add that DHA will have to integrate it into their monitoring as we used to do so that as and when a team is moving out then they will be able to check on those things.

<Internals\\IDIs DHMT\\UEDI_06> - § 3 references coded [15.52% Coverage]

Reference 1 - 9.27% Coverage

I: okay so would you have any suggestions for improving the processes of AEFI reporting?

P: yeah, then the suggestions should be if there can be a system, there can be a system, am also careful of this system thing because sometimes anything that deals with technology it like you have to go for data and those stuff and sometimes when it also fails you, you are down and I don’t know how it can be done that it’s a soft copy, you fill it sending to them becomes easy but the hard copy is not helping to some extent, to some extend its not helping, so if a form can be soft copy, when I do it and even I have to sent through mail or WhatsApp or whatever that can become somehow easier then the refund, refund of what they spend on the patient, ahaaaaa if the person is non insured and mmhm for because with the transportation thing if its hard copy, if its soft copy it means there is no need even ahaaaa. And how sometimes too region, is it region or how other people react towards you, when you submit something they say “why did you do this this way”, you know how somebody will speak to you, ahaaa it’s also a way that it can boost your this thing to keep submitting or stop; “you dei any thing small bia no, you report as AEFI, is this a case, this one is just fever, this one you ...”, you see and that can demoralise the person that oohhh, there is no need I keep reporting this and when the morale goes down what that means the reporting will not be done and when you come for instance they have posted you to mine facility for the first time and I come and see that you are not reporting on it, that attitude of you not reporting on it transcends to me then I will oohhh even the one that I was learning from was not interested in reporting then I will also transcend that same thing to the one who will come before me or will come after me, ahaaaa so that has been the this but training also will do, training will do.

Reference 2 - 2.34% Coverage

I: okay so in the case where the refund is not given to the facility or the health care worker at the grassroots or at the facility level, who takes liability for the cost then?

P: the facilities

I: okay okay

P: that’s why at times they will say this facility is has run out of drugs, he’s not able to account for the drugs and meanwhile when they were giving those treatment and recording and even submitted to you, you didn’t pay them and that’s running, giving them debt and stuffs

Reference 3 - 3.91% Coverage

I: so can you please tell me other issues that you want us to know regarding AEFI and its reporting?

P: what I will say is the the the message to some extent is down with the, am talking about the caregivers reporting of AEFI is like, that one they have it to some extent, I will say that, they tell them but when they report, it is our responsibility to take the thing up, take it up, take the sample, eerrh, fill the form submit it to them then, you will get the feedback. So the problem really is not coming from the caregivers but much, much of it is not coming from the caregivers but much of it is coming from we the health staff, how we rubbish, this is not AEFI, go!!!!(With lot of raging emotions). How we don’t refund the amount spent on them, eerrh the knowledge as well and then our style of reporting.

<Internals\\IDIs DHMT\\UEDI_07> - § 2 references coded [12.16% Coverage]

Reference 1 - 8.90% Coverage

I: alright thank you sir so can you please let us know some of your suggestions on how to improve the process of reporting adverse events should they occur?

P: on the part of the health staff I think we should continue training, let them understand that there are so many mmhm reasons why a child may have an adverse event following immunization, it could be due to the drug itself , so not necessarily their fault alright and so if they know that they will be more than will to report them when there is an adverse event and also sensitizing even those on the other side, the clinicians that for any reaction resulting from vaccination or after vaccination, it needs to be reported its a public health concern that they should report. They know the need to report they may report.

Reference 2 - 3.26% Coverage

I: okay, so sir finally, can you please tell us if there are any other issues regarding reporting of adverse events.

P: apart from the fact that the reports are not forth coming even though we believe the events are happening mmhm, (loud voice persist) I can’t think of any other issue

<Internals\\IDIs DHMT\\UEDI_08> - § 2 references coded [9.75% Coverage]

Reference 1 - 3.67% Coverage

I: okay so please let me know your suggestions for improve the reporting process of AEFI’s?

P: it should be flexible to our staffs whenever they report, we should take it as such, we don’t need to criticise on what they report, sometimes tag them they don’t know anything, its not the best. We should embrace them the way they are, if they make mistake, correct them through that rather than blame them

I: will that be all?

P: yes

Reference 2 - 6.08% Coverage

I: okay thank you very much, so please tell me if you want me to know any other issue relating to adverse events and issues relating to reporting adverse events.

P: (long paused as she was thinking) you know, one thing is that the community people or community members, some of them reporting something to the facility they find it difficult to do so, so some when they see such things they relax at home, so I think its good we intensify our home visit so that we will be able to identify such reactions at the community level and treat them as such.

I: will that be the only thing you will want me to know regarding adverse events and reporting them?

P: mmhm, yes thats all for now.

I: you are sure?

P: yes I am

<Internals\\IDIs DHMT\\UEDI_09> - § 2 references coded [14.43% Coverage]

Reference 1 - 7.20% Coverage

M: Let me know your suggestions for improving reporting on AEFI’s

R: It can just be what is already happening but then involving all parties like if there is the need to train downward to everyone and also if we are able to capture weekly instead of waiting. Like I said on phone we can only say on phone this is it but it to accompany with the form takes a longer time before you know the person would have forgotten. So if we are able to improve on those things weekly and training those who are actively involved all the time and also making a fellow-up weekly as a way of reminding people. I think is will be better than the way it is done now.

Reference 2 - 7.23% Coverage

M: Please tell me any other issues that you would like me to know with regards to reporting AEFIs in general.

R: AEFI’S I think the general issue will just be like I said we over look them and some time we say no need because of how our reporting system is. At the end of the period you need to collate a lot of reports and sometimes we over look them. So if we can have something like reminders always because all these things we Co-ordinators that are Co-coordinating them. Once a while the Co-ordinator can remind you that I have not heard from you this week is everything correct just to remind you and all that. I am sure with that it will help.

<Internals\\IDIs DHMT\\UEDI_10> - § 2 references coded [20.28% Coverage]

Reference 1 - 7.39% Coverage

M: Can you please let me know your suggestions for instance, the process of reporting AEFI’S?

R: I think what we have to do is that we have to do quarterly AEFI’s monitoring. We also need to let our volunteers. That is the health care givers who are in the community level need to tell the health volunteers. In fact, ideally they are supposed to work hand and hand with the CHO’s in health care delivery and those things. So they should be made known that anything that happened after the immunization, they should report to them because they are very close to the volunteers. So the volunteers should be considered and then our level too we can do quarterly monitoring even though I know these days there are resources constraints and those things. Also if we give reports three months’ time something should be done about it because we cannot give you information and nothing is happening.

Reference 2 - 12.89% Coverage

M: Tell me any other issues that you would like me to know with regards to reporting AEFI’s in general?

R: I think AEFI’s in general are; we have serious and unserious ones unless of course it is a serious one we don’t report on it especially mass drug administering on school children the unserious adverse effects are always too many because it takes a short duration and if it is unserious nobody talks about it. Unless you take it and you are being admitted or any other serious thing even though we are supposed to report whether it is serious or unserious one but mostly we fell reluctant to report the unserious ones.

M: What do you think can be done so that an AEFI in general reporting cases, identifying cases and how to resolve them can help improve the system?

R: Unlike any other data that is important I think AEFI data is also important like I talked about the quarterly monitoring when we do the quarterly monitoring we can call some meetings with the sub-districts. So far what we are doing in the system on AEFI’s is that like the drugs we give and those things we can do presentations and show the trends of AEFI’s in the district if you immunized your children, they say come and give your coverage and those things do we care about AEFI’s? We don’t talk about their trends. So that one should also be considered as an important indicator, the number you have reported, so that it will motivate people to report and also people should not attach Negative mind set about AEFI’s. We should rather encourage people to report on AEFI’s.

<Internals\\IDIs DHMT\\UEDI_11> - § 2 references coded [16.15% Coverage]

Reference 1 - 8.51% Coverage

M: Can you please let’s know your suggestions for improving the process of reporting adverse events following immunization?

R: My suggestion is continue training of all the health providers on adverse events following immunization from the district level down to the CHPS compound level. Also, refreshers training to some of the volunteers who are supporting us because some of these things are picked up by them when they come across them; they call the health staff to follow up. So I think continue refresher training of these events is very key. Number two provision of the necessary tools for these data to be collected and also we the health authority should also insist on every health care provider to try as much as to fill every adverse event and not to consider some as minor, others as severe because we don’t sit to wait for clients to come with severe adverse event like Stephen Johnson syndrome then we have failed. It is a dangerous adverse event that would occur to a child. We should consider all adverse events very important whether minor or severe.

Reference 2 - 7.63% Coverage

M: Now I would want you to tell us any other issues you will like us to know with regards to reporting adverse events following immunization in general?

R: Yes I think it should be synchronized to have one template. I think adverse events following immunization and drug reactions all go to food and drug board. So they could come out with one form as and when we come across any then we use that to report but it looks like sometimes there are variations in the forms and when it happens that, you are looking for one you don’t get. So they should synchronize it to one form either adverse event following immunization stroke drug reaction. So that it will be one form. Also they should be available for us to use and if it could come in a booklet form, carbonated booklet form so as you write on the original form you tear and the duplicate is there for reference so that in case you even lose one copy the other one will be available for evidence based.

<Internals\\IDIs DHMT\\UEDI_12> - § 3 references coded [12.67% Coverage]

Reference 1 - 4.59% Coverage

I: okay, thank you, please let us know your suggestions for improving the processes of reporting AEFI? (voices still in the background)

P: my suggestion will be that, the few that is able to compile and submit, we should get a feedback, a feedback comes with a solution to it and if that is right to receive and its gotten to the client it would encourage others to give. Another one I will talk of will be the training, building capacity on the reporting of AEFI, people understand the concept, then right from the giver to the recipient all the information you need you will get, clients will learn to give these reports to the caregivers, caregivers will also learn to give submit reports their superiors and it will be smooth, yeah

Reference 2 - 5.04% Coverage

I: alright, so what will be your suggestions or any issues that you would want us to know regarding AEFI and its reporting?

P: yeah what I will talk of is will be the link right from the district to sub-district to community so if that link is there you see that the information will flow from the community like from the programme time we were talking of and the reporting will go on well because it’s the client who is having the effect, the health worker who is at the sub-district level or the community level sometime visit, if they see anything like that they can record them and report to the sub-district from the sub-district to the office and the office will also forward so the one dealing directly with the client who need the knowledge most is not having it I think that is why the gap is there.

Reference 3 - 3.04% Coverage

I: so what would be the mmhm, sorry would you have anything other issues concerning the reporting of adverse events that you want us to know apart from what you said earlier?

P: yes with the other issue is the you see that some of the clients comes with a complain, the healthcare givers who does not know much about the effect of such a this thing may attribute it to a different sickness else, and that lead down to the reporting rate, aaaaaha so the bottom line is capacity building.

<Internals\\IDIs DHMT\\VRDI_01> - § 2 references coded [11.20% Coverage]

Reference 1 - 6.16% Coverage

I: That’s great, P: hmm. I: eem please let me know your suggestion for improving the process of reporting AEFI.

P: eer I think as I indicated earlier maybe during the immunization sections, ooh you know once in while at durbars and whatever anywhere you can get a group of people disseminate this information, talk to them bout, even if not immunization so that … parents, relatives will be aware of the situation, so by doing that you know when the problem comes is society you know is eer family

I: yes

P: immediate

I: okay

P: so now if this information go down they are aware and they know what steps to take, I think when we do this, and during the eer immunization sections too at the CWC at the grassroots they should talk about it I: and what other…. P: because surely a child will take immunization that day even if not all I: aah P: so I think, I think when we do that it will remind them, prompt them what steps to take when it happens

Reference 2 - 5.04% Coverage

I: That is fine, and eeeeh please let me know other issues that you would like me to know with regards to reporting AEFI in general.

P: the question again

I: The question is, please let, or tell me other issues that you would like me to know okay, eeem with regards to reporting AEFI in general

(4seconds)

P: ooh I think I mention a lot so I don’t know which area you want to

I: I want to hear more P: mm because yeah, ooh I think what I have is what I just mentioned so I don’t know, you ask of how the reporting should be… you think should …we, we think when we do it it will improve, I mentioned it that we need to give them the education the community need to be aw, aw.. care givers need to aware and when it happens they will report. That is the only thing I have

<Internals\\IDIs DHMT\\VRDI_02> - § 2 references coded [19.44% Coverage]

Reference 1 - 6.26% Coverage

P: Simple… yhh! Manageable ones yeah!

I: Okay. Errm Could you help us with a few suggestions for improving the process of reporting… or you think the phone system and the documentation is fine?

P: Mmhn… I don’t have [laughs] any I don’t have anything to say around that yet…

I: Okay, okay

P: Those are the two ways, documentation and then the phone calls.

Reference 2 - 13.18% Coverage

I: Please are there any other issues that I’ve not talked about or asked you about?

[Pauses to think and laughs]

I: Okay

P: But my only feeling that since errr we are becoming used to some of the minor… ahaa and we handle it is okay maybe that’s why maybe the reporting might not ahaa be too threatening since the minor minor it’s managed…

I: …yh that’s why the reporting is not…

P: So I was also thinking about, see when when I saw the letter I was thinking like that ah, why, I don’t know really why is not that people are not reporting, but I took the trouble to interview some of the old ones and they were telling me oh! this time they are used to managing the minor minor ones so they don’t have the problem of errr making too much noise about it.

<Internals\\IDIs DHMT\\VRDI_04> - § 2 references coded [23.10% Coverage]

Reference 1 - 20.01% Coverage

I: Okay, errhm can you please help us with some suggestions to improve the process of reporting?

P: Yes, the the like… the process of reporting I think like I stated, the community level [clears throat] is is quite okay. We don’t have much of a problem because like I said, most of the parents we’ve interacted with, they they are concerned with the welfare to of their children, so if something is happening to a child most especially having received the immunization, you want to follow up to find out what is happening to my my… let me give a classic example… There was this child that was haemophilic. Unfortunately, the nurse didn’t have an idea to that, so after they gave the PENTA and the mother went home, the child, the bleeding did not stop so I think that either that day or the following day they went to the nurse and complained that this is what happened. Then, actually they dididnt inform me, they they they have to see a doctor because I think it was within a hospital premises and the way the thing was, it was actually serious so they have to see a doctor and upon interrogation they got to realize that it was, that was when they now call to to to inform me. So one way that I did was I try to build their, their their… what do you call it their their… I encourage them not to be disappointed or to feel bad when they encounter those things because like I stated, I actually indicated the causes of the adverse events following immunization and made them understand that you you encountering it does not necessarily mean that you the staff, because if you are vaccinating out of a number of children maybe you pick a vial, you vaccinate a number of children, you will vaccinating over the period, yes, if genuinely it is you the the the staff… this thing errh ehat do you call it? Challenge! Or your staff problem, we should be able to deal with it so that we don’t encounter most of them; otherwise you drive away the mothers who will not come. Secondly, if it is as a result of, the I mean the manufacturing defect if you don’t pick it, I mean I also made them understand that the vaccine we are trying to, we are supposed to mon… we are monitoring it in case there is any problem with that batch, that’s why we ask them to, so we can quickly withdraw them from the system. So, I think when when I made mention of that th… them, I… I actually realize that some, the few ones that we… they they they personally called. And I also told them that it might not be their fault sometimes the the client or the baby has different reaction to it. So that if if maybe one particular staff is consistently recording adverse events following immunization if she doesn’t report, you know they are always in a team, the other one can hint me behind the scene so that we will go in, speak with the person and then offer I mean some training so that it will also boost that confidence in them.

Reference 2 - 3.09% Coverage

I: Okay, please is there any other issue you would like me to know with regards to reporting of adverse events, anything that I didn’t ask you in the interview process? … (pauses)…

P: Yh… I think… (pauses)… I think if, I even touched on it, like I said, I thik the other ones since we manage them locally, I mean errh national or regional necessarily doesn’t unless it’s a hospitalized case then maybe they will follow up but I think so far… errh…

<Internals\\IDIs DHMT\\VRDI_05> - § 2 references coded [19.88% Coverage]

Reference 1 - 11.54% Coverage

I: ooh okay that’s fine, and please let me know your suggestion for improving the process of reporting AEFI.

(3seconds)

P: for this one the, the, the health workers are aware may be we give more education to the caregivers that after eee immunization, immunization whatever happens don’t wait for that twenty eee, eee forty eight hours or seventy-two hours just come an do report that yesterday after going home we have, the child have fever and…. In fact am coming back or I’ve seen that the place is bit swelling or harden. We need to educate the caregivers that whatever happen to them they should come back that is the only thing eem and, and also to educate the caregivers, the health workers that if something happen after immunization that doesn’t mean you didn’t do your work , it could be that the child is reacting to the, the vaccines that you are given that doesn’t you have not done your work well so anything that happen after immunization they should report is not their fault, the, the eeeh you have everything you know where to give it but if something happen just report you might not know that you will be saving somebody so is just education that they should report, you cannot force them or do anything but just to I: educate P: educate them, educate the caregivers that just come whatever happens come.

Reference 2 - 8.34% Coverage

I: Thank you, please tell me other issues that you will like me to know with regards to reporting AEFI in general

(4seconds)

P: Well (5seconds) for reporting well what eeh they will not, what will I even say is just that eeeh we, we have more or region have more interaction with, with us or eem maybe eem, we don’t need eee, eee, eee even a workshop for this, the only thing is just inform the populace and then the health workers about AEFI, but I don’t …. In general eee, I think all that we are doing already that should be it (laughed) I: all that you are doing P: no, yes like we educating the caregivers, educating the health workers that you need to report, we need to report and if the eee, eee,ee there is any new vaccines we should have enough training on it to know the side effect so that they can also eem inform the mothers that this is what is going to happen if you take this vaccine, well that is all that I can say (took paper) (laughing)

<Internals\\IDIs DHMT\\VRDI_06> - § 1 reference coded [14.00% Coverage]

Reference 1 - 14.00% Coverage

I: do you have any other thing that you want to talk about that I didn’t ask you, concerning adverse events following immunization?

P: oh! errh I think it’s…it’s it’s ok, because they are so many things happening at the same time…

I: mhmm

P: urhhh most of the time it gets mixed up…errh…

I: …crowd…?

P: the…the crowd of errh things that we’re doing. So basically I think errh… maybe active search after some of those national immunizations or the programmed immunizations may help to distinguish it. And then a lot more literature and erhh erhh maybe, maybe should I say noise about it…

I: Mhmm!

P: errh… because there is so much noise about HIV, malaria, …this tha...tha...tha...that…

I: yhhh…

P: ...you get drowned in…

I: Ok!

P: hehe (laughs)… the noise… (laughs)…

I: uhh….

P: sometimes if it’s an issue that we think should come off, then we should flag it a little more than we’re doing

<Internals\\IDIs DHMT\\VRDI_07> - § 4 references coded [29.22% Coverage]

Reference 1 - 5.42% Coverage

I: Ok. Ehmm, please what do you think accounts or what are the factors that account for the low reporting of adverse event following immunisation?

P: May be, maybe I can also say, normally when you forward, most often you don’t hear the… they don’t give the. . . . the final feedback you don’t get. Then within the Ghana… One of the things I could say is ehrr, there were …there is always the promise go they will treat them free of charge etc, then they would pay back and all these but most often those payback don’t happen.

I: Ok.

P: So there are times that some facilities, when you send, they are reluctant to ehrr, do the treatment

I: Ok.

P: because of some of these processes. Normally they will promise, forward the bill, we’ll pay etc. As usual but you forward and that is the end.

Reference 2 - 13.80% Coverage

I: ok. Alright, please do you have any suggestions you think we, we… when adopted can help improve upon the reporting of AEEI?

P: AEFI from the district?

I: Yes

P: It all needs err regular training of the staff. Like I said initially, most staff are now on the field just about three years.

I: Ok.

P: And they have no …some have not experience such effects before,

I: Ok.

P: So we need lot of orientation and then training the newly posted staff about the reporting systems.

I: Ok.

P: We often do it but like human institution you may get one or two people who might have forgotten or maybe lazy,

I: ok.

P: Would not want to take up those task etc. Or maybe where I’m operating is a small place and nobody will border me so, you could just allow the reporting those things to go.

I: Ok.

P: Yea but so we need regular errh… At least if it’s yearly or once in every three as you come to brief them ehmm. . . This reporting, but I know is something that the disease control unit does.

I:ok, ok. Erhmm, you talked, you just talked about training, erhm, what kind of training do you think the staff would need with respect to this whole issue? How should the training be?

P: Yes, you see normally like Avemectin distribution, we will do training, you train volunteer etc, so at each of these trainings, you tell them about the… what to lookout for.

I: Ok.

P: The adverse events that could happen or side effects of those drugs. You give them out, so when they spot any such thing, they bring them to you. Not too long ago, about two months or a month back, we did these gifts, given err this-thing to errh… Yes, there were series, one or two reported cases of adverse effect that we have to send to the facility. And that was the place where the boy, my nutrition officer came to tell me that… Well you said such things are free but then, you know health insurance, you buy your drugs and do everything, so some of the facilities were not willing to just treat and let go of the money and things like that.

Reference 3 - 3.27% Coverage

I: ok. Now again about the training, should, should such training be facility base or at the district level or how should it be?

P: Errh, for a training like that, it will be time consuming moving from facility to facility but if you bring together at the district and then get training…

I: But given the fact that, errh we want to have an all –inclusive training, how is that possible?

P: Yea, you organise one for the health staff and then another for the volunteers. I: Ok.

Reference 4 - 6.74% Coverage

P: Because they are the people who will pick the cases from the communities more. They interact more often with the community than the nurses, so errh training from community to community might be time consuming and errh financially not sound to do..

I: Ok, alright. Please do you have any other thing, you think can be of help to this whole process?

P: Wuuw! What I can say is ehmm, it’s like we are… is not a main stream health activity so we often forget about it. Theee, the need to actually be reporting those things, so sometime we just let it go. So we have to main sweep in, in health service delivery.

I: Ok.

P: Is like a routine activity which we’ll pick up.

I: Ok

P: Then people will be more confident in reporting those AEFI.

I: ok, ok. Any other thing?

P: Mhmm, just to improve the health.., the managers of system too should also come up and not expect… I mean they follow-up their demands on the districts with actions that will help actualize what they want to do.

<Internals\\IDIs DHMT\\VRDI_08> - § 3 references coded [25.75% Coverage]

Reference 1 - 5.64% Coverage

I: Ehmm, do you have any suggestions that you think we can adopt, that can help improve upon the reporting of adverse events?

P: Yah, you know, ehmm we just need to sensitize our, our, our workers, our workers on… the lower level, workers that even the slightest, a minor complains also must be, must be reported on. Not only the very severe one that they should report on, because even if is a rise in temperature or whether is just erhrr, vomiting or whatever. Even the slightest erhrr event that they could even manage, they should report about it to the district level.

I: Ok.

P: Yes

Reference 2 - 15.64% Coverage

I: By way of training, do you think there is something…

P: errh, by way of training, what it also means is that, until we continue to periodically update their knowledge on it, they will forget and think that oh is something of the past and they will never report on it. But if we continue on a regular basis, errh remind them. Because you know arrh a lot… the turnover is very high now. You train somebody today after three years, he going to school, next time he becomes a midwife. About immunization he has forgotten about it and new people have taken over, so if you don’t re-train those people, it means they will not be… they will be ignorant about these adverse events. So even whether. . . even if the midwife is there but know anything about it but is not directly involve in the immunization ,she may not have the opportunity report on any of, of these forms. And the new ones that have come, they don’t have any idea. They have not be sensitized on it, they don’t have any information on it. Meaning they will never report and now even people two and half years… even the three years will not even reach before they will be forcing that they will go to school. So we need periodic update on the information on this.

I: So what kind of training do you subscribe to? Is it facility level training or…?

P: well, I think it could be on the job training.

I: Ok.

P: On the job training so that… because is not… I don’t think is anything so difficult that the district level staff cannot do, but if we’ve just been empowered and reminded that it’s necessary to do this on a periodic basis, so that know body will forget about it.

Reference 3 - 4.47% Coverage

I: ok. Director, please do you have any other thing, you think will help improve upon the reporting system?

P: ammh! As I said, errh, from the national level, regional level to district level, they, they, they, they… we should be for or be reminded that we need to update the knowledge of our lower level carders on report, reporting of these adverse effects. Without that one, two years, they will forget about it and it goes to rest. Nobody will think about it. Yes.

<Internals\\IDIs DHMT\\VRDI_09> - § 1 reference coded [35.96% Coverage]

Reference 1 - 35.96% Coverage

I: ok

P: I don’t think so because the same staff who is doing the immunization, is the same staff who is reporting. So if he or she has the time to report on all vaccinations given, that too fills on the immunization form indicating AEFI, whether is serious or not serious,

I: ok

P: that should not be an issue.

I: ok,

P: nhm

I: ok. Ehmm, what about also looking at it from the point of ignorance on the part of the healthcare worker?’

P: ok, ignorance, well, I will… that I’m not very certain about because…

I: Yes, I say so because, you know the turn over that we have in the system,

P: nhm

I: where we have a lot of people going back to school,

P: yea

I: and then we have news crop of people in the system who may not be introduced to some of these things.

P: ok

I: couldn’t that also be a factor?

P: ok, ok, I think there is some truth in it.

I: ok.

P: nhm, that is also possible. Is also possible (*someone talking)* and so for, for me personally as the EPI coordinator, with our training needs for next year, which we were made to compile, I factored some of those things in

I: ok, ok,

P: so that if we have that opportunity by bring all of them together, we would go through immunization in general and would touch on all those things.

I: ok

P: but that notwithstanding, even with the little… the one that was reported, ehrr, last month as I was telling you, as I got there, I had to sensitize and educate the staff on the condition. We went through the filling of the forms,

I: ok

P: ehm, I think three, three CHNs who were present as at the time I went. We went through it again and so… that’s also part of it.

I: ok. Amhm, please how available are the forms at the facilities?

P: Yea, the forms are available. Only that sometimes because they don’t make use of it, they don’t become conversant with it and some people may not even know where they placed them.

I: ok.

P: As a matter of fact, when I was informed of that case, when I went they couldn’t… well they said they don’t have the form.

I: ok

P: so I gave them enough anyway. When I came back to… with our people who are here, we have an RCH here, I went to them to… they are a bit ehrr about six or seven or so. I took all of them through it again and then gave them the, the forms.

I: ok, ok. So couldn’t that also be part of the reasons why some may not be reporting?

P: Ehrr, as a matter of fact, the one who reported was not having a form.

I: ok.

P: ok but she reported.

I: ok

P: Now…

I: through a phone call?

P: Yes, this with errh, our people here, is over a month,

I: ok

P: is over a month when I had the interactions with them. I gave them the forms but since then errh, not one case was also reported.

I: ok,

P: ahaan, we had a lengthy discussion, they asked questions especially on this issue of fever because on the AEFI form, we have fever there.

I: yes

P: Ahaan! And so with them, fever is normal so long as especially PENTA is concern. And so would you have to be filling AEFI forms for every child that you give PENTA to? It was a concern, but we agreed that we all know fever errh, presents after PENTA.

I:yes.

P: Ahaan, and so that should not be so much of a big deal order than that, every child that receives PENTA, we are going to fill AEFI form for.

I: ok. What about the FDA, Food and Drugs Authority forms? Do you have any forms

P: yes

I: front their point?

P: yes.

I: So how is the communication like with respect to their forms in the district?

P: aaah, in the district here, (*someone talking*) as at the time I came as a matter of fact, we had my colleague here informed me. We had one errh, adverse event as a result of the Avemetin.

I: Ok.

P: Ahaan, so in collaboration with the FDA, I had to…. she had to fill the form, they had to take some sample to the national level and all that. But as at the time I came here, any direct link with FDA, I’m not even sure if they have any

I: ok

P: office in town.

I: Ok. But are those forms also available at the facilities?

P: The FDA forms?

I: Yes please.

I: That I can’t tell.

I: Ok.

P: I can’t tell because my… at my former place, it was the pharmacist who was handling the

I: FDA

P: yes, the FDA forms.

I: ok. and that isn’t the case over here?

P: errh, here, the pharmacist is gone to school but we have errh, a staff who is errh, taking care of the office.

I: Alright, ok, amhm, so when those forms are filled, to your desk for onward transmission, how are they stored?

P: We file them.

I: you file them?

P: yes.

I: hard copies?

P: Yes.

I: ok, ok.

P: But we also have the soft copy.

I: Ok. Ehmm, what suggestions do you have, that you think when adopted can help improve upon the reporting of AEFI?

P: Improve on the reporting of AEFI, I think that some, some of the, the erh, signs and symptoms should be re-looked at.

I: Ok.

P: Because as I mentioned in the case of fever for PENTA, it is the normal thing

I: ok

P: and so the staff who know that with PENTA, the child is going to have fever, and yet on our AEFI form, it is stated you have to errh, indicate if the child has fever and that also qualifies for AEFI. I: yes

P: it will mean that, they would be filling more than errh, necessary forms and so they will not even fill it at all. So long as it’s just fever.

I: yah

P: If it goes beyond fever and may be an abscess, errh, a lump, something of that sort, they may report it. A rash or anything of that sort, they may report but something like just fever, errh, pain. Ehmm, I foresee is one of the reasons why they don’t even report it.

I: ok, any other thing you think we can, we can do to improve upon the reporting?

P: ehm, it is also on my part as the district EPI focal person to be constantly reminding them of it. I think that is also one of the things that we could do.

I: Ok. Any other thing?

P: I think that is basically, what I think we should do.

I: Do you have any other thing you want to say with respect to AEFI?

P: With respect to AEFI?

I: Yes.

P: Ooh nothing more, nothing more.

<Internals\\IDIs DHMT\\VRDI_10> - § 1 reference coded [7.84% Coverage]

Reference 1 - 7.84% Coverage

I: ok, ok, emmh, do you think, emmh, are there ways you think we can help improve the system as far reporting of AEFI cases are concern? Do you have any suggestions?

P: Actually for, for me, the only ways that you can improve upon it, that’s what I have already said, that there should be a frequent reminder to our health staff because they are having close contact with the children which we are, as a municipality are doing. So to me, I think there should be errh, always reminding them, if there is a gathering as we’ve being doing, we should continue to, to enlighten them more about this condition which they must also report as they are doing.

I: Alright, any other thing?

P: now, I think errh at the training level, at the school level, we go through all these, so if it depends on the level of the school, institution if they think for some of the institution, the emphasis is not on the AEFI but if for some school, they laid emphasis on all the EPI conditions.

I: ok

P: So I think if there is any other school that or any institution that is not taking them through all these issue, they have to start telling them from there before they complete the school and comedown.

I: Ok, any other issues you think you might want to share with us with regards to the reporting of AEFI?

P: Reporting to the AEFI, I think that’s all we shared,

<Internals\\IDIs DHMT\\VRDI_11> - § 2 references coded [6.82% Coverage]

Reference 1 - 3.68% Coverage

I: Madam please what are the ways, what do you think we can do to actually help improve upon the entire reporting process as far as adverse events are concern?

P: I think errh, we need to re-orient our officers to have a better understanding on the need for them to report the adverse effect and then errh also taken them through what an adverse event is. And then errh making the form, forms available to them. And then also our monitoring.

I: Ok

P: yes monitoring and then errh continuous good orientation.

Reference 2 - 3.14% Coverage

I: Ok, Ok. Madam please do you have any other thing to say with respect to the adverse event following immunization?

P: What I would say is that we need to be reporting so we will also have to attach some kind of seriousness to it. And be on our officers and then make sure every month, they summit report even if there is none, they should give us zero report. So that will make them aware that they have that responsibility towards us.

<Internals\\IDIs DHMT\\VRDI_12> - § 3 references coded [17.99% Coverage]

Reference 1 - 7.95% Coverage

I: Ok, alright. What do you think we can do improve upon the reporting of AEFI from our facilities?

P: We need to sensitize the health staff as well as the caregivers.I think the caregivers should be more targeted because if they experiences those things, fever and, and we know that as for fever is one of the side effect of those…ahaan, but if there are some other untoward effects that have been experienced in the house and if we don’t tell them, that these, that if you see this, if you see that, then you can come and report to the health staff, they will not do that. So we need to errh, target the nurses as well as the caregivers and maybe the training should be in–depth for the CHNs or the…, whoever will give the immunization or any drug. Then we the supervisors too, we have to play our role as supervisors not only getting the name as a supervisor and then sitting in the office but.

Reference 2 - 6.22% Coverage

I: ok. Please do you have any other suggestions you think can help improve upon the reporting?

P: And then the forms too should be made available, like if should be in a booklet form so that, when-they finish with a booklet they can request for… but if you tell them to go and photocopy, ahaan taking out their 20p, is something that they value so much so they will not… so they will not report. So I if can’t photocopy then, I better not report on it. So we should… it should be like a booklet form, then given to each facility. We encourage them to doing that because with that, it will help the system, to help the manufacturers to know that ooh this batch of product has something errh with it so.

Reference 3 - 3.82% Coverage

I: ok. Is there any other thing in general that you want to say with respect to AEFI?

P: errh, we know that with the cold-chain, if it has not been met, there can be AEFR I, so the EPI or program managers should do well to give out vaccine fridge to all facilities, all CHPS zones so that districts will not be tasked to maybe go and buy these domestic fridges for facility. There should be vaccine fridges for all the facilities.

<Internals\\IDIs DHMT\\VRDI_13> - § 2 references coded [14.55% Coverage]

Reference 1 - 9.13% Coverage

I: [bleating] (laughs) eeeh please eeh please then let me know …..Your suggestion [bleating] for improving the process of reporting AEFIs?

P: Mmmm ……..ok l will suggest that we keep eere hammering, counselling the care eeere caregivers they are the mothers we let them understand so that they report on time that they don’t delay then we so need to be motivated enough you know when eeere a case come to you you need to move maybe the means of transport is not even there for you to move so you also sit at your corner and be trying to do your own thing as to how best you can also manage at times u need to follow up but with the means for you th follow up is there what do you do and the terrain looking at the terrain you get to the road side to a taxi to a facility you will be for more than five hours ’’ kraa’’ you will not get any car if the car didn’t get full you cannot contract the car all alone whiles there is no money in the system right now so.

Reference 2 - 5.41% Coverage

I: Ok ok sir please eere now tell me other issue that you would like me to know with regards to reporting AFFIs in general?

P: Yes all that I was saying that need to constantly be doing rehearsal training for the staff you know every year we receive new staff so if we can be getting funds to be training during their orientation we constantly take them through the AEFI forms it will help then also we need to eere either designed an account purposely for AEFI so that there will be constant availability of funds that will propel you to be moving on that direction.

<Internals\\IDIs DHMT\\VRDI_14> - § 2 references coded [19.17% Coverage]

Reference 1 - 10.74% Coverage

I: Ok, ok eehe madam please let me know your suggestion for improving the process of reporting AEFIs?

P: Mmm ok

I: What do you think we can do to improve the reporting process?

P: The reporting, then it means we the officers here have to put on more moral or have more vitality to integrate it in every activity that we do like [ loud noise in background] our monitoring anytime we go this thing must be included it suppose not to be once a while.

I: You, you this thing you mean the AEFI?

P: Aaah AEFI must be integrated in our monitoring it suppose not to be maybe once a while but always any monitoring we do we have to include it the AEFI and also once a while the region or national have to come maybe they have new ideas or new way of reporting and they shouldn’t channel it to only the disease control unit today you have come to this unit it’s a long time l have heed of even this aahha so they should do the thing across so that every officer have to be aware and anytime even if you are on your rounds just you want pay a facility a visit you can educate them have more knowledge on it and educate the people and I think they will start reporting on it mmm that is my view.

Reference 2 - 8.43% Coverage

I: Ok please tell me any other issues that you would like me to know with regards to reporting AEFIs in general, what issues will you want me to know?

P: (laughing) well l don’t have much issues oo the issues is, the issue is like ehmmm l have said it all (laughing) we we, we have to work hard like now we to be giving education add to our education talks to be given to the people on the AEFI its not only the child have to have the bigger absence before bringing the child and mmmmm l think that’s all (laughing).

I: Ohh madam l can see you have a lot to talk,

P: Eehemm (laughing).

I: elaborate expand more, tell me more.

P: Mmmm mmmm and all also we, during our home visit we have to be asking mothers or caregivers ahhaa those we know that they came for the immunization when we get to our various home we ask the child was he or she worm was she having hard in the tire or reddish if it is yes then that is it mhumm.

<Internals\\IDIs DHMT\\VRDI_15> - § 2 references coded [10.47% Coverage]

Reference 1 - 3.78% Coverage

I: Ok madam please let me know your suggestions for improving the process of reporting AEFIs?

P: Yeah l will say maybe the motivation we can give to them is to train them if we train them and it’s like emmm a formal reporting they will also be happy to report.

Reference 2 - 6.69% Coverage

I: Madam pleases eerre [laugh] eerre please tell me other issues that you, you would like me to know with regards to reporting an AEFIs in general?

P: Yeah the training will serve as a motivation to the staff then like we said the feedback, we need feedback what we have reported are we on line to what you expect from us so you tell us something also when there’s AEFI and it is beyond our scope we actually want the regional level to actually support us, yes.

<Internals\\IDIs FDA\\GAFDA_01> - § 2 references coded [12.41% Coverage]

Reference 1 - 10.19% Coverage

I: Okay, so please could you let me know your suggestions for improving the process of reporting AEFIs?

P: Improving the process

I: Yes, your suggestion to improve the process?

P: [4 seconds] as in the manual reporting or…

I: Yes the process of reporting AEFI from the…

P: From the…

I: Down till it gets to the national level, the whole process?

P: Am sure ’erhm’ when we continue to create awareness it will let them report like you are already doing.

I: Okay

P: So we need to constantly remind them that they need to report so that ‘erhm’ they know the reporting pathways. Some of them are already aware but they still don’t report that you will find out and tell us and ‘erhm’ we just need to continue to remind them to report.

Reference 2 - 2.22% Coverage

I: Okay ’erhm’, please ‘erhm’ could you please let me know if there are other issues regarding the reporting of AEFI that you would want to tell me about.

P: No.

<Internals\\IDIs FDA\\GAFDA_02> - § 2 references coded [23.60% Coverage]

Reference 1 - 13.57% Coverage

I: okay. ‘erh’ please can you let me know your suggestions on how to improve the reporting of AEFIs.

P: How to improve ‘erhm’?

I: The process of reporting AEFIs

P: [3 seconds] [coughs] what I can think about is to continue to tell them why they have to report everything whether they are familiar with the AEFI or they are not familiar with it, they have to report everything that they come across. Secondly, what we start doing is that giving them targets. You know, WHO has a target that okay for ‘erh’ number of ‘erh’ surviving infants, this is the number of AEFI that should be reported. So what we started doing to shared up to the regions and the regions look at the district and share this information with them, that okay! If this is the surviving infants at the regional level, these are the number of AEFIs that’s expected, I mean from the reporting system. We’ve done that and they show you the regions and we hope this will motivate them in a way to report and say that ‘oh’ if you are not reporting then it means that you are not doing well and then the third thing we plan to do is to share information from all other regions…nationally with everybody so that okay you say that ‘oh’ maybe Volta region is reporting, Northern region there is zero there so you say that ‘oh’ yeah! Maybe we are not; there is something we are not doing well. So these are few things that we are doing at the national level. To make sure everybody report the AEFIs or health workers report the AFEIs. [Phone rings in background]

Reference 2 - 10.03% Coverage

I: Okay, ‘erhm’ please are there any other issues concerning the reporting of AEFIs that you will like me to know…in general?

P: What we are concerned about is the reason why people don’t report and we are trying to put these strategies in place to improve reporting. ‘erhm’ as I already explained, we started sharing information on what we received with the regions, and then to the districts and then within the FDA. What we are doing to improve reporting, there are few things that we started doing. We give our regions targets, not based, not on number of AEFIs that you should submit, how to, it’s about system strengthening. So we believe that if the system is improving, if they are doing what they are supposed to do at the regional levels, people will report. So things like okay we have to train ‘erh’ you have provide this number of training to health workers on AEFI or adverse drug reaction per month, we have to follow up to the facilities these number of times per month. So these are some of the things we are doing. But the target to the, our offices are not based on numbers of AEFIs [phone rings in background]

<Internals\\IDIs FDA\\NRFDA_01> - § 1 reference coded [12.35% Coverage]

Reference 1 - 12.35% Coverage

I: ok, ok, please tell me other issues you will like me to know with regards to reporting Adverse Events Following Immunization in general?

P: oh, for instance these days we have what do we call it, fluctuation of power, so when we go to facilities we always ask of their back up for power in case there is a light off or power outage for a long period of time , how do the keep because we visit some the districts and then the tell as when there is continues power outages and then there is a very big challenge, the try to move the vaccines from where they are supposed to store them to other places where we cannot really authenticate whether the temperature regime there, the cold chain there is not broken so these are some of the challenges we are confronting if every may be district health directorate can get may be a very reliable power source like a generator.

I: ok

P: I think that will also help because most of these vaccines when there is cold chain break, trouble it means the vaccines will go bad because they are supposed to be stored under a certain temperature regime when there is light off for a long period of time, it means this condition cannot be that will endanger the vaccines which in the end can even endanger the lives of the children or clients who are going to take the vaccine so I think that is where we have a little challenge and we are trying to even talk to the regional health directorate to see if they can help you know in the procurement of power generators in every district such that when there is power outage all these conditions can still be intact such that we have a safe and quality vaccine on you know on the market so that when people use them the will not have such problems. For now these are some of the things I can talk about.

I: is there any other issue concerning vaccine safety in your region?

P: for now I don’t think we have any, “ehhh” anything that we will talk about will general, I think in Ghana is just not an Island, whatever happens in Upper East will be same condition in Northern region same condition in Upper West so basically these are some of the issues that we confronting with.

<Internals\\IDIs FDA\\UEFDA_01> - § 2 references coded [19.59% Coverage]

Reference 1 - 5.96% Coverage

M: what would be your suggestions to help improve the processes of reporting adverse events following Immunization?

R: I think training which is on-going should be intensify and education. I think when people are made to understand the importance of reporting these adverse events that alone is enough motivation for them to report. When they understand that this is the reason why somebody has come back to complain, I have run diarrhea or headache or I have had this as a result of taking the medication. Then you report it because when we collate these thing the go into the system where if many people have reported on a particular drug. Then they can look at it to see what the reason is, what are the likely reasons why many people are reporting on this. Then may be the technical advisory committee will advise on the next step.

Reference 2 - 13.63% Coverage

M: Now would you please tell me any other issues that you would like us to know with regards to adverse events following Immunization in general?

R: Oh talking about Immunizations normally, let me say national events we all are well prepared for and that the issues are very limited because structures are being put in place to administer the drug and then to collate these adverse events so that the comes with less challenges especially in this part of the region where Navrongo health research is there. The volunteers are well trained. Another aspect aside this general one, I think the volunteers from the Navrongo health research that you people trained and used, I think the regional health directorate will have to train the care givers at the grass roots to understand what it means by adverse events, the importance of reporting adverse events to the health facilities and then somebody also being selected to also receive these adverse events and we will also forward them because we are not in all the communities we tried to have institutional contact persons in all the CHPS Compounds but the communities that do not have these CHPS compounds, how do they report to the CHPS compounds? So we think that if training could be extended to them the care givers at the local level, the community level we don’t take things for granted.

If you take paracetamol and as a result you have swollen and all those things, you do not attribute it to any spiritual or cultural kind of thing you just report that I was sick I was given paracetamol and as a result I am running diarrhea. Generally a lot of people do not read instructions on the labels of these medications so sometimes some medication are there, do not take milk, do not do this when taking this medication so that results into other events. But we say report everything to the health facility and they will also collate the forms and it will get back to us.

<Internals\\IDIs FDA\\VAFDA_01> - § 3 references coded [47.57% Coverage]

Reference 1 - 8.89% Coverage

P: I am not saying it negatively

I: yea I get it

P: but there should have been a collaborative whatever procedural. You see, what that ICP is doing must be recognised by his hierarchy as performance. But I don’t think it is there. The performance review we attend actually goes to the level of facility, but as to an individual’s performance, I don’t see it there. So when the person is actually… they have their indicators for performance of staff, staff performance. They have their indicators, so if it doesn’t actually errh, errh, necessitate going extra mile and then one your procurement procedure, your distribution in the hospital, they are looking at your field, whatever, your medication list, always making sure, availability. They are looking at these whiles they are not looking at adverse drug reaction, number of adverse drug reaction you’ve actually whatever, you monitoring medication error, how many education have you done and how many wards to I mean to uplift the level of science practise in the system? They are not there, so monitoring and evaluation. I believe if these… we are easy to state that, there is errh, errh, errh under reporting, there is underreporting but this is the first time I’m seeing us trying to evaluate why underreporting.

I: ok

P: Some of them, they are not written on paper, nobody will write it on paper. (*We laugh*) It’s within them. So I believe these are some of the things, we should put structures in place that will actually bring up the reasons. I happened to have been engage to give speech in Togo on counterfeiting and I made mentioned of the system in Ghana and they didn’t take it well because if you…, a hospital, if you report and then won’t they come and shut your place down? Won’t your clientel feel you are poisonous to them *(he laughs)*, so they will stop coming to your facility. So it’s better you keep quiet and you work. But will it help the system? As I speak to you now two gentlemen came here with pad, the one the women use

I: ok

P: and according to them, one of them wife used it and rashes in her pubic area, so they brought it. We wanted to pick it on a blue form for an assessment, for an evaluation of the product, if the importer will be call upon to make an adjustment to whatever it is. We wanted the facility to put it on… up till now, the medical officer there doesn’t want to give the form, so the samples is as over there *(pointing a finger at the sample on a table).* The two gentlemen are always coming and we are waiting

I: for the report from the medical officer.

P: yes, yes. So you know you can go coercing him to fill *(he laughs)* a blue form. It should be voluntary. So these are some of the issues. But with Ghana Health Service, I believe there should be a recognition of the ICP officially.

I: ok

P: The term ICP has coined by FDA. When you look at the hierarchy of Ghana Health Service, you won’t see ICP; Institutional Contact Person, it wouldn’t be there.

I: yes

P: It wouldn’t be there, because you can have an ICP being a nurse by reason of where the person is. For a TB, whatever facility you see an ICP being a nurse.

Reference 2 - 0.82% Coverage

I: Ok, alright ammh, do you have any suggestion for us, what do you think we can do apart from what you said earlier to improve upon the system, the reporting system?

P: The reporting system, as I speak now what I will suggest is the structure of the Ghana Health Service, the recognition

Reference 3 - 37.86% Coverage

I: Ok thank you very much. Do you please have any other thing you think might help?

I: Errh, for now the team that started this adverse whatever, errh, the spontaneous reporting, that’s the safety monitoring which involves spontaneous reporting and then AEFI and all that, have done some job, tremendous job till where we are but we’ve mostly depended on passive reporting, So I’m here expecting that when that doctor if he has observed something, it is his responsibility to come and report to me. At the end of maybe a period that I call; “haven’t you actually observed anything?” He says “no I’ve not gotten anything”. It’s passive, so passive but if it will be made active. Active reporting, but we know infrastructure needs are also there. So then, we will look at how to offset some of the challenges and problems. Now they manage to put the erh, erh, errh E- Reporting,

I: ok

P: so you register and then when you observe anything you key it in and it goes straight to the head office.

I: ok

P: When it does that it also errh, errh… there is a hotline that it follows, it goes to there. So the regional focal person is also left out. *(Both laughs).* You see and there are certain times that, there are investigations with this.

I: ok

P: So it means that report will go, the head office finds it or deems it necessary for an investigation

I: it comes back to the region

P: and then it comes to the region and is novel to the person. He doesn’t know what has of one on, so he goes in, the little that he can…. Aside this too, aside from making it active where it becomes mandatory for every medication that is over, it becomes mandatory on the manufacturer to follow-up and also make whatever. Wherever your product is should be responsible for its movement. *(A knock on the door. R: yes come in)* wherever your product is, you should be responsible for its movement, its storage, its transport, everything. I believe they should errh…., The QPPV that is the trained guides is also now coming up and I believe we will get to where the UK and those people have gotten to.

I: ok

P: Yea and then I also believe if we could look at erh, erh, the reporting format, erh, to also honour, I mean publicly honour the people that are doing the good jobs and then, you know in all these we are talking of morbidity due to medication, what about mortality due to medication? Are we interested autopsy reports? Is there a way that these things have been observed or like how the narcotics guys are interested deaths due cocaine swallowing? Even the picture, so they use for education. Are we interested (*he laughs*). So I believe maybe we can also get to those stages and then… Death is inevitable but if deaths are being due to some medication, we should see it and then avoid them. We can say we are not observing that, because there are some deaths that are occurring due to under dosing.

I: ok

P: Is not a quality issue, is under dosing is a practise issue, can we correct them? So maybe you’ll one day, one day as we keep developing, I believe mortality due to medication will also come up.

I: ok. Emmh, you just drew my attention to the electronic platform for reporting. What is your take on that?

P: It circumvent *( Both laugh),* it circumvents the regional guy.

I: yes but don’t you think it can be re-tooled, re-tooled such that it will pass through the region?

P: Yea, that’s due to monitoring and evaluation. That’s what you are doing. We both have actually observed that is actually circumventing the… and when there is the need for a re-visit on the issue, errh, looking at the geographical terrain, the person in Accra that received that initial report wouldn’t know of the terrain.

I: yes

P: You are in Bolga and you are talking of going to Bawku hospital, and may think it’s just like from Kaneshie to Circle *(laughs*). Which can take you a whole day,

I: yes,

P: yes it can take you a whole day and looking at even where we are now from, if somebody report from Kpassa and then you are asking me to fellow-up to go and do it, it will take me a whole day. So the regional focal person knows of the region where he is, so he should be part of the whole report system. Now if you go and then the person tells you, I’ve being reporting to your head office. Which means I don’t have the hard copy. You see and he will tell you that his structure is also demanding that he sends his report direct to his director at the health directorate. So the person also is there and doesn’t the sense of *(He laughs*) FDA, whatever. So sort of things must be ironed out. It’s for the good of all of us and I don’t see the success of it being celebrated by just one institution, is a team work. Yea when I was in Bolga I think I. . . . was it Dr. Yeboah or I think there was this autopsy thing I wanted him to take us through, that’s the investigations that go on after mortality set in, all that but it was so detailed (*he laughs)*. I, I, I sometimes when I look at my archives, I see the forms he gave me. *(Both laughs).* I see the forms that he gave me. There are legalities that also go with some of these things so, some of the times there is hesitation but I believe one day we will come over this not to think of punishments but more about rewards.

I: ok. Is there any other issue that you may like to talk about?

P: I’m looking at a vibrant reporting system but reason of review of how is being done so far and how collectively errh…. I believe in collective discussion with all bodies involve. Errh, erh, we may be here and we may not think that the tax person called the Ghana Revenue Authority is necessary in what we do but if the Ghana Revenue Authority person does not call attention of the erh, regulator that there is tramadol that is not what you have registered coming and then because it’s call tramadol their system picks it and then it passes and enters the system. So he is a tax collection alright but I think we have to re-think because when you say tramadol and then their system picks it as tramadol so is filled and people are just misusing it. So I believe we should look at the stakeholders, the stakeholder errh, errh, emmh, how do I put it? Review should be done to include all that is necessary in what we do.

I: ok

P: yes. There may be remote stakeholders. There are remote ones.

I:ok

P: Yes there are remote stakeholders.

I: ok. Emmh, before I, we finally wrap up, how has being the working relation, I mean the FDA with the health directorate in the region?

P: Is very cordial, because most at times, the errh, headship of the… we go through the headship of the pharmacy

I: ok

P: which is mostly a pharmacist and understand all these things. So is always cordial. I enjoyed it whiles I was at Upper East Region, over here I’ve enjoyed same.

I: ok

P: is cordial, very, very cordial. Should I say friendly, yes but where procedures and structures come into play then observation of the procedures is what is actually where we should look at,

I: ok

P: yes, where we should look at. For instance, emmh, the CHPS compound person is a nurse under the director of nursing service,

I: yess

P: she would like to report through her boss. So we should look at….. and then if your boss says you should report to me before anything gets out of my system, so that when I’m also called upon, I can say something. Then it becomes the time that we are looking at, because we want to be timely in reporting,

I: yes

P: so it is defeated. Is it possible us to make duplications of these so that initial one goes but we still end up reporting to our superior?

I: ok

P: I don’t buy the idea of you usurping the authority and then doing whatever. I don’t buy into that, but is it possible that it will be given re-organisation or like you said re-tool, that we send a copy to our boss and then whichever. Is it possible to use the e-….., soft whatever so that if it is whatsapping, he gets it whiles I’m getting it? We go paperless, because printing and what not at the end of the day we will have a room full. Maybe some ten years’ time, you come and I’m sitting on…., instead of, of a chair, I’m siting forms, (*laughs)* I won’t get anywhere to put, (*laughs)* you will be sitting on a form. So is it possible to also look at the way technology is going, to also have a virtual library?

I: ok

P: Forms that have been received can be scanned and then put into a virtual library. Is, is….. I understand there is one call cloud or something but I was asking of document security and I realized people can hack into it. But is it possible to create, we, we….. In UK the libraries, even Cokrain has a library but is not physical, it’s a virtual library. I think we should be getting toward something like that where somebody at the CHPS compound is not waiting to see blue form to go and see definition of a case but he can use his whatsapp and then see that this is case this, this is what’s it means. Evidence based medicine is using this softwares, so can we also get to that level? Can we get to that level of practice? This is a challenge, it’s a challenge.

I: Thank you very much. Emmh, is there any other thing you may want to share?

P: Money *(both laugh)*, mot, mot, motivation.

I: motivation

P: Motivation doesn’t only come by reason of money but recognition of your contribution. Let me just sight you as an example where I will say you’ve had to travel from Upper East to this place in order to get the opinion leaders to see what is happing on the ground.

I: yea

P: It’s not in the negative sense, so even though you might not have been part of the group that designed the documentation and what not, but you are looking at the further advancement of the use of those documents. So you are actually sacrificing and it’s wealth I mean mentioning it. Even if not being given physical erh, erh, whatever, it should work. Ehern, it should be recognised at a certain stage. Others are doing a lot.

I: yes

P: I was in Upper East, somebody went to eat Kenke, happen to be at the hospital, they didn’t….. They actually cannot identify what was the problem, so I had to be called in to Navrongo, War Memorial to go and observe, only to quiz him and realized that the guy ate shrimps and then he reacts to shrimps, so the mouth was swollen. You realize it,

I: yea

P: so and even Bolga to Navrongo, you are not talking of a stone thrown.

I: no

P: You talking of a distance and some of the times the call that, at the time that you receive the call and you had to leave whatever you are doing to actually attend, come back, all these things in whichever level the person is whether the director, whether the national service person, whether is a first level errh, nursing practitioner or whichever practitioner. I believe we should look at the motivation. And I also believe if we could errh, train, we could add the research bit of, errh, research and then reporting on some of these things if the… In Upper East, we manage to develop a newsletter of the errh, erh, Bolga hospital,

I: ok

P: so errh, people could write in whichever way but as you keep writing l believe you develop

I: yes

P: so we could do that. We have people reporting on various things and then it even becomes motivational. He wants to find out what is happing so you could develop a paper but who guides him or her? So you look at Lancet and you don’t see many Ghanaians writing, I believe we could also use the safety monitoring as a platform to get people to write up.

I: Alright thank you very much. Your final words.

P: Final words,

I: and we draw the curtains

P: I believe safety monitoring has come to stay.

I: ok’

P: There is no policy that does not have hitches but as we observe and we make amendments to them, that’s what will perfect the system.

I: ok

P: So I believe we should contribute our quota because we giving the medication, if we don’t find out the effect of it, some years to come we suffer the thalidomide thing. It might not be immediate but you and I may up coming up with other forms which is not observable physically. For all you know I we are being told not take errh, errh lead and whatever but this thing is in the erh, erh,cooking port. How many of us are mad *(Both laugh)* by reason of these things in our brain and not knowing…., so you talk to somebody he flares up may be he is mad ooh *(Both laugh).* I’m picking that as an example but we can broaden that thing and then see our reporting will go a long way to help the whole situation. So I believe we should encourage professionals to report

I: ok

P: but the patient reporting should also be stepped up. The patient reporting, the patient still feels if he reports, he has given up on…., he has given his prescriber out. Yea, so the patient doesn’t want to come out

I: report

P: yea report but if they should be made to know that, the patient-prescriber relationship still holds. Because some of them, the prescriber is there is their family doctor

I: ok

P: so he, he or she believes if I do so, I may curtail the relationship and when I’m not well, this prescriber may sake me .So let me keep quiet and suffer. So there are still issues,

I: yea

P: there are still issues. So this my final word that this relation, even though we are not directly involve in that prescriber-patient relationship, we should pay attention to it.

<Internals\\IDIs PROVIDERS\\GAPI_01> - § 1 reference coded [7.57% Coverage]

Reference 1 - 7.57% Coverage

I: So how can we improve the overall process of reporting among health workers and caregivers?

R: Education for both the mothers and the health workers, and making the form available like the blue form I don’t know if they have a different form or they just don’t want to fill the form. They should try I know forgetfulness is there but if the work is too much for them they will not be thinking about it, and the mother has the card that has the information, imagine they are just two and the work burden so it is not easy.

<Internals\\IDIs PROVIDERS\\GAPI_02> - § 2 references coded [20.69% Coverage]

Reference 1 - 10.77% Coverage

I: So how can we improve the overall process of reporting?

R: From the mothers side when they come to the CWC, education is given a lot is said so they should try and add AEFI, the vaccines contains antigens and they cause so so and so, they have to be educated on AEFI what they may experience after he vaccine is given so that they have that prompt idea for reporting, sometimes too they should bring their relatives so they all have the knowledge in case the mother forgets the other person will remind them that this what the health worker said and I think it will help and on the part of the health workers we have to keep, on keep on even if we have 0 we have to report, most of the time they don’t do it and there should be supervision and supportive monitoring from the higher level to the lower level.

Reference 2 - 9.92% Coverage

I: So is there any other issue on AEFI that you would like us to know about?

R: I don’t think there are any other issues but I think during our campaigns we have to include AEFI and the disease control officers should demand even if we have 0 so that we know what is going on, fortunately we are not having such issues, we have to make posters and put it all over on the bill boards, hospitals and health Centres so that everyone will be aware and also the non-medical staff should also know. If there is any durbar we have to put a short training on AEFI that in case someone having such signs you should report as early as possible and we shouldn’t continue giving paracetamol paracetamol for three days. Education is the key and documentation.

<Internals\\IDIs PROVIDERS\\GAPI_03> - § 1 reference coded [11.84% Coverage]

Reference 1 - 11.84% Coverage

I: How can we improve the overall process of reporting of AEFI’s?

R: The process for reporting is not so cumbersome; it’s simple probably a form of texting or mobile calling will be helpful.

I: Are there any other issues regarding

R: Like I said if the mothers are promptly reporting on the issues then reporting will be fort coming but if they wait for like one month before they report and all that leads to delay so the mothers have to be educated continuously on the need to report AEFI’s when they see them and the nurses also have to be able to identify AEFI cases and act on them. So this are the few suggestions I would like to give.

I: Are there any other?

R: No, I think that is all.

<Internals\\IDIs PROVIDERS\\GAPI_04> - § 1 reference coded [16.48% Coverage]

Reference 1 - 16.48% Coverage

I: So how can we improve the overall process of reporting nationally?

R: Generally we have a reporting procedure, from the clinic to the sub- district to the district but one thing is that they should give a feedback so you know that we reported this and got a feedback so you also know that anytime you report you will get a feedback and it encourages you to report. But if you send a report and you don’t get a feedback you might think it is normal so you won’t report again.

I: Are there any other issues about reporting that you would like us to know about?

R: For instance, they normally provide report forms, so they should make it always available and when you came you saw that we have different forms so they can just make it into one so you know the particular form to use and also there should be education on radios so the mothers in the house also know that if they encounter such cases they can report and say I heard that if I bring my child to the CWC and there is that,that, that I should come and report so they also know something about it.

I: Any other thing to add?

R: That is all or there can be a jingle that if you send your child to immunization and you see some signs come and report but if you see it as normal, like you child has body temperature and you take it to be normal it might not be normal so more education should be done.

<Internals\\IDIs PROVIDERS\\GAPI_05> - § 1 reference coded [11.48% Coverage]

Reference 1 - 11.48% Coverage

I: How can we improve the overall process of reporting?

R: We improve the overall process by training, frequent training, feedback because a nurse spend the whole time brining the mother and child to the facility and the form is submitted from district to region to national and there is no feedback we are doing this or that, so if you are working and there is no feedback then it means the work is not so important to them so if we give importance to the work then we will also be reporting and also resources should be made available, we should resource the personnel for transportation and incentives, it goes a long way if the nurse knows that when I do this I will get something then it will encourage the reporting and they will attach some importance to it and finally feedback, as I sit here when I send the forms to the next level I expect some feedback that this is what they are doing about it.

I: Are there any other issues that you like us to know about?

R: I think your questionnaire has covered it all for AEFI this is our problems and we hope to improve reporting.

<Internals\\IDIs PROVIDERS\\GAPI_07> - § 1 reference coded [18.22% Coverage]

Reference 1 - 18.22% Coverage

I: we would also like to know, how can we improve the overall process of reporting adverse events following immunization? [Door opens and closes in background] [Talking in background]

P: I think the research from the top was, should put a strategy down so that all these forms should be there and all the time they should be reporting or calling. Whether there is any problem, so that, we too we will sit up, ‘hmm’

I: [talking in background] [door closes in background] so we are almost done with the interview. Before we finish everything, we would like to know if there is anything you would like to tell us or ‘erh’ you will like us to know with regards to reporting of adverse events in general

P: the feedback too is important; we don’t get feedback from the region where it is sent [inaudible] so that it will come, so that we will know that oh! What you sent, this is or they are taking action. It’s like last time when they were getting the abscess [talking in background] we thought it’s the vaccine…yes it came the same month and they were having it. So we thought, because the report went and we didn’t know whether it’s from the vaccine or is the, the, the, the caregiver didn’t give the injection well. So if it goes there, we should get the feedback so that we can also know what we are doing. [talking in background]

<Internals\\IDIs PROVIDERS\\GAPI_08> - § 1 reference coded [17.03% Coverage]

Reference 1 - 17.03% Coverage

I: So now we will like to know how, can we improve the overall process of reporting Adverse Events Following Immunization?

P: Overall process?

I: Yeah.

P: We need to start with training. Training is the very, the basic thing that we need to do, then the ‘erhm’ system that we follow to report should also be improved, then we should also have feedbacks from the level , like where it,it, it is referred to we should have it back from them what was done and how the case went.

I: Is that all?

P: Then education too we have to make sure we educate mothers on identification, because they are supposed to report to us for us to take it from there. So if they are able to identify the, the effects, the side effects and they, they come then we will be able to report it.

I: Okay, so we are, we are almost done with the ‘erh’ ‘erh’ interview; before we go we will like to know if there is any other issue you will like us to know with regards to reporting of AEFI in general.

P: [talking in background] Yes is, we need training we have to get for, for example last, the whole last year we didn’t get any training on and we are having new staff who have no idea of ‘erhm’ Adverse Reactions. So if we are able to train more staffs to be able to identify and report the Adverse Reaction it will be able to help us improve the reporting system of adverse ‘erhm’ cases.

<Internals\\IDIs PROVIDERS\\GAPI_09> - § 2 references coded [19.62% Coverage]

Reference 1 - 6.34% Coverage

I: then how can we improve the overall process of reporting adverse events?

P: there should be proper ‘erhh’ data management. At least one person should be, one or two people should be trained. Apart from the whole march training, one or two people should be trained that you are going to manage the reporting so when I get the information I will come. And apart from the people were, there should be… computerized system, yes that you will have the data on it, ‘erh’ a copy will be sent to the district. If probably to the DHIMS then we know what is going on

Reference 2 - 13.29% Coverage

I: okay. so we are, we are almost done with the interview ‘erh’ but before we finalize everything, we will like to know if there are any other issues you could tell us regarding the reporting of AEFIs in general

P: hmm… I, I, I will say they should bring some posters so that apart from we having knowledge other places like the maternity, the OPD, because somebody may take an immunization, sometimes others may link ‘erh’ the symptoms ‘erh’ signs and symptoms with the immunization. Others may not also link it with it just like we have [inaudible] schedule. Even if you don’t know, once you see this one you will be able to give and explain to the client. So they should be other posters. After taking this, these are the some of the things you may see. Report let’s say by two, three days, then we can tell other let’s say staff, all staff. Or if the person see it on the notice board “arh adier wei [inaudible]” ‘hmm’ because they can’t train everybody and they can’t train all the health staff. Mostly when these training come is for those at the preventive side, so the curative if there’s ‘erh’ policy or posters or something like that, they can also see and report

<Internals\\IDIs PROVIDERS\\GAPI_10> - § 2 references coded [9.92% Coverage]

Reference 1 - 3.43% Coverage

I: okay. Then we will also like to know how we can improve the overall process of reporting AEFIs?

P: the overall? One: the forms should be readily available in our consulting rooms, at the maternity, at every department should have the forms and also as I’m saying, the training ‘dier’ it’s very necessary for every kinda of staff. So that, everybody will have an idea about the AEFI and I think ‘mhm’, it’s okay [laughs]

Reference 2 - 6.49% Coverage

I: okay. So we are done with the interview but before we go we will like to know if there is any other issues you will like us to know with regards to reporting of AEFIs in general

P: ‘mhm’, yes! When we report the AEFIs then I think they should be a feedback. So the ways ‘erh’, ‘erh’, they should tell us that whether we should give them reminder or the, they should be a schedule that if you report for this case, it will take this number ‘uhuh’ for, of time or these months to get your feedback so that when you are not getting the feedback, we, we can do a follow up. And also phone numbers should be readily available so that anything we can call and find out whether our re, report is ready or not. Yes please

I: is that all?

P: yes!

I: you can probably add some more

P: oh, no, no, no, no

<Internals\\IDIs PROVIDERS\\GAPI_11> - § 1 reference coded [20.23% Coverage]

Reference 1 - 20.23% Coverage

I: so now having said that, how can we improve the overall process of reporting the AEFI?

P: overall reporting of AEFI? Hmmmm! Sometimes! Human nature, when the pressure is not on you then you too you relax. So the one who receiving too should be on the…maybe calling every ooh, I will bring your report, doing this. When it’s like that the person will be alert. But if you are not…they will say that this one is not necessary let me leave it. So you should pressure them to send the report and follow ups and monitoring. If you are doing monitoring then, I know they will be coming so I have to do my things right

I: before we finish we will like to know if there is any other issues you will like me know with regards to reporting AEFI in general. If something has not been mentioned

P: i think we’ve mentioned all…then maybe what is…the education, you understand? Education especially on the [inaudible] of the mothers, because some of them, you will tell them, you know our illiteracy! That’s where we could get the problem. So when we continue to give the education, give the education i think it will stick.

I: is that all?

P: hmmm! Like once a while the authorities should come in vans, information centres telling them. That one too will help. Because sometimes we have been giving it at the static session or maybe somebody is in the house maybe she has not, she didn’t come so she won’t hear so ,some ones a while when they go around then it will help. Sometime people go on this cinema vans or video shows to them so that when they see too they …oh! ‘uhh yes’ then they can at least rush and report when they see something like that.

<Internals\\IDIs PROVIDERS\\NRPI_01> - § 1 reference coded [29.07% Coverage]

Reference 1 - 29.07% Coverage

I: So how can we improve the overall process of reporting?

P: Mmm the if train, everybody is creating awareness to the parents and awareness other workers who do not have fair idea about AEFIs, then there should be feedback as well then logistics for reporting.

I: Yoo!!! Madam I will like you to tell me issues that you like me to know about regarding the reporting if the AEFI in general.

P: Certain times when you report AEFI is the feel like, the general perception is that may be you did like in case of a fever or abscess it is towards the service the provider you didn’t do your well that’s why there is an AEFI so through some caregivers health workers might experience or encounter the cases but the turn to ignore them because they don’t want report and will look like you didn’t do your work well that is why you are experience such things.

I: yoo thank you for participating I hope that is all what you have to give me or is there any other thing that you might forgotten.

P: If you learn from school and you come out and things like that are not done is only report and AEFI you don’t know what learn from school definitely is different from the field so there should always be coaching and support the health workers when you come out from school you still be like a “neeno” until you work for some time and get the experience so there should always be a routine supportive supervising to help health workers improve on their job.

Here per say the clients are always in a hurry to go to their farms the time you need to educate one client on the side effects or the AEFIs the others are waiting so certain time we don’t do it properly as its expected because you are trying beat down time for them to go to their farms if you delay they will not come you would be able to get them or meet the target the number you are forgetting so certain times because of human resources here I am alone I have a lot of registers to enter will do immunization I will go for the weighing processes too so at the end the education doesn’t go down will if you are not able to do properly for the client to understand and even if she have questions what time will she use to ask. So human resource is a challenge to us.

<Internals\\IDIs PROVIDERS\\NRPI_02> - § 2 references coded [19.34% Coverage]

Reference 1 - 4.42% Coverage

I: So how do we improve the reporting if you want that reporting to improve?

P: It’s just about collaboration with all the health staffs and then we and may be emphasis on the importance and the like you said how reporting of the issue, make it know to all staffs that is the challenge we are encountering low figures mmm and if give more reasons why we should report eeh, why not?

Reference 2 - 14.92% Coverage

I: So eeh eeh let say now eeh I’m still trying to ask you questions concerning the reporting

P: Hmmm

I: Would, you know, some of the reasons that account for reasons why nurses or health care workers do not report would it have something to do with administrative or staff issues or logistics?

P: Em let’s say is not staff issues or administrative issues but is just more education was not given en haa not staff staff issues or administrative issue.

I: So for you is basically lack of education. Ok so at this point I just want to ask you if only like collectively as a health family how do we improve the whole reporting issue because like I said in the beginning you know under reporting is the major issue so how do we collectively improve that?

P: I would say noo as I said earlier on let’s make it known to every staff that under reporting is a challenge so as it is a challenges, no health staff would hear this and then still stick to what he was doing. If we all know that we see how to improve on it.

I: ooh thanks you very much I have come to the end of my, my interview with you. I appreciate your contribution and your experience would count a lot when it comes to the time we will be analyzing this whole thing. Is there my anything that you would like to add generally?

<Internals\\IDIs PROVIDERS\\NRPI_03> - § 2 references coded [18.07% Coverage]

Reference 1 - 9.89% Coverage

I: How can we improve the overall process of reporting adverse events following immunization?

P: Emmm I think emm collectively, we have to be conscious about it, we have to be remaindered and then there must be feedback...... If there is a feedback on it people will know that oh is actually being worked on (inaudible) emmm for example I said the last case or ehh adverse report I submitted about a year or more ago , just recently they were calling me , F.D.A was calling me on it and I couldn't even remember those cases. I can't even remember anything about it and you call me I don't have much detail about the patient then you want to know what I did, I mean is so long, how can I keep those information in my head. I don't have the folder information to go and look at it and know be able to retrieve and you just call me and ask of the patients name..... and then you want me to give you detail of what I did a year ago about that particular, I don't see one patient …. a year ehhmm so I can't remember.

Reference 2 - 8.18% Coverage

I: Okay emm we are almost ending our interaction but I will like you to tell me any other issue or issues that you will like me to know with regards to reporting adverse events following immunization in general?

P: Emm in general, I have already captured it, I think is just the matter of sensitization, more education and then ehm feedback coming from emm the higher level or wherever emm who is managing the emm collating the adverse effects, their findings. Eherrr leting us having the feedback and the ehmm using the information to improve the system that will encourage people to report. And then we ourselves, I mean the health ... care providers also given more education to the parents and and then to people who are been given immunization to report back any suspicious or any ehm thing that happens to them after immunization.

<Internals\\IDIs PROVIDERS\\NRPI_04> - § 1 reference coded [13.63% Coverage]

Reference 1 - 13.63% Coverage

I : Any other way that can help to improve the reporting?

P : Hmmm that like is just the training. If they, if if if Ehhh they that’s why am saying some one night have the knowledge alright ( inaudible) into practice but if errrh errrh training is done it awakens people errh.

I : How can we improve the overall process of reporting Adverse Events Following Immunization?

P : [ motor tricycle passing] [ noise from OPD] overall

I : yesss!

P : ( laugh) that one thierrr okay sensitization ( inaudible) among staff and what .... the community also...... Sensitization is just key Ehher in communities and among staff So that they will all be aware of AEFI, so if they are all aware reporting will be easy. Is not any tedious thing ... If someone comes first , comes and tells you, if if the mother just say oh they injected by child yesterday and today she is having fever. She will come and say ohhh after they injected my child she has a fever she might even though she might know that after the injection but if she is sensitized she can come out and say it Ehher and staff too if staff are sensitize sensitized they know more about it even though they know it. They will also start reporting. [noise from cars passing]

I : Please tell me ... other issues that you will like me to know with regards to reporting Adverse Events in general? .... Any thing that you will like me to?

P : Nmmmm [noise from reception] Noo ....no anything apart from what we just said?

I : Yes! ...... may be there are other issues that youuuu will l. i. k. e me to know with regards to reporting Adverse Events in general.

P : Hmmm ........ no is like everything is almost covered there so.

<Internals\\IDIs PROVIDERS\\NRPI_05> - § 1 reference coded [17.78% Coverage]

Reference 1 - 17.78% Coverage

I: So how can we improve the overall process of reporting adverse events following immunization?

P: Ahrrhh it should start from the top ….. Errh from Fegion or whatever or National …. Errherr they should make it .... a policy …. That like every year …. Newly train staff like we all who are already in the system we should be trained but they should always do like what we were doing with IDT’s. like when new staff are posted they should organize work shops and train people on it ….. so with time you realize that all staffs are train on it, they will know the importance of it and they will be ready to report. Then from there we will now take it over from there and also educate the caregivers….. that this thing is very important ..... so when you come and we inject and give you immunization and this and this happen bring the child to the hospital and we, when they bring it to, you too we should treat them well and report it, when we do that I think it will increase like the overall errh errherr performance of the reporting system.

I: Please tell me …. Other issues that you would like me to know with regards to reporting adverse events following immunization in general?

P: Other issues that ..... your question again?

I: I say you should ..... emmm tell me other issues …. That you will like me to *know* with regards to reporting adverse events in general?

P: Errherr I think errh, arrrh …. For this thing to work, we have already talk of the training and everything …. And I think there should also be reward for staff who even have the courage ..... to report it, like if I should give injection and they bring the child back and I treat the child and go and reported it ….. in the form it should be encouraged. So even if there is award for that you realize that everybody will be eager to report it yes then, reward is not like in monetary terms or ( inaudible) even citation alone….. for ….. that person it will encourage others to also do same ..... yaah I think that’s the other thing that.

I: So with the reward [door opening] won’t it make people even force to report false re errh, information?

P: Nooo the thing is that eeerh just like you said. We have a Technical Officer here, …. So if you report false information she will see it … we have a Disease Control Officer at the District level an just at the District level too …. When it happen the moment the Field Technician reported to him, he will not just take it up from there he will *also .....* come and assess and see what truly is this, before report it. But let me add (laugh) no one wants that thing (laugh) like we are all matured. Who will like to report something in the first place is not a good a thing ….. so it will not encourage anybody to just intentionally errh report…. That we have encountered this, this what is what I did and I encountered it and am now reporting it. No one will like to even do that in the first place … yaah.

<Internals\\IDIs PROVIDERS\\NRPI_06> - § 2 references coded [10.67% Coverage]

Reference 1 - 5.32% Coverage

**I:** How can we improve the overall process of reporting AEFI?

**P:** The whole staff needs training, because I don’t know everything about AEFIs. There are people who can give us knowledge on those things and sometimes if they don’t tell us what we need to know then that is where the problem comes and basically the training is main thing to improve our knowledge on AEFI and that can easily make us more (inaudible) and forms too should be provided and made easily accessible and also when I report on an AEFI and I don’t get a feedback next time I will feel relaxed because I have reporting and don’t get, why should I keep reporting. In fact, feedback is very important, especially in health, because if I report and I am not getting feedback definitely I will relax in my reporting.

Reference 2 - 5.35% Coverage

**I:** Please, tell me other issues that you would like me to know with regards to reporting AEFIs in general. Any other issue.

**P:** I don’t have anything specific thing to say but the AEFI cases sometimes like I said; our clinicians adjust the cause of it. They are mainly public health, so when there are things like that they think it is just clinical, clinical, so they will not like you what is going on. Recently, I was telling colleagues that there are some cases they just need primol to care for it, so definitely we are able to train to let and all the Staff know that maybe, at least if a mother comes with fever and other things during your malaria testing you should also find out how long has your child received any immunization and those things are going to help [phone rings].

<Internals\\IDIs PROVIDERS\\NRPI_07> - § 1 reference coded [12.74% Coverage]

Reference 1 - 12.74% Coverage

**I:** How can we improve the overall process of reporting AEFIs?

**P:** Aaa... like, like i said already, the overall process of reporting AEFI cuts across all sectors. it runs from the house, the initial staff and then to the sub district or district level. so for this to improve, all these units will have to be informed so that if it is for the basic level, for us the nurses at the grassroot, like i said earlier we can intensify our education so that parents would report to the facility for as and when they get adverse effect and for us nurses like i said already, a training with regards this will help us a lot to be able to report appropriately [loud voice heard at the background] the district management team and sub district too should please be given a form of training that will also help that reporting for such is very important, so that even as and when we report , they will also see it as a duty to..., maybe to give us feedback as to what has been done. Particularly for a client that was referred and i think that would be appropriate. Otherwise we only see the patient come back healthy and walking about, yea but we dont get any feedback.so training will be of a great help to us.

**I:** Please tell me other issues that you will like me to know with regard to reporting AEFIs in general.

**P:** As a matter of fact i don’t think there is any other major issue that i should bother about. i think what i will want to acquire is, because we have not had any updated form of training. We are not sure as to whether every client who report to the facility adverse vaccination should be reported to the district or not. we aren’t sure and that is why for us we think a particular category of people who come to the facility without adverse effect should be referred not all. This base on the understanding of the entire concept and so that only what i want tailor across so that we will know every client who comes with AEFI should be reported. We don’t have any other thing.

<Internals\\IDIs PROVIDERS\\NRPI_08> - § 1 reference coded [7.06% Coverage]

Reference 1 - 7.06% Coverage

**I:** OK…, thank you very much, we are almost done. How can we improve the overall process of reporting AEFI?

**P:**  Overall…

**I:**  In general, how can we report the overall process of reporting Adverse Events Following Immunization?

**P:** We have sure the reporting forms are always available then, at the end of the month when they reports we go through to make and find out if there are no any reporting on it, we find out from the staff whether this particular they get a case like that because at times they get the cases but maybe at the end of the day they forget to even report it or remember that this case was reported and then to fill the form. So we will be finding we have to be asking them continuously to remind so that they will fill the forms.

<Internals\\IDIs PROVIDERS\\NRPI_09> - § 1 reference coded [8.77% Coverage]

Reference 1 - 8.77% Coverage

I: Please tell me another issues that you like me to know with regard to reporting AEFI in general.

P: They AEFI should be treated free of charge it must be it must be it must be simple and they nurses must make sure that all AEFI cases must be treated aside and must and must be known that that’s the only way that can bring assurance to the work that you do and give the mothers the confident that the work that you are given is very go to them.

P: and measures our potencies and the qualities of the vaccines or the drug you are given to the children.

<Internals\\IDIs PROVIDERS\\NRPI_10> - § 1 reference coded [12.04% Coverage]

Reference 1 - 12.04% Coverage

I: How can we improve the overall process of reporting AEFI?

P: as I said in if staff are taking through then training training on AEFI definitely is going to improve upon their reporting.

I: So please tell me other issues that you would like me to know with regards to reporting AEFI in general.

P:the other issues would have to do with feedback normally its good we get feedback from every level form the mother to the highest level so I think if feedback is also improve upon is going to improve upon our AEFI reporting

<Internals\\IDIs PROVIDERS\\UEPI_01> - § 1 reference coded [5.70% Coverage]

Reference 1 - 5.70% Coverage

I: so in general can you please tell me other issues that you would like me to know with regards to reporting of AEFIs …. In general any other issues

P: ….. yeah so … any other issues so basically I say equipping the right people with the right knowledge,so the knowledge is important so giving the necessary knowledge so they know what to do what time and at what point and the channels to fo to go to follow its very important because adverse events must be reported the should not remain at the facility level

I: ok …. errm this the end of my questions errmm do you have any other thing you want to add or

P: …. Yeah errmm … yeah so we would like to … errhh urge that … even even there can be some ... errh let’s say the the district errh focal person on this adverse events so so from time to time there can be routine monitoring to the staff when they come then they have some interacting with the staff tell them how things are supposed to be and then what to look out for uhuh you know sometimes the mind but once you are repeatedly being reminded that yes you need to do this you need to do that and then somebody with really keen interest and there should also be the provision of errh resources and logistics let’s say in terms of fuel motor bikes so that some of them you need to do follow up so you need motors and you need fuel to do that then you need errhh money for credit then you need money for the reporting formats and all those things all those things are important

<Internals\\IDIs PROVIDERS\\UEPI_02> - § 2 references coded [13.77% Coverage]

Reference 1 - 10.64% Coverage

I: So when you talk about like overall process of reporting adverse events, how can we improve the overall process of reporting adverse events?

P: Yes. As health workers, the overall one is we shouldn’t feel shy to report (Ok). You understand (Umm). We shouldn’t feel reluctant to report (Ok). We should always report if it comes. Because it’ll help the child, and it’ll even help you the health worker too (Ok). So we should always report.

I: So now we’re done with the questions, so umm would you like to tell me other issues that you would like me to know, with regards to reporting these adverse events following immunizations in general?

P: In general?

I: Yeah.

P: In general

I: Yeah.

P: But not this my facility?

I: Yeah (laughs)

P: In general, well hmm…that’s why I’m saying that in general, I think others don’t report because of the issue you don’t know your work. You understand (yeah). Because when you begin to report adverse effects following immunizations…adverse effects, adverse effects, adverse effects; there will be a question mark (yeah). Why? Is it that you don’t know your work (yeah), or you don’t know how to immunize (Ok)? You understand. So I think maybe probably, and others too maybe you can’t tell, but to me I feel like in general most people…will not like to report because of this issue. But to me I feel it’s a bad thing or it’s wrong doing practice, because you need to re report; it’s also important. So that when you report, if there is any action, they can take it on before it becomes complicated. You understand. So that’s why I was saying that you have to try, and if you people are to organize a workshop on that, you have to really cover everybody (Ok), so that when you talk on it… I think those of us…excuse me that’s in quote who feel shy to report, maybe might come out. (Ok). Because if you care to know, everyday new nurses they train them to come (Ok). You know mostly the old ones we’re in the system and we know (yeah). But those who always come out new, some of them it’s like I’ve just come so if I do maybe in-charge or…they’ll say look at…the he or she doesn’t know anything (yeah). You understand (yeah). So it’s high time…anyway when you organize you’ll talk to them so they’ll learn. So in general, that’s how I just think it is. But I don’t think anyone in our facility as for our facility we report (Ok).

Reference 2 - 3.13% Coverage

I: Anyway, I think this is the end of our questionnaire with you. Will like to thank you so much for your participation. Thank you so much.

P: But don’t forget – when you go back tell your superiors (Ok), they should organize a workshop because mostly you people come and pick the data, at the end we’ll not even hear anything again.So when you pick, once it’s of interest and important to us (yeah), and then I know Research it’s like you’re also into health service like that (yeah). You have to really pick it serious (yeah) so that things will move. But mostly I think the adverse effects doesn’t come like that’s why you don’t see us reporting them (Ok). It’s just maybe once in a while (Ok).

<Internals\\IDIs PROVIDERS\\UEPI_03> - § 4 references coded [20.27% Coverage]

Reference 1 - 1.59% Coverage

I: the job training ok. So how about the overall process of reporting AEFIs how can we improve that?
P: mmm that one if we also have electronic way of reporting it. be it through the mobile phone.because if it’s through the mobile phone you can just access the form there and you fill in and then lets send it. That one can also be faster and then it will aid.

Reference 2 - 6.04% Coverage

I: ok so can you tell me other issues that you would like to you would like me to know regarding reporting of AEFIs in general?

P: … mmm in general … (inaudible) most of the things they are just errh even with other AEFI whether being injection or even giving drugs or what. some of them when they … some of them let me say errh the drug side some of them they feel that when they come back they may give them more drugs.not knowing if they rather come back maybe it’s that particular drug that is not helping them.so in that way when they come back maybe you can change a different drug or even treat what they are already having problem.and then know that they are allergic to this particular drug.but some will some those who fear drugs they will feel like if I come back they may even give me more drugs or do something what and ever.so that’s why some of errhh in general some will not come back and you will not also get the report.mhm … and the other thing is that some also … fear because when you fill that one and it go there is no way WF… food and drugs board will not call you from the national or even the region

because when you fill it your phone number will be there, they will call and ask you question. some feel like if … when they fill and then they call and ask and they can’t answer some of the questions they feel like even if I don’t send it

Reference 3 - 1.64% Coverage

I: (laughing) … so this is the end of errm the our our questions here, I would like to thank you so much for your participation.

P: and before you even run out some of the things that even because it got to some time we run short of because (background noise) we got short of some syringes because we have different type of category of syringes for the various vaccines.

Reference 4 - 10.99% Coverage

I: category of what?

P: syringe needles and syringes, syringes and needles

I: ahhh (inaudible) with the syringes ok.

P: ehuh so it got to sometime we got short of the 0.5 syringes so you have you then have to improvise.so in improvising if you are in somebody who is experienced you will end up causing pain and a lot of discomfort for the children.and that one can equally cause … even though we for me I have we have not experienced one but sometimes when you are not experienced a lot it’s difficult, you either give less or you can give more. ehuh because syringe as I said they are in different categories (going through stuff) there was one lying here (going through stuff) ehuh this one is just an example, this one is 5 ml

there was one we have 0.5 that one given that one you give you can use it to give measles,YF, men A, pneumococcal then penta … then TD, that’s tetanus dipsedia and then we have one too that is 0. 0.05 and that one is giving BCG you can’t use it for a different apart from BCG the next one is either used for immunization for you either use it to (inaudible) the other ones or you use it to immunize, that one is for mainly for injection.that is people who are sick and have come you can use it, or even better still you can use this one for the ATS those like they have had an accident they come. But when you are to use this one for PENTA, measles, YF, because that means you now have to gage it in such a way that you will be able to get what you want.and you have 2 ml too, so sometimes there was a time we got short and then you have to buy and then if you don’t also have money to buy the 0.5 then you feel you get stranded and you have to improvise. and in the improvising if you are not very careful you either give more dose,or less dose because you have to estimate.ehuh so that was the challenge but currently like we have received some quantity that we that particular one (ok) because it was that particular one that we short (ok), and it was like it was from national.ehuh it was like our, we are now buying (ok) so because of we are now buying sometimes and health insurance too is not doing good to us (ok) because it will take several months they won’t pay (mmm),you go to medical stores they will refuse you because you are you owe a lot (ok) that you have not settled (ok) ,so that is some of the causes, and then when you immunize you need cotton (ok) there are things we have to buy we are now buying them which we use not to buy.

<Internals\\IDIs PROVIDERS\\UEPI_05> - § 1 reference coded [2.46% Coverage]

Reference 1 - 2.46% Coverage

I: will that be all on improving the reporting?

P: well and making sure that we remind, if we train and remind we always ask whether it has been done or not because it’s very important, training alone does not achieve anything, we have to make sure that it happens.

I: thank you sir, so how can we improve the overall process of reporting cases.

P: making sure that’s why I said making sure that everything has to be captured and everything has to be documented. If it is captured, documented then we have done it.

<Internals\\IDIs PROVIDERS\\UEPI_06> - § 3 references coded [36.55% Coverage]

Reference 1 - 11.61% Coverage

I: Okay, so would you like to tell us if you have suggestions on how to improve on AEFI’s.

P: mmhm yes yes so the suggestion that I will have that will improve the immunization processes and how to manage the adverse reaction is that, there shouldn’t be some selected few people to go for the training, a whole facility of more than ten people and one person has gone for the training you understand, why don’t they rather go come to each facility and train all the staff, select a day, this sub-district we are going to the facility you train all the, go to this facility do! Everybody will have ample knowledge about how immunization is done and the adverse reactions that we expect, you know if you are a clinician hardly will you come into immunization side excerpt that they have staff crisis that you have to go in even though you learnt something years back, that can be an outdated one. There can be current things that they have learnt that you don’t know so you cannot say you have enough knowledge to operate but when they have staff crisis you can come augment that, work will go on. I suggest that every facility should get the training and all staff should be part of the training. Number two, maybe the coordination, they should you know there is always some small money allocated but and because of that one they are able to involve some many people you understand so they are unable to involve so many people, so there should be enough funds so you can get enough people to play various roles, not one person to be playing a huge role that what we intend doing the last one that came off what I intend doing but there was no funds is that one particular community should be someone supervising there, another community another person is supervising there. Its better than one sub-district person supervising about so many communities, you see that its difficult. If they even call, if this person call you for something another person call another person calls you, what do you do?

You understand, it becomes difficult but that is what I intended to do but because there is no funds (claps hands in despair) those who are doing it, they want to do and take some small funds so who will you ask to do what for nothing, you understand, but if I had my own authority, I would have made sure that all the staff is involved, each community one staff is supervising that community if there is a problem he rush inside he or she rush inside to assist. Mmhm the overall boss is the disease control officer, he will now be the administrative head of or the coordinator of the whole program in the sub-district, it would have been very very good.

I: so if I get you, you talked of a disease control officer who is here at the facility, it means your facility is the sub-district?

P: yes..... Sub-district head

I: okay so he is resident here with you?

P: yes, even though he renders services to the other facilities within the sub-district because they don’t have disease control officers so the disease control officer who is resident in the headquarters is still in-charge of all the other health facilities.

I: so will he be directly under your supervision?

P: he will because there is a sub-district in-charge in this facility, uhuhh.

Reference 2 - 3.28% Coverage

I: will there be any other thing on how we can improve the reporting

P: mmhm that’s two and maybe we can also erase certain myths that they have. What happen is somebody you know when you inject somebody and abscesses develop, it’s like a boil you understand. Some people have a perception that when you have a boil and go to hospital and they inject you, you will die, even the whole of this community it’s there, not only here the whole of Kusaag here; zebilla, bawku, pusiga every where it is there they have that myth and so there is the tendency that they don’t want to report that particular case. It means that we have to eradicate some of the misconceptions that the people have I mean we are able to do all these things then it will also be okay. And that has to do with symposiums where you can tell them that look we are told ABC but that is not true, this is the fact on the ground and they will accept it.

Reference 3 - 21.66% Coverage

I: so how can we improve the overall process of reporting an adverse event?

P: improve the overall, now the one the report has a destination, yes if the one where it is coming to stay I must demand that report from the one immediately after me, the one immediately after me must demand from all those who are also working under him and it would move from that to the last person at my facility, so if there is demand and there is timelines too for submission at the end of every day if you have reported one report it you don’t wait for the whole exercise to be over and that is why I said like if director general is demanding that way, the regional director is demanding that way, the district director is demanding that way and now the district focal person who is working for director there will call all the sub-district leaders in the evening every evening if the process has started like there is a mass immunization and once the process has started, you call in the evening, sub-district leader how many sub-districts like seven or six, you call every sub-district leader on that immunization, am asking for my report on adverse reactions, if you have you give and when he takes those things you quickly compile and forward and it gets to the final destination, this is how it should happen, this is one of them. Number two is the resources am talking about, if am given enough resources if I gather the report and they don’t even call me I will send it. But if there is no fuel no nothing even sometime even staffs we have to use their own motorbikes for these activities and there is no allowances for the maintenance or for any other thing, you see that its demoralising and ... (laughs out again)... and here self rivers are there you go and have to cross rivers and a whole of things, you see if they even give you the resources and you are going through these things these hazards you don’t have a problem. The time that (laughs) you have to sacrifice yourself and sacrifice the limited resources that is meant for your family and your dependence you see that it’s a problem.

I: so all together do you think there are any other issues concerning adverse events that you would want us to know?

P: mmhm, there is nothing new, there is nothing new apart from all that I have said about the adverse reaction, we are more than ready and that is why I have indicated that those who will go and provide the care will tell them we tell the caregiver when you see ABC, report, if you give your number to them and they call you if the resources are there, you quickly move there. The education the symposiums that I said we organise and tell them that no, when you have a boil and take an injection you will not die, that myth is still there, it is difficult to even eradicate it but we can talk to them to understand. These are the things the challenges are there, if we are able to remove the challenges things will become smooth.

I: are there any other challenges you want us to know about?

P: mmhm, I don’t know, I think these are the challenges; the resources side and the coordination when you don’t involve everybody the coordination will be difficult, just like I said that the last SMC that came I took the pain to say that look once we as a sub-district, I will move to every facility to involve all the staff the nurses there involve all the queues there and ask them to assist the volunteers to do the work and everybody was on board and we made sure that yes if you are working in any facility know what SMC is and what the target group and what we are suppose to do, when you know that and there is somebody in every facility in-charge of adverse reactions and there is adverse reaction the person number there is given to all of them, you call and refer the person and we manage and at the end of the day we are all successful but because of I want to belief because of lack of resources that you call one person from a sub-district or about two three people from one sub-district to come for the training mean while you have about 30 staff so if they are coming back you won’t also give them resources to commence training for the rest of the people so how do they get the information how do they cooperate with you. So it’s difficult.

I: so this SMC you keep referring to, SMC would mean?

P: that is seasonal malaria chemoprevention, that is suppose to be four months in a year and that is the peak season of malaria

I: so this SMC that you were referring to earlier, was it organized by the district or it was organized by your facility here

P: is SMC I think is organize it should be the whole region and my district is not in exception, so it was something that runs across the whole region the malaria peak season we all have to embarks on that.

I: so your focal person you talked about; the disease control officer is it this opportunity he took to talk about the adverse events that you talked about?

P: aaaha I don’t get that question please?

I: you earlier on talked of a training some months ago on adverse events where you said your focal person was involved, am asking if it was at that opportunity that he used to talk about the adverse events.

P: yes yes when they went for the training and came I then decided that, that was the second but last one we went to all the CHPS compounds under our catchment area, all staff was given some ample knowledge about it, so they should be aware, you understand so if you are not even aware and they have gone to be given if a child comes here will you cross check if he has been given or not given, so we took that opportunity to go to all facilities with him, I went with him to all the facilities and we select people who will be in-charge of adverse reactions in each of the facilities and their numbers were given to all the volunteers and anything at all they call them.

I: so that was your initiative?

P: yes

I: okay, alright, Mr. Solomon we are very grateful to you. Navrongo Health Research Centre thanks you and Ghana Health Service is proud of you.
