# Supplementary material for: Optimising reporting of adverse events following immunisation by healthcare workers in Ghana: A qualitative study in four regions
Source: PLoS One. 2022 Dec 20;17(12):e0277197. doi: 10.1371/journal.pone.0277197 (PMC9767370; doi:10.1371/journal.pone.0277197)
Supplement: Attachment — Submitted filename: Response to comments.docx [file pone.0277197.s002.docx]

Dear Editor

**Response to Reviewers’ Comments**

We have attempted to respond to all the comments of the reviewers and where necessary, revisions have been made to the manuscript in tracked changes to guide the review process.

We thank the editor and the reviewers for the insightful comments and we are happy to make any further revisions if necessary.

Please find below a point by point response to the comments.

1. Please ensure that your manuscript meets PLOS ONE's style requirements, including those for file naming. The PLOS ONE style templates can be found at

https://journals.plos.org/plosone/s/file?id=wjVg/PLOSOne_formatting_sample_main_body.pdf and

https://journals.plos.org/plosone/s/file?id=ba62/PLOSOne_formatting_sample_title_authors_affiliations.pdf

2. You indicated that ethical approval was not necessary for your study. We understand that the framework for ethical oversight requirements for studies of this type may differ depending on the setting and we would appreciate some further clarification regarding your research. Could you please provide further details on why your study is exempt from the need for approval and confirmation from your institutional review board or research ethics committee (e.g., in the form of a letter or email correspondence) that ethics review was not necessary for this study? Please include a copy of the correspondence as an "Other" file.

Response

The study was actually reviewed and approved by the CDC’s Human Subject’s Office, USA and the Ghana Health Service Ethical Review Committee. The statement under the section on “ethical approval” has been amended to read appropriately.

3. Please include additional information regarding the survey or questionnaire used in the study and ensure that you have provided sufficient details that others could replicate the analyses. For instance, if you developed a questionnaire as part of this study and it is not under a copyright more restrictive than CC-BY, please include a copy, in both the original language and English, as Supporting Information, or include a citation if it has been published previously.

Response

Under the data collection section, we indicated the tools that were used and how they were designed. It is our believe that these tools were only guides for data collection and therefore cannot be standardized for use globally. However, we have uploaded them for the purposes of the journal and any other interested party.

4. In the Methods, please discuss whether and how the questionnaire was validated and/or pre-tested. If these did not occur, please provide the rationale for not doing so.

Response

As indicated in our response above, qualitative data collection tools are only guides and therefore cannot be validated or standardized. Interviewers in qualitative research are allowed to deviate from the tool based on emerging themes to ask questions that were not deductively derived from the research objectives.

5. We note that you have indicated that data from this study are available upon request. PLOS only allows data to be available upon request if there are legal or ethical restrictions on sharing data publicly. For information on unacceptable data access restrictions, please see http://journals.plos.org/plosone/s/data-availability#loc-unacceptable-data-access-restrictions.

 The data are available at the data repository at the Navrongo Health Research Centre. The contact information for the individual responsible is as follows: peter.wontuo@navrongo-hrc.org

In your revised cover letter, please address the following prompts:

a) If there are ethical or legal restrictions on sharing a de-identified data set, please explain them in detail (e.g., data contain potentially identifying or sensitive patient information) and who has imposed them (e.g., an ethics committee). Please also provide contact information for a data access committee, ethics committee, or other institutional body to which data requests may be sent.

Response

We do not have such restrictions and so the data are available as indicated above.

b) If there are no restrictions, please upload the minimal anonymized data set necessary to replicate your study findings as either Supporting Information files or to a stable, public repository and provide us with the relevant URLs, DOIs, or accession numbers. Please see http://www.bmj.com/content/340/bmj.c181.long for guidelines on how to de-identify and prepare clinical data for publication. For a list of acceptable repositories, please see http://journals.plos.org/plosone/s/data-availability#loc-recommended-repositories.

We will update your Data Availability statement on your behalf to reflect the information you provide.

 Response

 An e-mail contact has been provided for data access.

6. Please include your tables as part of your main manuscript and remove the individual files. Please note that supplementary tables should be uploaded as separate "supporting information" files-


[Note: HTML markup is below. Please do not edit.]

Reviewers' comments:

Reviewer's Responses to Questions

**Comments to the Author**

Reviewer #1: Optimising reporting of adverse events following immunisation by healthcare workers in Ghana: A qualitative study in four regions

This is a useful study that explains some of the factors which result in low AEFI reporting rates in the districts sampled.

The following comments are minor;
1. Line 69 “All major and minor”
a. Suggest use standard terminology of “serious or severe” -

Response

The statement actually means all AEFIs should be reported. We have edited it to read appropriately. Thank you.

2. Line 91 In Ghana a national electronic
a. This will require more explanation as the flow of an AEFI involves many steps (as subsequently described) that are paper based and not electronic.
b. This could be better illustrated by a Figure to explain the flow of an AEFI report from a HCH to the national level with roles and responsibilities at each level.

Response

The flow has been described under the study site information. A diagram has also been provided to better capture the steps in the reporting system as suggested by the reviewer.

3. Line 99 FDA and EPI conducted training session…..
a. Is there any way of quantifying the participants who have undergone training?
b. This is important to understand this given the findings of the study which includes the lack of training for HCW

Response

We do agree with the thinking of the reviewer. Unfortunately the numbers are not available to authors.

4. Line 120, we conducted descriptive………….
a. What was the population coverage over these 4 regions or districts covered ?

Response

The combined population of 9,654,312 has been inserted in the manuscript and the source duly acknowledged.

5. Line 135. Caregiver(parent)
a. How would a caregiver report - could they report directly to the district or regional level, without the aid of a HCW?

Response

They could but the official at the district and regional levels would have to refer them to a health care worker, preferably, the one who vaccinated their wards so that monitoring the event or feedback could be done.

6. Line 139 “complaints”
a. This is an unusual term to be used in the AEFI context as these should be regarded as AEFI notifications.
b. Use of this term is relevant to one of the findings of the study as regarding these as “complaints” could be a barrier to reporting

Response

We do agree with the reviewer. The term has been replaced accordingly.

7. Line 137-153
a. Could be better represented in a Figure
b. This is discrepant with an “electronic system” that is described in a previous paragraph. This requires some explanation.

Response

As per the reviewer’s request in a previous comment, a diagram has been provided. Also, the electronic system does exist in isolation – it is part of the system and we have indicated the level of the reporting structure in which the electronic system is used.

8. Line 161 Sampling
a. Can the authors provide an indication of what percentage health facilities and districts were sampled (national facilities as the denominator)

Response

The data collection approach was qualitative and so determining the percentage of health facilities and districts was not of interest in the sampling approach. Our sampling was mainly informed by the category and number of interviewers who would ensure thematic saturation.

9. Line 164 HCW’s
a. These HCW had encountered an AEFI. Were they required to have reported the AEFI to be included ?

Response

They were required to have ENCOUNTERED an AEFI to be included. Whether they reported it or not was one of our key questions and the reasons behind non-reporting is the main focus of our paper.

10. Line 271
a. Could the authors indicate if “minor events or side effects if not serious are reportable as an AEFI, according to the guidelines in Ghana.

Response

Yes, as noted in the introduction, all AEFIs, regardless of their level of severity, are to be reported.

11. Line 291
a. Could the authors indicate if AEFI reporting is a core responsibility of HCW according to guidelines, protocols ?

Response

Yes it is a core responsibility of HCWs.

12. Line 532
a. Should community / caregiver education also occur so that an AEFI is not considered as a complaint by a caregiver?

Response

The health care worker education could change that perception. Recommendations to educate the community and caregivers are contained in another paper that focuses on interviews with caregivers and community leaders.

Reviewer #2: Thank you for this important qualitative study looking at AEFI reporting in the LMIC setting. Your data is comprehensive, and is important to present because of the paucity of similar data in the literature that has originated from the LMIC context. The following feedback is provided for you to consider how to improve the readability of the paper.

Response

We thank the reviewer for recognizing the importance of the theme that our paper focuses on.

I have some issues with the style of writing. I found descriptions overly wordy, and at times unclear. I think further editing to make the document more concise will assist in allowing your main points to come through better.

Response

We have closely edited the manuscript to make it more readable. We would have appreciated reference to specific lines and/or paragraphs that require our attention. Unfortunately this was not provided and so we did a general editing of the manuscript.

Methods
Specific areas that I would suggest be re-worked to tighten up your methods:
- AEFI reporting structure in Ghana. I wonder whether this would be better presented as a flowchart/figure.

Response

This has now been captured in a flow diagram.

- Sampling. Please clarify the role of the larger study in identifying your participants. I’m unsure if the 116 who participated in this study were from the 352 from reference 6, or whether you also included others to ensure representation across the AEFI reporting structures in Ghana.

Response

This is a very important question. Part of the sample was from the quantitative study (the HCWs who reported encountering an AEFI during the quantitative survey) but as you rightly indicated, we also sampled other cadres across the AEFI reporting structure. So for instance, the national and regional coordinators for EPI and FDA and staff of the RHMTS and DHMTs were not part of the quantitative sample but were purposively selected for key informant interviews because of the roles they play in the reporting structure. The section has been revised to read appropriately.

- Data collection. Overly descriptive. I am more interested in the actual questions that were asked in the interviews, and the prompts provided the interviewers – could this be included either as a figure or a supplement?

Response

The data collection tools have been uploaded into the submission system and we hope the journal will make them available to the reviewers. Broadly, the tools asked questions on the AEFI surveillance system, the training of health workers on the surveillance system, barriers to the surveillance system and suggestions to improve the system. This explanation is provided in the second but last paragraph under that section.

- Ethical approval. Paragraph could be truncated.

Response

We think all the information in that section is relevant as it relates to the procedures that we followed in order to ensure that the rights of our participants were ensured. We are willing to truncate the section if the editor deems it necessary.

Additional areas where grammar could be addressed:
- L 36 – 45. Sentence structure requires more work. Maybe requires shortening, or use of semicolons.

Response

This is summary of the findings of we feel all the information there is necessary. We have done some edits to make it more readable.

- L 125. There is a stray parenthesis

Response

Thank you. Corrected.

Results
- There may be value in presenting your major themes in a table.

Response

It is not clear what the reviewer means by presenting major teams in tables. We think the current descriptions and narratives in prose conveys succinctly the results to our readers. We are willing to revise this if the reviewer thinks otherwise.

- You make a point of collecting data across the spectrum of staff involved in the AEFI reporting process (which is fantastic). I’m interested to know if there was a difference in the themes between the staff at these levels. Could you compare and contrast this data?

Response

We do appreciate this comment from the reviewer. In analyzing the data, our interest was in normative patterns that cut across the different categories of interviewers. The different respondents operate at different levels and therefore their focus in sharing information was mostly what happens at their level. This did not allow for many contradictions or opposing views. The few that were identified especially regarding training were acknowledged in the presentation of the findings. We are happy to revise these if the comment could be more specific.

Discussion
- It is interesting, albeit not surprising, that developed countries have similar themes when it comes to AEFI reporting. I wonder if there could be greater discussion around cost and resources as a factor, and consideration of the cost/resource-effective measures that improve AEFI reporting generally.

Response

Regardless of the reporting format, cost is incurred in the reporting of AEFIs. Either facilities spend on paper, printing and photocopying or on computers, phones and internet data.

- I was interested by the comment in the results regarding the benefits of phone reporting, particularly regarding the immediacy of feedback (and likely the benefit of actual human interaction!). As we move towards more automated systems – Apps, SMS etc – which have obvious benefits, there may be a chance here to reflect/expand on the pros/cons of the different technologies in the discussion more. Some technology is likely better than the current situation, but too much may not deliver the necessary results either, and may come at significant cost. There are a few articles in the literature which review novel solutions and may be worthwhile to review:
o Tsafack M, Ateudjieu J. Improving community based AEFI (adverse events following immunisation) reporting rate through telephone “beep” in a Cameroon health district: a randomized field trial. Pan Afr Med J 2015;22:351–57;
o Leeb A, Regan AK, Peters IJ et al. Using automated text messages to monitor adverse events following immunisation in general practice. Med J Aust 2014;200:416–18;
o Willson K, Atkinson KM, Westeinde J et al. An evaluation of the feasibility and usability of a proof of concept mobile app for adverse event reporting post influenza vaccination. Hum Vacc Immunother2016;12:1738–48

Response

We thank the review for the suggested readings. We have included a paragraph in the discussion that focuses on the use of electronic reporting systems at the community level.

- L 542 – 543. Needs a reference.

Response

Inserted.

- L 551 – 553. Has integration of AEFI into routine facility/district reports been done in other LMIC areas? If so, with any success, or is this just adding to the cumbersome aspects of AEFI reporting?

Response

As frontline providers of vaccines and the intermediaries between the health system and the vaccine recipients or their guardians, reporting of AEFIs should be part of the routine process but this is currently lacking in Ghana’s health care system. The paragraph has been amended to read more appropriately.

Referencing
There are multiple errors, with typos and absent journal/publisher names affecting references 7, 8, 9, 10, 25, 26, 28 and 34.

Response

Thank you for the critical review. We have fixed the references.

Additional
It wasn’t clear to me how to access your data, in keeping with the PLOS Data policy. Could you provide a url for your data repository, or address this in the data availability section of your submission.

 Response

This has now been provided.

We thank the reviewers for their useful comments. We think the comments have helped to improve the quality of the manuscript.
