# Supplementary material for: Optimising reporting of adverse events following immunisation by healthcare workers in Ghana: A qualitative study in four regions
Source: PLoS One. 2022 Dec 20;17(12):e0277197. doi: 10.1371/journal.pone.0277197 (PMC9767370; doi:10.1371/journal.pone.0277197)
Supplement: Attachment — Submitted filename: Rebuttal letter.docx [file pone.0277197.s003.docx]

Re: PONE-D-21-25213

Optimising reporting of adverse events following immunisation by healthcare workers in Ghana: qualitative study in four regions

PLOS ONE

Dear Dr. Aborigo,

Thank you for submitting your manuscript to PLOS ONE. After careful consideration, we feel that it has merit but does not fully meet PLOS ONE’s publication criteria as it currently stands. Therefore, we invite you to submit a revised version of the manuscript that addresses the points raised during the review process.

Look  that there are some revision to do, but after done the manuscript may be accepted.

Please submit your revised manuscript by Sep 15 2022 11:59PM. If you will need more time than this to complete your revisions, please reply to this message or contact the journal office at [plosone@plos.org](mailto:plosone@plos.org). When you're ready to submit your revision, log on to https://www.editorialmanager.com/pone/ and select the 'Submissions Needing Revision' folder to locate your manuscript file.

Please include the following items when submitting your revised manuscript:

- A rebuttal letter that responds to each point raised by the academic editor and reviewer(s). You should upload this letter as a separate file labeled 'Response to Reviewers'.
- A marked-up copy of your manuscript that highlights changes made to the original version. You should upload this as a separate file labeled 'Revised Manuscript with Track Changes'.
- An unmarked version of your revised paper without tracked changes. You should upload this as a separate file labeled 'Manuscript'.

If you would like to make changes to your financial disclosure, please include your updated statement in your cover letter. Guidelines for resubmitting your figure files are available below the reviewer comments at the end of this letter.

If applicable, we recommend that you deposit your laboratory protocols in protocols.io to enhance the reproducibility of your results. Protocols.io assigns your protocol its own identifier (DOI) so that it can be cited independently in the future. For instructions see: [https://journals.plos.org/plosone/s/submission-guidelines#loc-laboratory-protocols](https://journals.plos.org/plosone/s/submission-guidelines#loc-laboratory-protocols). Additionally, PLOS ONE offers an option for publishing peer-reviewed Lab Protocol articles, which describe protocols hosted on protocols.io. Read more information on sharing protocols at [https://plos.org/protocols?utm_medium=editorial-email&utm_source=authorletters&utm_campaign=protocols](https://plos.org/protocols?utm_medium=editorial-email&utm_source=authorletters&utm_campaign=protocols).

We look forward to receiving your revised manuscript.

Kind regards,

Ricardo Q. Gurgel, PhD
Academic Editor
PLOS ONE

Journal Requirements:

When submitting your revision, we need you to address these additional requirements.

1. Please ensure that your manuscript meets PLOS ONE's style requirements, including those for file naming. The PLOS ONE style templates can be found at 
https://journals.plos.org/plosone/s/file?id=wjVg/PLOSOne_formatting_sample_main_body.pdf and 
https://journals.plos.org/plosone/s/file?id=ba62/PLOSOne_formatting_sample_title_authors_affiliations.pdf

RESPONSE: We have done that.

2. We note that the grant information you provided in the ‘Funding Information’ and ‘Financial Disclosure’ sections do not match.

When you resubmit, please ensure that you provide the correct grant numbers for the awards you received for your study in the ‘Funding Information’ section.

Response: The senior author received the grant from CDC but the grant number was never provided as the lead institution in the implementation of the research were sub-recipients.

3. Thank you for stating the following in the Acknowledgments Section of your manuscript: "We would like to thank in-country stakeholders, including EPI, the Ghana Food and Drugs Authority, Navrongo Health Research Centre, and the African Collaborating Centre for Pharmacovigilance. We also would like to thank the data collectors who worked tirelessly under difficult field conditions during data collection. Additionally, we greatly appreciate Mohamed Jalloh, Abigail Shefer, David Fitter, and Sureyya Hornston’s critical review of this qualitative manuscript.

The research was supported by the Centers for Disease Control and Prevention/the Agency for Toxic Substances and Disease, Atlanta, USA."

We note that you have provided funding information that is not currently declared in your Funding Statement. However, funding information should not appear in the Acknowledgments section or other areas of your manuscript. We will only publish funding information present in the Funding Statement section of the online submission form. 
Please remove any funding-related text from the manuscript and let us know how you would like to update your Funding Statement. Currently, your Funding Statement reads as follows: "No -The funders had no role in study design, data collection and analysis, decision to publish, or preparation of the manuscript."

Please include your amended statements within your cover letter; we will change the online submission form on your behalf.

Response: We have deleted the funding information in the acknowledgement section. The only role played by the funder was the provision of funding.

- Initials of the authors who received each award: **RAA**
- Grant numbers awarded to each author - **Subcontract No: AF-NRC001/2019**
- The full name of each funder: **Centers for Disease Control and Prevention/the Agency for Toxic Substances and Disease, Atlanta, USA, Through the African Field Epidemiology Network (AFINET)**
- URL of each funder website: <https://www.cdc.gov>
- Did the sponsors or funders play any role in the study design, data collection and analysis, decision to publish, or preparation of the manuscript?
  - **YES** - Specify the role(s) played.: They participated in study design and in the decision t*o publish*

4. In your Data Availability statement, you have not specified where the minimal data set underlying the results described in your manuscript can be found. PLOS defines a study's minimal data set as the underlying data used to reach the conclusions drawn in the manuscript and any additional data required to replicate the reported study findings in their entirety. All PLOS journals require that the minimal data set be made fully available. For more information about our data policy, please see http://journals.plos.org/plosone/s/data-availability.

Upon re-submitting your revised manuscript, please upload your study’s minimal underlying data set as either Supporting Information files or to a stable, public repository and include the relevant URLs, DOIs, or accession numbers within your revised cover letter. For a list of acceptable repositories, please see http://journals.plos.org/plosone/s/data-availability#loc-recommended-repositories. Any potentially identifying patient information must be fully anonymized.

Important: If there are ethical or legal restrictions to sharing your data publicly, please explain these restrictions in detail. Please see our guidelines for more information on what we consider unacceptable restrictions to publicly sharing data: http://journals.plos.org/plosone/s/data-availability#loc-unacceptable-data-access-restrictions. Note that it is not acceptable for the authors to be the sole named individuals responsible for ensuring data access.

We will update your Data Availability statement to reflect the information you provide in your cover letter.

Response: The minimal underlying data set has been uploaded as Supporting Information.

5. Please upload a new copy of Figure 1 as the detail is not clear. Please follow the link for more information: https://blogs.plos.org/plos/2019/06/looking-good-tips-for-creating-your-plos-figures-graphics/" <https://blogs.plos.org/plos/2019/06/looking-good-tips-for-creating-your-plos-figures-graphics/>

Response: A clearer figure was uploaded to PACE and was deemed ok. We have therefore uploaded the new figure.

6. Please include your tables as part of your main manuscript and remove the individual files. Please note that supplementary tables (should remain/ be uploaded) as separate "supporting information" files"

Response: The table has been included in the main manuscript.

7. Please include captions for your Supporting Information files at the end of your manuscript, and update any in-text citations to match accordingly. Please see our Supporting Information guidelines for more information: <http://journals.plos.org/plosone/s/supporting-information>.

Response: This has been done.

8. Please review your reference list to ensure that it is complete and correct. If you have cited papers that have been retracted, please include the rationale for doing so in the manuscript text, or remove these references and replace them with relevant current references. Any changes to the reference list should be mentioned in the rebuttal letter that accompanies your revised manuscript. If you need to cite a retracted article, indicate the article’s retracted status in the References list and also include a citation and full reference for the retraction notice.

Response: This has been done.


Reviewers' comments:

Reviewer's Responses to Questions

5. Review Comments to the Author

Please use the space provided to explain your answers to the questions above. You may also include additional comments for the author, including concerns about dual publication, research ethics, or publication ethics. (Please upload your review as an attachment if it exceeds 20,000 characters)

Reviewer #1: Dear authors. The research carried out with a broad character on vaccine notification is valid, relevant, necessary and very interesting. It would only reduce the number of keywords. The introduction is well written, relating to the topic. It's good, comprehensive, and complete. The materials and methods, the description is a little confusing, although it has a table that makes it a little easier. The part that refers to the data collection technique needs to be better clarified. the data analysis refers to the use of NVivo software for qualitative research and the presence of two judges. Discussions in a separate session of the results were made a synthesis. very interesting needed in qualitative work, although here somewhat repetitive

Please be advised that out of consideration for the authors, if we do not receive your review or a response within the next three days, we must assume that you are not available to complete this assignment and will remove it from your account. The Academic Editor will then proceed to render a decision based on previously secured reviews and their own expertise. If it is your intention to submit your review, please do let us know.

Reviewer #2: Reviewer's comments
1. Line 64: kindly amend the sentence.

Response: We thank the reviewer for catching the typo. The sentence has been edited appropriately.

2. Line 103: Reference should be properly placed.

Response: The reference has been placed appropriately.

3. References needs to be correctly inserted. For example, references 10-13.

Response : This has been done

4. How were the districts chosen?

Response: Under the section on sampling we stated that “In each region, we randomly selected five districts and conducted KIIs with two members of the DHMT (the public health nurse and the health information officer) and the EPI coordinator who, in most cases, was the disease control officer”.

5. Line 140 should be proofread and edited.

Response: The sentence has been edited appropriately. We thank the reviewer for pointing this out.

6. Line 141: “According to the FDA guidelines, a suspicion alone is enough grounds for 142 reporting.” This should be referenced

Response: The reference has been inserted

7. Generally, four levels 145 of reporting are used during mass immunization campaigns. These are the health 146 worker, the district, the regional and the national levels. These need to be adequately broken down as it is confusing in its present state. Can an AEFI report from the region? Quite unlikely!

Response: The sentence has been edited appropriately. We intended capturing the flow of information on AEFIs related to mass immunization campaigns.

8. Line 154: spelling of vaccinee is wrong. Kindly correct it

Response: This has been corrected.

9. Inconsistent capitalization of proper noun

Response: This has been corrected. Thanks for catching that.

10. Is the Regional Focal Person the same the Regional EPI Coordinator? There should be consistency in the use of these designations.

Response: We have the Regional EPI Coordinator and the FDA Regional Focal Person. These are two different designations. There is no such position as Regional Focal Person.

11. The manuscript should be properly formatted. For example, see page 12.

Response: This has been done

12. “Data 240 collectors conducted 116 KIIs from December 2017 to January 2018 with respondents 241 at their work-places in four regions.” Why these large number of respondents for a qualitative survey keeping in mind the concept of saturation?

Response: We do recognize that some of the numbers were too large and could have been reduced without compromising the quality of our data or attaining thematic saturation. However, you will realize that we had different categories of respondents who were spread across a wide range of locations and we were interested in triangulating across data sources and to explore geographical variations that may be related to AEFI reporting. These factors contributed to the large numbers.

13. Discussion: comparison was not made with studies conducted within the subregion or elsewhere regarding the understanding or knowledge of AEFI by the health workers and immunization manager. You may check out this study: Omoleke et al. BMC Health Services Research (2022) 22:741 <https://doi.org/10.1186/s12913-022-08133-9>

Response: Thank you for the reference. We have referenced the paper in our manuscript.

14. “Some studies have shown that HCWs believe that only proven adverse events should be reported”. What does the author mean by proven adverse events?

Response: Adverse events that HCWs know to be related to the vaccine. The word proven has been replaced appropriately.

15. “Re-orienting HCWs to think differently about AEFI 581 reporting will require focused training with an understanding of adult learning techniques and clear case definitions.” This assertion could also be substantiated by findings from the recently published study from Nigeria that found suboptimal understanding of AEFI about health workers and called for a regular refresher training on the subject.

Response: This reference has been added.

16. Again, the referencing for the entire manuscript needs to be reworked. Ogunyemi et al was cited twice- reference number 31 and 33.

Response: The references have been reworked as suggested.

17. “The participation of primary health care nurses 608 in reporting AEFIs will also improve patient safety and reduce cost associated with AEFIs[37]” Why singling out community health nurses when the authors had clearly mentioned elsewhere that CHO are mainly responsible for implementing Ghana PHC flagship programme at the health facility level?

Response: They are the frontline providers for immunizations but they are currently missing in the reporting system. Also, they are the closest to the clients and therefore accessible when clients want to report AEFIs. They are therefore better positioned to receive and report AEFIs.

18. Line 648: New vaccine introductions could serve as avenues for additional training for frontline HCWs on AEFI. This was not completed- how does it affect motivation of the HCW to report?

Response: The preceding sentence which reads “Even participation in training workshops, which could serve as motivation is not afforded to frontline HCWs who are the primary reporters of AEFIs” explains how training opportunities motivate HCWs.

19. Line 670 “Immunisation is an effective way of saving lives”- this should be re-written.

Response: This has been revised appropriately.

We thank the reviewers for their useful feedback, and we are happy to address any further queries that they may have.

Sincerely,

Raymond Akawire Aborigo, Ph.D
